# Electroreductive amination of carboxylic acids by cobalt catalysis

**DOI:** 10.1038/s41467-025-62396-4

**Published:** 2025-08-04

**Authors:** Huihua Bi, Zhizheng Chen, Changsheng Bi, Shuanglin Qu, Jie Liu

**Affiliations:** 1 College of Chemistry and Chemical Engineering, State Key Laboratory of Chemo and Biosensing, Hunan University, Changsha, China; 2 Greater Bay Area Institute for Innovation, Hunan University, Guangzhou, China

**Keywords:** Synthetic chemistry methodology, Electrocatalysis

## Abstract

Catalytic reduction of carboxylic acids to valuable chemicals is highly desirable yet challenging for both biomass conversion and organic synthesis. Here we describe an efficient and sustainable electrocatalytic hydrogenation of carboxylic acids with amines utilizing protons as the hydrogen source. The application of an earth-abundant cobalt complex enables electrochemical generation of a cobalt-hydride intermediate, which serves as the key catalytically active species for this reductive process. Obviating the need for flammable H_2_ gas or sensitive hydrides, this general hydrogenative coupling of carboxylic acids with amines and nitroarenes allows producing a wide range of structurally diverse complex alkylamines under mild electrocatalytic conditions. Furthermore, the practicality and versatility of this protocol are demonstrated through its application in valuable isotope labeling using readily available deuterium sources.

## Abstract (web summary)

Catalytic reduction of carboxylic acids to valuable chemicals is highly desirable yet challenging for both biomass conversion and organic synthesis. Here the authors describe an efficient and sustainable electrocatalytic hydrogenation of carboxylic acids with amines utilizing protons as the hydrogen source.

## Introduction

**Fig. 1:**
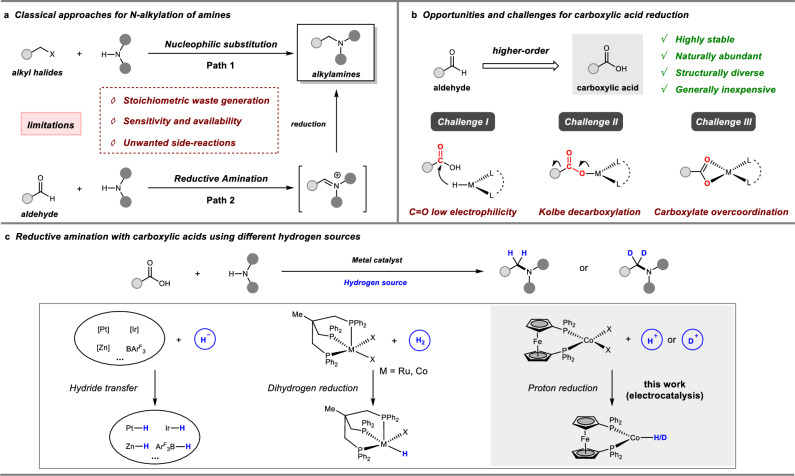
Introduction of *N*-alkylation of amines using different alkylating reagents and hydrogen sources. **a** Classical methods for *N*-alkylation of amines. **b** Opportunities and challenges for carboxylic acid reduction. **c** Reductive amination of carboxylic acids using different hydrogen sources.

Direct *N*-alkylation of amines has long-standing interest to synthetic chemists, as the resulting products are highly valuable bulk and fine chemicals such as pharmaceutical agents, agrochemicals, dyes, and natural products^1^. Conventional nucleophilic substitution approach to *N*-alkylated amines mainly relies on the use of alkyl halides as alkylating reagents (Fig. 1a)^2^. However, it suffers from the limitations of stoichiometric amounts of halide waste generation and poor chemoselectivity due to the undesired overalkylations. Although carbonyl reductive amination with aldehydes or ketones has been the benchmark method^3,4^, in some cases, their availability, sensitivity, and unwanted side reactions such as aldol condensations still restrict their applications. Carboxylic acids represent an important class of carbonyl compounds that are naturally abundant and structurally diverse from biomass feedstock^5^. The *N*-alkylation of amines using carboxylic acids, a higher-order variant of classical reductive aminations, provides an attractive alternative for the streamlined synthesis of complex alkylamines (Fig. 1b)^6,7^. Pioneered by Gribble and Marchini, the plausibility of reductive alkylation of amines with carboxylic acids was first demonstrated in the 1970s^8,9^. However, the requirement for relatively harsh reaction conditions and superstoichiometric metal hydrides (H^−^) significantly limited the scope of these early methods. Subsequently, a surge of synthetically useful methods have been developed with a diversity of catalytic systems, ranging from the noble metals^10–13^ to base metals^14–17^ even main group elements^18–20^ in combination with hydrosilanes as terminal reductants (Fig. 1c). More recently, Cole-Hamilton^21^, Beller^22,23^, and Sundararaju^24^ demonstrated the feasibility of this transformation using molecular hydrogen (H_2_) in the presence of Ru or Co/triphos catalysts. Despite these elegant advances, the utilization of sensitive hydrides or pressurized H_2_ gas as hydrogen sources might lead to potential security risk, as well as elaborate autoclave manipulation, which restricts their specific utility in synthetic chemistry.

A rising trend in catalytic hydrogenation is the harness electricity as a renewable energy source for green and sustainable synthesis^25–33^. Complementary to classical thermal hydrogenations, electrohydrogenation directly employs protons (H^+^) and electrons (e^−^) as the hydrogen source and redox equivalent to substitute for traditional molecular hydrogen (H_2_) or hydride (H^−^) donors. Related to this strategy, the electrohydrogenation of various unsaturated bonds, such as carbonyl compounds, has received widespread attention^34–37^. The early examples of electrocatalytic hydrogenation of carbonyl compounds mainly focused on the reduction of relatively high reactive aldehydes and ketones^38–41^. We recently reported selective and efficient electrohydrogenation of less electrophilic nitriles to secondary and tertiary amines catalyzed by a cobalt bipyridine complex^42^. However, to the best of our knowledge, the electroreduction of more challenging carboxylic acids has seriously lagged behind related reductions of other carbonyl compounds (Fig. 1b). This is due to the low electrophilicity and thermodynamic stability of the carboxyl group, which renders it less reactive and difficult to reduce, therefore a high negative reductive potential is required^43,44^. Additionally, the Kolbe decarboxylation as a side reaction also competes with the desired carbonyl reduction process^45–47^. Moreover, the interaction of carboxylic acids with the metal catalyst, such as carboxylate overcoordination, may result in detrimental catalyst deactivation^48–50^. Consequently, it is highly desirable to develop a general and robust electrocatalyst system that can reduce carboxylic acids to value-added chemicals, considering their conceivable advantages of natural availability and structurally diversity.

In light of these challenges and opportunities, we herein report a mild and efficient electroreductive amination with carboxylic acids catalyzed by an earth-abundant cobalt complex (Fig. 1c). By utilizing protons as the hydrogen source, this method enables direct *N*-alkylations, including trifluoroethylation and methylation, to access a broad range of complex amines derived from carboxylic acids. Particularly, a key advantage of this electrocatalytic transformation is the feasible and valuable divergent incorporation of deuterium (D) into the resulting amines from readily available deuterated acids.

## Results

### Optimization reaction conditions

At the beginning of this study, a bench-stable, inexpensive, and bulk trifluoroacetic acid (TFA) was selected as the model substrate, as the resulting trifluoroethylation products are highly attractive in medicinally relevant fluorinated building blocks^51^. Compared to volatile trifluoroacetaldehyde or expensive trifluoroethylation reagents, TFA offers a cost-effective and practical alternative for synthesizing valuable β-fluoroalkylamines. Furthermore, as the simplest and ultrashort-chain perfluoroalkyl substance, the efficient and sustainable conversion of TFA into value-added chemicals has garnered significant attention^52^. To explore this potential, we investigated the reaction parameters for the electrocatalytic hydrogenative coupling of TFA **1** with 4-phenylaniline **2** (Table 1). Based on previous studies^53–55^, a key intermediate cobalt-hydride can be generated by cathodic reduction of a cobalt (II) precatalyst and followed by protonation in an acidic medium. Therefore, we evaluated the electrocatalytic performance of cobalt complexes with various ligands. We were pleased to obtain the desired β-fluorinated amine **3** in 93% yield using the commercially available diphosphine dppf (**L1**). Notably, the transformation exhibited good chemoselectivity, as neither reductive defluorination byproducts nor dialkylated species were observed. Other bidentate phosphines such as dppe (**L2**) and binap (**L3**) exhibited moderate reactivity in the model reaction, with the formation of a corresponding amide as a side product. The monodentate as well as tridentate phosphine ligands such as PPh_3_ (**L4**) and CH_3_C(CH_2_PPh_2_)_3_ (**L5**) also resulted in lower yields. Additionally, bipyridine (**L6**) and other ligands (Supplementary Table 1) did not improve the yield of the *N*-trifluoroethylation product. With the optimal ligand **L1** identified (Table 1, entry 1), we further optimized other reaction parameters. While Co(OTf)_2_ provided the best results, other cobalt salts yielded slightly lower efficiencies, and non-noble metal salts such as Fe or Ni failed to produce the desired product (entries 2 and 3). The choice of Lewis acid additive proved critical for effective carbonyl group activation. For instance, ZnCl_2_ or BF_3_·Et_2_O instead of Ti(O^*n*^Bu)_4_ did not give better results in this reaction (entry 4). When the reactions were performed at higher or lower temperatures, decreasing yields were observed (entry 5). As to solvent, the use of MeOH or THF suppressed the reaction completely (entry 6). Other sacrificial anodes, such as Mg, Al, or Fe, were inactive under these electrochemically conditions (entry 7). As comparison, stoichiometric reductants like Zn, Mn, Mg dust, PhSiH_3_^56^, or atmospheric H_2_ gas, used in place of electrolysis, showed very low or no catalytic activity in the model reaction (entries 8–10). Control experiments confirmed that the cobalt catalyst was essential, and Ti(O^*n*^Bu)_4_ significantly promoted the reaction (entries 11 and 12).

**Table 1:** Effects of reaction parameters^a^


Entry	Deviation from standard conditions with L1 ligand	Yield of 3^b^
1	None	93
2	CoCl_2_, CoBr_2_ or Co(OAc)_2_ instead of Co(OTf)_2_	85, 82, 90
3	FeCl_2_ or NiCl_2_ as a catalyst	0, 0
4	ZnCl_2_ or BF_3_·Et_2_O instead of Ti(O^*n*^Bu)_4_	78, 73
5	60 °C or 80 °C instead of 70 °C	69, 74
6	MeOH or THF as solvent	0, 0
7	Mg (+), Al (+) or Fe (+) instead of Zn (+)	0, 0, 0
8	Zn, Mn, or Mg dust instead of electrolysis	14, 0, 0
9	PhSiH_3_ instead of electrolysis	0
10	1 bar H_2_ instead of electrolysis	0
11	No catalyst	0
12	No Ti(O^*n*^Bu)_4_	77

^a^Reaction condition: trifluoroacetic acid **1** (4.0 mmol), 4-phenylaniline **2** (0.2 mmol), Co(OTf)_2_ (0.02 mmol, 10 mol%), L1 (0.02 mmol, 10 mol%), Ti(O^*n*^Bu)_4_ (0.2 mmol), MeCN (2.0 mL), toluene (2.0 mL) in an undivided cell with zinc cathode and anode, constant current 20 mA, 70 °C, 3 h, N_2_.

^b^NMR yield using CH_2_Br_2_ as an internal standard.

### Substrate scope

**Fig. 2:**
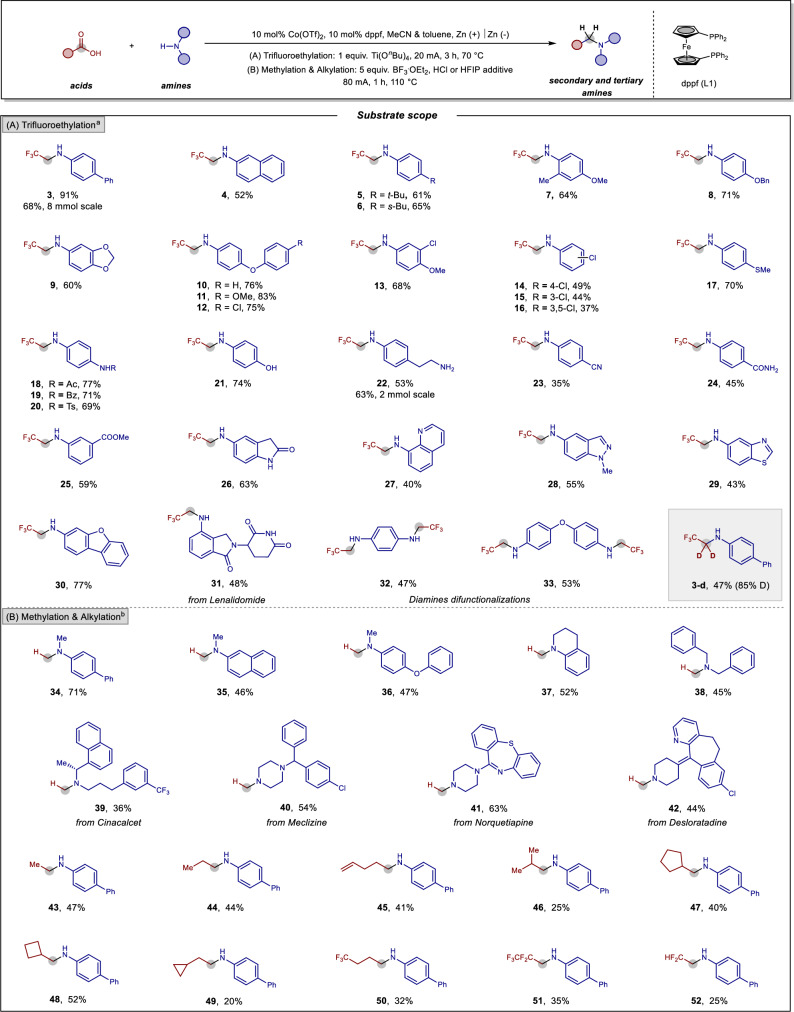
Substrates scope of *N*-trifluoroethylations, methylations, and alkylations of amines with carboxylic acids. ^a^ Reaction condition (**A**) as shown in Table 1. ^b^ Reaction condition (**B**): HCOONa or carboxylic acid (2.0 mmol), amine (0.2 mmol), Co(OTf)_2_ (10 mol%), L1 (10 mol%), BF_3_·Et_2_O (1.0 mmol), HCl (2.4 mmol, for methylation) or HFIP (0.2 mL, for alkylations), MeCN and toluene in an undivided cell with zinc cathode and anode, 80 mA, 110 °C, 1 h, N_2_.

With the optimal reaction conditions established, we explored the substrate scope of this cobalt-electrocatalytic hydrogenative transformation (Fig. 2). Initially, reductive *N*-trifluoroethylations using TFA were performed to access a variety of structurally diverse β-fluoroalkylamines (Fig. 2A). In addition to the model substrate 4-phenylaniline, 2-naphthylamine and anilines bearing alkyl, alkoxy, and aryloxy substituents all reacted efficiently, yielding fluorinated amines **4–12** in moderate to good yields (52–83%). A critical issue in hydrogenative reaction is the challenging but highly desirable chemoselectivity of diverse functional groups. Remarkably, anilines containing chlorine, trifluoromethyl, thioether, and sulfonamide functionalities underwent the transformation smoothly, providing synthetically useful yields of products **13–20**. Notably, substrates with free hydroxyl and amino groups were well-tolerated without the need for pre-protection, yielding the corresponding products **21** and **22**. A 2 mmol-scale reaction using 4-(2-aminoethyl)aniline as the substrate afforded product **22** in 63% yield. Under these electrohydrogenative conditions, sensitive and reducible functional groups such as nitrile, ester, and amide were also compatible, and their successful conversion to β-fluoroalkylamines **23–26** further expanded the reaction scope. Additionally, heteroaromatic amines, including quinoline, indazole, benzothiazole, and dibenzofuran, proved to be effective coupling partners, delivering the corresponding products **27–30** in 40–77% yields. Lenalidomide, an immunomodulatory drug used to treat multiple myeloma and anemia, furnished the desired product **31** in 48% yield. Furthermore, diamines reacted smoothly, providing the corresponding dialkylated amines **32** and **33** in 47% and 53% yields, respectively. While alkylamines such as benzylamine or morpholine failed to undergo the desired *N*-alkylations, this method offered unique advantages in isotopic labeling. By substituting CF_3_COOD instead of CF_3_COOH^57^, we achieved direct deuterium incorporation into β-fluorinated amine **3-d**. This transformation enables the direct introduction of two important functional groups, CF_3_ and D, to an amine in catalytically one-pot process, highlighting the synthetic utility of this method.

Next, we turned our attention to the scope in terms of other less electrophilic carboxylic acids, including formic acid and aliphatic carboxylic acids (Fig. 2B). Installing a “magic methyl” group into molecules is highly desirable in modern drug discovery and chemical synthesis^58–62^. Among various methylation reagents, formic acid (HCOOH) stands out as one of the most convenient, non-toxic, easily manipulated, and readily available C1 sources, derived from biomass fermentation or CO_2_ reduction^63,64^. In many cases, the activation and reduction of less electrophilic carboxylic acids often require strong Brønsted or Lewis acid additives^65^. We were pleased to discover that BF_3_·OEt_2_, rather than Ti(O^*n*^Bu)_4_, served as the optimal Lewis acid additive for the electroreduction of aliphatic carboxylic acids. With this additive, the molecular cobalt-electrocatalyst system enabled efficient *N*-methylation of diverse amines **34–38** using HCOOH or HCOONa as the methyl source. To further demonstrate the synthetic utility of this methylation method, we applied it to the direct functionalization of several natural products and pharmaceutical molecules. For instance, Cinacalcet **39**, Meclizine **40**, Norquetiapine **41**, and Desloratadine **42** underwent this methylating transformation efficiently, highlighting the compatibility of the electroreductive protocol and its potential utility in late-stage functionalization. In addition to TFA and HCOOH, we also explored electrohydrogenative alkylations with less electrophilic aliphatic carboxylic acids. A representative set of chain and cyclic aliphatic carboxylic acids bearing olefinic and perfluoroalkyl functionalities proved compatible with this reductive coupling, yielding the corresponding alkylated anilines **43–52** in moderate yields, whereas substituted quinolines as the side products were observed through the Doebner von Miller pathway (Supplementary Fig. 8). Notably, aromatic acids showed poor conversion under these electroreductive conditions. Despite this limitation, the protocol offers a sustainable alternative to classical Eschweiler-Clarke reaction and reductive amination method, eliminating the need for toxic formaldehyde or unstable aldehydes.

### Synthetic applications

**Fig. 3:**
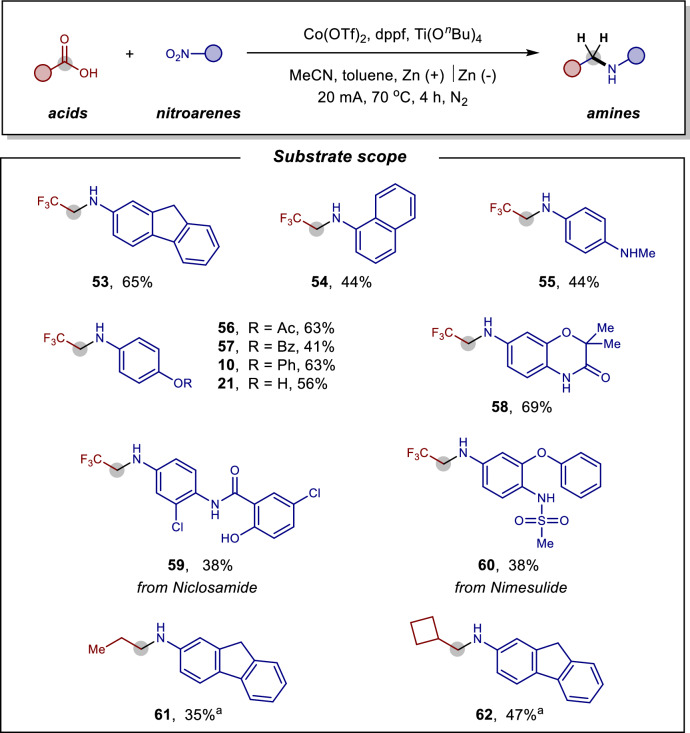
Examples of electrocatalytic reductive amination of carboxylic acids with nitroarenes. ^a^ 5 equiv. BF_3_·Et_2_O instead of Ti(O^*n*^Bu)_4_, HFIP (0.2 mL), 40 mA, 100 °C, 3 h.

Subsequently, we explored the electroreductive *N*-alkylation of readily available and inexpensive nitroarenes, given that most anilines are derived from their corresponding nitroarene precursors (Fig. 3). This hydrogenative coupling strategy with nitroarenes not only eliminates at least one synthetic step but also leverages bulk and cost-effective starting materials. Using TFA as the carboxylic acid substrate, the cobalt-electrocatalytic trifluoroethylation of various substituted nitroarenes proceeded smoothly, yielding the desired products **53–58** in 41–69% yields. Notably, pharmaceutical molecules such as Niclosamide **59** and Nimesulide **60** also performed comparably well under these conditions. A variety of reducible or sensitive functional groups in these molecules, including hydroxyl, ether, chlorine, and amide functionalities, were tolerated. Furthermore, aliphatic carboxylic acids, such as propionic acid and cyclobutanecarboxylic acid, were successfully converted into their corresponding *N*-alkylated products **61** and **62**. By eliminating the need for pre-reducing nitroarenes to anilines, this electrochemical approach demonstrates remarkable versatility and practicality, enabling a convenient and economical one-pot synthesis of *N*-alkylamines directly from commercially accessible nitroarenes.

**Fig. 4:**
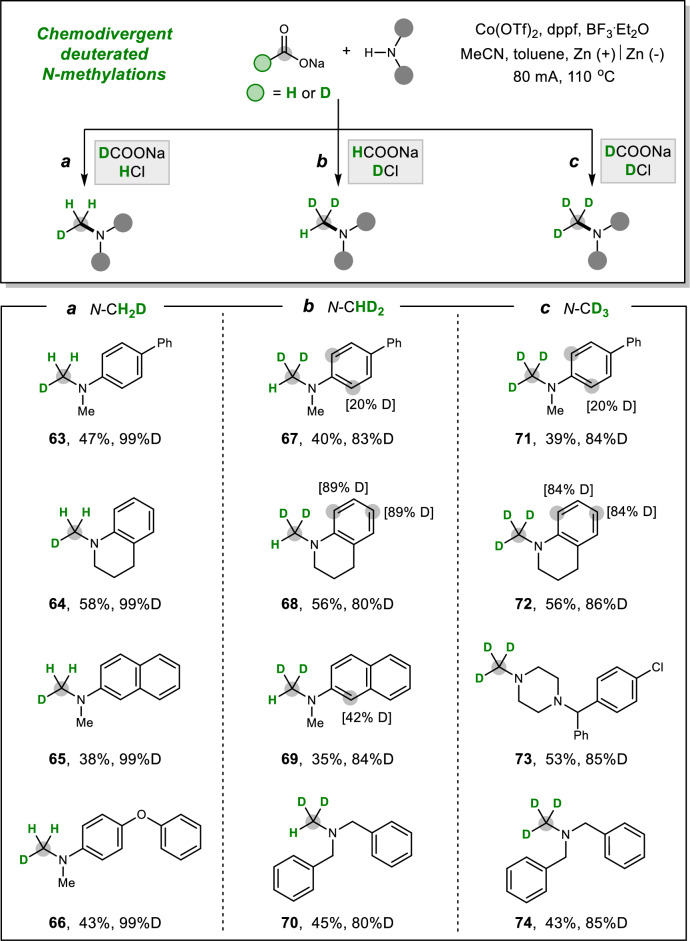
Electrocatalytic chemodivergent deuterated *N*-methylation of amines using isotopic formic acids. **a** Installation of *N*-CH_2_D groups using DCOONa and HCl. **b** Installation of *N*-CHD_2_ groups using HCOONa and DCl. **c** Installation of *N*-CD_3_ groups using DCOONa and DCl.

Organic molecules labelled with hydrogen isotopes have garnered considerable attention due to their potential applications in pharmaceutical discovery and radiation chemistry^66–69^. Traditional reductive deuteration processes often rely on expensive and non-recoverable D_2_ gas or stoichiometric deuteride reagents. Consequently, the development of cost-effective and readily accessible deuterium sources, such as deuterated protons (D^+^), would be highly desirable and demanding. In this work, we present an electrocatalytic approach for the incorporation of deuterium (D) at the α-positions of amines (Fig. 4). Remarkably, we achieved a practical and chemodivergent deuteration of *N*-methylation by strategically tuning the isotopic formates (HCOONa or DCOONa) and protic acids (HCl or DCl) as hydrogen (H) or deuterium (D) sources. Using this method, three distinct deuterium-labeled amines (–CH_2_D, –CHD_2_, –CD_3_) were obtained in a selective manner. For instance, the d^1^-methylated amines **63–66** were efficiently synthesized with high deuterium incorporation by employing DCOONa as the C1 source and aqueous HCl as the proton donor. When commercially available DCl was used, the valuable d^2^-methylation of amines with HCOONa was achieved, yielding products **67–70** with over 80% deuterium incorporation. Furthermore, the combination of DCOONa with DCl enabled full d^3^-methylation of *N*-methyl-4-phenylaniline **71**, tetrahydroquinoline **72**, meclizine **73**, and dibenzylamine **74**. This divergent incorporation of deuterated magic methyl groups represents a powerful tool for isotope labeling of small molecules, offering substantial potential for drug optimization and development.

### Mechanistic investigations

**Fig. 5:**
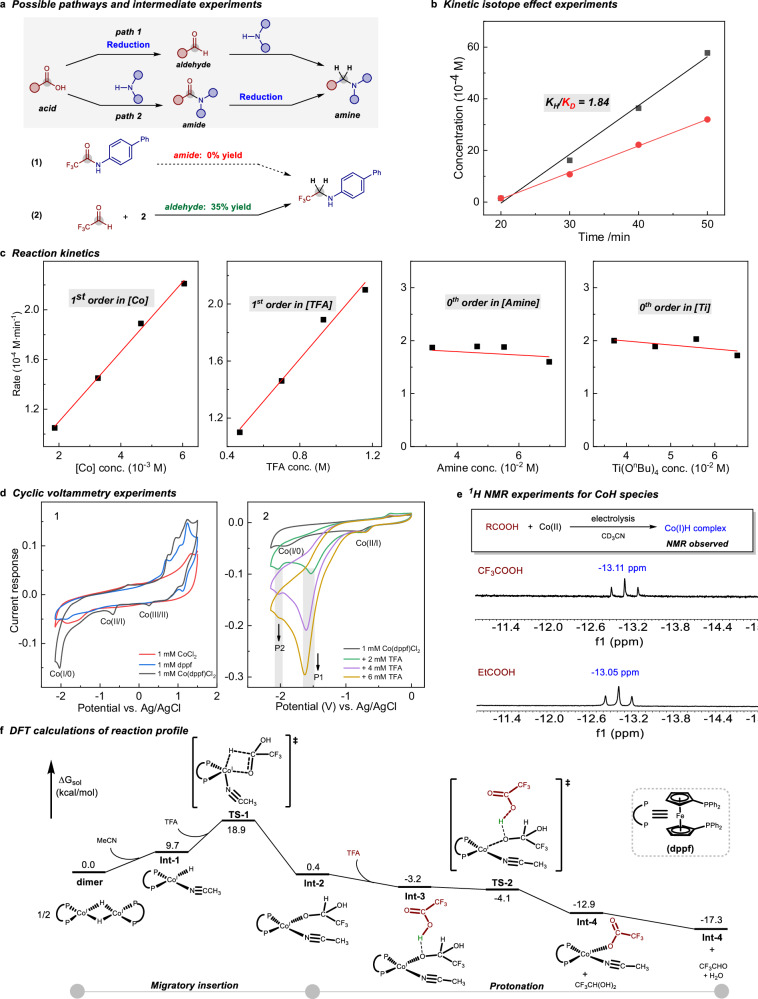
Mechanistic investigations. **a** Possible pathways and intermediate experiments. **b** Kinetic isotope effect experiments. **c** Reaction kinetics. **d** Cyclic voltammetry experiments. **e** ^1^H NMR experiments for CoH species. **f** DFT calculations of reaction profile.

To gain some insights into this electroreduction of carboxylic acids, a series mechanistic experiments were conducted. As shown in Fig. 5a, two plausible pathways were proposed for the hydrogenative coupling of carboxylic acids with amines. The carboxylic acid can be reduced to an aldehyde, which subsequently undergoes reductive amination to form the desired alkylamine. In addition, the amide formation followed by deoxygenative reduction might also exist as an alternative pathway. To elucidate the mechanism, we investigated the potential intermediacy of amide or aldehyde in this electroreduction. When the reaction was conducted with amides under electrocatalytic conditions, the expected corresponding amines were not formed. In contrast, treatment of trifluoroacetaldehyde hydrate as the alkylating reagent in place of TFA, the desired *N*-trifluoroethylative product was obtained in 35% yield. These findings suggest that amide formation prior to reduction is not involved in this transformation.

Next, we conducted further kinetic studies to elucidate the rate-determining step of the carboxylic acid electrohydrogenation process. Initially, kinetic isotope effect (KIE) experiments were performed using deuterated and non-deuterated TFA as substrates (Fig. 5b). The rate profiles from the KIE experiments revealed a K_H_/K_D_ value of 1.84, indicating a primary KIE and suggesting that hydrogen transfer is a key step in the reaction mechanism. Subsequently, we investigated the reaction kinetics by varying the concentrations of the cobalt catalyst, TFA, 4-*tert*-butylaniline, and Ti(O^*n*^Bu)_4_ within the first 50 min of the reaction (Fig. 5c). The kinetic analysis demonstrated a first-order dependence on the concentrations of both the cobalt catalyst and TFA, while the reaction exhibited zero-order dependence on the concentrations of 4-*tert*-butylaniline and Ti(O^*n*^Bu)_4_. Although Ti(O^*n*^Bu)_4_ is not involved in the rate-determining step, its role as a Lewis acid in activating the carboxylic acid carbonyl group was confirmed by ^13^C NMR spectroscopy (Supplementary Fig. 30). These findings suggest that the reduction of the carboxylic acid by the cobalt catalyst is involved in the turnover-limiting step.

Moreover, cyclic voltammetry (CV) experiments were investigated to acquire further understanding for this transformation. As shown in Fig. 5d, measurement of CV curve for the ligand-free cobalt catalyst showed weak redox peaks in a wide range scan from +1.5 to −2.2 V vs. Ag/AgCl (red line). Notably, the application of a dppf-ligated cobalt complex displayed three distinct cathodic peaks at +0.3, −0.7, and −2.0 V vs. Ag/AgCl, corresponding to the Co(III/II), Co(II/I), and Co(I/0) redox processes, respectively (black line). To further probe the interaction between the cobalt complex and TFA, CV measurements were performed with the addition of 2–6 mM TFA to the cobalt complex solution. This resulted in the disappearance of the Co(I/0) peak and the emergence of a new potential (P1) at −1.5 V vs. Ag/AgCl, accompanied by an enhanced current response. This observation suggests the coordination of trifluoroacetate to the cobalt complex. Additionally, a more negative peak (P2) at −2.1 V vs. Ag/AgCl was detected, indicating the possible formation of a more reductive cobalt species (Co-H) following acid addition^70^. To further characterize this key intermediate, NMR experiments were performed. The reactions were carried out by direct electrolysis of carboxylic acids (TFA or propionic acid) with the cobalt complex in CD_3_CN for 1.5 h. As shown in Fig. 5e, the ^1^H NMR spectra exhibited similar chemical shifts at approximately −13.1 ppm, which can be attributed to the hydride signal of a Co(I)-H species^71,72^. Furthermore, the corresponding aldehyde and alcohol products resulting from acid reduction were also detected (Supplementary Fig. 29). These findings provide evidence for the generation of an active cobalt(I) hydride intermediate under electrocatalytic conditions, which plays a pivotal role in the hydrogenation of carboxylic acids.

Furthermore, we employed density functional theory (DFT) calculations to investigate mechanistic details of the key step of hydride transfer to carboxylic acid (Fig. 5f and Supplementary Data 1). Based on the experimental results, the active species is identified as a cobalt(I) hydride complex. Previous studies have shown that cobalt(I) hydride complexes typically form dimeric structures^73,74^, which are considered the resting state and thus serve as the starting point for the energy profile in our DFT calculations. Initially, the Co(I) dimer transforms into the active Co(I)-H species (**Int-1**) with the assistance of solvents, which is endergonic by 9.7 kcal/mol. Next, the Co(I) hydride undergoes migratory insertion into TFA, proceeding through the transition state **TS-1** to form a hemiacetal complex, **Int-2**. This hydride transfer step exhibits an overall activation barrier of 18.9 kcal/mol, representing the rate-determining step of the reaction^75^. Following this, the protonation process occurs. The proton of TFA initially approaches **Int-2**, forming a hydrogen bond with the hemiacetal oxygen atom, resulting in **Int-3**, with a decrease in energy by 3.6 kcal/mol. Subsequently, the proton transfer proceeds readily via the transition state **TS-2**, leading to the formation of 2,2,2-trifluoroethane-1,1-diol and a carbonate complex **Int-4**. This protonation process is energetically favorable, with a decrease of 13.3 kcal/mol from **Int-2** to **Int-4**. The transition state **TS-2**, while identifiable in terms of electronic energy in the gas phase, disappears after corrections for solvation effects and thermal contributions, indicating that the proton transfer can proceed smoothly. The resulting 2,2,2-trifluoroethane-1,1-diol is unstable and readily converts to the more stable trifluoroacetaldehyde and H_2_O, which is energetically downhill by 4.4 kcal/mol. The subsequent reaction processes and competitive reaction pathways have also been calculated and are presented in the Supplementary Figs. 31–36.

**Fig. 6:**
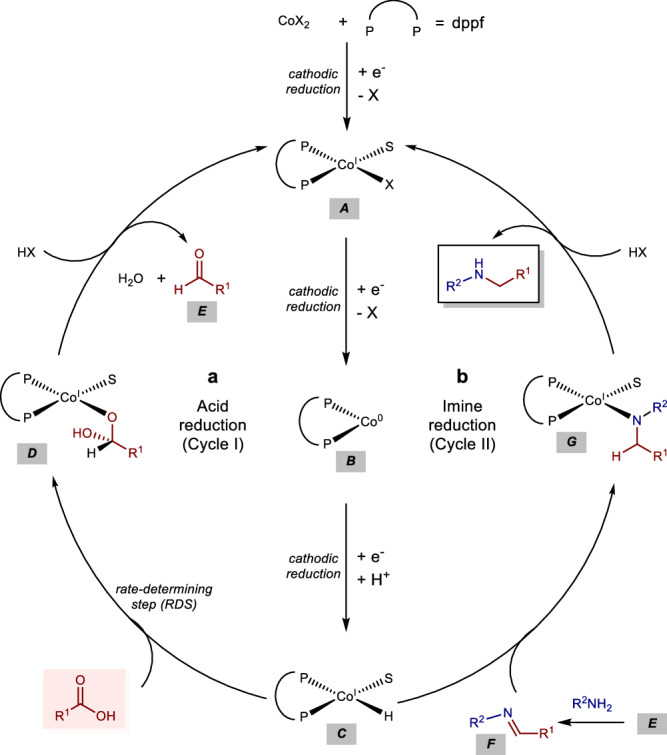
Proposed mechanism. **a** Acid reduction (Cycle I) **b** Imine reduction (Cycle II).

Based on these results, we propose a plausible mechanism in Fig. 6. Firstly, the monoligated cobalt complex is reduced to Co(I) **A** and then to Co(0) **B** species through a stepwise electron reduction process on cathode. In the presence of an acid, this complex **B** is further converted to the corresponding Co(I) hydride complex **C**, which is the key active species to initiate the following catalytic cycles. In cycle I, which involves the reduction of the carboxylic acid, the electrogenerated Co(I)−H species **C** transfers a hydride to the carboxylic group, forming a hemiacetal intermediate **D**. This hydride transfer step has been identified as the rate-determining step of the reaction. Following this, intermediate **D** undergoes rapid protonation by an acid, releasing the aldehyde **E** and regenerating the Co(I) species **A**, thereby completing cycle I. In cycle II, the Co(I)−H species **C** reacts with the imine **F**, which is generated through the rapid condensation of the aldehyde **E** with the added amine, to form the cobalt complex **G**. Finally, protonation of complex **G** yields the desired amine product and regenerates the Co(I) species **A**, thus completing cycle II.

## Discussion

In summary, we have developed a selective and efficient cobalt-electrocatalytic reductive amination method using stable and versatile carboxylic acids as substrates. Mechanistic studies reveal that an electrochemically generated Co(I)−H species serves as the key intermediate for carboxylic acid reduction, with hydride transfer to the carboxylic acid identified as the rate-determining step. By utilizing readily available protons as the hydrogen source, this electrochemical approach offers a mild, safe, and easily manipulated method for synthesizing complex alkylamines with a broad range of functionally and structurally diverse substituents. Furthermore, this protocol is applicable for the practical and cost-effective incorporation of deuterium isotopes into various amines, including pharmaceutical molecules. We anticipate that this transformation will enable robust and sustainable access to value-added alkylamines, providing a viable alternative to current benchmark methods.

## Methods

### Procedure for the synthesis of compound 3

A 10 mL glass tube equipped with a magnetic stir bar was charged with 4-phenylaniline **2** (0.2 mmol), Co(OTf)_2_ (0.02 mmol), and dppf (0.02 mmol). Toluene (2.0 mL), acetonitrile (2.0 mL), Ti(O^*n*^Bu)_4_ (0.2 mmol), and TFA **1** (4.0 mmol) were then added sequentially. The reactor was equipped with Zn plates as both the cathode and anode (size 2.5 cm × 1.0 cm × 0.05 cm). The reaction was purged with N_2_ for three minutes, and the tube was wrapped with tape. The reaction was then electrolyzed under a constant current of 20 mA for 3 h at 70 °C. Upon completion of the reaction, the mixture was quenched with saturated NaHCO_3_ solution and diluted with ethyl acetate. The organic layer was washed with saturated NaHCO_3_, dried over anhydrous Na_2_SO_4_, and concentrated under reduced pressure. The resulting residue was purified by silica gel flash chromatography to afford the product **3** in 91% isolated yield.

## Supplementary information

Supplementary Information

Description of Additional Supplementary Files

Transparent Peer Review file

Supplementary Data 1

## Data Availability

The data reported in this paper are available within the article and its Supplementary Information files. All data is also available from the corresponding author upon request.

## References

[1] Trowbridge, A., Walton, S. M. & Gaunt, M. J. New strategies for the transition-metal catalyzed synthesis of aliphatic amines. *Chem. Rev.* **120**, 2613–2692 (2020). 32064858 10.1021/acs.chemrev.9b00462

[2] Ricci, A. & Bernardi, L. *Methodologies in Amine Synthesis: Challenges and Applications* (Wiley-VCH, 2021).

[3] Murugesan, K. et al. Catalytic reductive aminations using molecular hydrogen for synthesis of different kinds of amines. *Chem. Soc. Rev.* **49**, 6273–6328 (2020). 32729851 10.1039/c9cs00286c

[4] Irrgang, T. & Kempe, R. Transition-metal-catalyzed reductive amination employing hydrogen. *Chem. Rev.* **120**, 9583–9674 (2020). 32812752 10.1021/acs.chemrev.0c00248

[5] Li, J.-B., Huang, C.-Y. & Li, C.-J. Deoxygenative functionalizations of aldehydes, ketones and carboxylic acids. *Angew. Chem. Int. Ed.* **61**, e202112770 (2022). 10.1002/anie.202112770 34780098

[6] Dong, J. & Yang, Z. Catalytic one-pot reductive amination of carboxylic acids. *ChemCatChem* **16**, e202301427 (2024).

[7] Cabrero-Antonino, J. R., Adam, R. & Beller, M. Catalytic reductive N-alkylations using CO₂ and carboxylic acid derivatives: recent progress and developments. *Angew. Chem. Int. Ed.* **58**, 12820–12838 (2019). 10.1002/anie.201810121 30306704

[8] Gribble, G. W. et al. Reactions of sodium borohydride in acidic media. I. Reduction of indoles and alkylation of aromatic amines with carboxylic acids. *J. Am. Chem. Soc.* **96**, 7812–7814 (1974).

[9] Marchini, P. et al. Sodium borohydride-carboxylic acid systems. Useful reagents for the alkylation of amines. *J. Org. Chem.* **40**, 3453–3456 (1975).

[10] Sorribes, I., Junge, K. & Beller, M. Direct catalytic N-alkylation of amines with carboxylic acids. *J. Am. Chem. Soc.* **136**, 14314–14319 (2014). 25230096 10.1021/ja5093612

[11] Andrews, K. G., Summers, D. M., Donnelly, L. J. & Denton, R. M. Catalytic reductive N-alkylation of amines using carboxylic acids. *Chem. Commun.* **52**, 1855–1858 (2016). 10.1039/c5cc08881j 26669845

[12] Nguyen, T. V. Q., Yoo, W. J. & Kobayashi, S. Chelating bis(1,2,3-triazol-5-ylidene) rhodium complexes: versatile catalysts for hydrosilylation reactions. *Adv. Synth. Catal.* **358**, 452–458 (2016).

[13] Ouyang, L., Miao, R., Yang, Z. & Luo, R. Iridium-catalyzed reductive amination of carboxylic acids. *J. Catal.* **418**, 283–289 (2023).

[14] Wu, J., Tongdee, S., Ammaiyappan, Y. & Darcel, C. A concise route to cyclic amines from nitroarenes and ketoacids under iron-catalyzed hydrosilylation conditions. *Adv. Synth. Catal.* **363**, 3859–3865 (2021).

[15] Stoll, E. L. et al. A practical catalytic reductive amination of carboxylic acids. *Chem. Sci.* **11**, 9494–9500 (2020). 34123174 10.1039/d0sc02271c PMC8161228

[16] Trillo, P. & Adolfsson, H. Direct catalytic reductive N-alkylation of amines with carboxylic acids: chemoselective enamine formation and further functionalizations. *ACS Catal.* **9**, 7588–7595 (2019).

[17] Qiao, C., Liu, X.-F., Liu, X. & He, L.-N. Copper(II)-catalyzed selective reductive methylation of amines with formic acid: an option for indirect utilization of CO₂. *Org. Lett.* **19**, 1490–1493 (2017). 28263072 10.1021/acs.orglett.7b00551

[18] Fu, M.-C., Shang, R., Cheng, W.-M. & Fu, Y. Boron-catalyzed N-alkylation of amines using carboxylic acids. *Angew. Chem. Int. Ed.* **54**, 9042–9046 (2015). 10.1002/anie.201503879 26150397

[19] Ogiwara, Y., Uchiyama, T. & Sakai, N. Reductive amination/cyclization of keto acids using a hydrosilane for selective production of lactams versus cyclic amines by switching of the indium catalyst. *Angew. Chem. Int. Ed.* **55**, 1864–1867 (2016). 10.1002/anie.201509465 26689435

[20] Wei, Y., Xuan, Q., Zhou, Y. & Song, Q. Reductive N-alkylation of primary and secondary amines using carboxylic acids and borazane under mild conditions. *Org. Chem. Front.* **5**, 3510–3514 (2018).

[21] Shi, Y., Kamer, P. C. J. & Cole-Hamilton, D. J. A new route to α,ω-diamines from hydrogenation of dicarboxylic acids and their derivatives in the presence of amines. *Green. Chem.* **19**, 5460–5466 (2017).

[22] Liu, W. et al. Tailored cobalt-catalysts for reductive alkylation of anilines with carboxylic acids under mild conditions. *Angew. Chem. Int. Ed.* **57**, 11673–11677 (2018). 10.1002/anie.201806132 30019810

[23] Sorribes, I. et al. Catalytic N-alkylation of amines using carboxylic acids and molecular hydrogen. *J. Am. Chem. Soc.* **137**, 13580–13587 (2015). 26484397 10.1021/jacs.5b07994

[24] Emayavaramban, B., Chakraborty, P. & Sundararaju, B. Cobalt-catalyzed reductive alkylation of amines with carboxylic acids. *ChemSusChem* **12**, 3089–3093 (2019). 30418691 10.1002/cssc.201802144

[25] Yan, M., Kawamata, Y. & Baran, P. S. Synthetic organic electrochemical methods since 2000: on the verge of a renaissance. *Chem. Rev.* **117**, 13230–13319 (2017). 28991454 10.1021/acs.chemrev.7b00397 PMC5786875

[26] Novaes, L. F. T. et al. Electrocatalysis as an enabling technology for organic synthesis. *Chem. Soc. Rev.* **50**, 7941–8002 (2021). 34060564 10.1039/d1cs00223f PMC8294342

[27] Zhu, C., Ang, N. W. J., Meyer, T. H., Qiu, Y. & Ackermann, L. Organic electrochemistry: molecular syntheses with potential. *ACS Cent. Sci.* **7**, 415–431 (2021). 33791425 10.1021/acscentsci.0c01532 PMC8006177

[28] Malapit, C. A. et al. Advances on the merger of electrochemistry and transition metal catalysis for organic synthesis. *Chem. Rev.* **122**, 3180–3218 (2022). 34797053 10.1021/acs.chemrev.1c00614 PMC9714963

[29] Kaefer, N. & Leitner, W. Electrocatalysis with molecular transition-metal complexes for reductive organic synthesis. *JACS Au* **2**, 1266–1289 (2022). 35783173 10.1021/jacsau.2c00031 PMC9241009

[30] Cheng, X. et al. Recent applications of homogeneous catalysis in electrochemical organic synthesis. *CCS Chem.* **4**, 1120–1152 (2022).

[31] Li, Y., Wen, L. & Guo, W. A guide to organic electroreduction using sacrificial anodes. *Chem. Soc. Rev.* **52**, 1168–1188 (2023). 36727623 10.1039/d3cs00009e

[32] Liu, Y., Li, P., Wang, Y. & Qiu, Y. Electroreductive cross-electrophile coupling (eXEC) reactions. *Angew. Chem. Int. Ed.* **62**, e202306679 (2023). 10.1002/anie.202306679 37327185

[33] Zeng, L., Wang, J., Wang, D., Yi, H. & Lei, A. Comprehensive comparisons between directing and alternating current electrolysis in organic synthesis. *Angew. Chem. Int. Ed.* **62**, e202309620 (2023). 10.1002/anie.202309620 37606535

[34] Yang, J. et al. Advances in electrochemical hydrogenation since 2010. *Adv. Synth. Catal.* **363**, 5407–5416 (2021).

[35] Shi, Z. et al. Recent advances in the electrochemical hydrogenation of unsaturated hydrocarbons. *Curr. Opin. Electrochem.* **28**, 100713 (2021).

[36] Durin, G., Kaeffer, N. & Leitner, W. Electrocatalytic hydrogenation of unsaturated organic compounds with molecular complexes: mechanistic views. *Curr. Opin. Electrochem.* **41**, 101371 (2023).

[37] Hua, Y., Bi, H. & Liu, J. Electrohydrogenation of unsaturated bonds catalyzed by earth-abundant metal complexes. *ChemElectroChem* **11**, e202400462 (2024).

[38] Chen, Z., Glasson, C. R. K., Holland, P. L. & Meyer, T. J. Electrogenerated polypyridyl ruthenium hydride and ligand activation for water reduction to hydrogen and acetone to iso-propanol. *Phys. Chem. Chem. Phys.* **15**, 9503–9507 (2013). 23681418 10.1039/c3cp51946e

[39] Armstrong, K. C. & Waymouth, R. M. Electroreduction of benzaldehyde with a metal–ligand bifunctional hydroxycyclopentadienyl molybdenum(II) hydride. *Organometallics* **39**, 4415–4419 (2020).

[40] Fokin, I. & Siewert, I. Chemoselective electrochemical hydrogenation of ketones and aldehydes with a well-defined base-metal catalyst. *Chem. Eur. J.* **26**, 14137–14143 (2020). 32497312 10.1002/chem.202002075 PMC7702145

[41] Fokin, I., Kuessner, K.-T. & Siewert, I. Electroreduction of carbonyl compounds catalyzed by a manganese complex. *ACS Catal.* **12**, 8632–8640 (2022).

[42] Wang, T., He, F., Jiang, W. & Liu, J. Electrohydrogenation of nitriles with amines by cobalt catalysis. *Angew. Chem. Int. Ed.* **63**, e202316140 (2024). 10.1002/anie.202316140 38124405

[43] Qu, R., Junge, K. & Beller, M. Hydrogenation of carboxylic acids, esters, and related compounds over heterogeneous catalysts: a step toward sustainable and carbon-neutral processes. *Chem. Rev.* **123**, 1103–1165 (2023). 36602203 10.1021/acs.chemrev.2c00550

[44] Korstanje, T. J., van der Vlugt, J. I., Elsevier, C. J. & de Bruin, B. Hydrogenation of carboxylic acids with a homogeneous cobalt catalyst. *Science* **350**, 298–302 (2015). 26472903 10.1126/science.aaa8938

[45] Li, L., Yao, Y. & Fu, N. Free carboxylic acids: the trend of radical decarboxylative functionalization. *Eur. J. Org. Chem.* **26**, e202300166 (2023).

[46] Ramadoss, V. et al. Advances in electrochemical decarboxylative transformation reactions. *Chem. Eur. J.* **27**, 3213–3228 (2021). 32633436 10.1002/chem.202001764

[47] Chen, N., Ye, Z.-H. & Zhang, F.-Z. Recent progress on electrochemical synthesis involving carboxylic acids. *Org. Biomol. Chem.* **19**, 5501–5520 (2021). 34079974 10.1039/d1ob00420d

[48] Yoshioka, S. & Saito, S. Catalytic hydrogenation of carboxylic acids using low-valent and high-valent metal complexes. *Chem. Commun.* **54**, 13319–13330 (2018). 10.1039/c8cc06543h 30311923

[49] Pritchard, J. et al. Heterogeneous and homogeneous catalysis for the hydrogenation of carboxylic acid derivatives: history, advances and future directions. *Chem. Soc. Rev.* **44**, 3808–3833 (2015). 25941799 10.1039/c5cs00038f

[50] Dub, P. A. & Ikariya, T. Catalytic reductive transformations of carboxylic and carbonic acid derivatives using molecular hydrogen. *ACS Catal.* **2**, 1718–1741 (2012).

[51] Zhou, M.-X. et al. Recent advances in trifluoroethylation reaction. *Org. Chem. Front.* **10**, 5986–6009 (2023).

[52] Arp, H. P. H. et al. The global threat from the irreversible accumulation of trifluoroacetic acid (TFA). *Environ. Sci. Technol.* **58**, 19925–19935 (2024). 39475534 10.1021/acs.est.4c06189 PMC11562725

[53] Ai, W., Zhong, R., Liu, X. & Liu, Q. Hydride transfer reactions catalyzed by cobalt complexes. *Chem. Rev.* **119**, 2876–2953 (2019). 30565455 10.1021/acs.chemrev.8b00404

[54] Jana, S., Mayerhofer, V. J. & Teskey, C. J. Photo- and electrochemical cobalt catalysed hydrogen atom transfer for the hydrofunctionalisation of alkenes. *Angew. Chem. Int. Ed.* **62**, e202304882 (2023). 10.1002/anie.202304882 37184388

[55] Li, Y. et al. Recent advances in cobalt-catalyzed regio- or stereoselective hydrofunctionalization of alkenes and alkynes. *CCS Chem.* **6**, 1130–1156 (2024).

[56] Andrews, K. G., Faizova, R. & Denton, R. M. A practical and catalyst-free trifluoroethylation reaction of amines using trifluoroacetic acid. *Nat. Commun.* **8**, 15913 (2017). 28649981 10.1038/ncomms15913 PMC5490195

[57] Wang, B., Huang, X., Bi, H. & Liu, J. Electroreductive alkylations of (hetero)arenes with carboxylic acids. *Nat. Commun.* **15**, 4970 (2024). 38862567 10.1038/s41467-024-49355-1 PMC11166922

[58] Chen, Y. Recent advances in methylation: a guide for selecting methylation reagents. *Chem. Eur. J.* **25**, 3405–3439 (2019). 30328642 10.1002/chem.201803642

[59] Steverlynck, J., Sitdikov, R. & Rueping, M. The deuterated “magic methyl” group: a guide to site-selective trideuteromethyl incorporation and labeling by using CD₃ reagents. *Chem. Eur. J.* **27**, 11751–11772 (2021). 34076925 10.1002/chem.202101179 PMC8457246

[60] Aynetdinova, D. et al. Installing the “magic methyl”-C-H methylation in synthesis. *Chem. Soc. Rev.* **50**, 5517–5563 (2021). 33690769 10.1039/d0cs00973c

[61] Huang, J., Chen, Z. & Wu, J. Recent progress in methyl-radical-mediated methylation or demethylation reactions. *ACS Catal.* **11**, 10713–10732 (2021).

[62] Goyal, V. et al. Recent advances in the catalytic N-methylation and N-trideuteromethylation reactions using methanol and deuterated methanol. *Coord. Chem. Rev.* **474**, 214827 (2023).

[63] Sorribes, I., Junge, K. & Beller, M. General catalytic methylation of amines with formic acid under mild reaction conditions. *Chem. Eur. J.* **20**, 7878–7883 (2014). 24889122 10.1002/chem.201402124

[64] Savourey, S., Lefèvre, G., Bertheta, J.-C. & Cantat, T. Catalytic methylation of aromatic amines with formic acid as the unique carbon and hydrogen source. *Chem. Commun.* **50**, 14033–14036 (2014). 10.1039/c4cc05908e 25268489

[65] Lu, G.-S. et al. Catalytic reductive amination and tandem amination-alkylation of esters enabled by a cationic iridium complex. *Angew. Chem. Int. Ed.* **63**, e202422742 (2024). 10.1002/anie.202422742 39655429

[66] Li, H., Shabbir, M., Li, W. & Lei, A. Recent advances in deuteration reactions. *Chin. J. Chem.* **42**, 1145–1156 (2024).

[67] Ou, W., Qiu, C. & Su, C. Photo- and electro-catalytic deuteration of feedstock chemicals and pharmaceuticals: a review. *Chin. J. Catal.* **43**, 956–970 (2022).

[68] Kopf, S. et al. Recent developments for the deuterium and tritium labeling of organic molecules. *Chem. Rev.* **122**, 6634–6718 (2022). 35179363 10.1021/acs.chemrev.1c00795

[69] Zhang, Z. et al. Semiconductor photocatalysis to engineering deuterated N-alkyl pharmaceuticals enabled by synergistic activation of water and alkanols. *Nat. Commun.* **11**, 4722 (2020). 32948764 10.1038/s41467-020-18458-w PMC7501254

[70] Ciancanelli, R., Noll, B. C., DuBois, D. L. & DuBois, M. R. Comprehensive thermodynamic characterization of the metal-hydrogen bond in a series of cobalt-hydride complexes. *J. Am. Chem. Soc.* **124**, 2984–2992 (2002). 11902890 10.1021/ja0122804

[71] Liu, B., Li, Y. & Liu, Q. Cobalt/Lewis acid cooperative catalysis for reductive etherification of ketones and aldehydes with alcohols. *Chem. Catal.* **2**, 883–897 (2022).

[72] Kong, D. et al. Cobalt-catalyzed (E)-selective hydrosilylation of 1,3-enynes for the synthesis of 1,3-dienylsilanes. *Organometallics* **40**, 2070–2080 (2021).

[73] Li, Y. et al. Ligand-controlled cobalt-catalyzed regiodivergent alkyne hydroalkylation. *J. Am. Chem. Soc.* **144**, 13961–13972 (2022). 35866845 10.1021/jacs.2c06279

[74] Zhang, Z.-L. et al. Cobalt-catalyzed facial-selective hydroalkylation of cyclopropenes. *Angew. Chem. Int. Ed.* **62**, e202306381 (2023). 10.1002/anie.202306381 37254230

[75] Wang, J., Wu, K. & Qi, X. Theoretical study of the ligand effect on NHC cobalt-catalyzed hydrogenation of ketones. *Catal. Sci. Technol.* **9**, 5315–5321 (2019).

