# Supplementary material for: Electroreductive amination of carboxylic acids by cobalt catalysis
Source: Nat Commun. 2025 Aug 4;16:7167. doi: 10.1038/s41467-025-62396-4 (PMC12322302; doi:10.1038/s41467-025-62396-4)
Supplement: Supplementary file 1 — Supplementary Information [file 41467_2025_62396_MOESM1_ESM.pdf]

Supplementary Information

Electroreductive amination of carboxylic acids by cobalt catalysis

Huihua Bi,[1] Zhizheng Chen,[1] Changsheng Bi,[1] Shuanglin Qu*[1] and Jie Liu*[1,2]

[1] *College of Chemistry and Chemical Engineering, State Key Laboratory of Chemo and Biosensing, Hunan University, 410082, Changsha, China*

[2] *Greater Bay Area Institute for Innovation, Hunan University, 511300, Guangzhou China*

E-mail: squ@hnu.edu.cn; jieliu@hnu.edu.cn

1. Supplementary Notes 1

2. Supplementary Methods 2

 2.1 Optimization of reaction conditions 2

 2.2 General reaction procedures 6

3. Supplementary Discussion 11

 3.1 Substrate limitations 11

 3.2 Experimental mechanistic studies 11

 3.3 DFT calculations 26

 3.4 Characterization of products 31

 3.5 NMR Spectra of products 55

4. Supplementary References 180

1. Supplementary Notes

Unless otherwise noted, all reagents were utilized as received from the commercial suppliers. Electrode materials are commercially available and directly used in this work. Reactions were monitored by thin-layer chromatography (TLC). TLC plates were visualized with UV light (254 nm) or iodine stain. Column chromatography was performed using silica gel (300-400 mesh). ^{1}H, ^{13}C and ^{19}F NMR spectra were recorded on a Bruker Avance III HD NMR 400 MHz instrument , with internal referencing to the residual undeuterated solvent signals ($CDCl_3$: 7.26 ppm for ^{1}H and 77.0 ppm for ^{13}C; DMSO-d^6: 2.50 ppm for ^{1}H and 39.5 ppm for ^{13}C; CD_3OD: 3.31 ppm for ^{1}H and 48.0 ppm for ^{13}C). Data for ^{1}H are reported as: chemical shift (δ ppm), integration, multiplicity (s: singlet, d: doublet, t: triplet, m: multiplet, br: broad peak), coupling constant (Hz) and assignment. High-resolution mass spectra (HRMS) were obtained using a Thermo Scientific Orbitrap Exploris 120. GC-MS (EI) analyses were performed on Agilent 8860A GC systems and 5977 Series MSD.

2. Supplementary Methods

2.1 Optimization of reaction conditions

Supplementary Table 1 Optimization of ligands

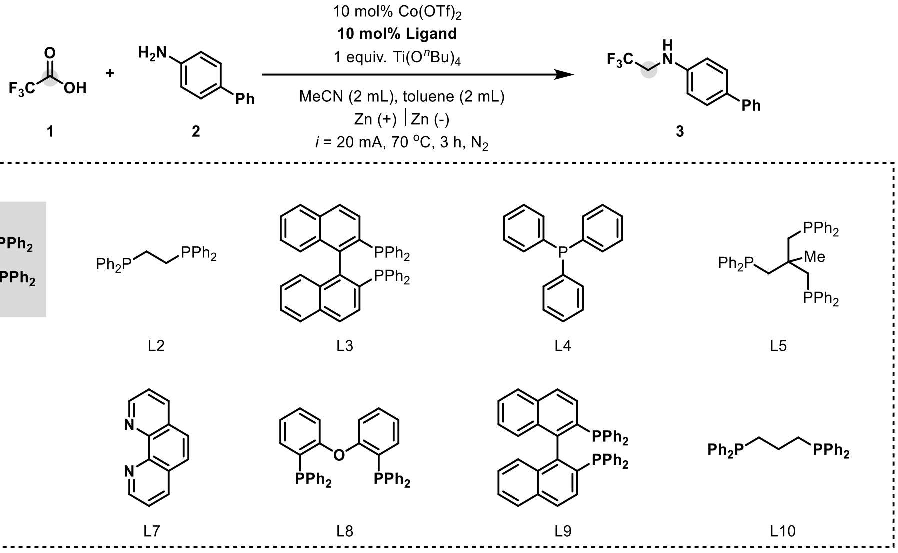

Entry	Ligand	Yield/%
1	10 mol% L1	93
2	10 mol% L2	31
3	10 mol% L3	53
4	10 mol% L4	51
5	10 mol% L5	28
6	10 mol% L6	49
7	10 mol% L7	48
8	10 mol% L8	41
9	10 mol% L9	49
10	10 mol% L10	40

Reaction condition: trifluoroacetic acid **1** (4.0 mmol), 4-phenylaniline **2** (0.2 mmol), $Co(OTf)_2$ (10 mol%), ligand (10 mol%), $Ti(O^nBu)_4$ (0.2 mmol), MeCN (2.0 mL), toluene (2.0 mL) in an undivided cell with zinc cathode and anode (2.5 cm × 1.0 cm × 0.05 cm), constant current 20 mA, 70°C for 3 h. Yields are determined by ^1H NMR.

Supplementary Table 2 Optimization of catalysts

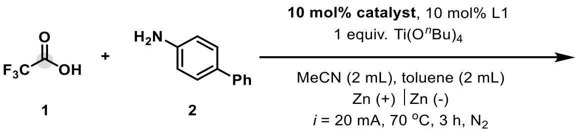

Entry	Catalyst	Yield/%
1	$CoCl_2$	85

2	$CoBr_2$	82
3	$Co(OAc)_2$	90
4	$Co(OTf)_2$	93
5	$Co(acac)_3$	80
6	$FeCl_2$	0
7	$NiCl_2$	0

Reaction condition: trifluoroacetic acid **1** (4.0 mmol), 4-phenylaniline **2** (0.2 mmol), catalyst (10 mol%), **L1** (10 mol%), $Ti(O^nBu)_4$ (0.2 mmol), MeCN (2.0 mL), toluene (2.0 mL) in an undivided cell with zinc cathode and anode (2.5 cm × 1.0 cm × 0.05 cm), constant current 20 mA, 70°C for 3 h. Yields are determined by ^{1}H NMR.

Supplementary Table 3 Optimization of solvents

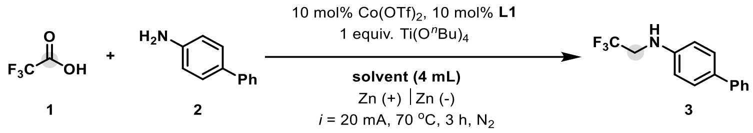

Entry	Solvent	Yield/%
1	MeCN:THF = 1:1	62
2	MeCN:dioxane = 1:1	59
3	MeCN:MeOH = 1:1	0
4	MeCN:*n*-BuOH = 1:1	0
5	MeCN:DMAc = 1:1	0
6	MeCN:DMSO = 1:1	7
7	MeCN:Toluene = 1:1	93
8	THF	0
9	MeCN	71

Reaction condition: trifluoroacetic acid **1** (4.0 mmol), 4-phenylaniline **2** (0.2 mmol), $Co(OTf)_2$ (10 mol%), L1 (10 mol%), $Ti(O^nBu)_4$ (0.2 mmol), solvent (4.0 mL) in an undivided cell with zinc cathode and anode (2.5 cm × 1.0 cm × 0.05 cm), constant current 20 mA, 70°C for 3 h. Yields are determined by ^{1}H NMR.

Supplementary Table 4 Optimization of temperature

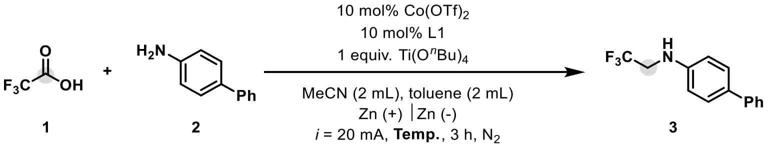

Entry	Temperature	Yield/%
1	60°C	81
2	70°C	93
3	80°C	84
4	90°C	75

Reaction condition: trifluoroacetic acid **1** (4.0 mmol), 4-phenylaniline **2** (0.2 mmol), $Co(OTf)_2$ (10 mol%), L1 (10 mol%), $Ti(O^nBu)_4$ (0.2 mmol), MeCN (2.0 mL), toluene (2.0 mL) in an undivided cell

with zinc cathode and anode (2.5 cm × 1.0 cm × 0.05 cm), constant current 20 mA, X°C for 3 h. Yields are determined by ^{1}H NMR.

Supplementary Table 5 Optimization of electrodes

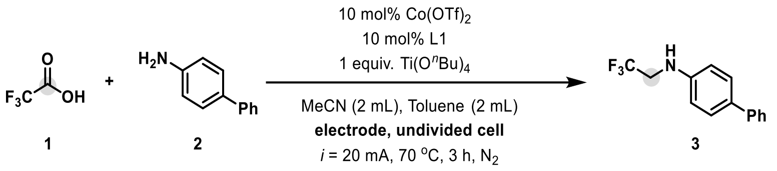

Entry	Electrode	Yield/%
1	Zn (+)|C plate (-)	85
2	Mg (+)|C plate (-)	0
3	Al (+)|C plate (-)	0
4	Fe (+)|C plate (-)	0
5	Zn (+)|Pt (-)	77
6	Zn (+)|Zn (-)	93

Reaction condition: trifluoroacetic acid **1** (4.0 mmol), 4-phenylaniline **2** (0.2 mmol), $Co(OTf)_2$ (10 mol%), L1 (10 mol%), $Ti(O^nBu)_4$ (0.2 mmol), MeCN (2.0 mL), toluene (2.0 mL) in an undivided cell with cathode and anode, constant current 20 mA, 70°C for 3 h. Yields are determined by ^{1}H NMR.

Supplementary Table 6 Optimization of the amount of TFA

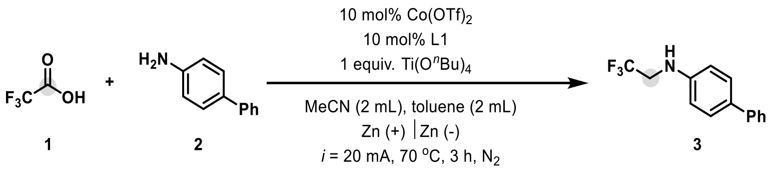

Entry	TFA	Yield/%
1	10 eq. TFA	63
2	15 eq. TFA	87
3	20 eq. TFA	93
4	25 eq. TFA	78

Reaction condition: trifluoroacetic acid **1** (X mmol), 4-phenylaniline **2** (0.2 mmol), $Co(OTf)_2$ (10 mol%), ligand (10 mol%), $Ti(O^nBu)_4$ (0.2 mmol), MeCN (2.0 mL), toluene (2.0 mL) in an undivided cell with zinc cathode and anode (2.5 cm × 1.0 cm × 0.05 cm), constant current 20 mA, 70°C for 3 h. Yields are determined by ^{1}H NMR.

Supplementary Table 7 Optimization of current

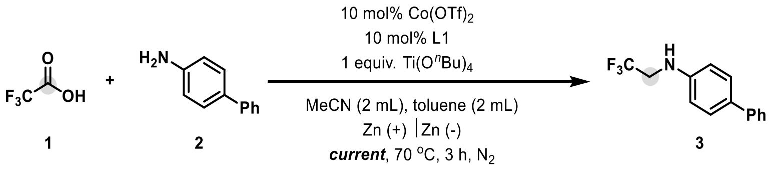

Entry	Current	Time	Yield/%
1	10 mA	6 h	85

2	20 mA	3 h	93
3	25 mA	2.5 h	81

Reaction condition: trifluoroacetic acid **1** (4 mmol), 4-phenylaniline **2** (0.2 mmol), $Co(OTf)_2$ (10 mol%), ligand (10 mol%), $Ti(O^nBu)_4$ (0.2 mmol), MeCN (2.0 mL), toluene (2.0 mL) in an undivided cell with zinc cathode and anode (2.5 cm × 1.0 cm × 0.05 cm), constant current X mA, 70°C for Y h. Yields are determined by ^1H NMR.

Supplementary Table 8 Effect of other reductants

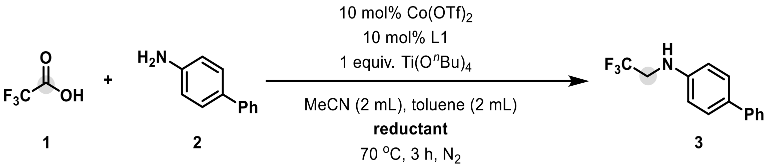

Entry	Reductant	Yield/%
1	10 eq. Mn dust	0
2	10 eq. Mg dust	0
3	10 eq. Zn dust	14
4	30 eq. Zn dust	10
5	2 equiv. $PhSiH_3$	0
5	H_2 ballon	0

Reaction condition: trifluoroacetic acid **1** (4 mmol), 4-phenylaniline **2** (0.2 mmol), $Co(OTf)_2$ (10 mol%), ligand (10 mol%), $Ti(O^nBu)_4$ (0.2 mmol), reductant (X equiv. to **2**), MeCN (2.0 mL), toluene (2.0 mL), 70°C for 3 h. Yields are determined by ^1H NMR.

2.2 General reaction procedures

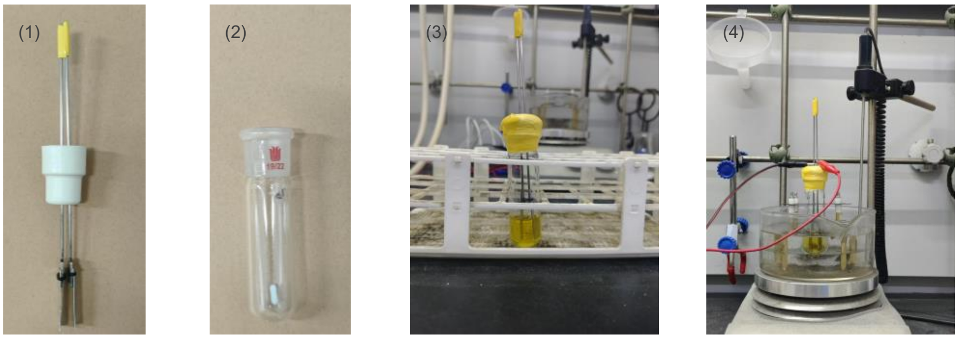

Supplementary Fig. 1 Reaction set-up.

(1) An electrode holder with zinc anode and cathode (both 2.5×1.0×0.05 cm) .

(2) A dried 10 mL glass tube.

(3) The electrodes were inserted into the tube.

(4) Conducted constant current electrolysis using a potentiostat.

2.2.1 Procedure for electrohydrogenation of carboxylic acids with amines

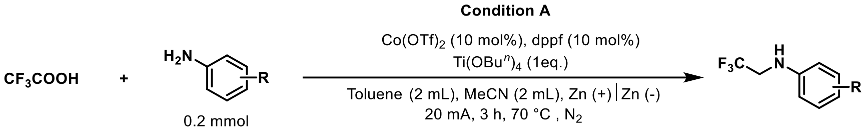

Supplementary Fig. 2 Reaction for electrohydrogenation of TFA with amines.

Condition A (trifluoroethylations): To a 10-mL glass tube equipped with a magnetic stir bar was charged with aniline (0.2 mmol, 1.0 equiv), $Co(OTf)_2$ (0.02 mmol, 10 mol%) and dppf (0.02 mmol, 10 mol%). To the resulting mixture was added sequentially toluene (2.0 mL), acetonitrile (2.0 mL), $Ti(OBu^n)_4$ (0.2 mmol, 1.0 eq.) and trifluoroacetic acid (4.0 mmol, 20 equiv) . The tube was equipped with anode (Zn, 2.5×1.0×0.05 cm) and cathode (Zn, 2.5×1.0×0.05 cm). The reaction mixture was purged with N_2 for three minutes, and tube was wrapped with tape. The electrolysis was carried out using a constant current of 20 mA for 3 h at 70°C. The reaction was quenched with saturated sodium bicarbonate solution and diluted with ethyl acetate. The organic layer was sequentially washed with saturated sodium bicarbonate solution, brine, and dried over anhydrous Na_2SO_4, filtered and concentrated under reduced pressure. The crude residue was subjected to column chromatography on silica gel to give the target product.

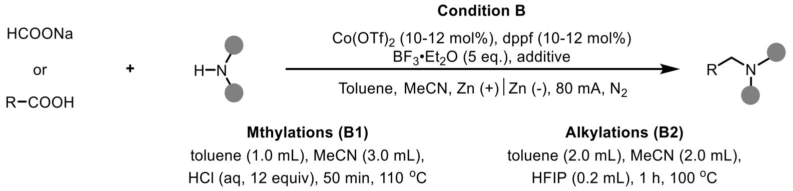

Supplementary Fig. 3 Reaction for electrohydrogenation of alkyl carboxylic acids with amines.

Condition B1 (methylations): To a 10-mL glass tube equipped with a magnetic stir bar was charged with amine (0.15 mmol, 1.0 equiv), HCOONa (1.8 mmol, 12 equiv), $Co(OTf)_2$ (0.018 mmol, 12 mol%) and dppf (0.018 mmol, 12 mol%). To the resulting mixture was added sequentially toluene (1.0 mL), acetonitrile (3.0 mL), HCl Conc. (37 wt. % in H_2O, 1.8 mmol, 12 equiv) and $BF_3 \cdot Et_2O$ (0.75 mmol, 5 equiv). The tube was equipped with anode (Zn, 2.5×1.0×0.05 cm) and cathode (Zn,

2.5×1.0×0.05 cm). The reaction mixture was purged with N_2 for three minutes, and tube was wrapped with tape. The electrolysis was carried out using a constant current of 80 mA for 50 min at 110°C. The reaction was quenched with saturated sodium bicarbonate solution and diluted with ethyl acetate. The organic layer was sequentially washed with saturated sodium bicarbonate solution, brine, and dried over anhydrous Na_2SO_4, filtered and concentrated under reduced pressure. The crude residue was subjected to column chromatography on silica gel to give the target product.

Condition B2 (alkylations): To a 10-mL glass tube equipped with a magnetic stir bar was charged with amine (0.2 mmol, 1.0 equiv), $Co(OTf)_2$ (0.02 mmol, 10 mol%) and dppf (0.02 mmol, 10 mol%). To the resulting mixture was added sequentially toluene (2.0 mL), acetonitrile (2.0 mL), HFIP (0.2 mL), carboxylic acid (2.0 mmol, 10 equiv) and $BF_3 \cdot Et_2O$ (1.0 mmol, 5 equiv.). The tube was equipped with anode (Zn, 2.5×1.0×0.05 cm) and cathode (Zn, 2.5×1.0×0.05 cm). The reaction mixture was purged with N_2 for three minutes, and tube was wrapped with tape. The electrolysis was carried out using a constant current of 80 mA for 1 h at 100°C. The reaction was quenched with saturated sodium bicarbonate solution and diluted with ethyl acetate. The organic layer was sequentially washed with saturated sodium bicarbonate solution, brine, and dried over anhydrous Na_2SO_4, filtered and concentrated under reduced pressure. The crude residue was subjected to column chromatography on silica gel to give the target product.

2.2.2 Procedure for electrohydrogenation of carboxylic acids with nitroarenes

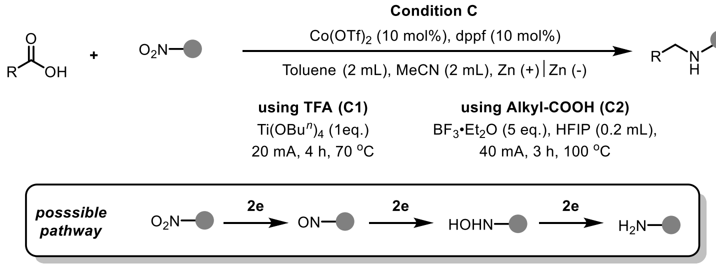

Supplementary Fig. 4 Reaction for electrohydrogenation of carboxylic acids with nitroarenes.

Condition C1 (using TFA): To a 10-mL glass tube equipped with a magnetic stir bar was charged with nitroaromatics (0.2 mmol, 1.0 equiv), $Co(OTf)_2$ (0.02 mmol, 10 mol%) and dppf (0.02 mmol, 10 mol%). To the resulting mixture was added sequentially toluene (2.0 mL), acetonitrile (2.0 mL), $Ti(OBu^n)_4$ (0.2 mmol, 1.0 eq.) and trifluoroacetic acid (4.0 mmol, 20 equiv) . The tube was equipped with anode (Zn, 2.5×1.0×0.05 cm) and cathode (Zn, 2.5×1.0×0.05 cm). The reaction mixture was purged with N_2 for three minutes, and tube was wrapped with tape. The electrolysis was carried out using a constant current of 20 mA for 4 h at 70°C. The reaction was quenched with saturated sodium bicarbonate solution and diluted with ethyl acetate. The organic layer was sequentially washed with saturated sodium bicarbonate solution, brine, and dried over anhydrous Na_2SO_4, filtered and concentrated under reduced pressure. The crude residue was subjected to column chromatography on silica gel to give the target product.

Condition C2 (using Alkyl-COOH): To a 10-mL glass tube equipped with a magnetic stir bar was charged with nitroaromatics (0.2 mmol, 1.0 equiv), $Co(OTf)_2$ (0.02 mmol, 10 mol%) and dppf (0.02 mmol, 10 mol%). To the resulting mixture was added sequentially toluene (2.0 mL), acetonitrile (2.0 mL), HFIP (0.2 mL), carboxylic acid (2.0 mmol, 10 equiv) and $BF_3 \cdot Et_2O$ (1.0 mmol, 5 equiv.). The tube was equipped with anode (Zn, 2.5×1.0×0.05 cm) and cathode (Zn, 2.5×1.0×0.05 cm). The

reaction mixture was purged with N_2 for three minutes, and tube was wrapped with tape. The electrolysis was carried out using a constant current of 40 mA for 3 h at 100°C. The reaction was quenched with saturated sodium bicarbonate solution and diluted with ethyl acetate. The organic layer was sequentially washed with saturated sodium bicarbonate solution, brine, and dried over anhydrous Na_2SO_4, filtered and concentrated under reduced pressure. The crude residue was subjected to column chromatography on silica gel to give the target product.

2.2.3 Procedure for divergent deuterated N-methylation of amines.

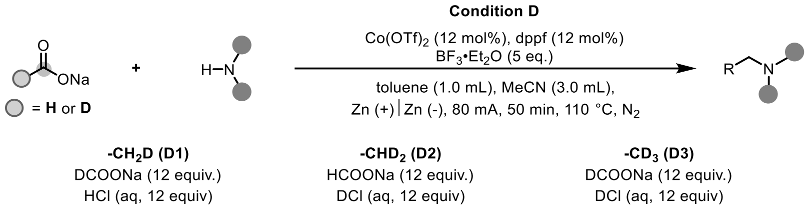

Supplementary Fig. 5 Reaction for divergent deuterated N-methylation of amines.

Condition D1 (CH_2D reaction): To a 10-mL glass tube equipped with a magnetic stir bar was charged with amine (0.15 mmol, 1.0 equiv), **Sodium formate-d** (99.5% D, 1.8 mmol, 12 equiv), $Co(OTf)_2$ (0.018 mmol, 12 mol%) and dppf (0.018 mmol, 12 mol%). To the resulting mixture was added sequentially toluene (1.0 mL), acetonitrile (3.0 mL), HCl Conc. (37 wt. % in H_2O, 1.8 mmol, 12 equiv) and $BF_3{\cdot}Et_2O$ (0.75 mmol, 5 equiv) . The tube was equipped with anode (Zn, 2.5×1.0× 0.05 cm) and cathode (Zn, 2.5×1.0×0.05 cm). The reaction mixture was purged with N_2 for three minutes, and tube was wrapped with tape. The electrolysis was carried out using a constant current of 80 mA for 50 min at 110°C. The reaction was quenched with saturated sodium bicarbonate solution and diluted with ethyl acetate. The organic layer was sequentially washed with saturated sodium bicarbonate solution, brine, and dried over anhydrous Na_2SO_4, filtered and concentrated under reduced pressure. The crude residue was subjected to column chromatography on silica gel to give the target product.

Condition D2 (CHD_2 reaction): To a 10-mL glass tube equipped with a magnetic stir bar was charged with amine (0.15 mmol, 1.0 equiv), Sodium formate (1.8 mmol, 12 equiv), $Co(OTf)_2$ (0.018 mmol, 12 mol%) and dppf (0.018 mmol, 12 mol%). To the resulting mixture was added sequentially toluene (1.0 mL), acetonitrile (3.0 mL), **DCl** Conc. (35 wt. % in D_2O, 1.8 mmol, 12 equiv) and $BF_3{\cdot}Et_2O$ (0.75 mmol, 5 equiv) . The tube was equipped with anode (Zn, 2.5×1.0×0.05 cm) and cathode (Zn, 2.5×1.0×0.05 cm). The reaction mixture was purged with N_2 for three minutes, and tube was wrapped with tape. The electrolysis was carried out using a constant current of 80 mA for 50 min at 110°C. The reaction was quenched with saturated sodium bicarbonate solution and diluted with ethyl acetate. The organic layer was sequentially washed with saturated sodium bicarbonate solution, brine, and dried over anhydrous Na_2SO_4, filtered and concentrated under reduced pressure. The crude residue was subjected to column chromatography on silica gel to give the target product.

Condition D3 (CD_3 reaction): To a 10-mL glass tube equipped with a magnetic stir bar was charged with amine (0.15 mmol, 1.0 equiv), **Sodium formate-d** (99.5% D, 1.8 mmol, 12 equiv), $Co(OTf)_2$ (0.018 mmol, 12 mol%) and dppf (0.018 mmol, 12 mol%). To the resulting mixture was added sequentially toluene (1.0 mL), acetonitrile (3.0 mL), **DCl** Conc. (35 wt. % in D_2O, 1.8 mmol, 12 equiv) and $BF_3{\cdot}Et_2O$ (0.75 mmol, 5 equiv) . The tube was equipped with anode (Zn, 2.5×1.0× 0.05 cm) and cathode (Zn, 2.5×1.0×0.05 cm). The reaction mixture was purged with N_2 for three

minutes, and tube was wrapped with tape. The electrolysis was carried out using a constant current of 80 mA for 50 min at 110°C. The reaction was quenched with saturated sodium bicarbonate solution and diluted with ethyl acetate. The organic layer was sequentially washed with saturated sodium bicarbonate solution, brine, and dried over anhydrous Na_2SO_4, filtered and concentrated under reduced pressure. The crude residue was subjected to column chromatography on silica gel to give the target product.

2.2.4 Procedure for scale-up reaction

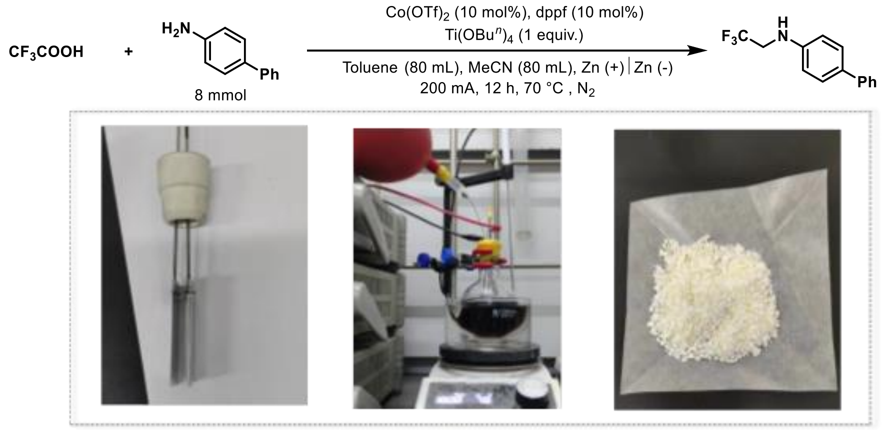

Supplementary Fig. 6 Scale-up reaction.

To a 250 mL round bottom flask equipped with a magnetic stir bar was charged with amine **1** (8 mmol, 1.0 equiv), $Co(OAc)_2$ (0.8 mmol, 10 mol%) and dppf (0.8 mmol, 10 mol%). To the resulting mixture was added sequentially Toluene (80 mL), acetonitrile (80 mL), $Ti(OBu^n)_4$ (8 mmol, 1.0 eq.) and TFA (160 mmol, 20 equiv). The flask was equipped with a Zn plate anode (2.3 cm x 5 cm x 0.15 cm) and a Zn plate (2.3 cm x 5 cm x 0.05 cm) cathode. The reaction mixture was purged with N_2 for 5 minutes, and flask was wrapped with tape. A nitrogen gas balloon was connected to the flask by a needle. The electrolysis was carried out using a constant current of 200 mA for 12 h at 70°C. The reaction was quenched with saturated sodium bicarbonate solution and diluted with ethyl acetate. The organic layer was washed with saturated sodium bicarbonate Sodium, brine, sequentially, and dried over anhydrous Na_2SO_4, filtered and concentrated under reduced pressure. The crude residue was subjected to column chromatography on silica gel eluting with dichloromethane/petroleum ether to give compound **3** as a white solid (1.382g, 68%).

2.2.5 Late-stage functionalizations

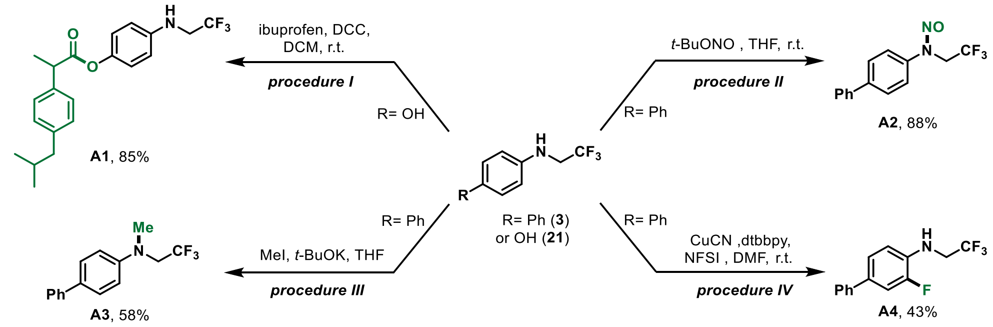

Supplementary Fig. 7 Late-stage functionalizations.

Procedure I: To a 10 mL reaction tube equipped with a stir bar, Compound **21** (0.24 mmol, 1.2 eq.),

ibuprofen (0.2 mmol, 1.0 eq.) and *N*,*N'*-dicyclohexylcarbodiimide (DCC, 0.3 mmol, 1.5 eq.) was sequentially added and dissolved in dichloromethane (DCM, 4 mL). The reaction mixture was stirred at room temperature overnight. After the reaction was completed, the reaction mixture was filtered through a celite pad and subsequently rinsed with 50 mL DCM. The filtrate was collected and concentrated under reduced pressure. The resulting residue was purified by column chromatography using petroleum ether and ethyl acetate (20:1, v/v) as the eluent. The target compound **A1** was obtained as a white solid with an 85% yield.

Procedure II: To a 10 mL reaction tube equipped with a stir bar, Compound **3** (0.2 mmol, 1.0 eq.) was added and dissolved in tetrahydrofuran (1 mL). Tert-butyl nitrite (0.2 mmol, 1.0 eq.) was then introduced, and the reaction mixture was stirred at room temperature for 40 minutes. Upon completion of the reaction, it was quenched with brine, diluted with ethyl acetate, and washed twice with saturated brine. The organic layer was separated, concentrated under reduced pressure. The resulting residue was purified by column chromatography using petroleum ether and ethyl acetate (200:1, v/v) as the eluent. The target compound **A2** was obtained as a pale yellow solid with an 88% yield.[1]

Procedure III: To a 10 mL reaction tube equipped with a stir bar, Compound **3** (0.2 mmol, 1.0 eq.) and t-BuOK (0.4 mmol, 2.0 eq.) was sequentially added and dissolved in tetrahydrofuran (THF, 4 mL). Methyl iodide (0.4 mmol, 2.0 eq.) was then introduced, and the reaction mixture was stirred at 70°C for 6 h. Upon completion of the reaction, it was quenched with brine, diluted with ethyl acetate, and washed twice with saturated brine. The organic layer was separated, concentrated under reduced pressure. The resulting residue was purified by column chromatography using petroleum ether as the eluent. The target compound **A3** was obtained as a white solid with a 58% yield.

Procedure IV: To a 10 mL reaction tube equipped with a stir bar, CuCN (0.02 mmol, 10mol%), 4,4'-di-tert-butyl-2,2'-dipyridyl (dtbbpy, 0.024 mmol, 12 mol%), Compound **3** (0.2 mmol, 1 eq.) and *N*-Fluorobenzenesulfonimide (NFSI, 0.32 mmol, 1.6 eq.) was sequentially added and dissolved in *N*,*N*-Dimethylformamide (DMF, 1 mL). The reaction mixture was stirred at room temperature overnight. Upon completion of the reaction, it was quenched with brine, diluted with ethyl acetate, and washed twice with saturated brine. The organic layer was separated, concentrated under reduced pressure. The resulting residue was purified by column chromatography using petroleum ether and ethyl acetate (20:1, v/v) as the eluent. The target compound **A4** was obtained as a white solid with a 43% yield.[2]

3. Supplementary Discussion

3.1 Substrate limitations

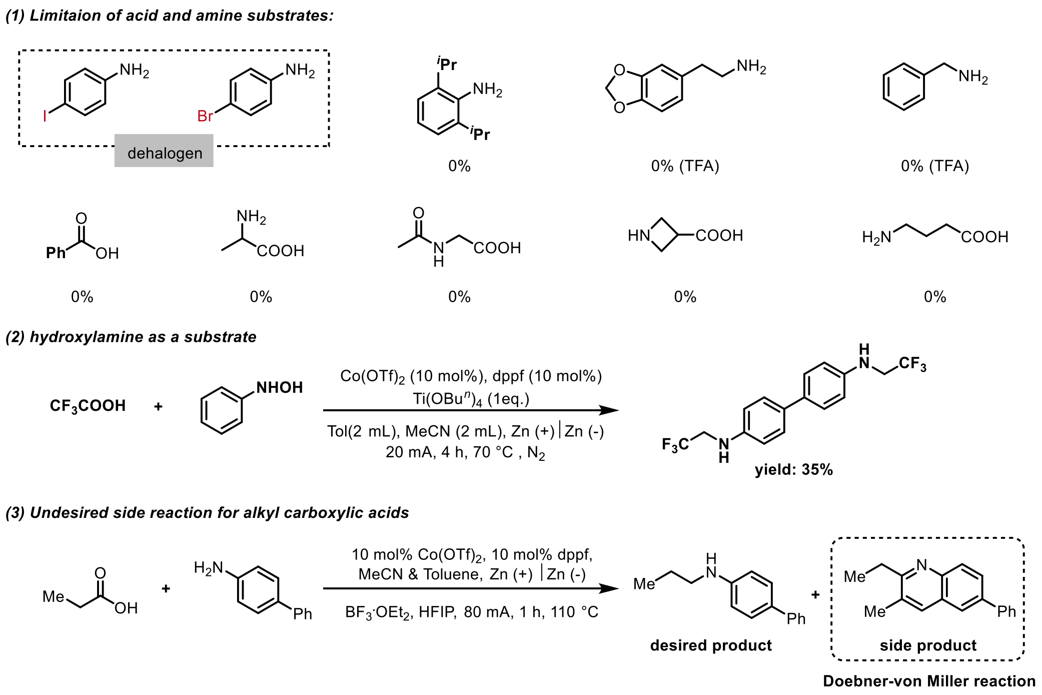

Supplementary Fig. 8 Substrate limitations.

3.2 Experimental mechanistic studies

3.2.1 Intermediate experiments

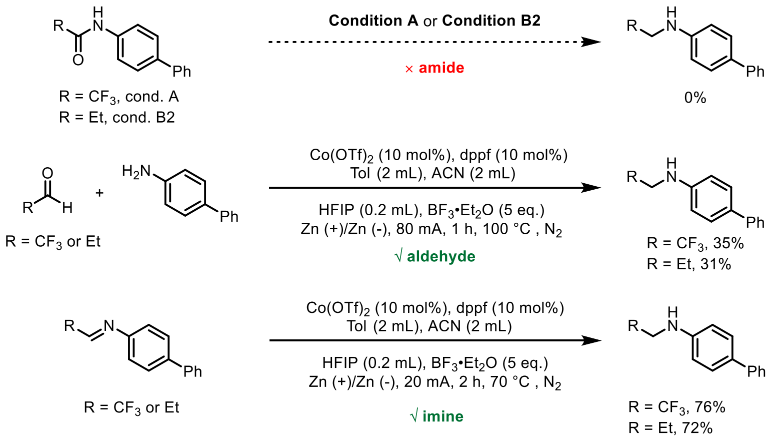

Supplementary Fig. 9 Intermediate experiments.

3.2.2 Kinetic isotope effect experiments

Two parallel reactions using TFA (CF_3COOH) or [d]-TFA (CF_3COOD) were carried out to determine the kinetic isotope effect by comparison of the initial rates. In a 10-mL glass tube equipped with a magnetic stir bar, 4-tert-butylaniline, $Co(OTf)_2$, and dppf were added sequentially. To this mixture, toluene (2.0 mL), acetonitrile (2.0 mL), $Ti(OBu^n)_4$, trifluoroacetic acid (TFA), and

dodecane (46 μL, used as an internal standard) were subsequently introduced. The tube was equipped with anode (Zn, 2.5 × 1.0 × 0.05 cm) and cathode (Zn, 2.5 × 1.0 × 0.05 cm). Prior to electrolysis, the reaction mixture was sparged with nitrogen for three minutes, after which the tube was wrapped with tape. Electrolysis was performed at a constant current of 20 mA and a temperature of 70°C. Aliquots of 0.1 mL from the reaction mixture were withdrawn at intervals of 20, 30, 40 and 50 minutes post-initiation of the reaction. The crude reaction mixture was analyzed by gas chromatography (GC). The initial reaction rate was determined from the slope of the concentration versus time plot. The slope ratio of two curves proves KIE of $k_H/k_D \approx 1.84$.

Supplementary Table 9 Kinetic isotope effect data.

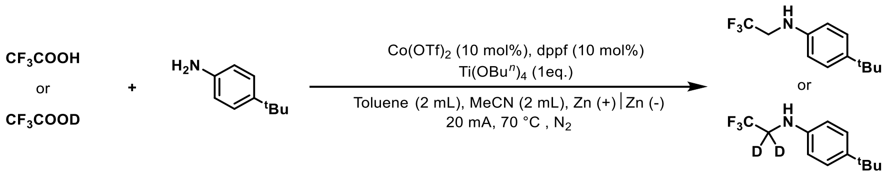

Time/min	20	30	40	50
Concentration of **[d]-5** (10^{-4} M)	1.50	10.75	22.20	31.99
Concentration of **5** (10^{-4} M)	1.47	16.18	36.39	57.77

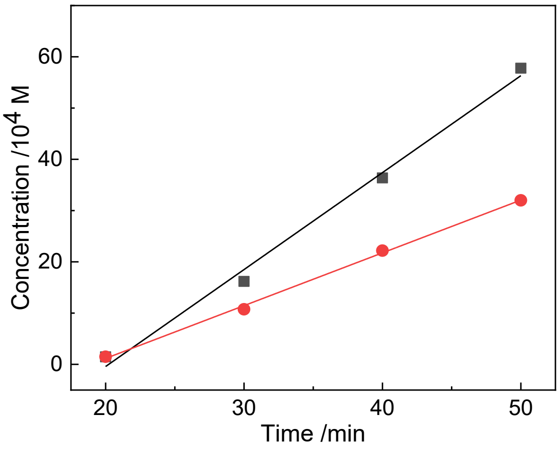

Supplementary Fig. 10 Kinetic isotope effect figure.

3.2.3 Kinetic order experiments

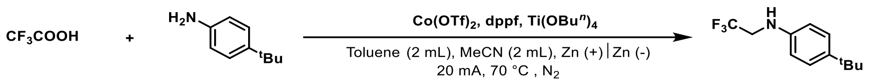

Supplementary Fig. 11 Kinetic model reaction.

In a 10-mL glass tube equipped with a magnetic stir bar, 4-tert-butylaniline, $Co(OTf)_2$, and dppf were added sequentially. To this mixture, toluene (2.0 mL), acetonitrile (2.0 mL), $Ti(OBu^n)_4$, trifluoroacetic acid (TFA), and dodecane (46 μL, used as an internal standard) were subsequently introduced. The tube was equipped with anode (Zn, 2.5×1.0×0.05 cm) and cathode (Zn, 2.5×1.0 ×0.05 cm). Prior to electrolysis, the reaction mixture was sparged with nitrogen for three minutes, after which the tube was wrapped with tape. Electrolysis was performed at a constant current of 20 mA and a temperature of 70°C. Aliquots of 0.1 mL from the reaction mixture were withdrawn

at intervals of 20, 30, 40, and 50 minutes post-initiation of the reaction. The crude reaction mixture was analyzed by gas chromatography (GC). The initial reaction rate was determined from the slope of the concentration versus time plot. Reaction orders were derived from the relationship between the initial reaction rate and reactant concentrations.

Determination of the reaction order in Co catalyst

The reaction was carried out using 4-tert-butylaniline (0.2 mmol), trifluoroacetic acid (4 mmol, 300 μL), $Ti(OBu^n)_4$ (0.2 mmol), dodecane (46 μL, used as an internal standard) and varying concentrations of $Co(OTf)_2$ and dppf (keep [Co]:dppf = 1:1) (1.86×10^{-3} M, 3.26×10^{-3} M, 4.65×10^{-3} M, and 6.05×10^{-3} M) in a solvent mixture of toluene (2.0 mL) and acetonitrile (2.0 mL). The initial rates exhibited a linear relationship with the catalyst concentration, indicating first-order kinetics with respect to the catalyst.

Supplementary Table 10 The concentrations of product **5** at different times under various catalyst amounts.

Cat. (10^{-3} M) \ Product (10^{-4} M) \ Time	20 min	30 min	40 min	50 min
1.86	1.50	10.66	19.94	33.30
3.26	3.54	18.75	30.02	48.11
4.65	1.47	16.18	36.39	57.77
6.05	6.35	30.66	51.22	73.08

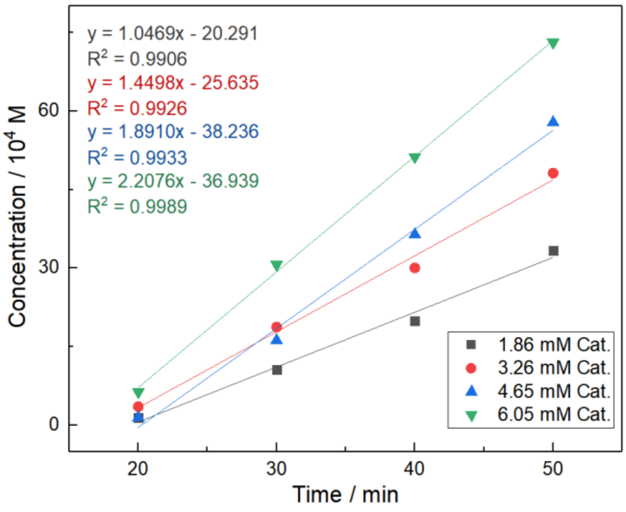

Supplementary Fig. 12 The figure for above data with different catalyst concentrations.

Supplementary Table 11 The initial rates with different catalyst concentrations.

Catalyst (10^{-3} M)	Rate (10^{-4} M/min)
1.86	1.05
3.26	1.45
4.65	1.89
6.05	2.21

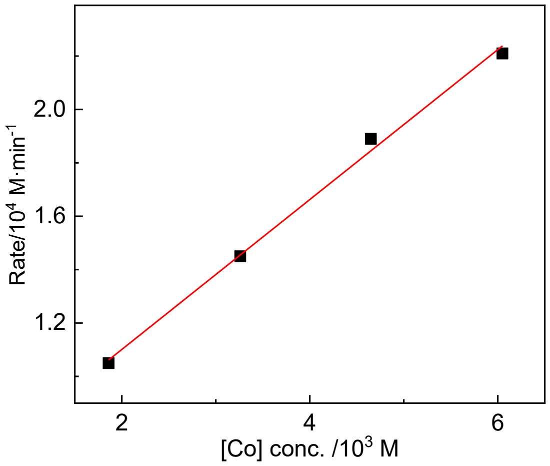

Supplementary Fig. 13 Reaction order on [Co].

Determination of the reaction order in trifluoroacetic acid

The reaction was carried out using $Co(OTf)_2$ (0.02 mmol), dppf (0.02 mmol), 4-tert-butylaniline (0.2 mmol), $Ti(OBu^n)_4$ (0.2 mmol), dodecane (46 μL, used as an internal standard) and varying concentrations of trifluoroacetic acid (0.47 M, 0.70 M, 0.93 M, and 1.16 M) in a solvent mixture of toluene (2.0 mL) and acetonitrile (2.0 mL). The initial rates exhibited a linear relationship with trifluoroacetic acid concentration, indicating first-order kinetics with respect to trifluoroacetic acid.

Supplementary Table 12 The concentrations of product **5** at different times under different TFA concentrations.

Product (10^{-4} M) / Time / TFA (M)	20 min	30 min	40 min	50 min
0.47	0.67	9.81	24.25	32.66
0.70	0.69	12.02	26.14	44.67
0.93	1.47	16.18	36.39	57.77
1.16	2.61	19.06	39.17	66.18

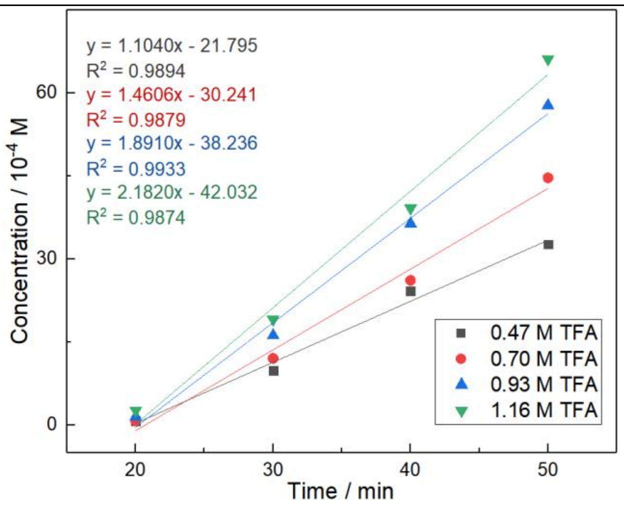

Supplementary Fig. 14 The figure for above data with different TFA concentrations.

Supplementary Table 11 The initial rates with different TFA concentrations.

TFA (M)	Rate (10^{-4} M/min)
0.47	1.10
0.70	1.46
0.93	1.89
1.16	2.18

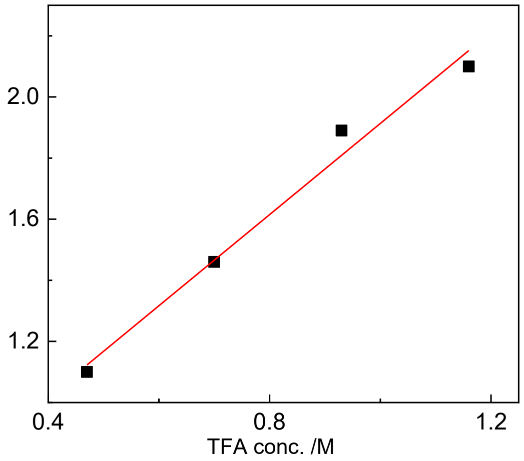

Supplementary Fig. 15 Reaction order on [TFA].

Determination of the reaction order in 4-tert-butylaniline

The reaction was carried out using $Co(OTf)_2$ (0.02 mmol), dppf (0.02 mmol), trifluoroacetic acid (4 mmol, 300 µL), $Ti(OBu^n)_4$ (0.2 mmol), dodecane (46 µL, used as an internal standard) and varying concentrations of 4-tert-butylaniline (3.20×10^{-2} M, 4.65×10^{-2} M, 5.52×10^{-2} M, and 6.98×10^{-2} M) in a solvent mixture of toluene (2.0 mL) and acetonitrile (2.0 mL). The observed initial rates remained consistent across these concentrations, suggesting zero-order kinetics with respect to 4-tert-butylaniline.

Supplementary Table 12 The concentrations of product **5** at different times under different amine concentrations.

Product (10^{-4} M) / Time / amine (10^{-2} M)	20 min	30 min	40 min	50 min
3.20	2.44	22.50	39.02	59.16
4.65	1.47	16.18	36.39	57.77
5.52	2.65	16.56	38.23	58.08
6.98	1.27	11.52	27.60	49.34

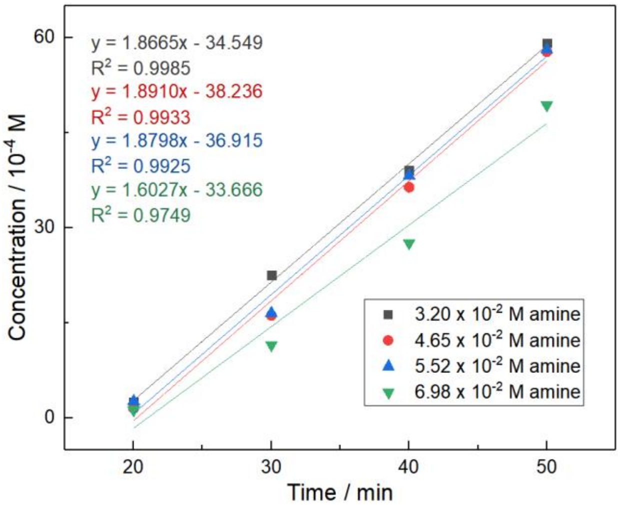

Supplementary Fig. 16 The figure for above data with different amine concentrations.

Supplementary Table 13 The initial rates with different amine concentrations.

amine (10^{-2} M)	Rate (10^{-4} M/min)
3.20	1.86
4.65	1.89
5.52	1.88
6.98	1.60

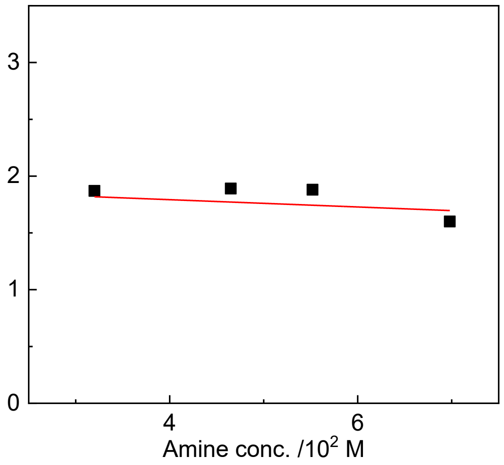

Supplementary Fig. 17 Reaction order on [amine].

Determination of the reaction order in $Ti(OBu)_4$

The reaction was carried out using 4-tert-butylaniline (0.2 mmol), $Co(OTf)_2$ (0.02 mmol), dppf (0.02 mmol), trifluoroacetic acid (4 mmol, 300 μL), dodecane (46 μL, used as an internal standard) and varying concentrations of $Ti(OBu^n)_4$ (3.72×10^{-2} M, 4.65×10^{-2} M, 5.58×10^{-2} M, and 6.51×10^{-2} M) in a solvent mixture of toluene (2.0 mL) and acetonitrile (2.0 mL). The observed initial rates remained consistent across these concentrations, suggesting zero-order kinetics with respect to $Ti(OBu^n)_4$.

Supplementary Table 14 The concentrations of product **5** under different additive concentrations.

Product (10^{-4} M) / Time / Ti(OBu^n)$_4$ (10^{-2} M)	20 min	30 min	40 min	50 min
3.72	6.14	24.09	44.30	66.15
4.65	1.47	16.18	36.39	57.77
5.58	4.94	20.63	42.75	65.32
6.51	1.32	14.84	33.56	52.32

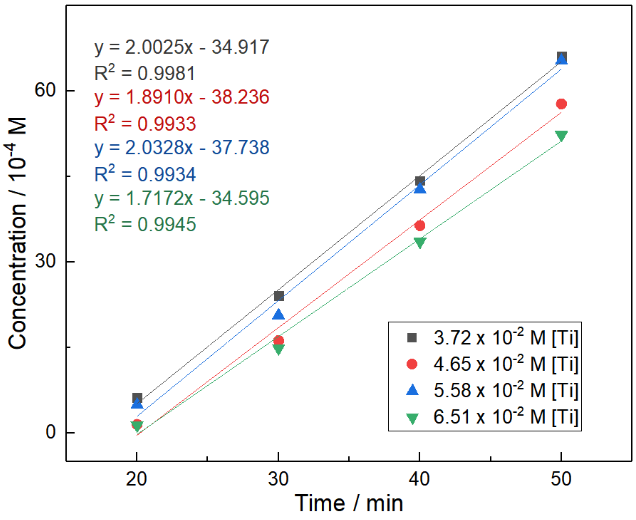

Supplementary Fig. 18 The figure for above data with different amine concentrations.

Supplementary Table 15 The initial rates with different additive concentrations.

Ti(OBu^n)$_4$ (10^{-2} M)	Rate (10^{-4} M/min)
3.72	2.00
4.65	1.89
5.58	2.03
6.51	1.72

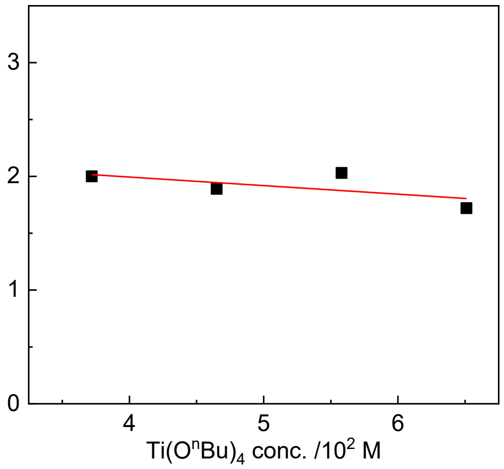

Supplementary Fig. 19 Reaction order on [Ti].

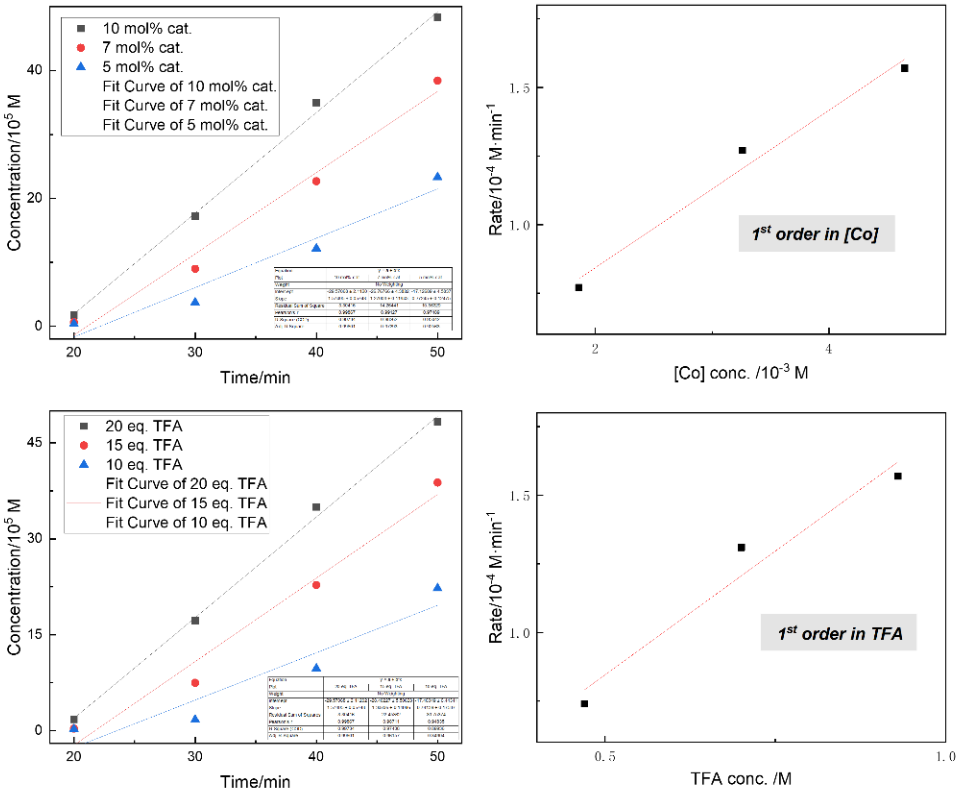

Supplementary Fig. 20 Kinetic reaction orders of TFA and cobalt catalyst at 15 mA.

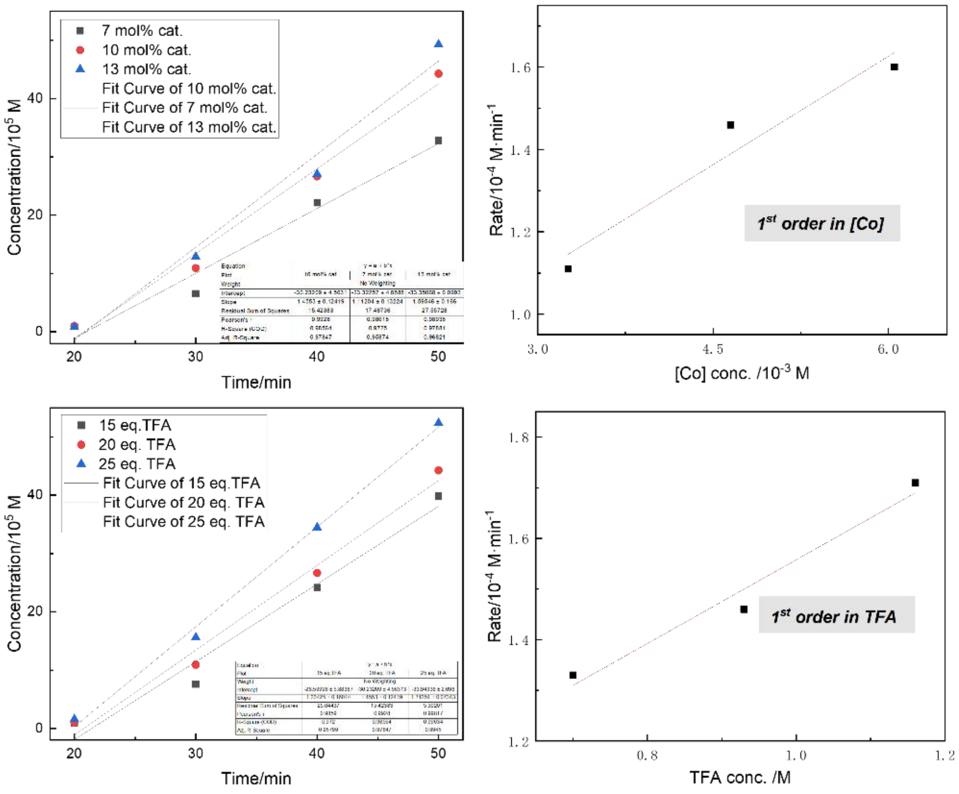

Supplementary Fig. 21 Kinetic reaction orders of TFA and cobalt catalyst at 10 mA.

As demonstrated above, both TFA and the cobalt catalyst maintain first-order kinetics at 15

mA and 10 mA, consistent with the results obtained under standard conditions at 20 mA. These results confirm that changing the current density does not alter the rate-determining step in this reaction.

3.2.4 Cyclic voltammetry experiments

Cyclic voltammetry measurements were conducted at ambient temperature using a CHI660E potentiostat. A glassy carbon electrode served as the working electrode, while a platinum wire and an Ag/AgCl electrode (in 3.5 M KCl) functioned as the counter and reference electrodes, respectively. Prior to the experiments, the glassy carbon electrode was polished with aluminum oxide powder. The samples were prepared by dissolving the substrate in 10 mL of 0.1 M tetrabutylammonium tetrafluoroborate ($TBABF_4$) in acetonitrile. All experiments were bubbled with N_2 for 10 minutes before data collection. Unless otherwise noted, scan rate = 400 mV/s.

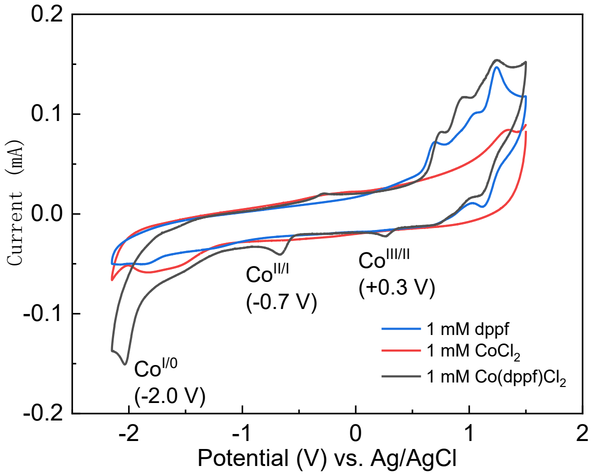

Supplementary Fig. 22 Cyclic voltammetry of cobalt complex and dppf (Blue line: 1 X 10^{-3} M dppf and 0.1 M $TBABF_4$ in 10 mL MeCN; Red line: 1 X 10^{-3} M $CoCl_2$ and 0.1 M $TBABF_4$ in 10 mL MeCN; Black line: 1 X 10^{-3} M Co(dppf)Cl_2 and 0.1 M $TBABF_4$ in 10 mL MeCN).

Measurement of CV curve for $CoCl_2$ showed weak redox peaks in a wide range scan from +1.5 to −2.2 V vs. Ag/AgCl (red line). Notably, the application of a diphosphine ligated Co(dppf)Cl_2 complex[3] presented obvious three cathodic peaks at +0.3 V, -0.7 V and -2.0 V vs. Ag/AgCl corresponding to the Co(III/II), Co(II/I) and Co(I/0) redox processes (black line).

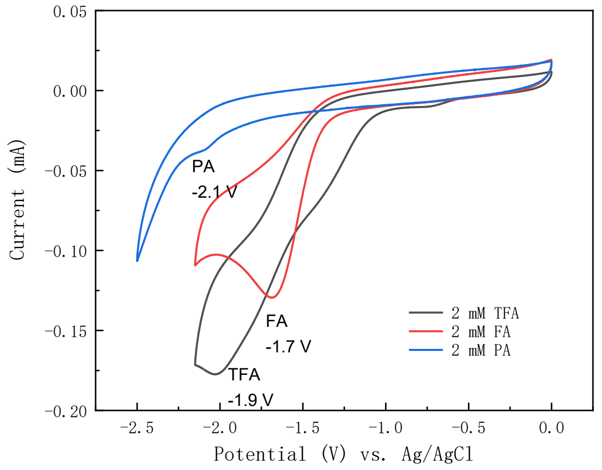

Supplementary Fig. 23 Cyclic voltammetry of carboxylic acids (Black line: 2 X 10^{-3} M trifluoroacetic acid and 0.1 M $TBABF_4$ in 10 mL MeCN ; Red line: 2 X 10^{-3} M formic acid and 0.1 M $TBABF_4$ in 10 mL MeCN; Black line: 2 X 10^{-3} M propionic acid and 0.1 M $TBABF_4$ in 10 mL MeCN).

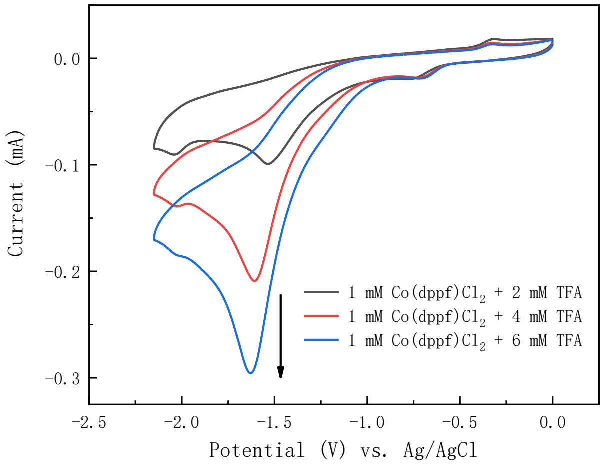

Supplementary Fig. 24 Cyclic voltammetry of cobalt complex in the presence of increasing equivalents of trifluoroacetic acid (Black line: 2 X 10^{-3} M trifluoroacetic acid, 1 X 10^{-3} M $Co(dppf)Cl_2$ and 0.1 M $TBABF_4$ in 10 mL MeCN; Red line: addition of 2 X 10^{-3} M trifluoroacetic acid to the black line condition; Blue line: addition of 2 X 10^{-3} M trifluoroacetic acid to the red line condition).

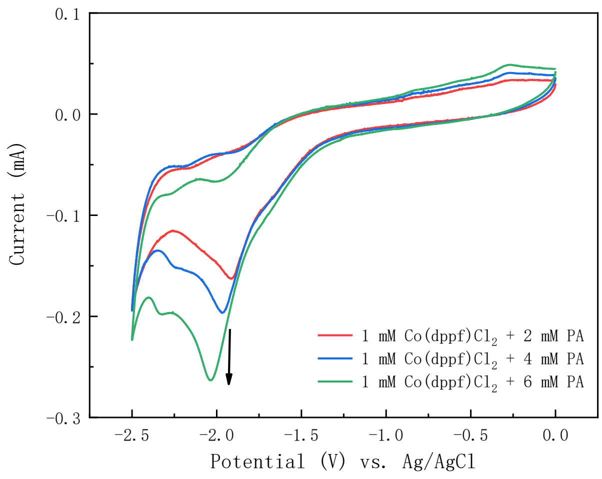

Supplementary Fig. 25 Cyclic voltammetry of cobalt complex in the presence of increasing equivalents of propionic acid (Red line: 2 X 10^{-3} M propionic acid, 1 X 10^{-3} M Co(dppf)Cl_2 and 0.1 M $TBABF_4$ in 10 mL MeCN; Blue line: addition of 2 X 10^{-3} M propionic acid to the red line condition; Green line: addition of 2 X 10^{-3} M propionic acid to the blue line condition).

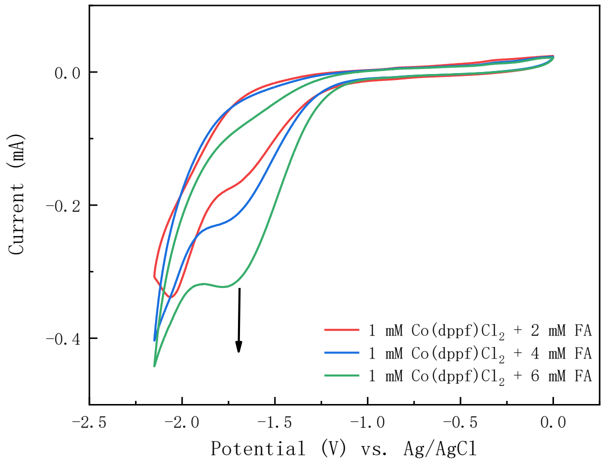

Supplementary Fig. 26 Cyclic voltammetry of cobalt complex in the presence of increasing equivalents of formic acid (Red line: 2 X 10^{-3} M formic acid, 1 X 10^{-3} M Co(dppf)Cl_2 and 0.1 M $TBABF_4$ in 10 mL MeCN; Blue line: addition of 2 X 10^{-3} M formic acid to the red line condition; Green line: addition of 2 X 10^{-3} M formic acid to the blue line condition).

3.2.5 NMR experiments

To a 10-mL glass tube equipped with a magnetic stir bar was charged with $Co(OTf)_2$ (0.2 mmol) and dppf (0.2 mmol). To the resulting mixture was added sequentially d^3-acetonitrile (4.0 mL) and trifluoroacetic acid (4.0 mmol, 20 equiv). The tube was equipped with anode (Zn, 2.5×1.0×0.05 cm) and cathode (Zn, 2.5×1.0×0.05 cm). The reaction mixture was purged with N_2 for three minutes, and tube was wrapped with tape. The electrolysis was carried out using a constant current of 20 mA for 1.5 h at 70°C. Upon cooling to room temperature, the reaction solution was carefully transferred using a syringe into an NMR tube for ^{1}H NMR analysis.

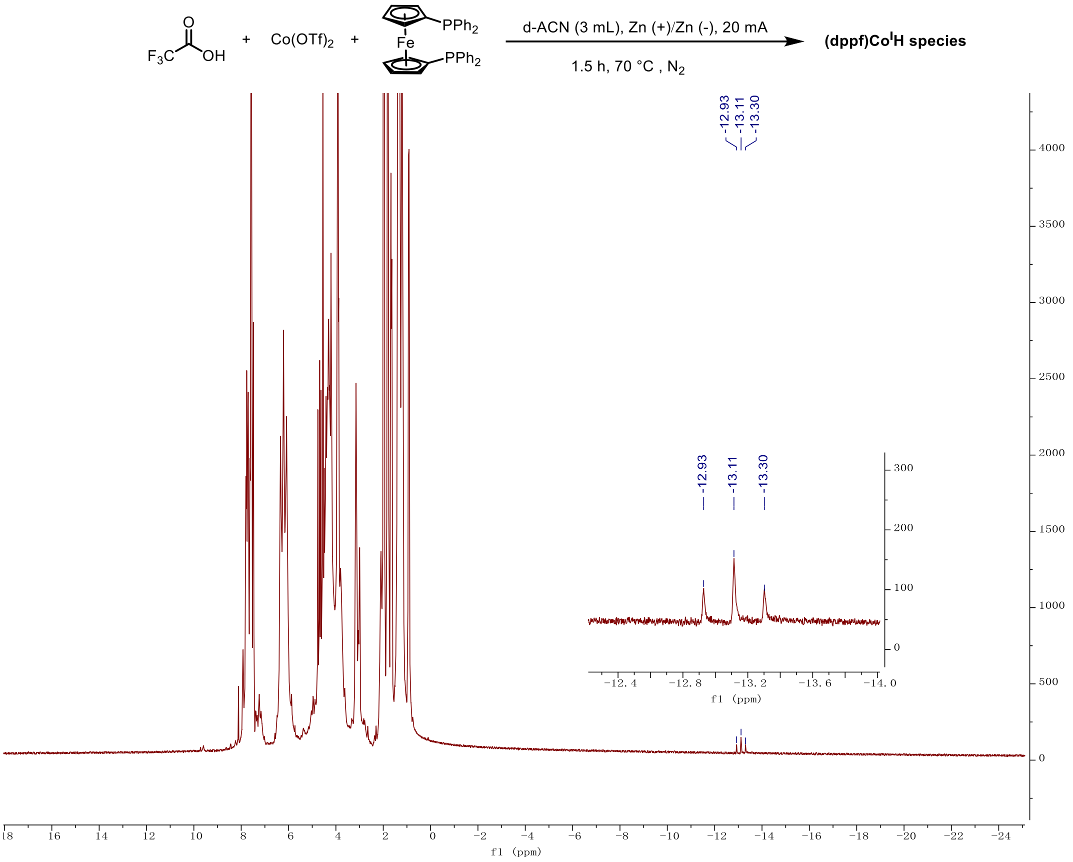

Supplementary Fig. 27 ^{1}H NMR (400 MHz, CD_3CN) for direct electrolysis of TFA with cobalt complex.

To a 10-mL glass tube equipped with a magnetic stir bar was charged with $Co(OTf)_2$ (0.2 mmol) and dppf (0.2 mmol). To the resulting mixture was added sequentially d^3-acetonitrile (4.0 mL), HFIP (0.2 mL), and propionic acid (2.0 mmol, 10 equiv) and $BF_3 \cdot Et_2O$ (1.0 mmol, 5 equiv) . The tube was equipped with anode (Zn, 2.5×1.0×0.05 cm) and cathode (Zn, 2.5×1.0×0.05 cm). The reaction mixture was purged with N_2 for three minutes, and tube was wrapped with tape. The electrolysis was carried out using a constant current of 80 mA for 0.5 h at 100°C. Upon cooling to room temperature, the reaction solution was carefully transferred using a syringe into an NMR tube for ^1H NMR analysis.

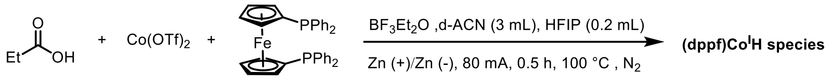

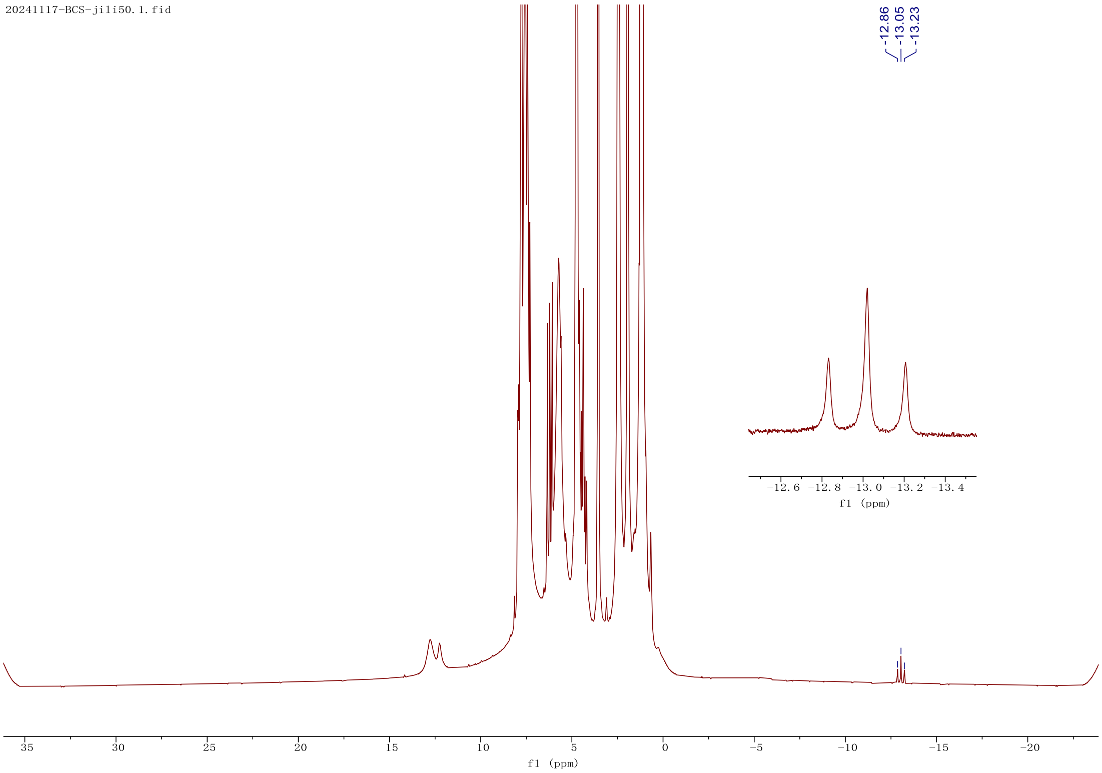

Supplementary Fig. 28 ^1H NMR (400 MHz, CD_3CN) for direct electrolysis of propionic acid with cobalt complex.

To a 10-mL glass tube equipped with a magnetic stir bar was charged with compound **2** (0.2 mmol, 1.0 equiv), $Co(OTf)_2$ (0.02 mmol, 10 mol%) and dppf (0.02 mmol, 10 mol%). To the resulting mixture was added d^3-acetonitrile (CD_3CN, 4.0 mL) and trifluoroacetic acid (4.0 mmol, 20 equiv) . The tube was equipped with anode (Zn, 2.5×1.0×0.05 cm) and cathode (Zn, 2.5×1.0×0.05 cm). Prior to electrolysis, the reaction mixture was sparged with N_2 for 3 minutes, and the tube was wrapped with tape. The electrolysis was carried out using a constant current of 20 mA for 1.5 h at 70°C. Upon cooling to room temperature, the reaction solution was carefully transferred using a syringe into an NMR tube for ^{19}F NMR analysis (trifluoromethylbenzene was added as an internal standard). The NMR spectrum indicates that trifluoroacetaldehyde (CF_3CHO) and trifluoroethanol (CF_3CH_2OH) were formed in the reaction system.

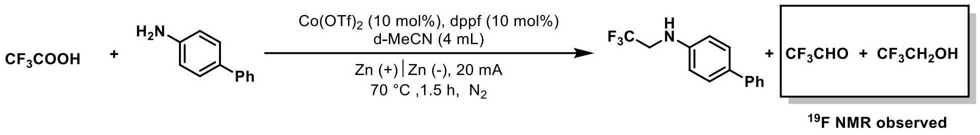

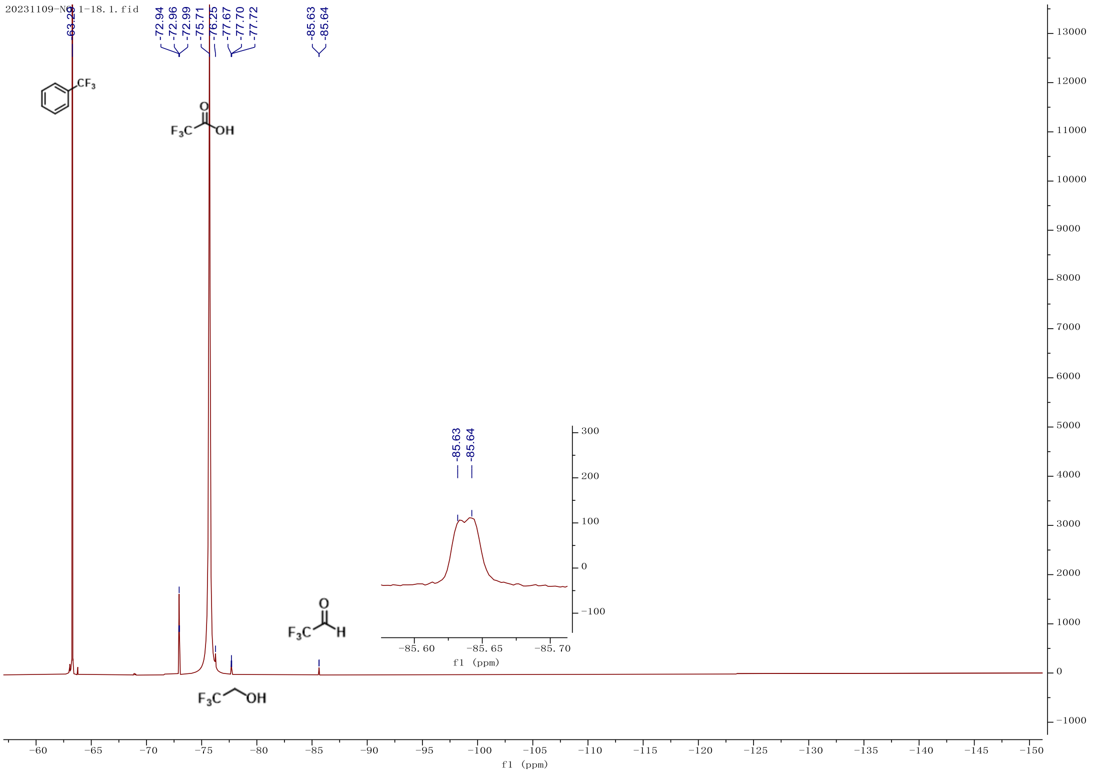

Supplementary Fig. 29 ^{19}F NMR (376 MHz, CD_3CN) for observation of aldehyde and alcohol products by acid reduction

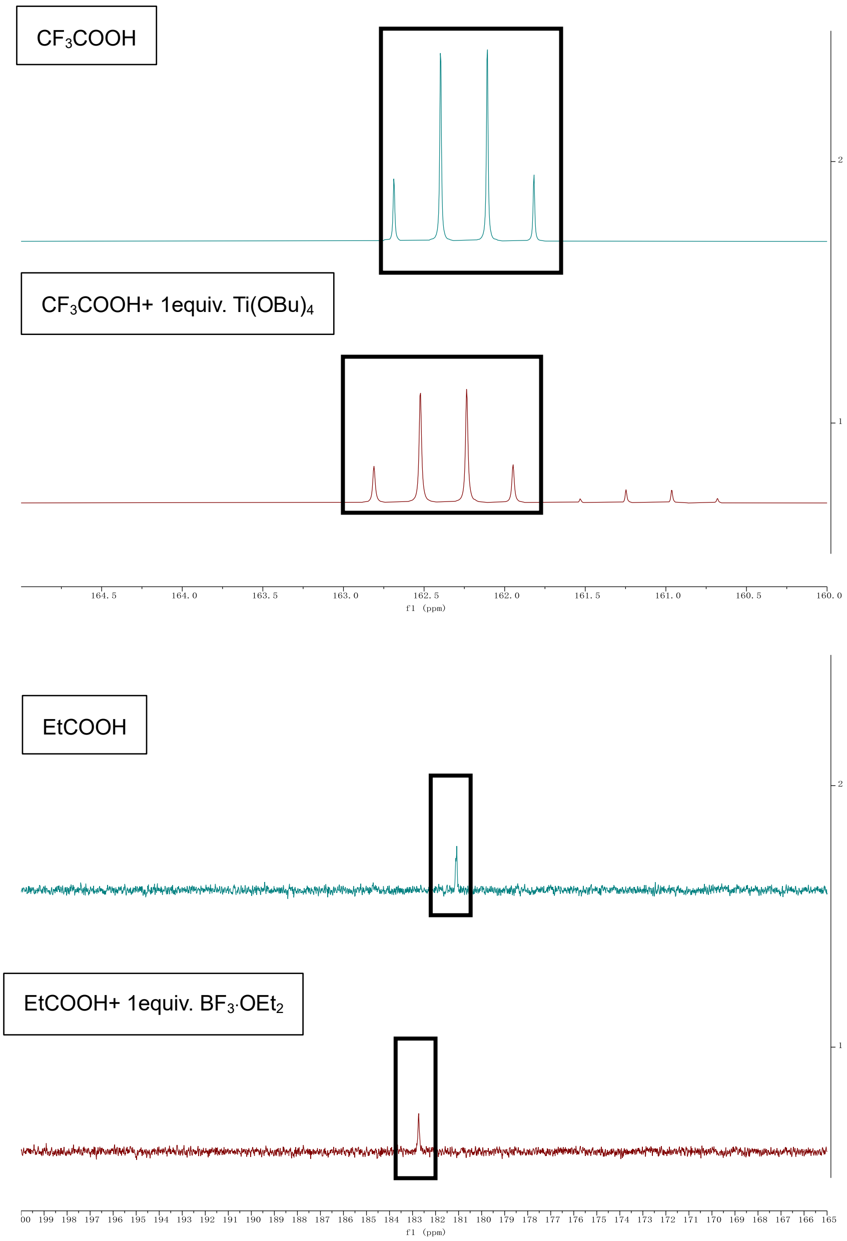

Supplementary Fig. 30 ^{13}C NMR (101 MHz, $CDCl_3$) experiments for the interaction between carboxylic acids and Lewis acids.

3.3 DFT calculations

3.3.1 Computational methods.

All of the DFT calculations were carried out with the Gaussian 16 software package[4]. All structures were optimized in the gas phase at the level of B3LYP-GD3(BJ)/BSI[5-8], where BSI denotes a basis set combination of SDD for Co and Fe atoms and 6-31G(d) for the main group atoms. Frequency calculations were performed at the same level to verify the nature of all optimized stationary points as either minima or transition-state (TS) structures, having zero and one imaginary frequencies, respectively, and to obtain thermal contributions to free energies at 298.15 K and 1 atm. Intrinsic reaction coordinate (IRC) was conducted to ensure that every transition state was connected to the right intermediate. The improved electronic energy of all structures was computed at M06-L[9]/BSII single-point calculations including solvation effects with the SMD[10] continuum solvation model. BSII represents a basis set combination of SDD for Co and Fe and 6-311++G(d,p) for all other atoms. The SMD computations for solvents Toluene: Acetonitrile = 1:1 were undertaken. The solvent parameters were calculated as a weighted average based on a 1:1 volume ratio of toluene to acetonitrile. The specific solvent parameters were defined as follows: eps = 19.03, epsinf = 2.02, HBondAcidity = 0.035, HBondBasicity = 0.23, SurfaceTensionAtInterface = 40.725, and CarbonAromaticity = 0.4285. These values were derived from the Minnesota Solvent Descriptor Database[11]. The free energy reported in this work is the sum of the electronic energy obtained from single-point calculations and the thermal correction to the Gibbs free energy derived from frequency calculations.

3.3.2 Competitive reaction pathways starting from dimer, Int-1 and Int-2

We have analyzed different spin states of the Co(I) species, and the results indicate that the ground states of both the **dimer** and **Int-1** are singlet states. As shown below, the energy of the quintet state structure of the dimer is 22.7 kcal/mol higher than that of its singlet state, and the energy of the triplet state structure of **Int-1** is also 23.5 kcal/mol higher than that of its singlet state. This is because the bidentate phosphine ligand is a strong-field ligand, and the Co(I) center tends to be in a low-spin state. It can be anticipated that the ground states of other Co(I) species are also singlet states; therefore, the potential energy surface calculations in this study only considered singlet states. In addition, we have compared the different coordination modes of Co(I) species and those starting from **Int-2**, and the computational results showed that the reaction pathway reported in the main text is the most energetically favorable.

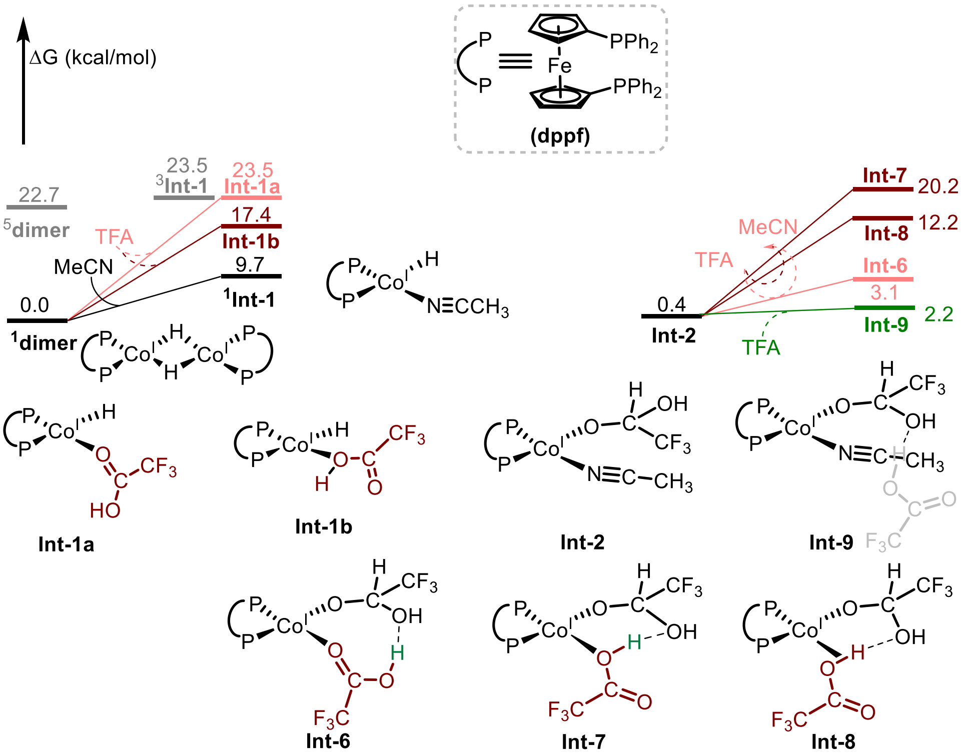

Supplementary Fig. 31 The values given by kcal/mol are the relative free energies calculated by M06-L/6-311++G(d,p)-SDD(Co, Fe)-SMD(Toluene: Acetonitrile = 1:1)//B3LYP-D3(BJ)/6-31G(d)-SDD(Co, Fe) level of theory.

We also compared the reaction pathways for the reduction of acetonitrile and trifluoroacetic acid (as illustrated in Fig. 32). The computational results reveal that the reaction barrier for the reduction of acetonitrile is higher (with **TS-3** being 2.4 kcal/mol higher than **TS-2**). Moreover, the reduction of acetonitrile process is thermodynamically disfavored, as the energy increases by 7.9 kcal/mol from **Int-1** to **Int-5**. Consequently, the reduction of acetonitrile process exhibits a strong tendency to revert to **Int-1**, thereby directing the overall reaction toward the reduction of trifluoroacetic acid.

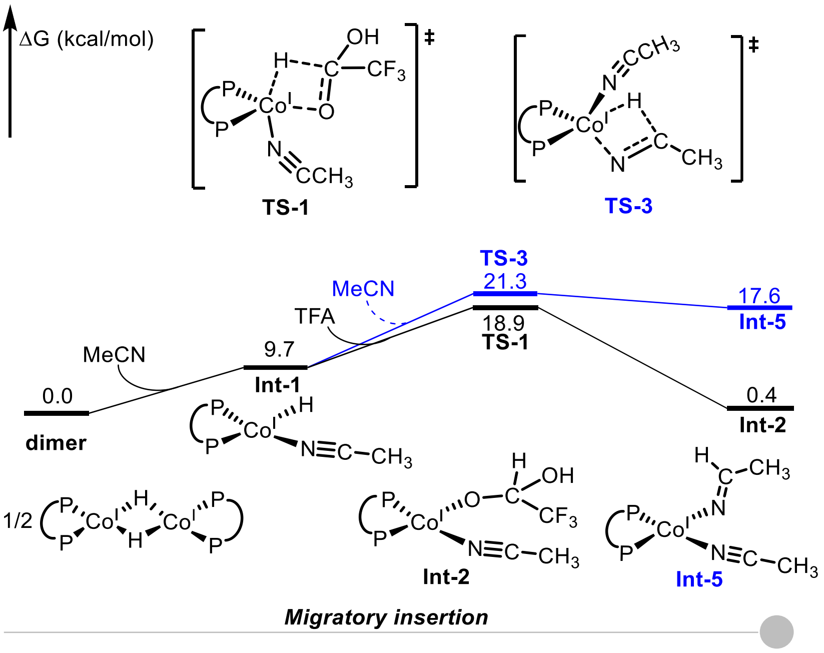

Supplementary Fig. 32 The values given by kcal/mol are the relative free energies calculated by M06-L/6-311++G(d,p)-SDD(Co, Fe)-SMD(Toluene: Acetonitrile = 1:1)//B3LYP-D3(BJ)/6-31G(d)-SDD(Co, Fe) level of theory.

3.3.3 The reduction of the imine

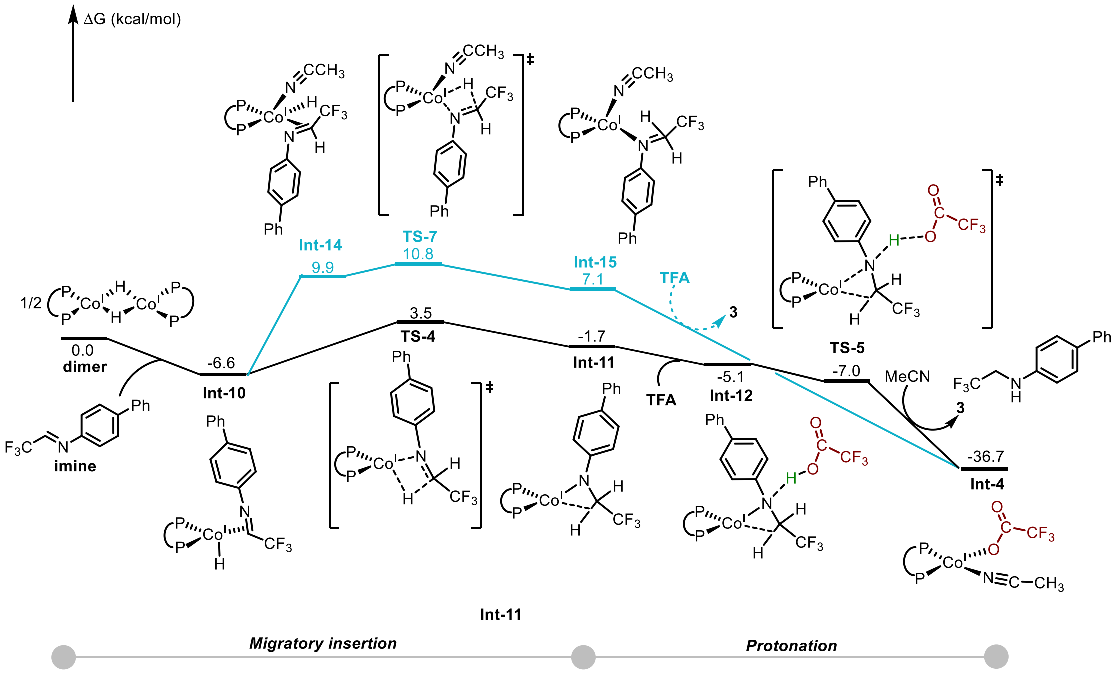

Supplementary Fig. 33 The calculated free energy profiles of the imine reduction process. The values given by kcal/mol are calculated by M06-L/6-311++G(d,p)-SDD(Co,Fe)-SMD(Toluene: Acetonitrile = 1:1)//B3LYP-D3(BJ)/6-31G(d)-SDD(Co,Fe) level of theory.

3.3.4 Supplementary calculation results

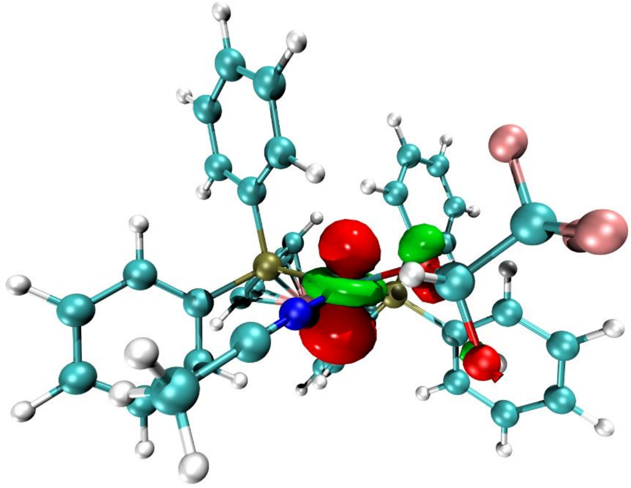

Supplementary Fig. 34 The HOMO orbital analysis of **Int-2**.

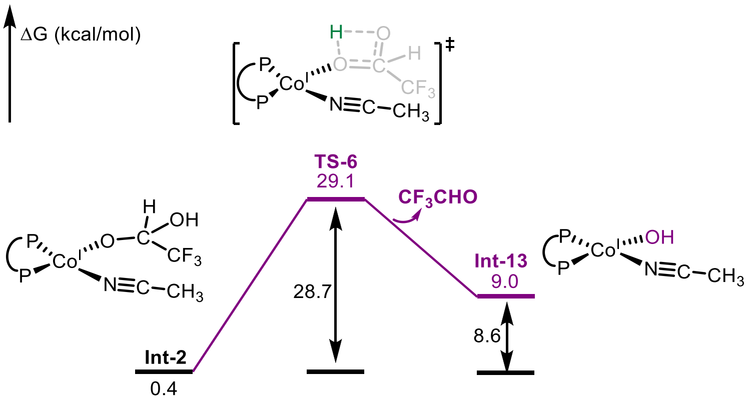

Supplementary Fig. 35 The calculated free energy profile of the intramolecular 1,3-H shift pathway from intermediate **Int-2**.

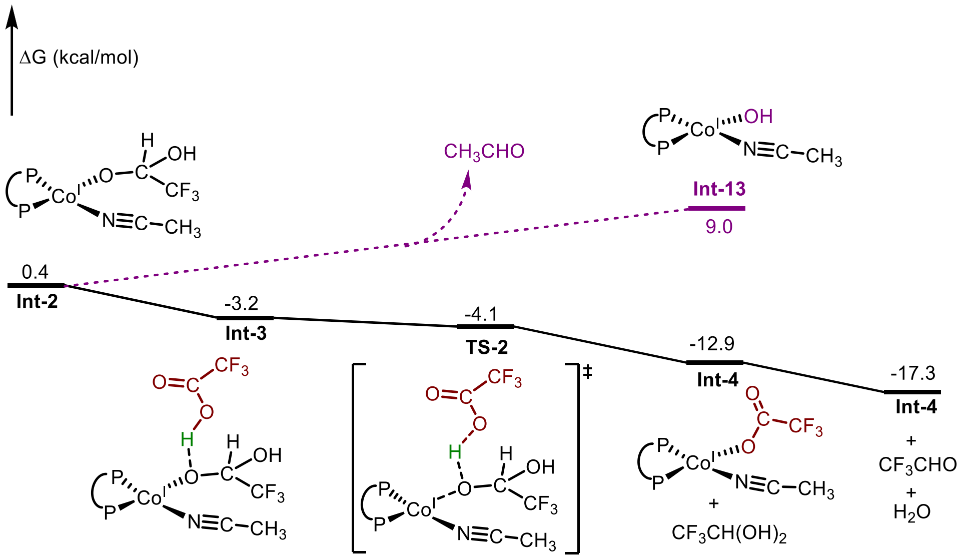

Supplementary Fig. 36 Comparison of the β-O elimination pathway (the purple one) with the protoantion pathway (the black one).

3.3.5 B3LYP-D3 and M06-L calculated absolute energies, enthalpies, and free energies for all structures

Supplementary Table 16 Calculated absolute energies, enthalpies, and free energies.

Geometry	$E_{(elec\text{-}B3\text{-}LYP\text{-}D3)}{}^{a}$	$H_{(corr\text{-}B3\text{-}LYP\text{-}D3)}{}^{b}$	$G_{(cor\text{-}B3\text{-}LYP\text{-}D3)}{}^{c}$	$E_{(solv\text{-}M06\text{-}L)}{}^{d}$	IF[e]
**1**	-526.789774	0.046456	0.007765	-526.901010	
**acetonitrile**	-132.760396	0.049986	0.022462	-132.784069	
**CF_3CHO**	-451.536600	0.039815	0.003730	-451.628943	
**CF_3COO^-**	-526.237759	0.033000	-0.006068	-526.447084	
**H_2O**	-76.418734	0.025163	0.003729	-76.448733	
**2**	-518.718502	0.209854	0.162442	-518.74034	
**3**	-895.069424	0.248525	0.188057	-895.154173	
**dimer**	-4531.653121	1.126291	0.953919	-4531.777186	
**dimer[e]**	-4531.695169	1.123382	0.94676	-4531.748086	
**Int-1**	-2398.563413	0.613112	0.501003	-2398.658828	
**Int-1[c]**	-2398.537877	0.611345	0.496001	-2398.617621	
**Int-1a**	-2792.581027	0.609161	0.490925	-2792.758293	
**Int-1b**	-2792.596398	0.61121	0.49274	-2792.769926	
**TS-1**	-2925.38907	0.661268	0.535184	-2925.571593	688.7*i*
**Int-2**	-2925.417735	0.665537	0.535964	-2925.601865	
**Int-3**	-3452.265786	0.711253	0.566142	-3452.530929	
**TS-2**	-3452.265669	0.709093	0.56486	-3452.531162	384.5*i*
**TS-3**	-2531.340226	0.66417	0.54623	-2531.447058	241.4*i*
**Int-4**	-2924.236772	0.642342	0.513401	-2924.430522	
**Int-5**	-2531.355759	0.668256	0.547358	-2531.454168	
**Int-6**	-3319.459333	0.661082	0.527853	-3319.721047	
**Int-7**	-3319.452189	0.66158	0.532156	-3319.698023	
**Int-8**	-3319.45739	0.661294	0.532156	-3319.710904	
**Imine**	-893.835191	0.223603	0.163855	-893.922074	
**Int-9**	-3452.246559	0.713631	0.565903	-3452.522125	
**Int-10**	-3159.692144	0.788763	0.655614	-3159.835988	
**TS-4**	-3159.673463	0.786466	0.652763	-3159.817098	674.1*i*
**Int-11**	-3159.686213	0.790438	0.656804	-3159.829425	
**Int-12**	-3686.514898	0.837841	0.683974	-3686.755248	
**Int-13**	-2473.820124	0.62178	0.507165	-2473.934793	
**Int-14**	-3292.45051	0.839884	0.696601	-3292.612231	
**Int-15**	-3292.464667	0.842568	0.69916	-3292.61935	
**TS-5**	-3686.51462	0.835109	0.682152	-3686.756409	643.2*i*
**TS-6**	-2925.369289	0.66027	0.53329	-2925.553994	1495.2*i*
**TS-7**	-3292.450493	0.838672	0.69734	-3292.611651	98.2*i*

[a] The electronic energy calculated by B3LYP-D3(BJ) in gas phase. [b] The thermal correction to enthalpy calculated by B3-LYP-D3 in gas phase. [c] The thermal correction to Gibbs free energy calculated by B3-LYP-D3 in gas phase. [d] The electronic energy calculated by M06-L in Toluene: Acetonitrile = 1:1 solvent. [e] The B3-LYP-D3 calculated imaginary frequencies for the transition states.

3.4 Characterization of products

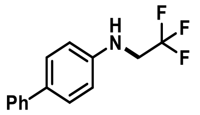

N-(2,2,2-trifluoroethyl)-[1,1'-biphenyl]-4-amine

Compound 3: followed the condition A, 0.2 mmol reaction scale, 91% yield, 45.9 mg, white solid. Purified by column chromatography on silica gel eluting with petroleum ether/dichloromethane (20:1). Reported compound[12].

^{1}H NMR (400 MHz, $CDCl_3$) δ 7.56 (d, *J* = 7.7 Hz, 2H), 7.50-7.46 (m, 2H), 7.42 (t, *J* = 7.6 Hz, 2H), 7.30 (t, *J* = 7.4 Hz, 1H), 6.79-6.75 (m, 2H), 4.01 (s, 1H), 3.83-3.65 (m, 2H).

^{13}C NMR (101 MHz, $CDCl_3$) δ 145.6, 140.9, 132.2, 129.2, 128.7, 128.1, 126.5, 125.1 (q, *J* = 281.0 Hz), 113.5, 46.1 (q, *J* = 33.3 Hz).

^{19}F NMR (376 MHz, $CDCl_3$) δ -72.26 (t, *J* = 8.9 Hz).

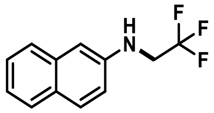

N-(2,2,2-trifluoroethyl)naphthalen-2-amine

Compound 4: followed the condition A, 0.2 mmol reaction scale, 52% yield, 23.4 mg, white solid. Purified by column chromatography on silica gel eluting with petroleum ether/ethyl acetate (200:1). Reported compound[13].

^{1}H NMR (400 MHz, $CDCl_3$) δ 7.84-7.62 (m, 3H), 7.43 (t, J = 7.6 Hz, 1H), 7.36-7.23 (m, 1H), 6.96 (d, *J* = 5.9 Hz, 2H), 4.40-3.62 (br, 1H), 3.98-3.82 (m, 2H).

^{13}C NMR (100 MHz, $CDCl_3$) δ 143.9, 134.8, 129.3, 128.2, 127.7, 126.6, 126.2, 125.1 (q, *J* = 281.0 Hz), 122.9, 117.4, 105.6, 46.1 (q, *J* = 33.3 Hz).

^{19}F NMR (376 MHz, $CDCl_3$) δ -72.02 (t, *J* = 9.0 Hz).

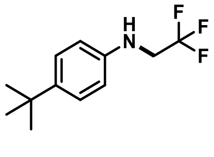

4-(tert-butyl)-N-(2,2,2-trifluoroethyl)aniline

Compound 5: followed the condition A, 0.2 mmol reaction scale, 61% yield, 28.4 mg, colorless oil. Purified by column chromatography on silica gel eluting with petroleum ether. Reported compound[12].

^{1}H NMR (400 MHz, $CDCl_3$) δ 7.29 (d, J = 8.2 Hz, 2H), 6.69 (d, *J* = 8.2 Hz, 2H), 4.06-3.67 (br, 1H), 3.83-3.72 (m, 2H), 1.34 (s, 9H).

^{13}C NMR (100 MHz, $CDCl_3$) δ 143.8, 141.9, 126.2, 125.1 (q, *J* = 281.0 Hz), 112.9, 46.1 (q, *J* = 33.3 Hz), 33.9, 31.5.

^{19}F NMR (376 MHz, $CDCl_3$) δ -72.35 (t, *J* = 9.0 Hz).

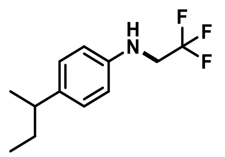

4-(sec-butyl)-N-(2,2,2-trifluoroethyl)aniline

Compound 6: followed the condition A, 0.2 mmol reaction scale, 65% yield, 29.8 mg, colorless oil. Purified by column chromatography on silica gel eluting with petroleum ether.

^{1}H NMR (400 MHz, $CDCl_3$) δ 7.06 (d, *J* = 6.8 Hz, 2H), 6.65 (d, *J* = 6.8 Hz, 2H), 3.84-3.67 (m, 2H), 3.69-2.81 (br, 1H), 2.60-2.45 (m, 1H), 1.64-1.49 (m, 2H), 1.22 (d, *J* = 6.9 Hz, 3H), 0.83 (t, *J* = 7.4 Hz, 3H).

^{13}C NMR (100 MHz, $CDCl_3$) δ 144.2, 138.4, 127.9, 125.1 (q, *J* = 281.0 Hz), 113.1, 46.1 (q, *J* = 33.3 Hz), 40.7, 31.3, 21.9, 12.2.

^{19}F NMR (376 MHz, $CDCl_3$) δ -72.35 (t, *J* = 9.0 Hz).

ESI HRMS [C12H16F3N + H^+] calculated:232.1313 , found: 232.1305

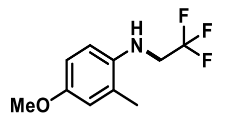

4-methoxy-2-methyl-N-(2,2,2-trifluoroethyl)aniline

Compound 7: followed the condition A, 0.2 mmol reaction scale, 64% yield, 27.9 mg, colorless oil. Purified by column chromatography on silica gel eluting with petroleum ether/ethyl acetate (100:1). Reported compound[3].

^{1}H NMR (400 MHz, $CDCl_3$) δ 6.76-6.69 (m, 2H), 6.65 (d, *J* = 9.7 Hz, 1H), 3.87-3.65 (m, 2H), 3.76 (s, 3H), 3.66-3.33 (br, 1H), 2.19 (s, 3H)

^{13}C NMR (100 MHz, $CDCl_3$) δ 152.7, 138.3, 125.1 (q, *J* = 281.0 Hz), 124.7, 117.0, 112.0, 111.6, 77.3, 77.0, 76.7, 55.6, 46.1 (q, *J* = 33.3 Hz), 17.6.

^{19}F NMR (376 MHz, $CDCl_3$) δ -72.21 (t, *J* = 8.9 Hz).

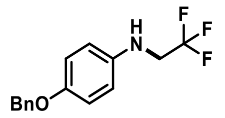

4-(benzyloxy)-N-(2,2,2-trifluoroethyl)aniline

Compound 8: followed the condition A, 0.2 mmol reaction scale, 71% yield, 40.1 mg, white solid. Purified by column chromatography on silica gel eluting with petroleum ether/ethyl acetate (50:1). Reported compound[14].

^{1}H NMR (400 MHz, $CDCl_3$) δ 7.48-7.37 (m, 4H), 7.37-7.29 (m, 1H), 6.89 (d, *J* = 8.8 Hz, 2H), 6.66 (d, *J* = 8.8 Hz, 2H), 5.02 (s, 2H),4.15-3.16 (br, 1H), 3.80-3.63 (m, 2H).

^{13}C NMR (100 MHz, $CDCl_3$) δ 152.4, 140.6, 137.4, 128.6, 127.9, 127.5, 125.1 (q, *J* = 281.0 Hz) 116.2, 114.6, 70.8, 46.1 (q, *J* = 33.3 Hz) .

^{19}F NMR (376 MHz, $CDCl_3$) δ -72.28 (t, J = 9.0 Hz).

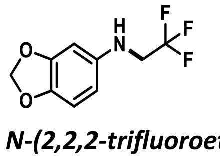

N-(2,2,2-trifluoroethyl)benzo[d][1,3]dioxol-5-amine

Compound 9: followed the condition A, 0.2 mmol reaction scale, 60% yield, 26.2 mg, colorless oil. Purified by column chromatography on silica gel eluting with petroleum ether/ethyl acetate (200:1).

^{1}H NMR (400 MHz, $CDCl_3$) δ 6.67 (d, *J* = 8.1 Hz, 1H), 6.31 (s, 1H), 6.12 (d, *J* = 8.1 Hz, 1H), 5.88 (s, 2H), 3.88-3.49 (br, 1H), 3.75-3.62 (m, 2H).

^{13}C NMR (100 MHz, $CDCl_3$) δ 148.5, 141.8, 140.8, 125.1 (q, *J* = 281.0 Hz), 108.6, 105.2, 100.9, 96.5, 77.4, 77.0, 76.7, 46.1 (q, *J* = 33.3 Hz).

^{19}F NMR (376 MHz, $CDCl_3$) δ -72.28 (t, *J* = 9.0 Hz).

ESI HRMS [C9H8F3NO2 + H^+] calculated: 220.0585 , found: 220.0576

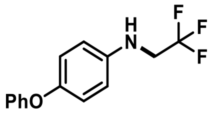

4-phenoxy-N-(2,2,2-trifluoroethyl)aniline

Compound 10: followed the condition A, 0.2 mmol reaction scale, 76% yield, 40.6 mg, colorless oil. Condition C1, 0.2 mmol reaction scale, 63% yield, 33.4 mg, colorless oil. Purified by column chromatography on silica gel eluting with petroleum ether/ethyl acetate (100:1).

^{1}H NMR (400 MHz, $CDCl_3$) δ 7.32 (t, *J* = 7.7 Hz, 2H), 7.06 (t, *J* = 7.4 Hz, 1H), 7.00-6.92 (m, 4H), 6.71 (d, *J* = 8.5 Hz, 2H), 3.89 (s, 1H), 3.83-3.70 (m, 2H).

^{13}C NMR (100 MHz, $CDCl_3$) δ 158.7, 149.1, 142.7, 129.6, 125.1 (q, *J* = 281.0 Hz), 122.3, 121.1, 117.4, 114.4, 46.1 (q, *J* = 33.3 Hz).

^{19}F NMR (376 MHz, $CDCl_3$) δ -72.29 (t, *J* = 9.0 Hz).

ESI HRMS [C14H12F3NO + H^+] calculated:268.0949 , found: 268.0953

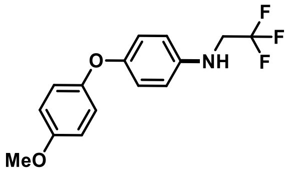

4-(4-methoxyphenoxy)-N-(2,2,2-trifluoroethyl)aniline

Compound 11: followed the condition A, 0.2 mmol reaction scale, 83% yield, 49.3 mg, colorless oil. Purified by column chromatography on silica gel eluting with petroleum ether/ethyl acetate (100:1-50:1).

^{1}H NMR (400 MHz, $CDCl_3$) δ 7.05-6.81 (m, 6H), 6.68 (d, *J* = 8.4 Hz, 2H), 3.89 (s, 1H), 3.82 (s, 3H), 3.81-3.69 (m, 2H).

^{13}C NMR (100 MHz, $CDCl_3$) δ 155.2, 151.8, 150.7, 142.1, 125.1 (q, *J* = 281.0 Hz), 119.9, 119.3, 114.8, 114.4, 55.7, 46.1 (q, *J* = 33.3 Hz).

^{19}F NMR (376 MHz, $CDCl_3$) δ -72.26 (t, *J* = 9.0 Hz)

ESI HRMS [C15H14F3NO2 + H^+] calculated:298.1055 , found: 298.1054

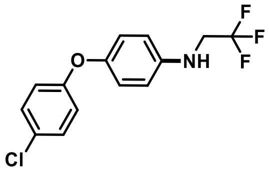

4-(4-chlorophenoxy)-N-(2,2,2-trifluoroethyl)aniline

Compound 12: followed the condition A, 0.2 mmol reaction scale, 75% yield, 45.1 mg, colorless oil. Purified by column chromatography on silica gel eluting with petroleum ether/ethyl acetate (100:1).

^{1}H NMR (400 MHz, $CDCl_3$) δ 7.27 (d, *J* = 8.8 Hz, 2H), 6.95 (d, *J* = 8.5 Hz, 2H), 6.89 (d, *J* = 8.5 Hz, 2H), 6.71 (d, *J* = 8.8 Hz, 2H), 3.93 (s, 1H), 3.83-3.65 (m, 2H).

^{13}C NMR (100 MHz, $CDCl_3$) δ 157.3, 148.7, 142.9, 129.5, 127.1, 125.1 (q, *J* = 281.0 Hz), 121.1, 118.5, 114.3, 46.1 (q, *J* = 33.3 Hz).

^{19}F NMR (376 MHz, $CDCl_3$) δ -72.26 (t, *J* = 9.0 Hz).

ESI HRMS [C14H11ClF3NO + H^{+}] calculated:302.0560 , found: 302.0560

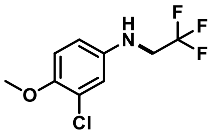

3-chloro-4-methoxy-N-(2,2,2-trifluoroethyl)aniline

Compound 13: followed the condition A, 0.2 mmol reaction scale, 68% yield, 32.7 mg, colorless oil. Purified by column chromatography on silica gel eluting with petroleum ether/ethyl acetate (50:1).

^{1}H NMR (400 MHz, $CDCl_3$) δ 6.82 (d, *J* = 8.8 Hz, 1H), 6.75 (d, *J* = 2.9 Hz, 1H), 6.56 (dd, *J* = 8.8, 2.9 Hz, 1H), 3.82 (s, 3H), 3.78-3.60 (m, 2H).

^{13}C NMR (100 MHz, $CDCl_3$) δ 148.5, 140.9, 125.1 (q, *J* = 281.0 Hz), 123.5, 115.7, 114.0, 112.4, 56.9, 46.1 (q, *J* = 33.3 Hz).

^{19}F NMR (376 MHz, $CDCl_3$) δ -72.32 (t, *J* = 8.7 Hz).

ESI HRMS [C9H9ClF3NO + H^{+}] calculated: 240.0403 , found: 240.0394

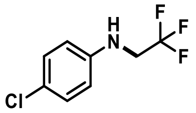

4-chloro-N-(2,2,2-trifluoroethyl)aniline

Compound 14: followed the condition A, 0.2 mmol reaction scale, 49% yield, 20.4 mg, colorless oil. Purified by column chromatography on silica gel eluting with petroleum ether. Reported compound[3].

^{1}H NMR (400 MHz, $CDCl_3$) δ 7.28 (d, *J* = 8.6 Hz, 2H), 6.64 (d, *J* = 8.6 Hz, 2H), 3.97 (s, 1H), 3.87-3.53 (m, 2H).

^{13}C NMR (100 MHz, $CDCl_3$) δ 144.8, 129.3, 125.1 (q, *J* = 281.0 Hz), 123.8, 114.3, 46.1 (q, *J* = 33.3 Hz).

^{19}F NMR (376 MHz, $CDCl_3$) δ -72.29 (t, *J* = 8.9 Hz).

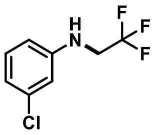

3-chloro-N-(2,2,2-trifluoroethyl)aniline

Compound 15: followed the condition A, 0.2 mmol reaction scale, 44% yield, 18.2 mg, colorless oil.

Purified by column chromatography on silica gel eluting with petroleum ether. Reported compound[3].

^{1}H NMR (400 MHz, $CDCl_3$) δ 7.15 (t, *J* = 8.0 Hz, 1H), 6.80 (d, *J* = 7.9 Hz, 1H), 6.70 (s, 1H), 6.58 (d, *J* = 7.9 Hz, 1H), 4.04 (s, 1H), 3.83-3.65 (m, 2H).

^{13}C NMR (100 MHz, $CDCl_3$) δ 147.4, 135.2, 130.4, 125.1 (q, *J* = 281.0 Hz), 119.1, 113.0, 111.4, 46.1 (q, *J* = 33.3 Hz).

^{19}F NMR (376 MHz, $CDCl_3$) δ -72.29 (t, *J* = 8.9 Hz)

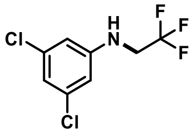

3,5-dichloro-N-(2,2,2-trifluoroethyl)aniline

Compound 16: followed the condition A, 0.2 mmol reaction scale, 37% yield, 18.2 mg, colorless oil. Purified by column chromatography on silica gel eluting with petroleum ether.

^{1}H NMR (400 MHz, $CDCl_3$) δ 6.78 (s, 1H), 6.56 (s, 2H), 4.10 (s, 1H), 3.81-3.65 (m, 2H).

^{13}C NMR (100 MHz, $CDCl_3$) δ 147.9, 135.7, 125.1 (q, *J* = 281.0 Hz), 119.0, 111.5, 46.1 (q, *J* = 33.3 Hz).

^{19}F NMR (376 MHz, $CDCl_3$) δ -72.24 (t, *J* = 8.7 Hz).

ESI HRMS [C8H6Cl2F3N + H^+] calculated: 243.9908, found:243.9903

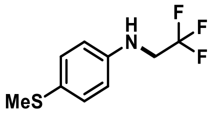

4-(methylthio)-N-(2,2,2-trifluoroethyl)aniline

Compound 17: followed the condition A, 0.2 mmol reaction scale, 70% yield, 31.1 mg, colorless oil. Purified by column chromatography on silica gel eluting with petroleum ether/ethyl acetate (50:1).

^{1}H NMR (400 MHz, $CDCl_3$) δ 7.23 (d, *J* = 7.2 Hz, 2H), 6.64 (d, *J* = 7.2 Hz, 2H), 4.17-3.84 (br, 1H), 3.83-3.65 (m, 2H), 2.42 (s, 3H).

^{13}C NMR (100 MHz, $CDCl_3$) δ 144.9, 130.9, 126.6, 125.1 (q, *J* = 281.0 Hz), 113.8, 46.1 (q, *J* = 33.3 Hz), 18.5.

^{19}F NMR (376 MHz, $CDCl_3$) δ -72.29 (t, *J* = 9.0 Hz).

ESI HRMS [C9H10F3NS + H^+] calculated:222.0564 , found: 222.0557

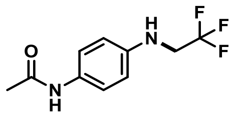

N-(4-((2,2,2-trifluoroethyl)amino)phenyl)acetamide

Compound 18: followed the condition A, 0.2 mmol reaction scale, 77% yield, 35.9 mg, white solid. Purified by column chromatography on silica gel eluting with petroleum ether/ethyl acetate (2:1). Reported compound[15].

^{1}H NMR (400 MHz, DMSO-d^6) δ 9.59 (s, 1H), 7.29 (d, *J* = 8.4 Hz, 2H), 6.64 (d, *J* = 8.4 Hz, 2H), 6.01 (t, *J* = 7.0 Hz, 1H), 3.95-3.76 (m, 2H), 1.96 (s, 3H).

^{13}C NMR (100 MHz, DMSO-d^6) δ 167.9, 144.0, 130.2, 126.4 (q, *J* = 281.0 Hz) 121.2, 112.8, 45.0 (q, *J* = 33.3 Hz), 24.1.

^{19}F NMR (376 MHz, DMSO-d^6) δ -70.63 (t, *J* = 9.0 Hz).

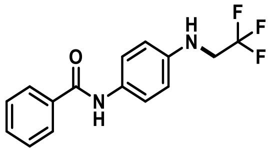

N-(4-((2,2,2-trifluoroethyl)amino)phenyl)benzamide

Compound 19: followed the condition A, 0.2 mmol reaction scale, 71% yield, 42.0 mg, white solid. Purified by column chromatography on silica gel eluting with petroleum ether/ethyl acetate (10:1-5:1).

^{1}H NMR (400 MHz, DMSO-d^6) δ 9.98 (s, 1H), 7.94 (d, *J* = 6.7 Hz, 2H), 7.62-7.43 (m, 5H), 6.73 (d, *J* = 8.9 Hz, 2H), 6.13 (s, 1H), 3.99-3.81 (m, 2H).

^{13}C NMR (100 MHz, DMSO-d^6) δ 170.0, 149.3, 140.4, 136.4, 134.6, 133.5, 132.7, 131.0 (q, *J* = 281.0 Hz), 127.3, 117.5, 50.1, 49.6 (q, *J* = 32.0 Hz).

^{19}F NMR (376 MHz, DMSO-d^6) δ -65.86 (t, *J* = 9.0 Hz).

ESI HRMS [C15H13F3N2O + H$^+$] calculated:295.1058 , found: 295.1051

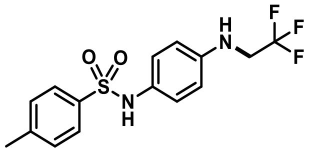

4-methyl-N-(4-((2,2,2-trifluoroethyl)amino)phenyl)benzenesulfonamide

Compound 20: followed the condition A, 0.2 mmol reaction scale, 69% yield, 47.5 mg, white solid. Purified by column chromatography on silica gel eluting with petroleum ether/ethyl acetate (5:1).

^{1}H NMR (400 MHz, DMSO-d^6) δ 9.57 (s, 1H), 7.56 (d, *J* = 8.0 Hz, 2H), 7.30 (d, *J* = 8.0 Hz, 2H), 6.81 (d, *J* = 8.4 Hz, 2H), 6.58 (d, *J* = 8.4 Hz, 2H), 6.12 (t, *J* = 7.0 Hz, 1H), 3.96-3.72 (m, 2H), 2.32 (s, 3H).

^{13}C NMR (100 MHz, DMSO-d^6) δ 145.6, 143.2, 137.4, 129.9, 127.4, 127.2, 126.3 (q, *J* = 281.0 Hz), 124.5, 113.1, 44.7 (q, *J* = 33.3 Hz), 21.4.

^{19}F NMR (376 MHz, DMSO-d^6) δ -70.72 (t, *J* = 9.8 Hz).

ESI HRMS [C15H15F3N2O2S + H$^+$] calculated:345.0885 , found: 345.0883

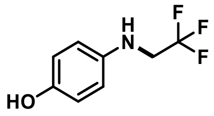

4-((2,2,2-trifluoroethyl)amino)phenol

Compound 21: Condition A, 0.2 mmol reaction scale, 74% yield, 28.2 mg, white solid. Condition C1, 0.2 mmol reaction scale, 56% yield, 21.4 mg, white solid. Purified by column chromatography on silica gel eluting with petroleum ether/ethyl acetate (5:1).

^{1}H NMR (400 MHz, $CDCl_3$) δ 6.73 (d, *J* = 8.5 Hz, 2H), 6.60 (d, *J* = 8.5 Hz, 2H), 5.34-4.42 (br, 1H), 3.99-3.24 (br, 1H), 3.79-3.53 (m, 2H).

^{13}C NMR (100 MHz, $CDCl_3$) δ 148.8, 140.3, 125.1 (q, *J* = 281.0 Hz), 116.3, 77.3, 77.0, 76.7, 47.2 (q, *J* = 33.3 Hz).

^{19}F NMR (376 MHz, $CDCl_3$) δ -72.29 (t, *J* = 9.0 Hz).

ESI HRMS [C8H8F3NO + H$^+$] calculated: 192.0636 , found: 192.0632

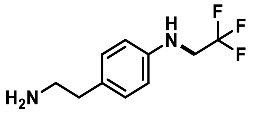

4-(2-aminoethyl)-N-(2,2,2-trifluoroethyl)aniline

Compound 22: followed the condition A, 0.2 mmol reaction scale, 53% yield, 23.1 mg, pale yellow solid. Purified by column chromatography on silica gel eluting with dichloromethane/methanol (20:1-5:1, Several drops of triethylamine were added to the eluent solution).

^{1}H NMR (400 MHz, CD_3OD) δ 7.06 (d, *J* = 8.0 Hz, 2H), 6.71 (d, *J* = 8.0 Hz, 2H),5.14-4.37 (br, 3H), 3.88-3.65 (m, 2H), 3.19-3.00 (m, 2H), 2.92-2.73 (m, 2H).

^{13}C NMR (100 MHz, CD_3OD) δ 146.7, 129.1, 125.6 (q, *J* = 281.0 Hz), 125.3, 112.9, 44.8 (q, *J* = 33.3 Hz), 40.9, 32.3.

^{19}F NMR (376 MHz, CD_3OD) δ -73.88 (t, *J* = 9.5 Hz).

ESI HRMS [C10H13F3N2 + H^+] calculated: 219.1109 , found: 219.1103

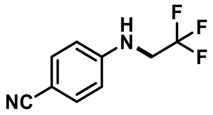

4-((2,2,2-trifluoroethyl)amino)benzonitrile

Compound 23: followed the condition A, 0.2 mmol reaction scale, 35% yield, 14.1 mg, white solid. Purified by column chromatography on silica gel eluting with petroleum ether/ethyl acetate (10:1). Reported compound[12].

^{1}H NMR (400 MHz, $CDCl_3$) δ 7.47 (d, *J* = 8.4 Hz, 2H), 6.69 (d, *J* = 8.4 Hz, 2H), 4.57 (s, 1H), 3.94-3.72 (m, 2H).

^{13}C NMR (100 MHz, $CDCl_3$) δ 149.6, 133.8, 124.5 (q, *J* = 281.0 Hz), 119.7, 112.7, 101.1, 77.3, 77.0, 76.7, 44.9 (q, *J* = 33.3 Hz).

^{19}F NMR (376 MHz, $CDCl_3$) δ -72.11 (t, *J* = 9.0 Hz).

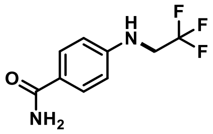

4-((2,2,2-trifluoroethyl)amino)benzamide

Compound 24: followed the condition A, 0.2 mmol reaction scale, 45% yield, 19.6 mg, white solid. Purified by column chromatography on silica gel eluting with petroleum ether/ethyl acetate (1:1, Several drops of triethylamine were added to the eluent solution).

^{1}H NMR (400 MHz, DMSO-d^6) δ 7.68 (d, *J* = 8.4 Hz, 2H), 7.64 (s, 1H), 6.95 (s, 1H), 6.72 (d, *J* = 8.4 Hz, 2H), 6.69 (t, *J* = 6.3 Hz, 2H), 4.16-3.80 (m, 2H).

^{13}C NMR (100 MHz, DMSO-d^6) δ 168.3, 150.6, 129.5, 126.2 (q, *J* = 281.0 Hz), 123.0, 111.8, 44.0 (q, *J* = 33.3 Hz).

^{19}F NMR (376 MHz, DMSO-d^6) δ -70.61 (t, *J* = 9.7 Hz).

ESI HRMS [C9H9F3N2O + H^+] calculated:219.0745 , found: 219.0737

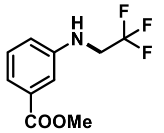

methyl 3-((2,2,2-trifluoroethyl)amino)benzoate

Compound 25: followed the condition A, 0.2 mmol reaction scale, 59% yield, 27.3 mg, white solid. Purified by column chromatography on silica gel eluting with petroleum ether/ethyl acetate (200:1-100:1).

^{1}H NMR (400 MHz, $CDCl_3$) δ 7.49 (d, *J* = 7.9 Hz, 1H), 7.39 (s, 1H), 7.29 (t, *J* = 7.9 Hz, 1H), 6.89 (d, *J* = 7.9 Hz, 1H), 4.19 (s, 1H), 3.92 (s, 3H), 3.89-3.74 (m, 2H).

^{13}C NMR (100 MHz, $CDCl_3$) δ 167.1, 146.3, 131.2, 129.4, 125.1 (q, *J* = 281.0 Hz), 120.1, 117.5, 113.6, 52.1, 45.7 (q, *J* = 33.3 Hz).

^{19}F NMR (376 MHz, $CDCl_3$) δ -72.30 (t, *J* = 9.0 Hz).

ESI HRMS [C10H10F3NO2 + H^+] calculated:234.0742 , found: 233.0733

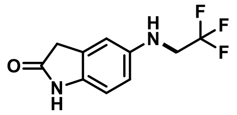

5-((2,2,2-trifluoroethyl)amino)indolin-2-one

Compound 26: followed the condition A, 0.2 mmol reaction scale, 63% yield, 29.0 mg, white solid. Purified by column chromatography on silica gel eluting with dichloromethane/ethyl acetate (10:1).

^{1}H NMR (400 MHz, DMSO-d^6) δ 10.01 (s, 1H), 6.68 (s, 1H), 6.59 (d, *J* = 8.3 Hz, 1H), 6.53 (d, *J* = 8.3 Hz, 1H), 5.82 (t, *J* = 7.2 Hz, 1H), 3.93-3.70 (m, 2H).

^{13}C NMR (100 MHz, DMSO-d^6) δ 176.4, 143.1, 135.1, 127.2, 126.4 (q, *J* = 281.0 Hz), 111.4, 110.7, 109.8, 45.4 (q, *J* = 33.3 Hz), 36.6.

^{19}F NMR (376 MHz, DMSO-d^6) δ -70.57 (t, *J* = 9.8 Hz).

ESI HRMS [C10H9F3N2O + H^+] calculated:231.0745 , found: 231.0748

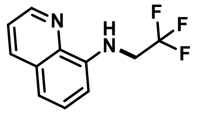

N-(2,2,2-trifluoroethyl)quinolin-8-amine

Compound 27: followed the condition A, 0.2 mmol reaction scale, 40% yield, 18.2 mg, colorless oil. Purified by column chromatography on silica gel eluting with petroleum ether/ethyl acetate (200:1).

^{1}H NMR (400 MHz, DMSO-d^6) δ 8.79 (d, *J* = 4.1 Hz, 1H), 8.26 (d, *J* = 8.3 Hz, 1H), 7.54 (dd, *J* = 8.3, 4.1 Hz, 1H), 7.41 (t, *J* = 7.9 Hz, 1H), 7.20 (d, *J* = 8.1 Hz, 1H), 7.01 (t, *J* = 7.2 Hz, 1H), 6.96 (d, *J* = 7.7 Hz, 1H), 4.28-4.13 (m, 2H).

^{13}C NMR (100 MHz, DMSO-d^6) δ 147.9, 143.8, 137.8, 136.6, 128.7, 127.9, 126.4 (q, *J* = 281.0 Hz), 122.4, 115.5, 105.8, 44.3 (q, *J* = 33.3 Hz).

^{19}F NMR (376 MHz, DMSO-d^6) δ -70.24 (t, *J* = 9.0 Hz).

ESI HRMS [C11H9F3N2 + H^+] calculated: 227.0796 , found: 227.0789

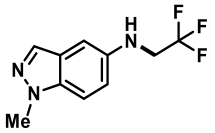

1-methyl-N-(2,2,2-trifluoroethyl)-1H-indazol-5-amine

Compound 28: followed the condition A, 0.2 mmol reaction scale, 55% yield, 25.3 mg, white solid. Purified by column chromatography on silica gel eluting with petroleum ether/ethyl acetate (5:1).

^{1}H NMR (400 MHz, $CDCl_3$) δ 7.84 (s, 1H), 7.27 (d, *J* = 8.9 Hz, 1H), 6.92 (s, 1H), 6.89 (d, *J* = 8.9 Hz, 1H), 4.03 (s, 3H), 3.95 (s, 1H), 3.87-3.74 (m, 2H).

^{13}C NMR (100 MHz, $CDCl_3$) δ 140.5, 135.7, 131.4, 125.1 (q, *J* = 281.0 Hz), 124.6, 117.8, 109.9, 100.8, 47.1 (q, *J* = 33.3 Hz), 35.5.

^{19}F NMR (376 MHz, $CDCl_3$) δ -72.01 (t, *J* = 9.0 Hz).

ESI HRMS [C10H10F3N3 + H^+] calculated:230.0905 , found: 230.0896

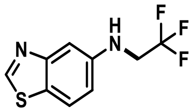

N-(2,2,2-trifluoroethyl)benzo[d]thiazol-5-amine

Compound 29: followed the condition A, 0.2 mmol reaction scale, 43% yield, 20.0 mg, white solid. Purified by column chromatography on silica gel eluting with petroleum ether/ethyl acetate (20:1-10:1).

^{1}H NMR (400 MHz, $CDCl_3$) δ 8.97 (s, 1H), 7.75 (d, *J* = 8.8 Hz, 1H), 7.44 (s, 1H), 6.89 (d, *J* = 8.8 Hz, 1H), 4.22 (s, 1H), 4.01-3.73 (m, 2H).

^{13}C NMR (100 MHz, $CDCl_3$) δ 155.0, 154.9, 145.5, 125.1 (q, *J* = 281.0 Hz), 124.1, 122.2, 114.5, 105.5, 46.2 (q, *J* = 33.3 Hz).

^{19}F NMR (376 MHz, $CDCl_3$) δ -72.10 (t, *J* = 9.0 Hz).

ESI HRMS [C9H7F3N2S + H^+] calculated: 233.0360 , found: 233.0352

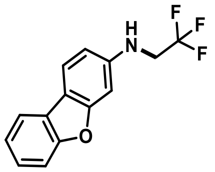

N-(2,2,2-trifluoroethyl)dibenzo[b,d]furan-3-amine

Compound 30: followed the condition A, 0.2 mmol reaction scale, 77% yield, 40.8 mg, white solid. Purified by column chromatography on silica gel eluting with petroleum ether/ethyl acetate (100:1).

^{1}H NMR (400 MHz, $CDCl_3$) δ 7.76-7.68 (m, 1H), 7.63 (d, *J* = 8.4 Hz, 1H), 7.40 (d, *J* = 7.8 Hz, 1H), 7.29-7.16 (m, 2H), 6.76 (d, *J* = 2.1 Hz, 1H), 6.58 (dd, *J* = 8.4, 2.1 Hz, 1H), 4.08 (t, *J* = 6.6 Hz, 1H), 3.83-3.64 (m, 2H).

^{13}C NMR (100 MHz, $CDCl_3$) δ 158.0, 156.0, 146.6, 125.4, 125.1 (q, *J* = 281.0 Hz), 124.6, 122.7, 121.4, 119.5, 116.1, 111.3, 110.0, 95.3, 46.3 (q, *J* = 33.3 Hz).

^{19}F NMR (376 MHz, $CDCl_3$) δ -72.08 (t, *J* = 8.9 Hz).

ESI HRMS [C14H10F3NO + H^+] calculated: 266.0793 , found: 266.0785

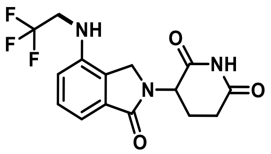

3-(1-oxo-4-((2,2,2-trifluoroethyl)amino)isoindolin-2-yl)piperidine-2,6-dione

Compound 31: followed the condition A, 0.2 mmol reaction scale, 48% yield, 32.8 mg, white solid. Purified by column chromatography on silica gel eluting with petroleum ether/ethyl acetate (1:2, Several drops of triethylamine were added to the eluent solution).

^{1}H NMR (400 MHz, DMSO-d^6) δ 11.03 (s, 1H), 7.34 (t, *J* = 7.8 Hz, 1H), 7.05 (d, *J* = 7.8 Hz, 2H), 7.02 (d, *J* = 7.8 Hz, 2H), 6.27 (t, *J* = 6.7 Hz, 1H), 5.12 (dd, *J* = 13.3, 5.1 Hz, 1H), 4.34-4.13 (m, 2H), 4.12-3.96 (m, 2H), 3.00-2.84 (m, 1H), 2.68-2.58 (m, 1H), 2.40-2.23 (m, 1H), 2.11-2.01 (m, 1H).

^{13}C NMR (100 MHz, DMSO-d^6) δ 173.4, 171.7, 169.0, 142.8, 132.8, 129.7, 127.2, 126.3 (q, *J* = 281.0 Hz), 113.1, 112.2, 52.0, 46.2, 44.1 (q, *J* = 33.3 Hz), 31.7, 23.2.

^{19}F NMR (376 MHz, DMSO-d^6) δ -70.46 (t, *J* = 9.6 Hz).

ESI HRMS [C15H14F3N3O3 + H^+] calculated:342.1066 , found: 342.1058

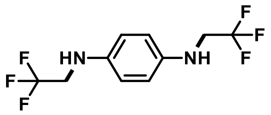

N^1,N^4-bis(2,2,2-trifluoroethyl)benzene-1,4-diamine

Compound 32: followed the condition A, 0.1 mmol reaction scale, 47% yield, 12.7 mg, white solid. Purified by column chromatography on silica gel eluting with petroleum ether/ethyl acetate (15:1).

^{1}H NMR (400 MHz, $CDCl_3$) δ 6.63 (s, 4H), 3.88-3.38 (br, 2H), 3.79-3.61 (m, 4H).

^{13}C NMR (100 MHz, $CDCl_3$) δ 139.6, 125.1 (q, *J* = 281.0 Hz), 115.1, 47.2 (q, *J* = 33.3 Hz).

^{19}F NMR (376 MHz, $CDCl_3$) δ -72.33 (t, *J* = 9.0 Hz).

ESI HRMS [C10H10F6N2 + H^+] calculated:273.0826 , found: 273.0821

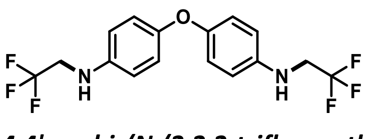

4,4'-oxybis(N-(2,2,2-trifluoroethyl)aniline)

Compound 33: followed the condition A, 0.1 mmol reaction scale, 53% yield, 19.3 mg, colorless oil. Purified by column chromatography on silica gel eluting with petroleum ether/ethyl acetate (15:1).

^{1}H NMR (400 MHz, $CDCl_3$) δ 6.87 (d, *J* = 8.6 Hz, 2H), 6.65 (d, *J* = 8.6 Hz, 2H), 4.02-3.60 (br, 2H), 3.78-3.66 (m, 4H).

^{13}C NMR (100 MHz, $CDCl_3$) δ 150.8, 142.0, 125.1 (q, *J* = 281.0 Hz), 119.7, 114.4, 46.8 (q, *J* = 33.3 Hz).

^{19}F NMR (376 MHz, $CDCl_3$) δ -72.31 (t, *J* = 8.8 Hz).

ESI HRMS [C16H14F6N2O + H^+] calculated:365.1089 , found: 365.1084

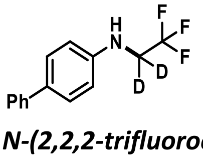

N-(2,2,2-trifluoroethyl-1,1-d^2)-[1,1'-biphenyl]-4-amine

Compound 3-d: followed the condition A using CF_3COOD instead of CF_3COOH, 0.2 mmol reaction scale, 43% yield, 85% D, 21.7 mg, white solid. Purified by column chromatography on silica gel eluting with petroleum ether/ethyl acetate (200:1).

^{1}H NMR (600 MHz, DMSO-d^6) δ 7.56 (d, *J* = 7.4 Hz, 2H), 7.45 (d, *J* = 8.5 Hz, 2H), 7.39 (t, *J* = 7.7 Hz, 2H), 7.24 (t, *J* = 7.3 Hz, 1H), 6.82 (d, *J* = 8.6 Hz, 2H), 6.36 (s, 1H).

^{13}C NMR (101 MHz, DMSO-d^6) δ 147.6, 140.8, 129.3, 129.2, 127.7, 126.5, 126.4 (q, *J* = 281.0 Hz), 126.0, 113.2.

^{19}F NMR (377 MHz, DMSO-d^6) δ -70.70.

ESI HRMS [C14H10D2F3N + H^+] calculated: 254.1126 , found: 254.1118.

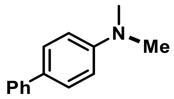

N,N-dimethyl-[1,1'-biphenyl]-4-amine

Compound 34: followed the condition B2, 0.2 mmol reaction scale, 71% yield, 28.1 mg, white solid, with HCOOH. Followed the condition B1, 0.15 mmol reaction scale, 58% yield, 17.1 mg, with HCOONa. Purified by column chromatography on silica gel eluting with petroleum ether/ethyl acetate (200:1). Reported compound[16].

^{1}H NMR (400 MHz, $CDCl_3$) δ 7.68-7.62 (m, 2H), 7.62-7.56 (m, 2H), 7.52-7.43 (m, 2H), 7.37 -7.30 (m, 1H), 6.95-6.80 (m, 2H), 3.06 (s, 6H).

^{13}C NMR (100 MHz, $CDCl_3$) δ 150.0, 141.3, 129.4, 128.7, 127.8, 126.4, 126.1, 112.9, 40.7.

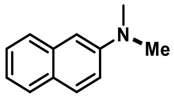

N,N-dimethylnaphthalen-2-amine

Compound 35: followed the condition B1, 0.15 mmol reaction scale, 46% yield, 11.9 mg, white solid. Purified by column chromatography on silica gel eluting with petroleum ether/ethyl acetate (200:1). Reported compound[17].

^{1}H NMR (400 MHz, $CDCl_3$) δ 7.89-7.67 (m, 3H), 7.52-7.38 (m, 1H), 7.31-7.24 (m, 1H), 7.24-7.16 (m, 1H), 6.99 (d, *J* = 2.5 Hz, 1H), 3.09 (s, 6H).

^{13}C NMR (100 MHz, $CDCl_3$) δ 148.6, 135.0, 128.7, 127.5, 126.9, 126.2, 122.2, 116.5, 106.6, 41.0.

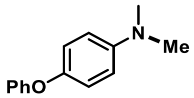

N,N-dimethyl-4-phenoxyaniline

Compound 36: followed the condition B1, 0.15 mmol reaction scale, 47% yield, 14.9 mg, colorless oil. Purified by column chromatography on silica gel eluting with petroleum ether/dichloromethane (7:1). Reported compound[17].

^{1}H NMR (400 MHz, $CDCl_3$) δ 7.36-7.29 (m, 2H), 7.08-7.03 (m, 1H), 7.03-7.00 (m, 2H), 7.00-6.96 (m, 2H), 6.84-6.74 (m, 2H), 2.98 (s, 6H).

^{13}C NMR (100 MHz, $CDCl_3$) δ 159.1, 147.7, 147.3, 129.5, 122.0, 121.0, 117.2, 114.0, 41.3.

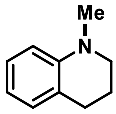

1-methyl-1,2,3,4-tetrahydroquinoline

Compound 37: followed the condition B1, 0.15 mmol reaction scale, 52% yield, 11.4 mg, colorless oil. Purified by column chromatography on silica gel eluting with petroleum ether. Reported compound[18].

^{1}H NMR (400 MHz, $CDCl_3$) δ 7.18-7.06 (m, 1H), 7.06 -6.92 (m, 1H), 7.02-6.95 (m, 2H), 3.35-3.18 (m, 2H), 2.92 (s, 3H), 2.80 (t, J = 6.5 Hz, 2H), 2.12-1.96 (m, 2H).

^{13}C NMR (100 MHz, $CDCl_3$) δ 146.6, 128.9, 127.1, 123.0, 116.5, 111.2, 51.3, 39.3, 27.8, 22.4.

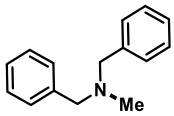

N-benzyl-N-methyl-1-phenylmethanamine

Compound 38: followed the condition B1, 0.15 mmol reaction scale, 45% yield, 14.2 mg, white solid. Purified by column chromatography on silica gel eluting with dichloromethane/methanol (100:1). Reported compound[19].

^{1}H NMR (400 MHz, $CDCl_3$) δ 7.54-7.47 (m, 4H), 7.47-7.40 (m, 4H), 7.40-7.33 (m, 2H), 3.65 (s, 4H), 2.31 (s, 3H).

^{13}C NMR (100 MHz, $CDCl_3$) δ 139.2, 129.0, 128.3, 127.0, 61.9, 42.2.

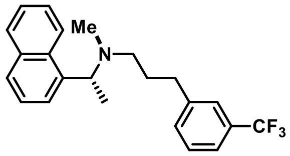

(R)-N-methyl-N-(1-(naphthalen-1-yl)ethyl)-3-(3-(trifluoromethyl)phenyl)propan-1-amine

Compound 39: followed the condition B1, 0.15 mmol reaction scale, 36% yield, 20.0 mg, colorless oil. Purified by column chromatography on silica gel eluting with dichloromethane/ethyl acetate (10:1). Reported compound[20].

^{1}H NMR (400 MHz, $CDCl_3$) δ 8.42 (d, J = 8.1 Hz, 1H), 7.94-7.83 (m, 1H), 7.77 (d, J = 8.1 Hz, 1H), 7.57 (d, J = 7.0 Hz, 1H), 7.54-7.47 (m, 2H), 7.45 (d, J = 7.6 Hz, 1H), 7.43-7.38 (m, 1H), 7.37-7.33 (m, 1H), 7.30 (t, J = 7.7 Hz, 1H), 7.17 (d, J = 7.6 Hz, 1H), 4.32 (q, J = 6.6 Hz, 1H), 2.61-2.41 (m, 4H), 2.32 (s, 3H), 1.78 (p, J = 7.4 Hz, 2H), 1.48 (d, J = 6.7 Hz, 3H).

^{13}C NMR (100 MHz, $CDCl_3$) δ 143.5, 140.6, 134.1, 131.9, 131.7, 130.5 (q, J = 128 Hz), 128.7, 128.6, 127.4, 125.5, 125.4, 125.3, 125.0 (q, J = 16 Hz), 124.5, 124.4, 124.3 (q, J = 1084 Hz), 122.5 (q, J = 16 Hz), 60.5, 53.6, 38.6, 33.2, 29.0, 16.8.

^{19}F NMR (376 MHz, $CDCl_3$) δ -62.5.

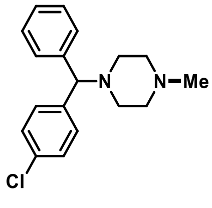

1-((4-chlorophenyl)(phenyl)methyl)-4-methylpiperazine

Compound 40: followed the condition B1, 0.15 mmol reaction scale, 54% yield, 24.3 mg, colorless oil. Purified by column chromatography on silica gel eluting with dichloromethane/methanol (30:1). Reported compound[18].

^{1}H NMR (400 MHz, $CDCl_3$) δ 7.42-7.35 (m, 4H), 7.33-7.24 (m, 4H), 7.23 -7.17 (m, 1H), 4.22 (s, 1H), 2.71-2.35 (m, 8H), 2.32 (s, 3H).

^{13}C NMR (100 MHz, $CDCl_3$) δ 142.2, 141.4, 132.6, 129.2, 128.7, 128.6, 127.8, 127.2, 75.5, 55.3, 51.7, 45.8.

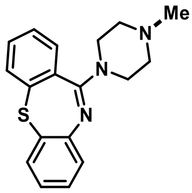

11-(4-methylpiperazin-1-yl)dibenzo[b,f][1,4]thiazepine

Compound 41: followed the condition B1, 0.15 mmol reaction scale, 63% yield, 29.4 mg, white solid. Purified by column chromatography on silica gel eluting with dichloromethane/methanol (40:1). Reported compound[21].

^{1}H NMR (400 MHz, $CDCl_3$) δ 7.60-7.49 (m, 1H), 7.41 (dd, J = 7.7, 1.4 Hz, 1H), 7.39-7.29 (m, 3H), 7.19 (td, J = 7.6, 1.5 Hz, 1H), 7.10 (dd, J = 8.0, 1.4 Hz, 1H), 6.90 (td, J = 7.5, 1.5 Hz, 1H), 3.59 (s, 4H), 2.69-2.43 (m, 4H), 2.37 (s, 3H).

^{13}C NMR (100 MHz, $CDCl_3$) δ 160.9, 149.0, 140.0, 134.2, 132.3, 132.3, 130.9, 129.2, 129.1, 128.4, 128.1, 125.4, 123.0, 77.5, 77.2, 76.8, 54.9, 46.2.

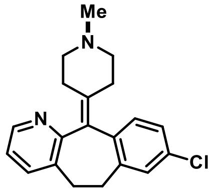

8-chloro-11-(1-methylpiperidin-4-ylidene)-6,11-dihydro-5H-benzo[5,6]cyclohepta[1,2-b]pyridine

Compound 42: followed the condition B1, 0.15 mmol reaction scale, 44% yield, 21.5 mg, white solid. Purified by column chromatography on silica gel eluting with dichloromethane/methanol (15:1). Reported compound[22].

^{1}H NMR (400 MHz, $CDCl_3$) δ 8.42 (dd, J = 4.8, 1.5 Hz, 1H), 7.45 (dd, J = 7.7, 1.5 Hz, 1H), 7.17 (s, 1H), 7.16-7.13 (m, 2H), 7.11 (dd, J = 7.7, 4.8 Hz, 1H), 3.51-3.31 (m, 2H), 2.98-2.70 (m, 4H), 2.65 -2.48 (m, 2H), 2.48-2.38 (m, 2H), 2.34 (s, 3H), 2.28-2.14 (m, 2H).

^{13}C NMR (100 MHz, $CDCl_3$) δ 157.4, 146.7, 139.6, 137.8, 137.7, 137.4, 133.4, 133.2, 132.8, 130.7, 129.0, 126.1, 122.2, 56.8, 45.8, 31.8, 31.5, 30.7, 30.5.

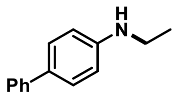

N-ethyl-[1,1'-biphenyl]-4-amine

Compound 43: followed the condition B2, 0.2 mmol reaction scale, 47% yield, 18.5 mg, white solid.

Purified by column chromatography on silica gel eluting with petroleum ether/ethyl acetate (50:1). Reported compound[23].

^{1}H NMR (400 MHz, $CDCl_3$) δ 7.62 -7.55 (m, 2H), 7.53-7.46 (m, 2H), 7.46-7.39 (m, 2H), 7.33-7.25 (m, 1H), 6.74 (d, *J* = 8.6 Hz, 2H), 4.2-3.1 (br, 1H), 3.24 (q, *J* = 7.1 Hz, 2H), 1.32 (t, *J* = 7.1 Hz, 3H).

^{13}C NMR (100 MHz, $CDCl_3$) δ 147.6, 141.3, 130.4, 128.7, 128.0, 126.3, 126.1, 113.2, 38.7, 14.9.

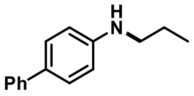

N-propyl-[1,1'-biphenyl]-4-amine

Compound 44: followed the condition B2, 0.2 mmol reaction scale, 44% yield, 18.7 mg, white solid. Purified by column chromatography on silica gel eluting with petroleum ether/ethyl acetate (200:1). Reported compound[24].

^{1}H NMR (400 MHz, $CDCl_3$) δ 7.60 (d, *J* = 7.6 Hz, 2H), 7.50 (d, *J* = 8.1 Hz, 2H), 7.44 (t, *J* = 7.6 Hz, 2H), 7.30 (t, *J* = 7.6 Hz, 1H), 6.73 (d, *J* = 8.1 Hz, 2H), 4.14-3.32 (br, 1H), 3.18 (t, *J* = 7.4 Hz, 2H), 1.72 (h, *J* = 7.4 Hz, 2H), 1.07 (t, *J* = 7.4 Hz, 3H).

^{13}C NMR (100 MHz, $CDCl_3$) δ 147.9, 141.4, 130.1, 128.7, 128.0, 126.3, 126.0, 113.0, 45.9, 22.8, 11.7.

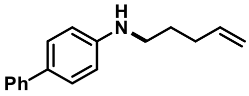

N-(pent-4-en-1-yl)-[1,1'-biphenyl]-4-amine

Compound 45: followed the condition B2, 0.2 mmol reaction scale, 41% yield, 19.6 mg, white solid. Purified by column chromatography on silica gel eluting with petroleum ether/ethyl acetate (200:1).

^{1}H NMR (400 MHz, $CDCl_3$) δ 7.59-7.53 (m, 2H), 7.49-7.44 (m, 2H), 7.40 (t, *J* = 7.7 Hz, 2H), 7.32 -7.24 (m, 1H), 6.71 (d, *J* = 8.6 Hz, 2H), 5.94-5.79 (m, 1H), 5.15-5.06 (m, 1H), 5.03 (d, *J* = 10.2 Hz, 1H), 3.20 (t, *J* = 7.1 Hz, 2H), 2.21 (q, *J* = 7.1 Hz, 2H), 1.77 (p, *J* = 7.1 Hz, 2H).

^{13}C NMR (100 MHz, $CDCl_3$) δ 147.6, 141.3, 138.0, 130.3, 128.7, 128.0, 126.3, 126.1, 115.2, 113.2, 43.6, 31.3, 28.6.

ESI HRMS [C17H19N + H$^+$] calculated: 238.1596 , found:238.1592

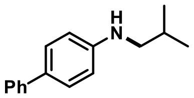

N-isobutyl-[1,1'-biphenyl]-4-amine

Compound 46: 0.2 mmol reaction scale, 25% yield, 11.3 mg, white solid. Purified by column chromatography on silica gel eluting with petroleum ether/ethyl acetate (300:1).

^{1}H NMR (400 MHz, $CDCl_3$) δ 7.58 (d, J = 7.6 Hz, 2H), 7.48 (d, J = 8.5 Hz, 2H), 7.43 (t, J = 7.6 Hz, 2H), 7.33-7.27 (m, 1H), 6.72 (d, J = 8.5 Hz, 2H), 4.18-3.38 (br, 1H), 3.02 (d, J = 6.8 Hz, 2H), 2.04-1.88 (m, 1H), 1.05 (d, J = 6.7 Hz, 6H).

^{13}C NMR (100 MHz, $CDCl_3$) δ 148.0, 141.4, 129.9, 128.7, 128.0, 126.3, 126.0, 112.9, 51.9, 28.1, 20.5.

ESI HRMS [C16H19N + H$^+$] calculated: 226.1596 , found: 226.1591

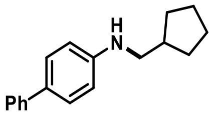

N-(cyclopentylmethyl)-[1,1'-biphenyl]-4-amine

Compound 47: followed the condition B2, 0.2 mmol reaction scale, 40% yield, 20.1 mg, white solid. Purified by column chromatography on silica gel eluting with petroleum ether/ethyl acetate (200:1).

^{1}H NMR (400 MHz, $CDCl_3$) δ 7.57 (d, *J* = 7.3 Hz, 2H), 7.47 (d, *J* = 8.5 Hz, 2H), 7.42 (t, *J* = 7.7 Hz, 2H), 7.30-7.24 (m, 1H), 6.74 (d, *J* = 8.5 Hz, 2H), 3.11 (d, *J* = 7.2 Hz, 2H), 2.28-2.16 (dt, *J* = 15.1, 7.6 Hz, 1H), 1.97-1.82 (m, 2H), 1.71-1.60 (m, 4H), 1.37-1.25 (m, 2H).

^{13}C NMR (100 MHz, $CDCl_3$) δ 147.6, 141.3, 130.4, 128.7, 127.9, 126.3, 126.0, 113.3, 49.8, 39.4, 30.7, 25.3.

ESI HRMS [C18H21N + H$^+$] calculated: 252.1752 , found:252.1744

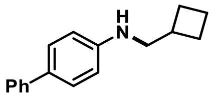

N-(cyclobutylmethyl)-[1,1'-biphenyl]-4-amine

Compound 48: followed the condition B2, 0.2 mmol reaction scale, 52% yield, 24.7 mg, white solid. Purified by column chromatography on silica gel eluting with petroleum ether/ethyl acetate (200:1).

^{1}H NMR (400 MHz, $CDCl_3$) δ 7.59 (d, *J* = 7.6 Hz, 2H), 7.49 (d, *J* = 8.4 Hz, 2H), 7.44 (t, *J* = 7.7 Hz, 2H), 7.36-7.24 (m, 1H), 6.74 (d, *J* = 8.4 Hz, 2H), 3.22 (d, *J* = 7.3 Hz, 2H), 2.74-2.58 (m, 1H), 2.25-2.13 (m, 2H), 2.07-1.91 (m, 2H), 1.88-1.74 (m, 2H).

^{13}C NMR (100 MHz, $CDCl_3$) δ 147.8, 141.3, 130.3, 128.7, 127.9, 126.3, 126.1, 113.2, 50.0, 35.0, 26.1, 18.6.

ESI HRMS [C17H19N + H$^+$] calculated:238.1596 , found:238.1597

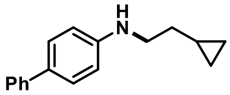

N-(2-cyclopropylethyl)-[1,1'-biphenyl]-4-amine

Compound 49: 0.2 mmol reaction scale, 20% yield, 9.5 mg, white solid. Purified by column chromatography on silica gel eluting with petroleum ether/ethyl acetate (300:1).

^{1}H NMR (400 MHz, $CDCl_3$) δ 7.60-7.54 (m, 2H), 7.51-7.45 (m, 2H), 7.45-7.38 (m, 2H), 7.31-7.25 (m, 2H), 6.80-6.73 (m, 2H), 4.82-3.77 (br, 1H), 3.29 (t, J = 7.0 Hz, 2H), 1.59 (q, J = 7.0 Hz, 2H), 0.85-0.74 (m, 1H), 0.57-0.48 (m, 2H), 0.19-0.11 (m, 2H).

^{13}C NMR (100 MHz, $CDCl_3$) δ 147.4, 141.2, 130.6, 128.7, 128.0, 126.3, 126.1, 113.5, 44.6, 34.3, 8.8, 4.3.

ESI HRMS [C17H19N + H$^+$] calculated: 238.1596 , found: 238.1592

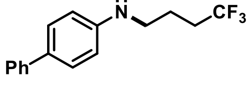

N-(4,4,4-trifluorobutyl)-[1,1'-biphenyl]-4-amine

Compound 50: followed the condition B2, 0.2 mmol reaction scale, 32% yield, 17.9 mg, white solid. Purified by column chromatography on silica gel eluting with petroleum ether/ethyl acetate (200:1).

^{1}H NMR (400 MHz, $CDCl_3$) δ 7.61-7.53 (m, 2H), 7.53-7.47 (m, 2H), 7.42 (t, *J* = 7.7 Hz, 2H), 7.31 (d, *J* = 7.4 Hz, 1H), 6.77 (d, *J* = 8.6 Hz, 2H), 3.30 (t, *J* = 7.0 Hz, 2H), 2.40-2.19 (m, 2H), 1.96 (dt, *J* = 14.5, 7.1 Hz, 2H), 1.88-1.41 (m, 1H).

^{13}C NMR (100 MHz, $CDCl_3$) δ 146.4, 141.0, 131.5, 128.7, 128.1, 127.2 (q, *J* = 275 Hz), 126.4, 126.3, 113.7, 43.3, 31.4 (q, *J* = 29 Hz), 22.0.

^{19}F NMR (376 MHz, $CDCl_3$) δ -66.06 (t, *J* = 10.8 Hz).

ESI HRMS [C16H16F3N + H^+] calculated: 280.1313 , found: 280.1308

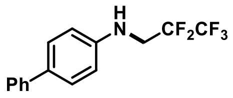

N-(2,2,3,3,3-pentafluoropropyl)-[1,1'-biphenyl]-4-amine

Compound 51: followed the condition A, 0.2 mmol reaction scale, 35% yield, 21.2 mg, white solid. Purified by column chromatography on silica gel eluting with petroleum ether/ethyl acetate (200:1). Reported compound[12].

^{1}H NMR (400 MHz, $CDCl_3$) δ 7.59 (d, *J* = 7.6 Hz, 2H), 7.52 (d, *J* = 8.1 Hz, 2H), 7.46 (t, *J* = 7.6 Hz, 2H), 7.34 (t, *J* = 7.6 Hz, 1H), 6.80 (d, *J* = 8.1 Hz, 2H), 4.21-3.55 (br, 1H), 3.89 (t, *J* = 14.8 Hz, 2H).

^{13}C NMR (100 MHz, $CDCl_3$) δ 145.8, 140.9, 132.2, 128.8, 128.1, 126.5, 126.5, 113.5, 44.2(t, *J* = 24.0 Hz). The nuclear magnetic resonance signal of carbon in the -CF_2 and -CF_3 groups is notably weak.

^{19}F NMR (376 MHz, $CDCl_3$) δ -83.90, -121.83 (t, *J* = 14.7 Hz).

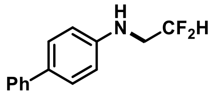

N-(2,2-difluoroethyl)-[1,1'-biphenyl]-4-amine

Compound 52: 0.2 mmol reaction scale, 25% yield, 11.7 mg, white solid. Purified by column chromatography on silica gel eluting with petroleum ether/ethyl acetate (200:1)[12].

^{1}H NMR (400 MHz, $CDCl_3$) δ 7.60-7.54 (m, 2H), 7.54-7.47 (m, 2H), 7.47-7.38 (m, 2H), 7.31 (tt, J = 6.8, 1.2 Hz, 1H), 6.85-6.67 (m, 2H), 5.98 (tt, J = 56.1, 4.2 Hz, 1H), 4.88-2.30 (br, 1H), 3.61 (td, J = 14.4, 4.2 Hz, 2H).

^{13}C NMR (100 MHz, $CDCl_3$) δ 146.2, 140.9, 131.7, 128.7, 128.2, 126.4, 114.5 (t, 240 Hz), 113.4, 46.5 (t, 26 Hz).

^{19}F NMR (376 MHz, $CDCl_3$) δ -122.6 (dt, *J* = 56.4, 14.4 Hz).

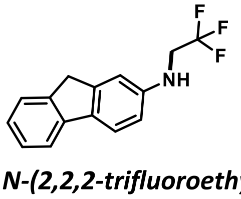

N-(2,2,2-trifluoroethyl)-9H-fluoren-2-amine

Compound 53: followed the condition C1, 0.2 mmol reaction scale, 65% yield, 34.0 mg, white solid.

Purified by column chromatography on silica gel eluting with petroleum ether/ethyl acetate (100:1). Reported compound[13].

^{1}H NMR (400 MHz, $CDCl_3$) δ 7.66 (d, *J* = 7.5 Hz, 1H), 7.61 (d, *J* = 8.2 Hz, 1H), 7.49 (d, *J* = 7.5 Hz, 1H), 7.34 (t, *J* = 7.5 Hz, 1H), 7.22 (t, *J* = 7.5 Hz, 1H), 6.88 (s, 1H), 6.71 (d, *J* = 8.2 Hz, 1H), 4.00 (s, 1H), 3.83 (s, 2H), 3.89-3.75 (m, 2H).

^{13}C NMR (100 MHz, $CDCl_3$) δ 145.7, 145.3, 142.3, 141.9, 133.6, 126.7, 125.3, 125.1 (q, *J* = 281.0 Hz), 124.8, 120.7, 118.8, 112.4, 109.8, 46.3 (q, *J* = 33.3 Hz), 37.0.

^{19}F NMR (376 MHz, $CDCl_3$) δ -72.19 (t, *J* = 9.0 Hz).

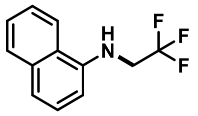

***N*-(2,2,2-trifluoroethyl)naphthalen-1-amine**

Compound 54: followed the condition C1, 0.2 mmol reaction scale, 44% yield, 19.8 mg, white solid. Purified by column chromatography on silica gel eluting with petroleum ether/ethyl acetate (200:1). Reported compound[16].

^{1}H NMR (400 MHz, $CDCl_3$) δ 7.93-7.78 (m, 2H), 7.57-7.47 (m, 2H), 7.45-7.35 (m, 2H), 6.81-6.71 (m, 1H), 4.95-4.24 (br, 1H), 4.06-3.90 (m, 2H).

^{13}C NMR (100 MHz, $CDCl_3$) δ 141.5, 134.3, 128.8, 126.2, 126.1, 125.3, 125.1 (q, *J* = 281.0 Hz), 123.6, 119.7, 119.5, 105.5, 46.3 (q, *J* = 33.3 Hz).

^{19}F NMR (376 MHz, $CDCl_3$) δ -71.63 (t, *J* = 8.9 Hz).

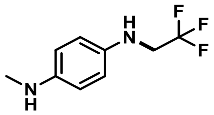

N^1-methyl-N^4-(2,2,2-trifluoroethyl)benzene-1,4-diamine

Compound 55: followed the condition C1, 0.2 mmol reaction scale, 44% yield, 18.1 mg, white solid. Purified by column chromatography on silica gel eluting with petroleum ether/ethyl acetate (7:1).

^{1}H NMR (400 MHz, $CDCl_3$) δ 6.76-6.51 (m, 4H), 3.83-3.65 (m, 2H), 3.65-3.36 (br, 1H), 3.11- 2.91 (br, 1H), 2.82 (s, 3H).

^{13}C NMR (100 MHz, $CDCl_3$) δ 142.8, 138.4, 125.3 (q, *J* = 281.0 Hz), 115.4, 114.2, 47.6 (q, *J* = 33.3 Hz), 31.8.

^{19}F NMR (376 MHz, $CDCl_3$) δ -72.29 (t, *J* = 9.0 Hz).

ESI HRMS [C9H11F3N2 + H^+] calculated: 205.0953 , found:205.0944

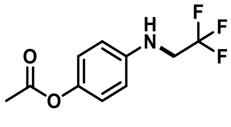

4-((2,2,2-trifluoroethyl)amino)phenyl acetate

Compound 56: followed the condition C1, 0.2 mmol reaction scale, 63% yield, 29.5 mg, white solid. Purified by column chromatography on silica gel eluting with petroleum ether/ethyl acetate (10:1).

^{1}H NMR (400 MHz, $CDCl_3$) δ 6.93 (d, *J* = 8.9 Hz, 2H), 6.65 (d, *J* = 8.9 Hz, 2H), 4.23-3.82 (br, 1H), 3.79-3.66 (m, 2H), 2.26 (s, 3H).

^{13}C NMR (100 MHz, $CDCl_3$) δ 170.1, 144.1, 143.1, 125.1 (q, *J* = 281.0 Hz), 122.3, 113.6, 46.1 (q, *J* =

33.3 Hz), 21.0.

^{19}F NMR (376 MHz, $CDCl_3$) δ -72.32 (t, *J* = 8.9 Hz).

ESI HRMS [C10H10F3NO2 + H^+] calculated:234.0742 , found:234.0737

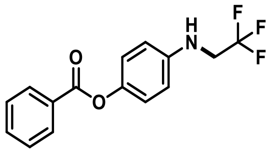

4-((2,2,2-trifluoroethyl)amino)phenyl benzoate

Compound 57: followed the condition C1, 0.2 mmol reaction scale, 41% yield, 24.3 mg, white solid. Purified by column chromatography on silica gel eluting with petroleum ether/ethyl acetate (30:1).

^{1}H NMR (400 MHz, $CDCl_3$) δ 8.22(d, *J* = 7.4 Hz, 2H), 7.65 (t, *J* = 7.4 Hz, 1H), 7.53 (t, *J* = 7.7 Hz, 2H), 7.09 (d, *J* = 8.8 Hz, 2H), 6.74 (d, *J* = 8.8 Hz, 2H), 4.23-3.54 (br, 1H), 3.87-3.67 (m, 2H).

^{13}C NMR (100 MHz, $CDCl_3$) δ 165.7, 144.2, 143.4, 133.5, 130.1, 129.7, 128.6, 125.1 (q, *J* = 281.0 Hz), 122.5, 113.7, 46.4 (q, *J* = 33.3 Hz).

^{19}F NMR (376 MHz, $CDCl_3$) δ -72.27 (t, *J* = 8.9 Hz).

ESI HRMS [C15H12F3NO2 + H^+] calculated: 296.0898 , found:296.0890

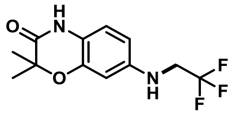

2,2-dimethyl-7-((2,2,2-trifluoroethyl)amino)-2H-benzo[b][1,4]oxazin-3(4H)-one

Compound 58: followed the condition C1, 0.2 mmol reaction scale, 69% yield, 37.9 mg, white solid. Purified by column chromatography on silica gel eluting with dichloromethane/ethyl acetate (10:1-8:1).

^{1}H NMR (400 MHz, DMSO-d^6) δ 10.24 (s, 1H), 6.64 (d, *J* = 8.4 Hz, 1H), 6.42-6.24 (m, 2H), 6.03 (t, *J* = 7.0 Hz, 1H), 3.94-3.71 (m, 2H), 1.35 (s, 6H).

^{13}C NMR (100 MHz, DMSO-d^6) δ 168.2, 144.6, 143.2, 126.3 (q, *J* = 281.0 Hz), 118.7, 116.1, 106.7, 101.8, 77.7, 44.9 (q, *J* = 33.3 Hz), 23.8.

^{19}F NMR (376 MHz, DMSO-d^6) δ -70.63 (t, *J* = 9.7 Hz).

ESI HRMS [C12H13F3N2O2 + H^+] calculated: 275.1007 , found:275.1000

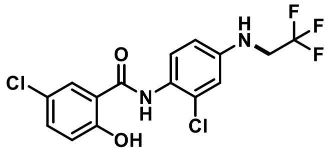

5-chloro-N-(2-chloro-4-((2,2,2-trifluoroethyl)amino)phenyl)-2-hydroxybenzamide

Compound 59: followed the condition C1, 0.2 mmol reaction scale, 38% yield, 28.6 mg, white solid. Purified by column chromatography on silica gel eluting with petroleum ether/ethyl acetate (5:1-3:1).

^{1}H NMR (400 MHz, DMSO-d^6) δ 12.24 (s, 1H), 10.48 (s, 1H), 8.05-7.96 (m, 1H), 7.81 (d, *J* = 8.8 Hz, 1H), 7.53-7.43 (m, 1H), 7.03 (d, *J* = 8.8 Hz, 1H), 6.98-6.86 (m, 1H), 6.84-6.70 (m, 1H), 6.52 (t, *J* = 6.6 Hz, 1H), 4.06-3.88 (m, 2H).

^{13}C NMR (100 MHz, DMSO-d^6) δ 164.1, 156.8, 146.5, 133.7, 129.5, 127.1, 126.3, 126.1 (q, *J* = 281.0

Hz), 124.6, 123.6, 119.6, 119.4, 112.7, 112.1, 44.3 (q, *J* = 33.3 Hz).
^{19}F NMR (376 MHz, DMSO-d^6) δ -70.61 (t, *J* = 9.6 Hz).
ESI HRMS [C15H11Cl2F3N2O2 + H$^+$] calculated:379.0228 , found:379.0229

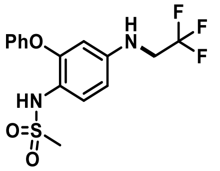

N-(2-phenoxy-4-((2,2,2-trifluoroethyl)amino)phenyl)methanesulfonamide

Compound 60: followed the condition C1, 0.2 mmol reaction scale, 38% yield, 27.7 mg, white solid. Purified by column chromatography on silica gel eluting with petroleum ether/dichloromethane (1:1-1:10).

^{1}H NMR (400 MHz, DMSO-d^6) δ 8.88 (s, 1H), 7.40 (t, *J* = 7.8 Hz, 2H), 7.21-7.08 (m, 2H), 7.04 (d, *J* = 8.0 Hz, 2H), 6.50 (d, *J* = 9.4 Hz, 1H), 6.41 (t, *J* = 7.0 Hz, 1H), 6.26 (s, 1H), 3.92-3.76 (m, 2H), 2.87 (s, 3H).
^{13}C NMR (100 MHz, DMSO-d^6) δ 156.7, 153.1, 148.1, 130.7, 130.3, 126.1 (q, *J* = 281.0 Hz), 123.9, 119.3, 117.2, 107.5, 103.3, 44.4 (q, *J* = 33.3 Hz).
^{19}F NMR (376 MHz, DMSO-d^6) δ -70.67 (t, *J* = 9.7 Hz).
ESI HRMS [C15H15F3N2O3S + H$^+$] calculated: 361.0834 , found:361.0830

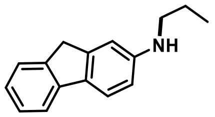

N-propyl-9H-fluoren-2-amine

Compound 61: followed the condition C2, 0.2 mmol reaction scale, 35% yield, 15.6 mg, white solid. Purified by column chromatography on silica gel eluting with petroleum ether/ethyl acetate (200:1).

^{1}H NMR (400 MHz, CDCl$_3$) δ 7.65 (d, *J* = 7.6 Hz, 1H), 7.60 (d, J = 8.2 Hz, 1H), 7.48 (d, *J* = 7.4 Hz, 1H), 7.33 (t, *J* = 7.5 Hz, 1H), 7.20 (t, *J* = 7.4 Hz, 1H), 6.86 (s, 1H), 6.69 (dd, *J* = 8.2, 2.1 Hz, 1H), 3.84 (s, 2H), 3.18 (t, *J* = 7.1 Hz, 2H), 1.78-1.63 (m, 2H), 1.06 (t, *J* = 7.4 Hz, 3H).
^{13}C NMR (100 MHz, CDCl$_3$) δ 147.7, 145.2, 142.4, 142.2, 132.0, 126.6, 124.8, 124.7, 120.6, 118.4, 112.2, 109.3, 46.4, 37.0, 22.7, 11.7.
ESI HRMS [C16H17N + H$^+$] calculated: 224.1439 , found:224.1445

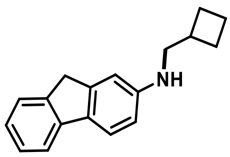

N-(cyclobutylmethyl)-9H-fluoren-2-amine

Compound 62: followed the condition C2, 0.2 mmol reaction scale, 47% yield, 23.3 mg, white solid. Purified by column chromatography on silica gel eluting with petroleum ether/ethyl acetate (200:1).

^{1}H NMR (400 MHz, CDCl$_3$) δ 7.64 (d, *J* = 7.6 Hz, 1H), 7.60 (d, *J* = 8.2 Hz, 1H), 7.48 (d, *J* = 7.4 Hz, 1H),

7.33 (t, J = 7.5 Hz, 1H), 7.20 (t, J = 7.4 Hz, 1H), 6.85 (s, 1H), 6.68 (d, J = 8.2 Hz, 1H), 3.84 (s, 2H), 3.23 (d, J = 7.3 Hz, 2H), 2.73-2.58 (m, 1H), 2.24-2.10 (m, 2H), 2.05-1.89 (m, 2H), 1.87-1.74 (m, 2H).

^{13}C NMR (100 MHz, $CDCl_3$) δ 148.0, 145.2, 142.4, 142.2, 131.9, 126.6, 124.8, 124.7, 120.6, 118.4, 112.1, 109.2, 50.2, 37.0, 35.0, 26.1, 18.6.

ESI HRMS [C18H19N + H^+] calculated: 250.1596 , found:250.1600

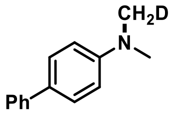

N-methyl-N-(methyl-d)-[1,1'-biphenyl]-4-amine

Compound 63: followed the condition D1, 0.15 mmol reaction scale, 47% yield, 99% D, 14.1 mg, white solid. Purified by column chromatography on silica gel eluting with petroleum ether/ethyl acetate (200:1).

^{1}H NMR (400 MHz, $CDCl_3$) δ 7.63-7.58 (m, 2H), 7.58-7.52 (m, 2H), 7.43 (t, J = 7.7 Hz, 2H), 7.36-7.25 (m, 1H), 6.86 (d, J = 8.7 Hz, 2H), 3.03 (s, 3H), 3.03-2.98 (m, 2H).

^{13}C NMR (100 MHz, $CDCl_3$) δ 149.9, 141.2, 129.5, 128.7, 127.8, 126.3, 126.0, 112.9, 40.4 (t, J = 21 Hz).

ESI HRMS [C14H14DN + H^+] calculated:199.1346 , found:199.1339

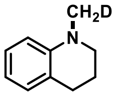

1-(methyl-d)-1,2,3,4-tetrahydroquinoline

Compound 64: followed the condition D1, 0.15 mmol reaction scale, 58% yield, 99% D, 12.9 mg, colorless oil. Purified by column chromatography on silica gel eluting with petroleum ether.

^{1}H NMR (400 MHz, $CDCl_3$) δ 7.11 (t, J = 7.6 Hz, 1H), 6.99 (d, J = 7.6 Hz, 1H), 6.71-6.51 (m, 2H), 3.34-3.18 (m, 2H), 2.95-2.86 (m, 2H), 2.80 (*t*, J = 6.4 Hz, 2H), 2.10-1.94 (m, 2H).

^{13}C NMR (100 MHz, $CDCl_3$) δ 146.7, 128.9, 127.1, 123.0, 116.3, 111.1, 51.3, 38.9 (t, J = 20 Hz), 27.8, 22.4.

ESI HRMS [C10H12DN + H^+] calculated: 149.1189 , found:149.1183

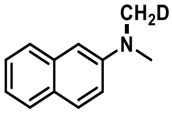

N-methyl-N-(methyl-d)naphthalen-2-amine

Compound 65: followed the condition D1, 0.15 mmol reaction scale, 38% yield, 99% D, 9.9 mg, white solid. Purified by column chromatography on silica gel eluting with petroleum ether/ethyl acetate (200:1).

^{1}H NMR (400 MHz, $CDCl_3$) δ 7.34-7.28 (m, 2H), 7.04 (t, J = 7.4 Hz, 1H), 7.02-6.98 (m, 2H), 6.98 -6.94 (m, 2H), 6.83-6.75 (m, 2H), 2.96 (s, 3H), 2.96-2.91 (m, 2H).

^{13}C NMR (100 MHz, $CDCl_3$) δ 148.5, 134.9, 128.7, 127.5, 127.0, 126.2, 122.2, 116.5, 106.8, 40.8 (t, J = 20 Hz).

ESI HRMS [C12H12DN + H^+] calculated: 173.1189 , found:173.1183

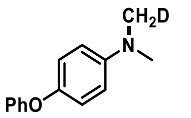

***N*-methyl-*N*-(methyl-*d*)-4-phenoxyaniline**

Compound 66: followed the condition D1, 0.15 mmol reaction scale, 43% yield, 99% D, 13.9 mg, colorless oil. Purified by column chromatography on silica gel eluting with petroleum ether/dichloromethane (7:1).

^{1}H NMR (400 MHz, $CDCl_3$) δ 7.34-7.28 (m, 2H), 7.07-6.94 (m, 5H), 6.83-6.75 (m, 2H), 2.96 (s, 3H), 2.96-2.91 (m, 2H).

^{13}C NMR (100 MHz, $CDCl_3$) δ 159.0, 147.7, 147.3, 129.5, 122.0, 120.9, 117.2, 114.3, 41.4.

ESI HRMS [C14H14DNO + H^+] calculated:215.1295 , found:215.1294

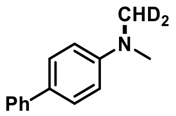

***N*-methyl-*N*-(methyl-d_2)-[1,1'-biphenyl]-4-amine**

Compound 67: followed the condition D2, 0.15 mmol reaction scale, 40% yield, 83% D, 11.9 mg, white solid. Purified by column chromatography on silica gel eluting with petroleum ether/ethyl acetate (200:1).

^{1}H NMR (400 MHz, $CDCl_3$) δ 7.65-7.58 (m, 2H), 7.58-7.52 (m, 2H), 7.43 (t, *J* = 7.7 Hz, 2H), 7.34 -7.25 (m, 1H), 6.86 (d, *J* = 8.8 Hz, 2H), 3.03 (s, 3H), 3.02-2.98 (m, 1H).

^{13}C NMR (100 MHz, $CDCl_3$) δ 149.9, 141.2, 129.5, 128.7, 127.8, 126.3, 126.0, 112.9, 40.7(m).

ESI HRMS [C14H13D2N + H^+] calculated: 200.1408 , found:200.1403

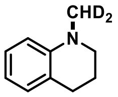

1-(methyl-d_2)-1,2,3,4-tetrahydroquinoline

Compound 68: followed the condition D2, 0.15 mmol reaction scale, 50% yield, 80% D, 12.5 mg, colorless oil. Purified by column chromatography on silica gel eluting with petroleum ether.

^{1}H NMR (400 MHz, $CDCl_3$) δ 7.11 (s, 1H), 6.99 (s, 1H), 3.33-3.20 (m, 2H), 2.91-2.86 (m, 1H), 2.80 (t, *J* = 6.5 Hz, 2H), 2.12-1.95 (m, 2H).

^{13}C NMR (100 MHz, $CDCl_3$) δ 146.6, 128.7, 126.8, 123.0, 115.9, 111.1, 51.3, 38.8(m), 27.8, 22.4.

ESI HRMS [C10H11D2N + H^+] calculated: 150.1252 , found:150.1248

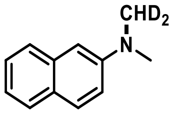

***N*-methyl-*N*-(methyl-d_2)naphthalen-2-amine**

Compound 69: followed the condition D2, 0.15 mmol reaction scale, 35% yield, 84% D, 9.2 mg, white solid. Purified by column chromatography on silica gel eluting with petroleum ether/ethyl acetate (200:1).

^{1}H NMR (400 MHz, $CDCl_3$) δ 7.79-7.71 (m, 2H), 7.70 (d, *J* = 8.3 Hz, 1H), 7.44-7.36 (m, 1H), 7.28-7.17 (m, 2H), 6.98 (d, *J* = 2.2 Hz, 1H), 3.08 (s, 3H), 3.07-3.03 (m, 1H).

^{13}C NMR (100 MHz, $CDCl_3$) δ 148.6, 135.0, 128.7, 127.5, 126.9, 126.2, 126.1, 122.1, 116.5, 106.6, 40.9(m).

ESI HRMS [C12H11D2N + H$^+$] calculated: 174.1252 , found:174.1247

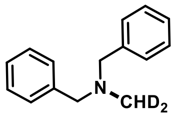

N,N-dibenzylmethanamine-d_2

Compound 70: followed the condition D2, 0.15 mmol reaction scale, 45% yield, 79% D, 14.3 mg, white solid. Purified by column chromatography on silica gel eluting with dichloromethane/methanol (100:1). Reported compound[25].

^{1}H NMR (400 MHz, $CDCl_3$) δ 7.29 (d, J = 7.2 Hz, 4H), 7.24 (t, J = 7.4 Hz, 4H), 7.16 (t, J = 7.1 Hz, 2H), 3.45 (s, 4H), 2.16-2.02 (m, 1H).

^{13}C NMR (100 MHz, $CDCl_3$) δ 139.3, 129.0, 128.3, 127.0, 61.8, 42.0(m).

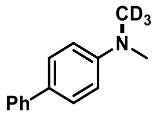

N-methyl-N-(methyl-d_3)-[1,1'-biphenyl]-4-amine

Compound 71: followed the condition D3, 0.15 mmol reaction scale, 39% yield, 84% D, 11.7 mg, white solid. Purified by column chromatography on silica gel eluting with petroleum ether/ethyl acetate (200:1).

^{1}H NMR (400 MHz, $CDCl_3$) δ 7.66-7.51 (m, 4H), 7.51-7.38 (m, 2H), 7.33-7.25 (m, 1H), 6.85 (d, J = 8.9 Hz, 2H), 3.03 (s, 3H).

^{13}C NMR (100 MHz, $CDCl_3$) δ 149.8, 141.2, 129.7, 128.7, 127.8, 126.3, 126.1, 113.0, 40.7.

ESI HRMS [C14H12D3N + H$^+$] calculated: 201.1471 , found:201.1466

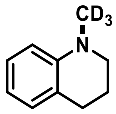

1-(methyl-d_3)-1,2,3,4-tetrahydroquinoline

Compound 72: followed the condition D3, 0.15 mmol reaction scale, 56% yield, 86% D, 12.5 mg, colorless oil. Purified by column chromatography on silica gel eluting with petroleum ether.

^{1}H NMR (400 MHz, $CDCl_3$) δ 7.11 (s, 1H), 6.99 (s, 1H), 3.37-3.20 (m, 2H), 2.93-2.85 (m, 1H), 2.80 (t, J = 6.5 Hz, 2H), 2.13-1.95 (m, 2H).

^{13}C NMR (100 MHz, $CDCl_3$) δ 146.6, 128.8, 126.9, 123.0, 115.9, 111.1, 51.2, 38.8, 27.7, 22.4.

ESI HRMS [C10H10D3N + H$^+$] calculated: 151.1315 , found:151.1310

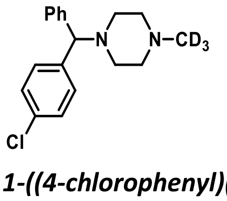

1-((4-chlorophenyl)(phenyl)methyl)-4-(methyl-d_3)piperazine

Compound 73: followed the condition D3, 0.15 mmol reaction scale, 53% yield, 85% D, 24.0 mg,

colorless oil. Purified by column chromatography on silica gel eluting with dichloromethane/methanol (30:1). Reported compound[26].

^{1}H NMR (400 MHz, $CDCl_3$) δ 7.32-7.24 (m, 4H), 7.23-7.13 (m, 4H), 7.13-7.07 (m, 1H), 4.12 (s, 1H), 2.77-2.03 (m, 8H).

^{13}C NMR (100 MHz, $CDCl_3$) δ 142.2, 141.4, 132.5, 129.2, 128.7, 128.6, 127.8, 127.2, 75.5, 55.2, 51.8, 45.0.

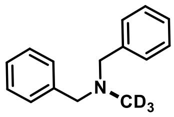

N,N-dibenzylmethanamine-d₃

Compound 74: followed the condition D3, 0.15 mmol reaction scale, 43% yield, 85% D, 13.8 mg, white solid. Purified by column chromatography on silica gel eluting with dichloromethane/methanol (100:1). Reported compound[27].

^{1}H NMR (400 MHz, $CDCl_3$) δ 7.44-7.38 (m, 4H), 7.38-7.32 (m, 4H), 7.31-7.25 (m, 2H), 3.56 (s, 4H).

^{13}C NMR (100 MHz, $CDCl_3$) δ 139.3, 129.0, 128.2, 127.0, 61.8, 41.6.

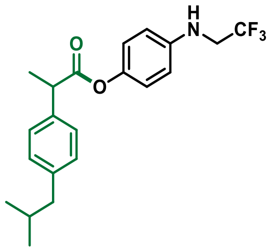

4-((2,2,2-trifluoroethyl)amino)phenyl 2-(4-isobutylphenyl)propanoate

Compound A1: 85% yield, 64.6 mg, white solid. Purified by column chromatography on silica gel eluting with petroleum ether/ethyl acetate (20:1).

^{1}H NMR (400 MHz, $CDCl_3$) δ 7.30 (d, J = 7.7 Hz, 2H), 7.15 (d, J = 7.7 Hz, 2H), 6.85 (d, J = 8.4 Hz, 2H), 6.61 (d, J = 8.5 Hz, 2H), 3.92 (q, J = 7.2 Hz, 1H), 3.78-3.61 (m, 2H), 2.49 (d, J = 7.2 Hz, 2H), 1.98-1.79 (m, 1H), 1.60 (d, J = 7.1 Hz, 3H), 0.93 (d, J = 6.6 Hz, 6H).

^{13}C NMR (101 MHz, $CDCl_3$) δ 173.8, 144.0, 143.4, 140.8, 137.4, 129.5, 127.2, 124.9(q, 279.0 Hz), 122.2, 113.6, 46.2(q, 34.2 Hz), 45.2, 45.1, 30.2, 22.4, 18.6.

^{19}F NMR (376 MHz, $CDCl_3$) δ -72.30 (t, J = 9.0 Hz).

ESI HRMS [C21H24F3NO2 + H^+] calculated: 380.1837, found: 380.1842

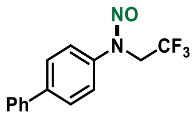

N-([1,1'-biphenyl]-4-yl)-N-(2,2,2-trifluoroethyl)nitrous amide

Compound A2: 88% yield, 49.5 mg, pale yellow solid. Purified by column chromatography on silica gel eluting with petroleum ether/ethyl acetate (200:1).

^{1}H NMR (400 MHz, $CDCl_3$) δ 7.74 (d, J = 8.4 Hz, 2H), 7.67-7.59 (m, 4H), 7.49 (t, J = 7.5 Hz, 2H), 7.42 (d, J = 7.3 Hz, 1H), 4.81-4.60 (m, 2H).

^{13}C NMR (101 MHz, $CDCl_3$) δ 141.2, 140.0, 139.6, 129.0, 128.4, 127.9, 127.1, 125.2(q, 283.0 Hz),

120.6, 43.3(q, 35.2 Hz).
^{19}F NMR (376 MHz, $CDCl_3$) δ -67.06 (t, J = 8.5 Hz).
ESI HRMS [C14H11F3N2O + H^+] calculated: 281.0902, found: 281.0906

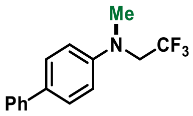

N-methyl-N-(2,2,2-trifluoroethyl)-[1,1'-biphenyl]-4-amine

Compound A3: 58% yield, 30.8 mg, white solid. Purified by column chromatography on silica gel eluting with petroleum ether.
^{1}H NMR (400 MHz, $CDCl_3$) δ 7.70-7.53 (m, 4H), 7.45 (t, J = 7.7 Hz, 2H), 7.37-7.23 (m, 1H), 6.91 (d, J = 8.3 Hz, 2H), 4.07-3.76 (m, 2H), 3.14 (s, 3H).
^{13}C NMR (101 MHz, $CDCl_3$) δ 148.0, 140.8, 131.1, 128.7, 127.9, 126.5, 126.4, 125.2(q, 281.0 Hz), 113.0, 54.3(q, 33.3 Hz), 39.3.
^{19}F NMR (376 MHz, $CDCl_3$) δ -70.38 (t, J = 8.9 Hz).
ESI HRMS [C15H14F3N + H^+] calculated: 266.1157, found: 266.1150

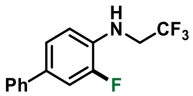

3-fluoro-N-(2,2,2-trifluoroethyl)-[1,1'-biphenyl]-4-amine

Compound A4: 43% yield, 23.2 mg, white solid. Purified by column chromatography on silica gel eluting with petroleum ether/ethyl acetate (20:1).
^{1}H NMR (400 MHz, $CDCl_3$) δ 7.55 (d, J = 7.7 Hz, 2H), 7.44 (t, J = 7.6 Hz, 2H), 7.38-7.27 (m, 3H), 6.88 (t, J = 8.6 Hz, 1H), 4.61-4.07 (br, 1H), 4.02-3.68 (m, 2H).
^{13}C NMR (101 MHz, $CDCl_3$) δ 151.8(d, 237.5 Hz), 139.9(d, 2.0 Hz), 134.0(d, 11.4 Hz), 132.2(d, 6.6 Hz), 128.8, 126.9, 126.5, 124.8(q, 278.4 Hz), 123.0(d, 3.1 Hz), 113.6(d, 19.4 Hz), 112.6, 45.7(d, 33.9 Hz).
^{19}F NMR (376 MHz, $CDCl_3$) δ -72.35 (t, J = 8.9 Hz), -135.76 (m).
ESI HRMS [C14H11F4N + H^+] calculated: 270.0906 , found: 270.089

3.5 NMR Spectra of products

^1H NMR of Compound 3 (400 MHz, $CDCl_3$)

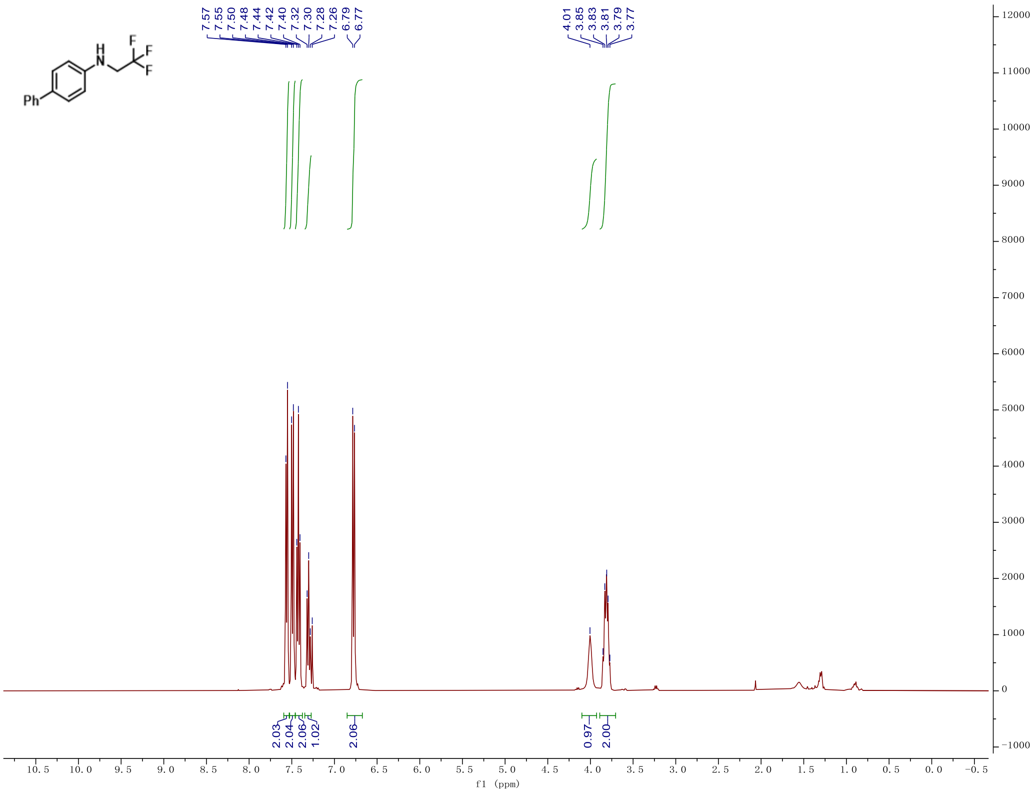

^{13}C NMR of Compound 3 (101 MHz, $CDCl_3$)

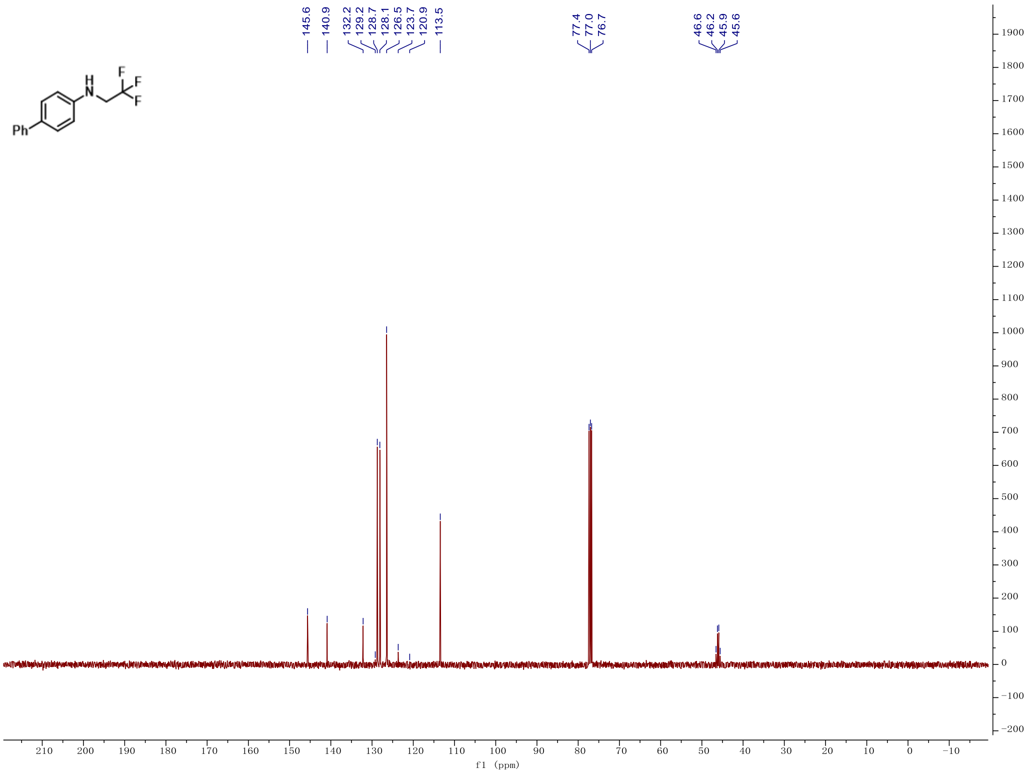

^{19}F NMR of Compound 3 (376 MHz, $CDCl_3$)

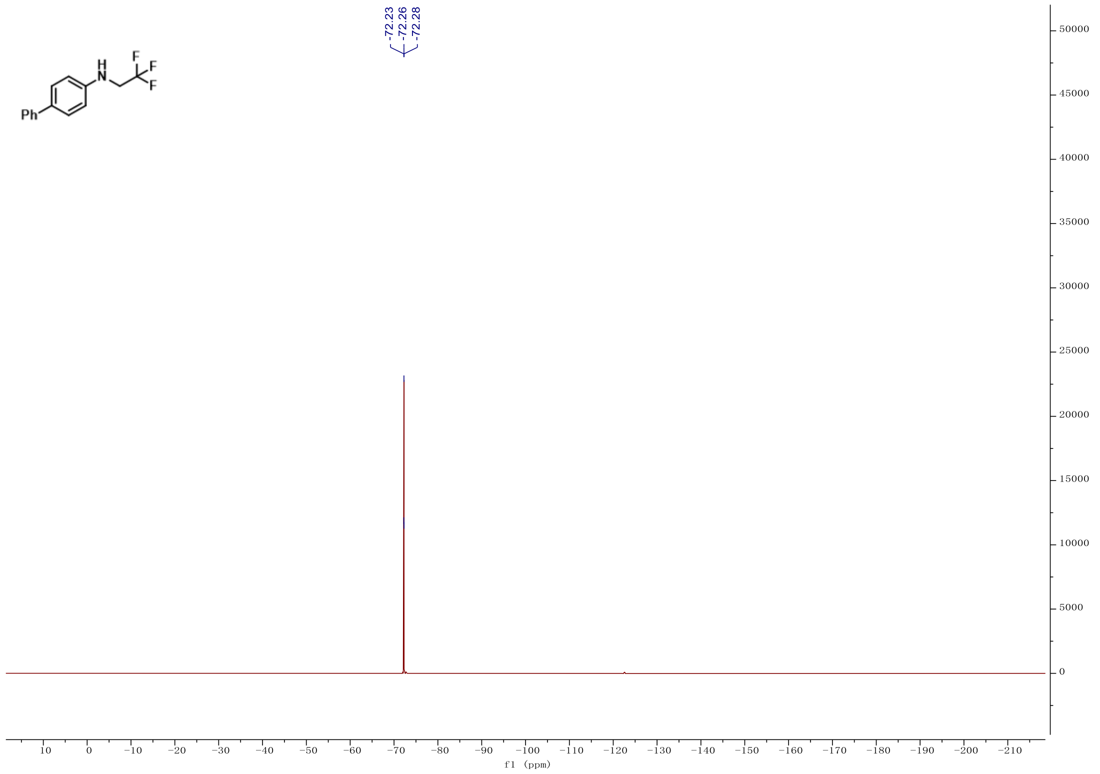

^{1}H NMR of Compound 4 (400 MHz, $CDCl_3$)

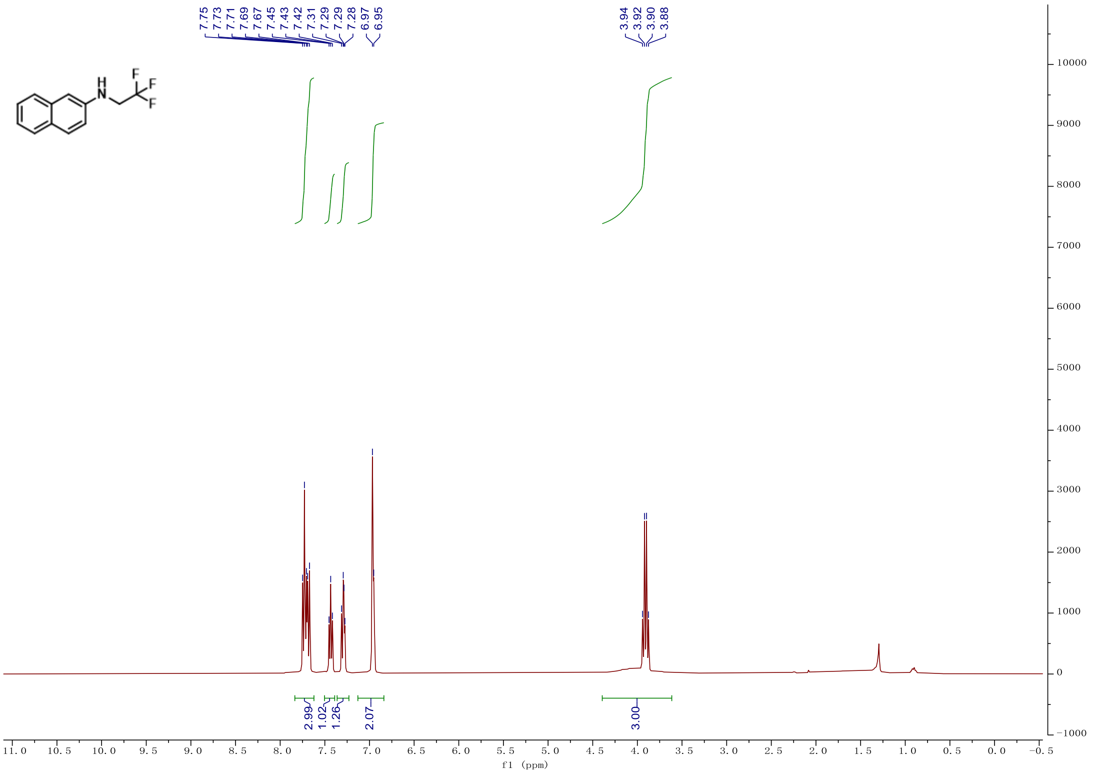

^{13}C NMR of Compound 4 (101 MHz, $CDCl_3$)

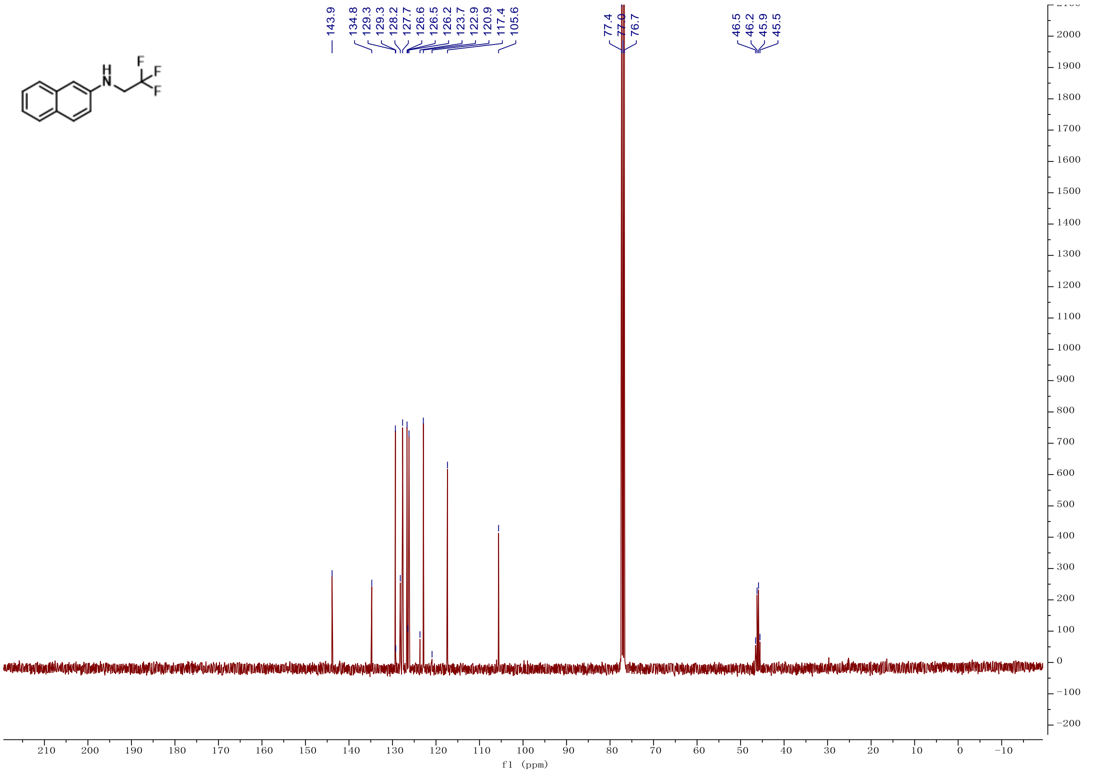

^{19}F NMR of Compound 4 (376 MHz, CDCl$_3$)

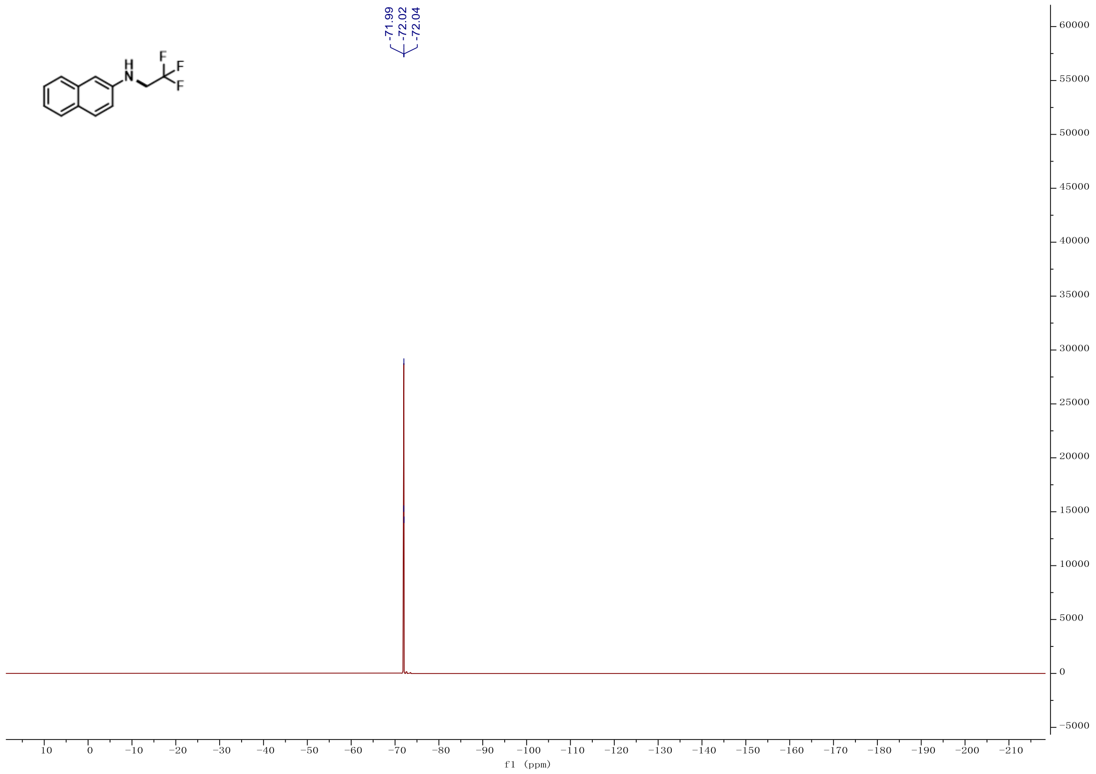

^1H NMR of Compound 5 (400 MHz, $CDCl_3$)

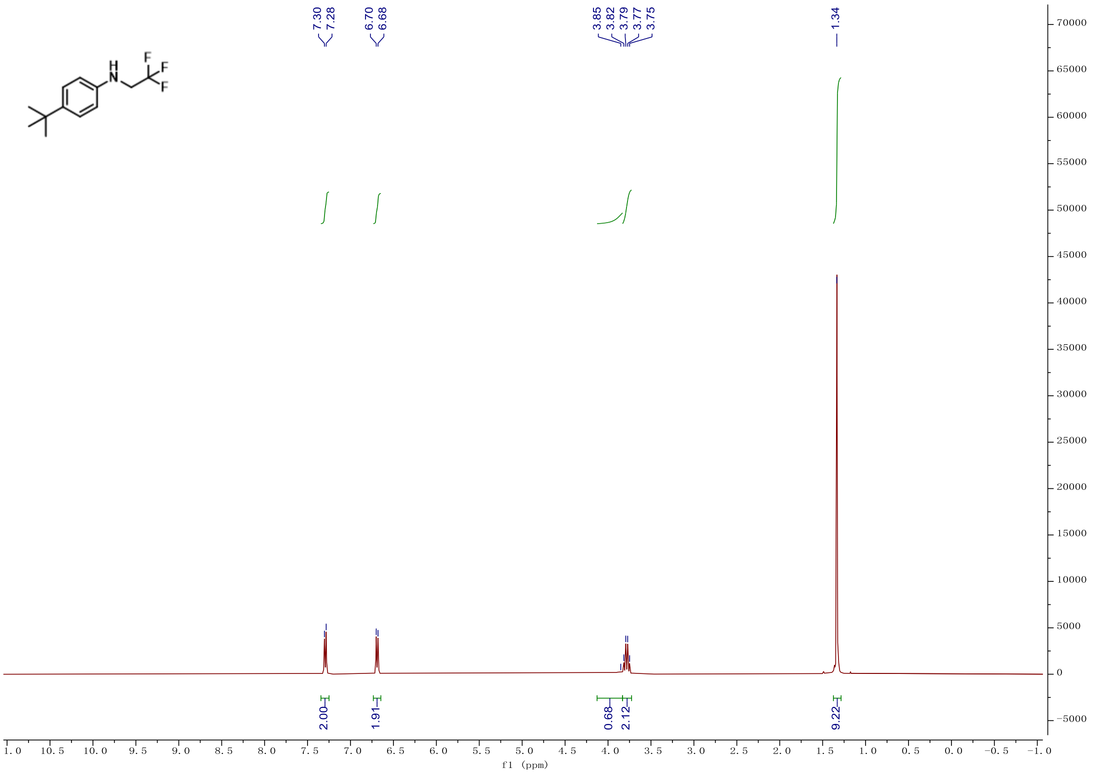

^{13}C NMR of Compound 5 (101 MHz, $CDCl_3$)

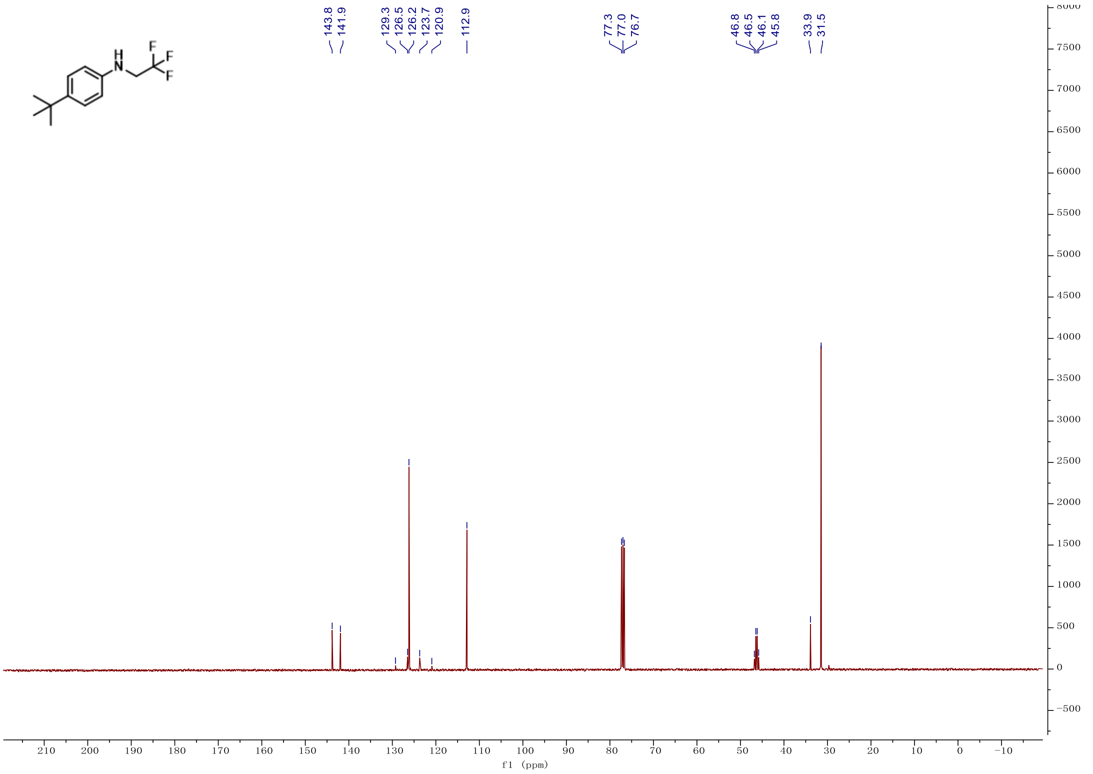

^{19}F NMR of Compound 5 (376 MHz, $CDCl_3$)

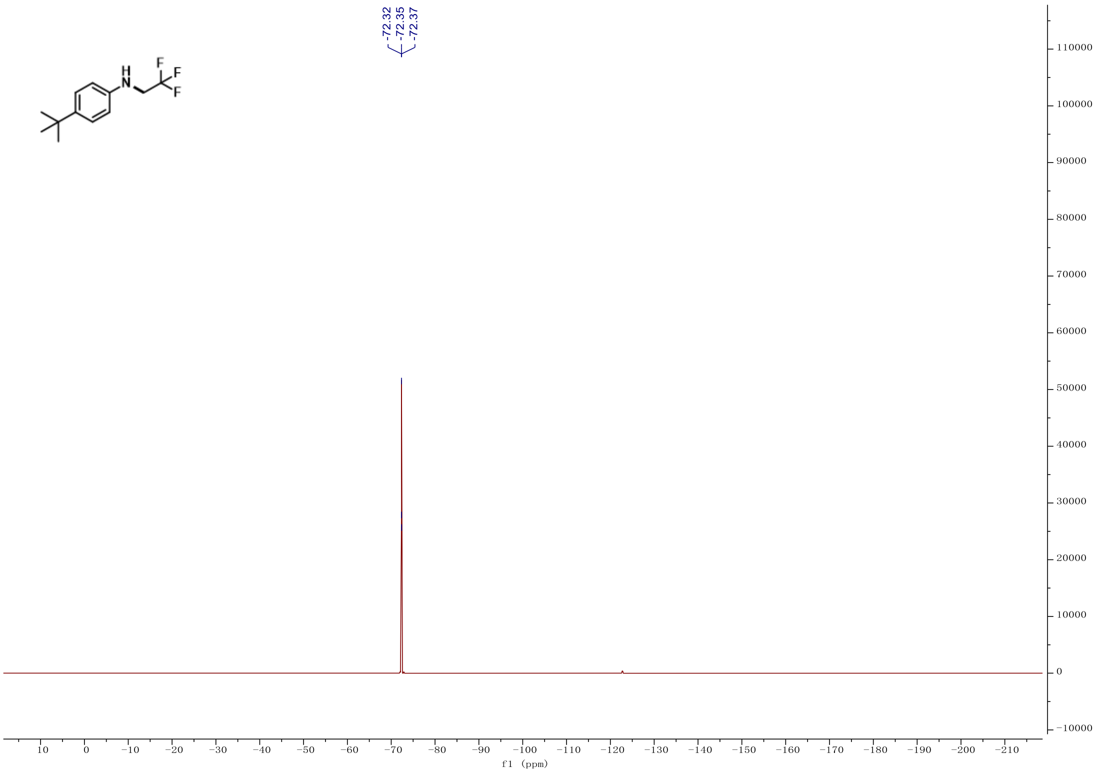

^{1}H NMR of Compound 6 (400 MHz, $CDCl_3$)

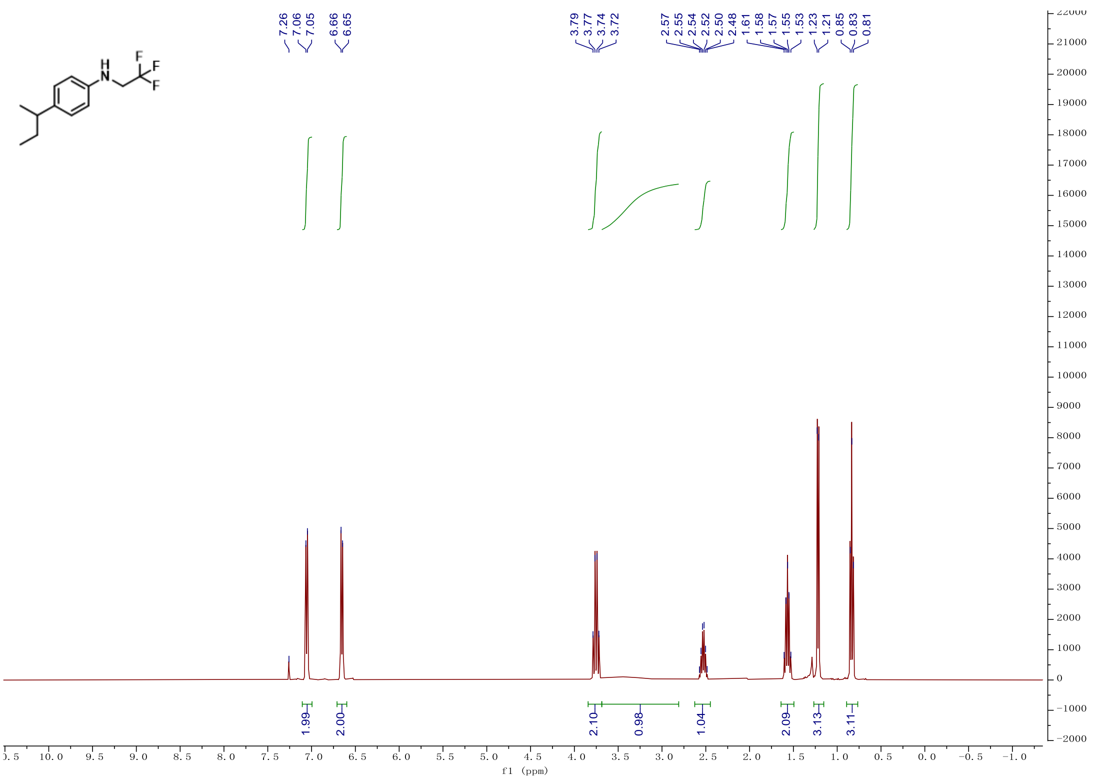

^{13}C NMR of Compound 6 (101 MHz, $CDCl_3$)

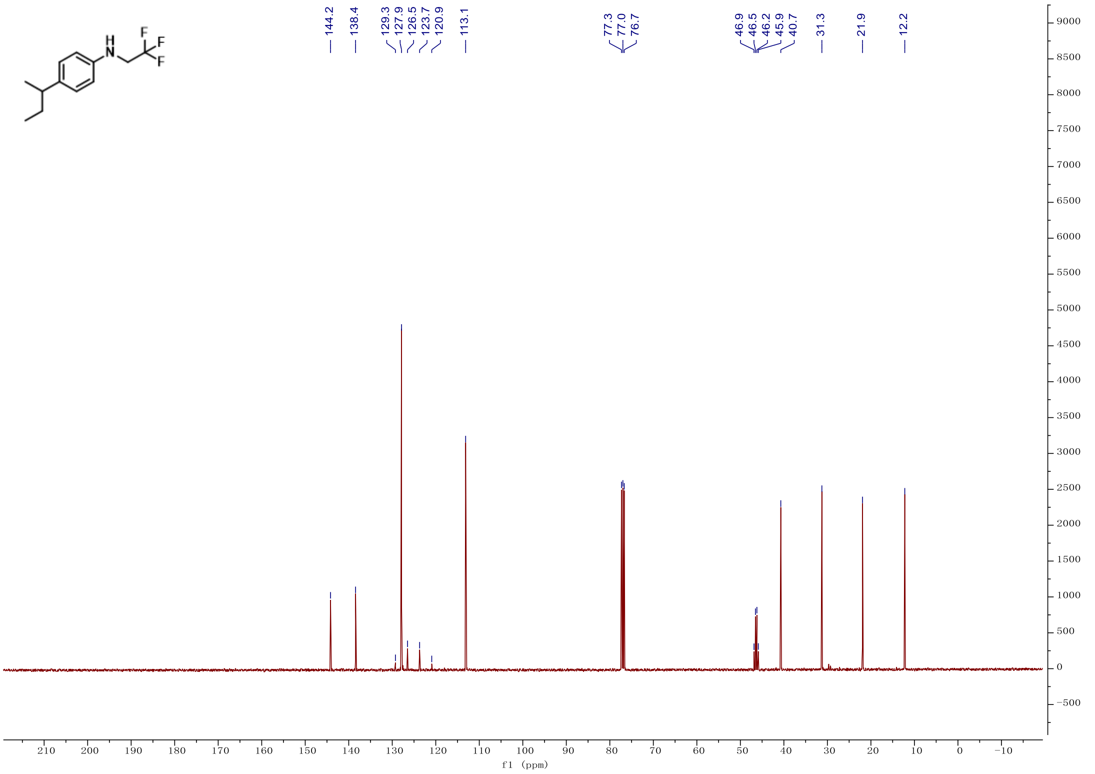

^{19}F NMR of Compound 6 (376 MHz, $CDCl_3$)

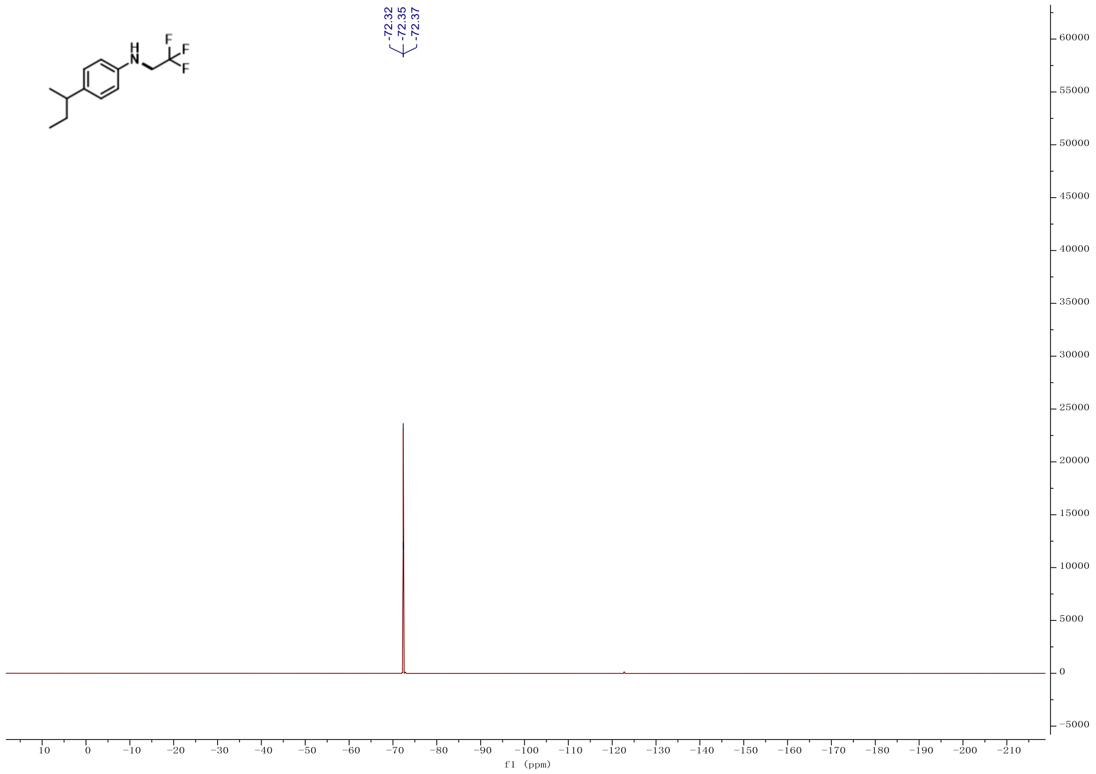

^{1}H NMR of Compound 7 (400 MHz, $CDCl_3$)

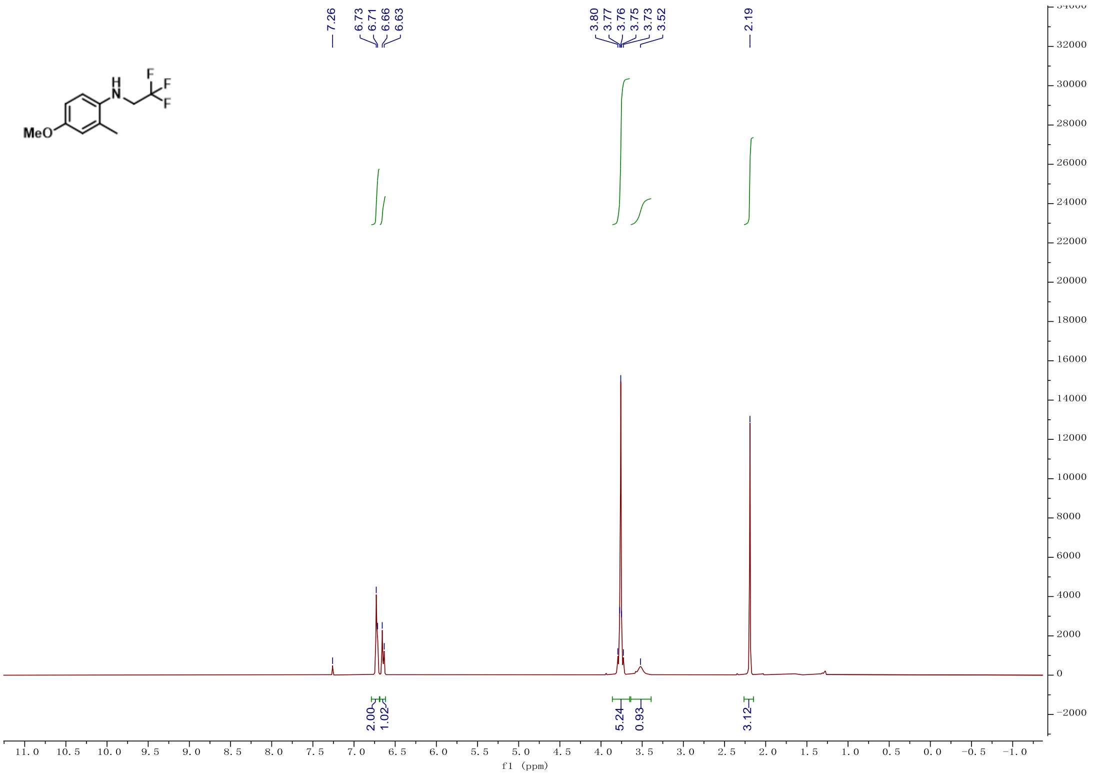

^{13}C NMR of Compound 7 (101 MHz, $CDCl_3$)

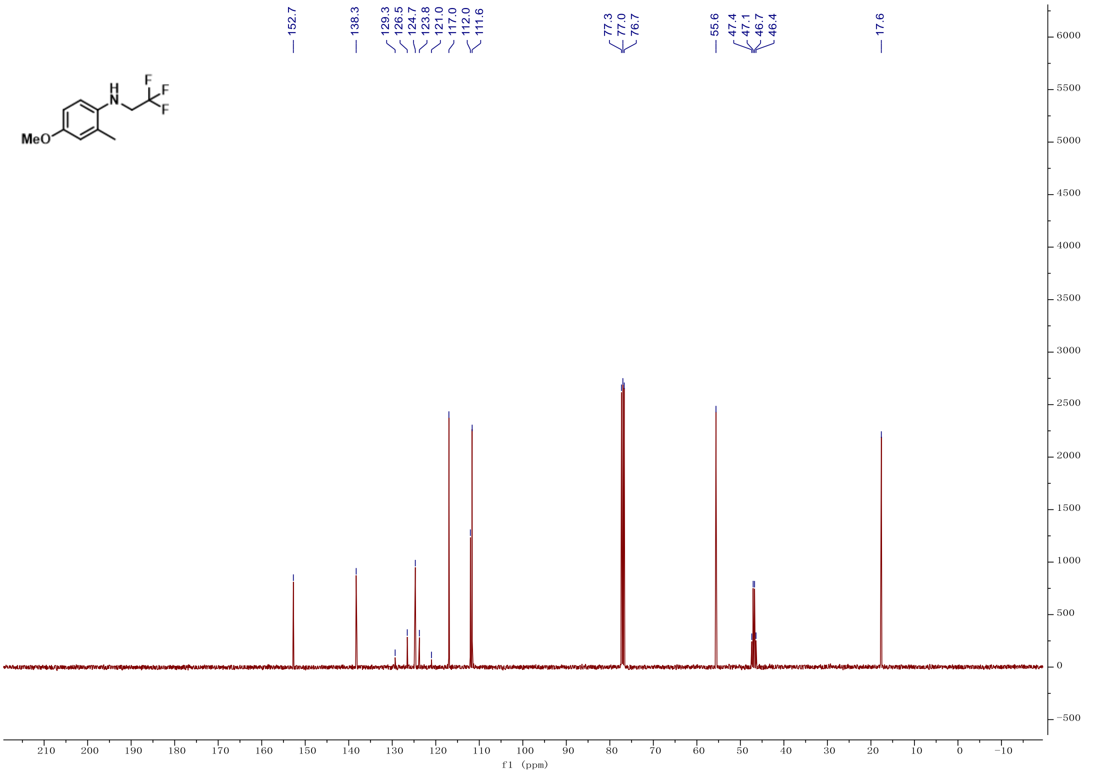

^{19}F NMR of Compound 7 (376 MHz, $CDCl_3$)

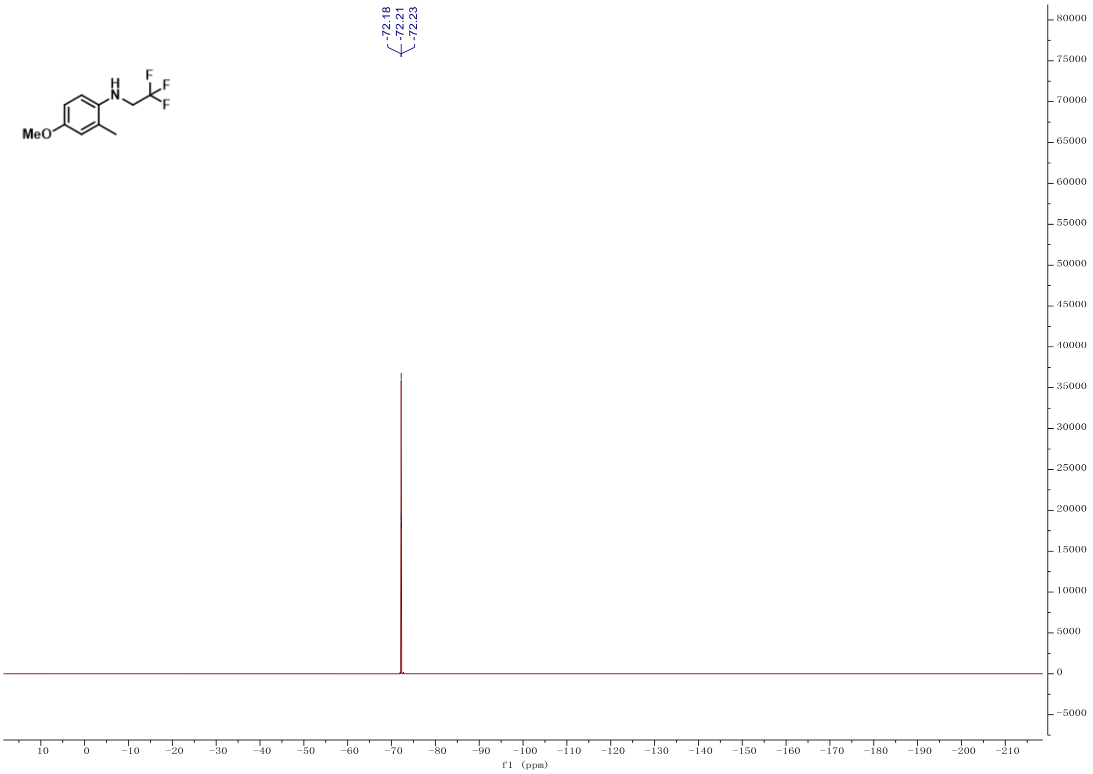

^{1}H NMR of Compound 8 (400 MHz, CDCl$_3$)

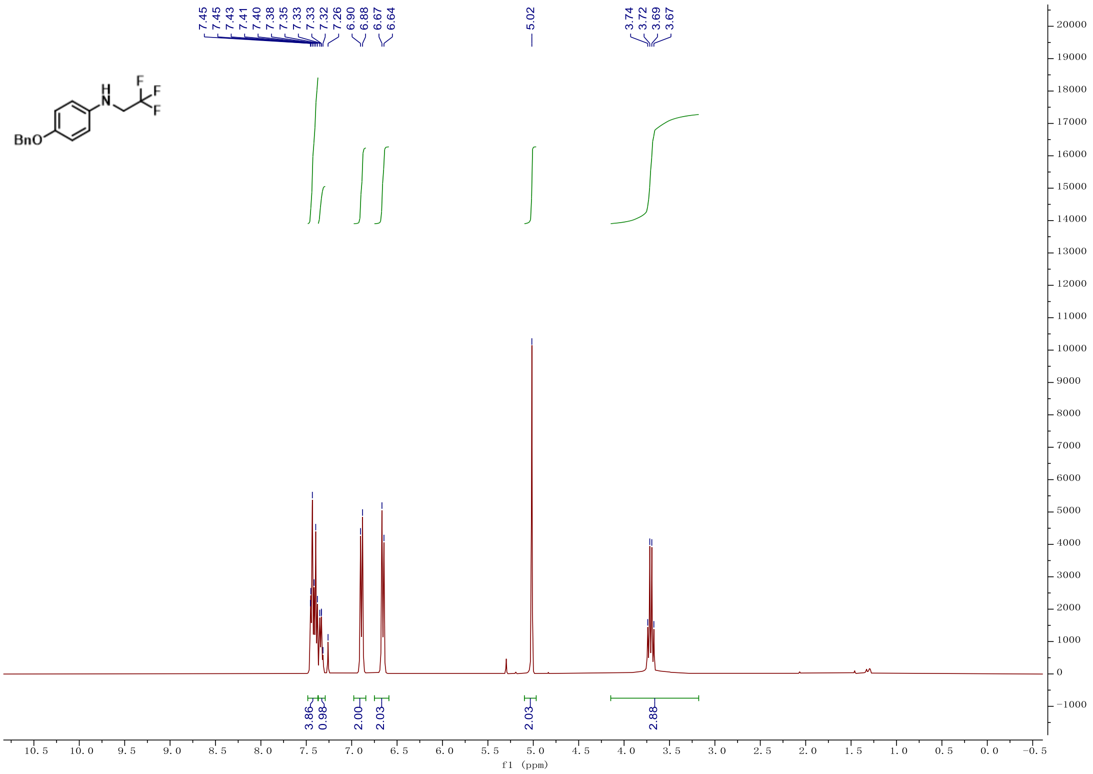

^{13}C NMR of Compound 8 (101 MHz, CDCl$_3$)

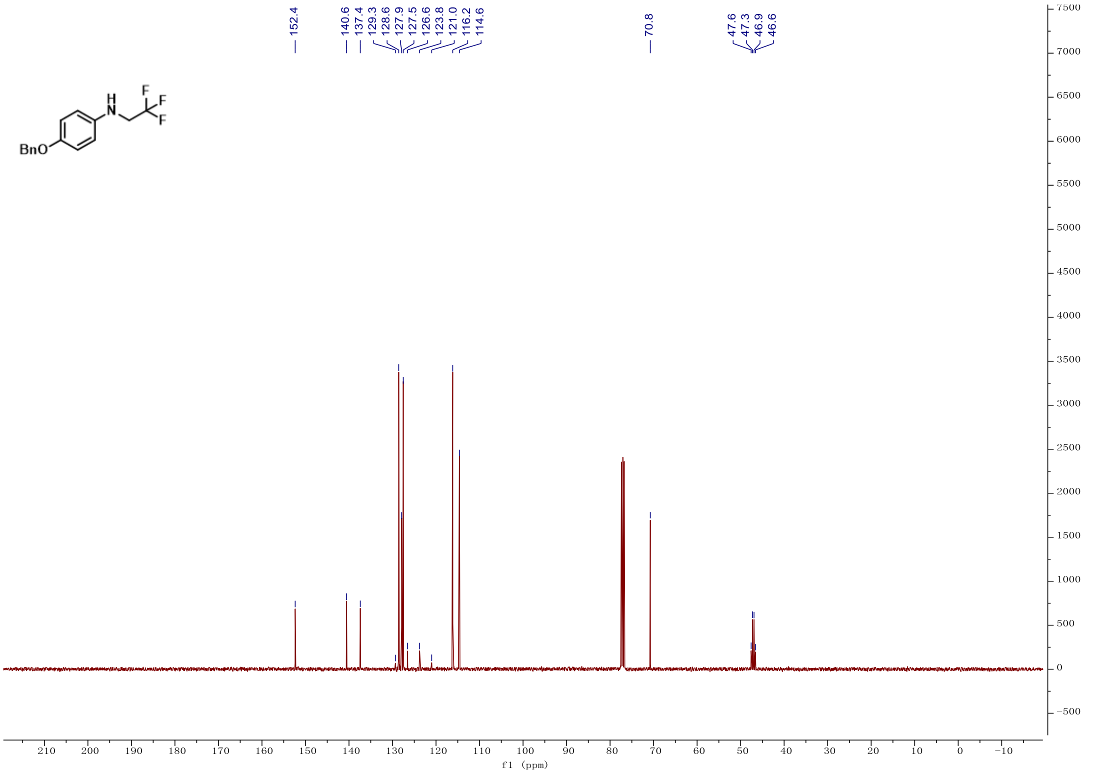

^{19}F NMR of Compound 8 (376 MHz, $CDCl_3$)

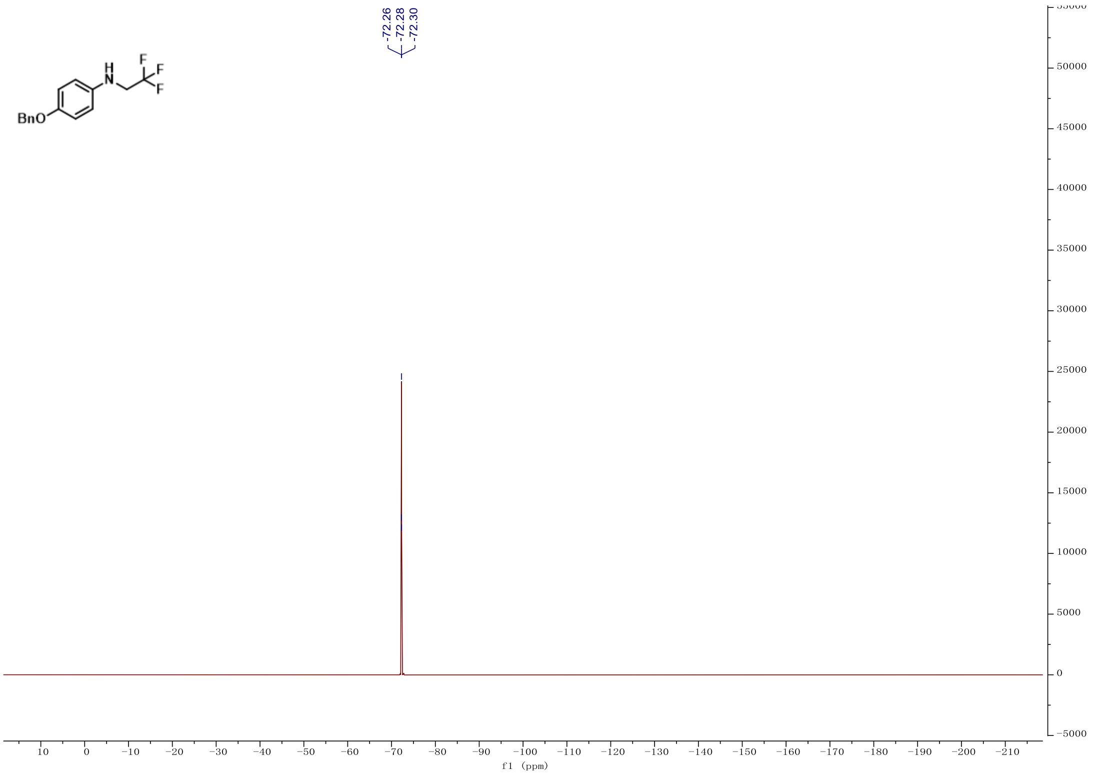

^{1}H NMR of Compound 9 (400 MHz, $CDCl_3$)

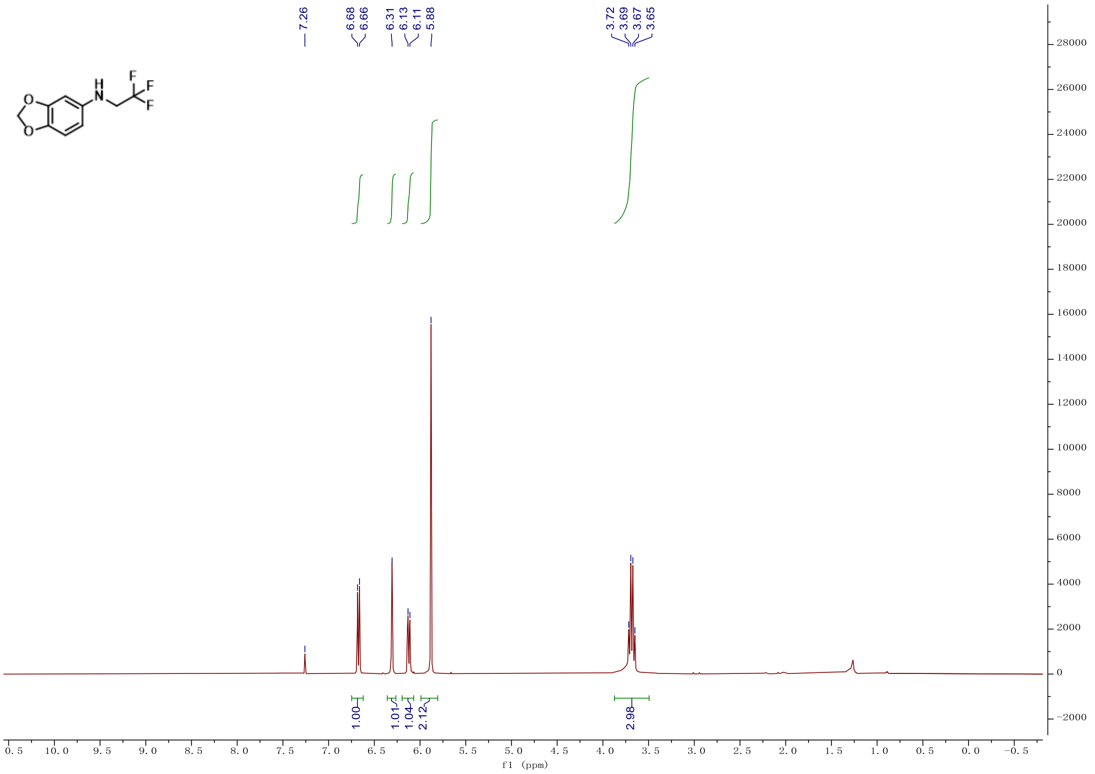

^{13}C NMR of Compound 9 (101 MHz, $CDCl_3$)

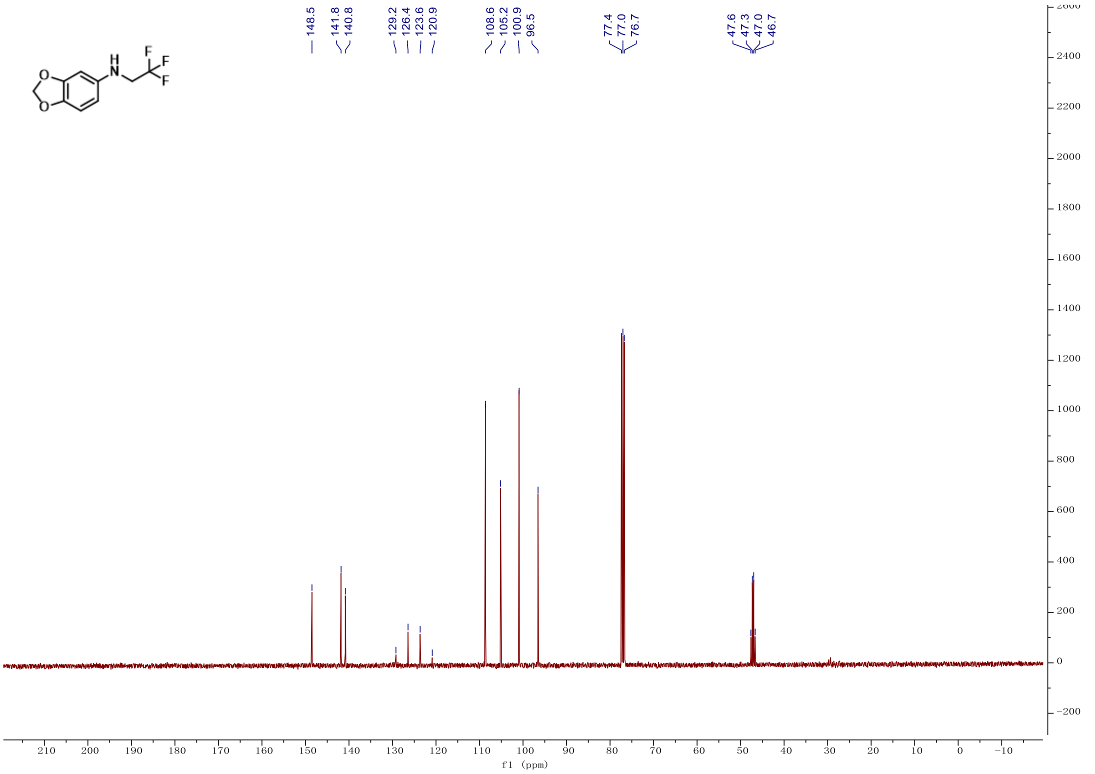

^{19}F NMR of Compound 9 (376 MHz, $CDCl_3$)

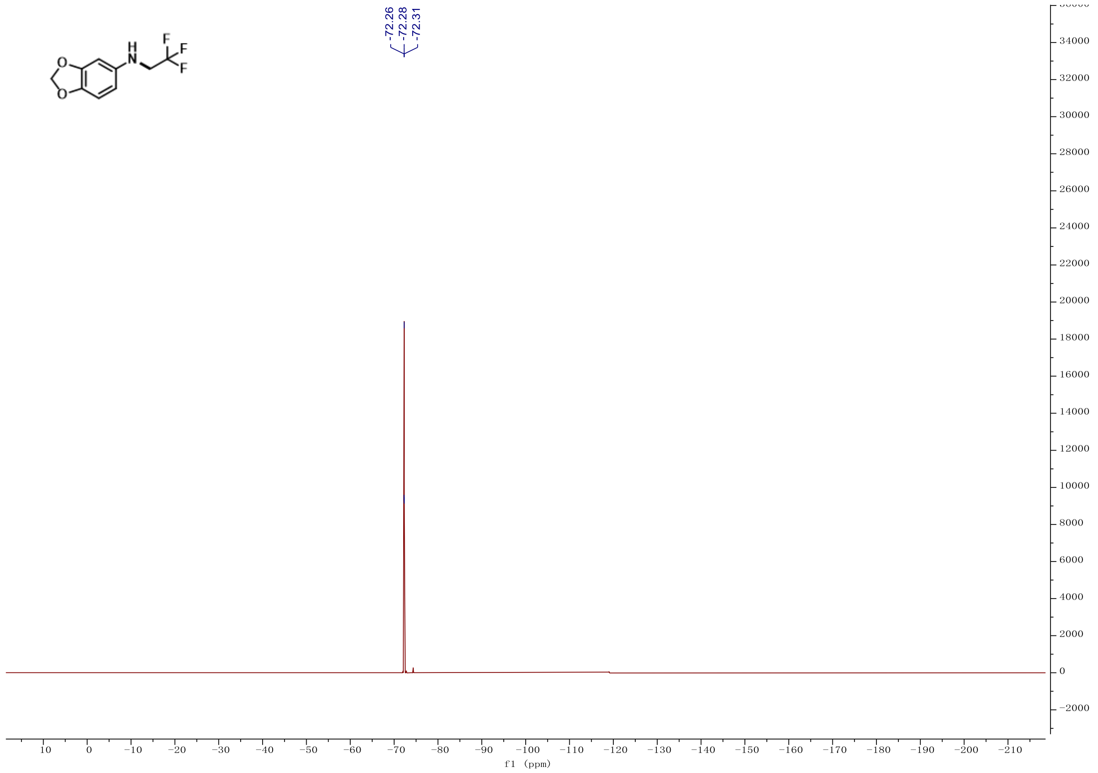

^{1}H NMR of Compound 10 (400 MHz, $CDCl_3$)

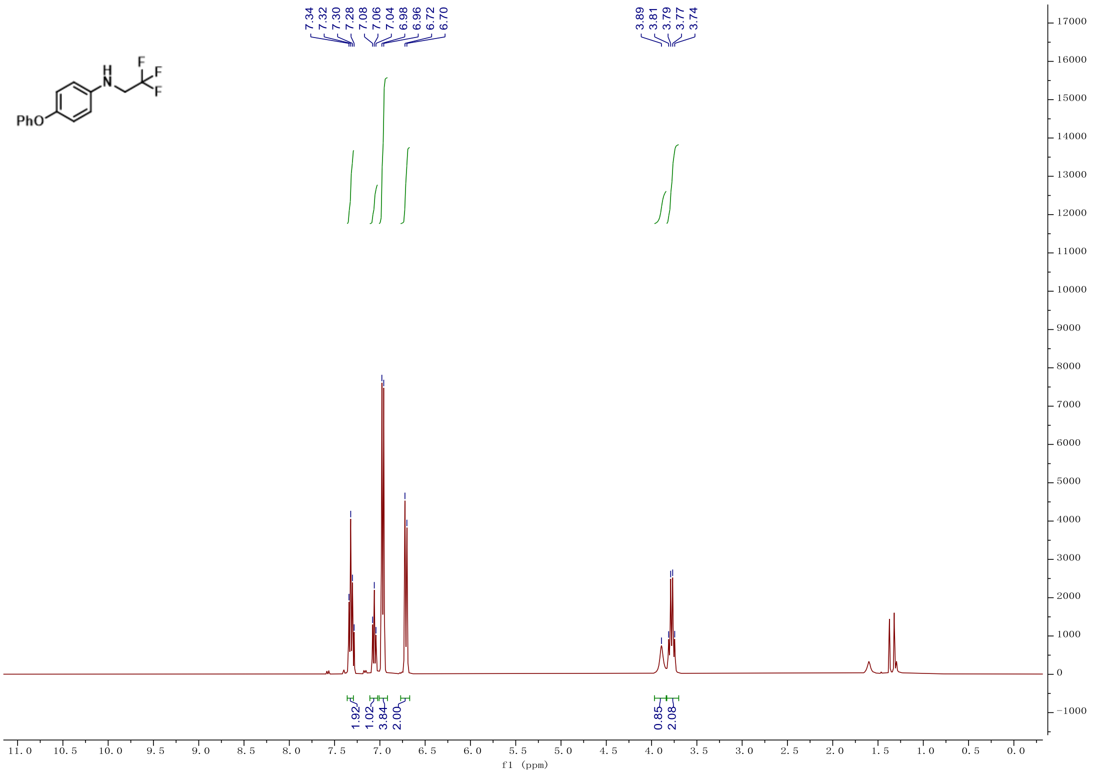

^{13}C NMR of Compound 10 (101 MHz, $CDCl_3$)

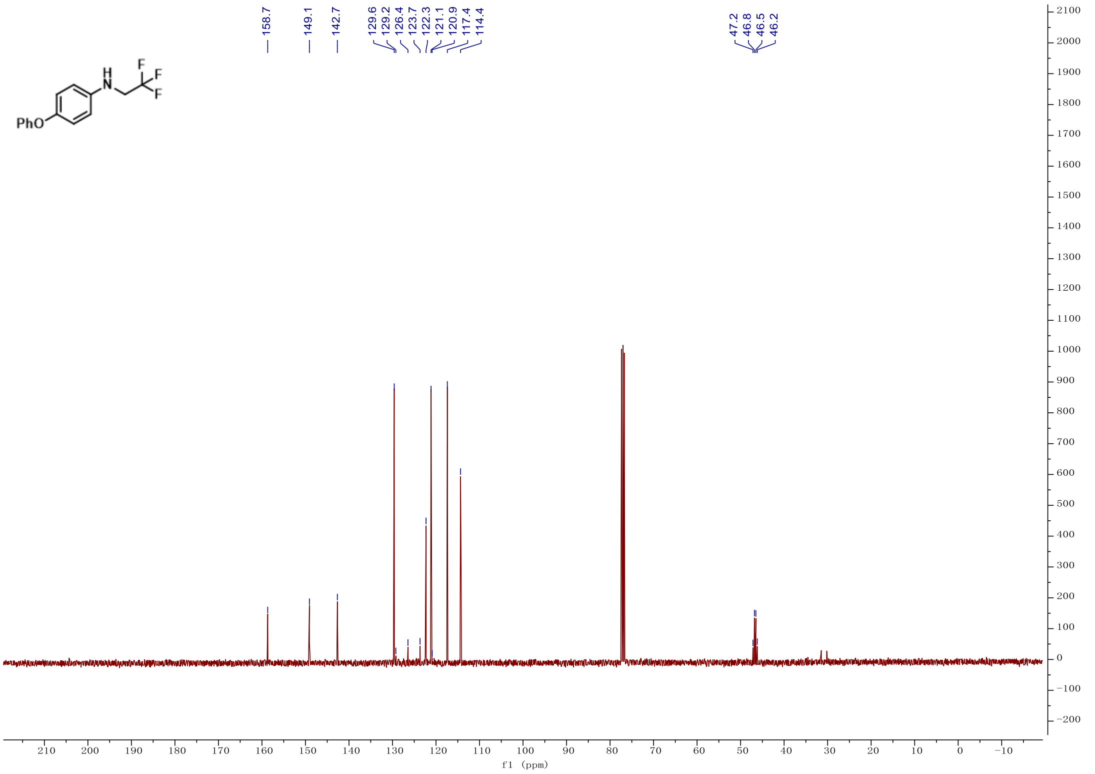

^{19}F NMR of Compound 10 (376 MHz, $CDCl_3$)

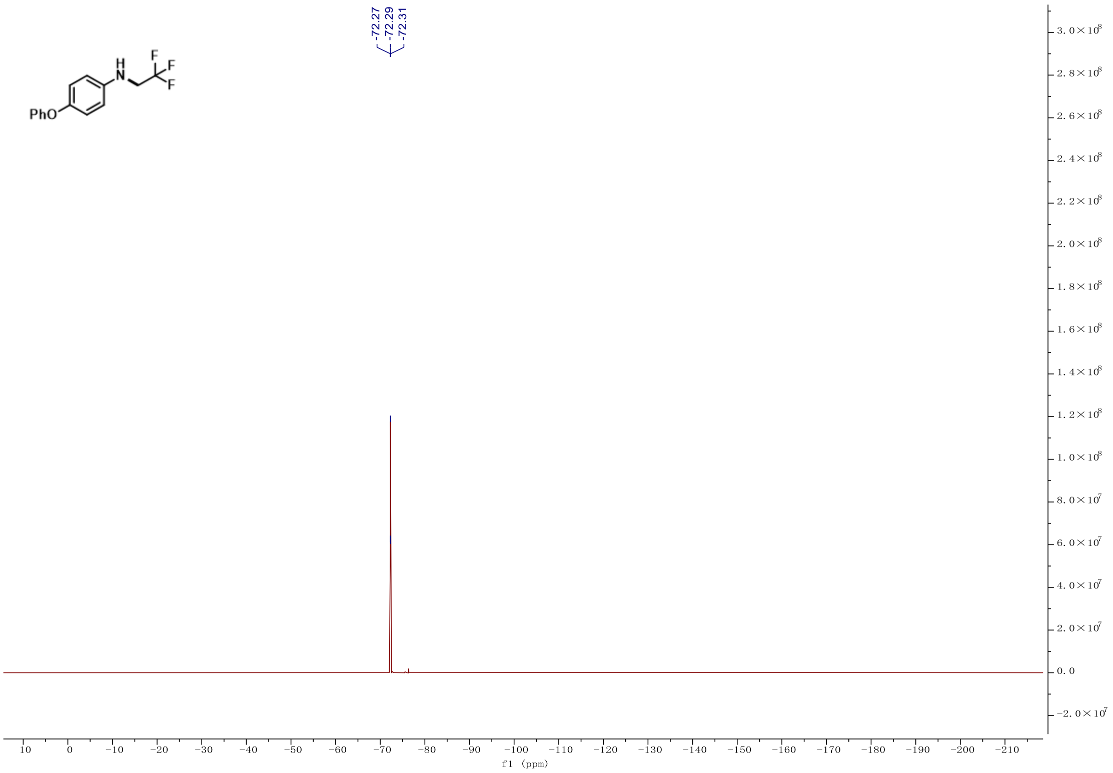

^{1}H NMR of Compound 11 (400 MHz, $CDCl_3$)

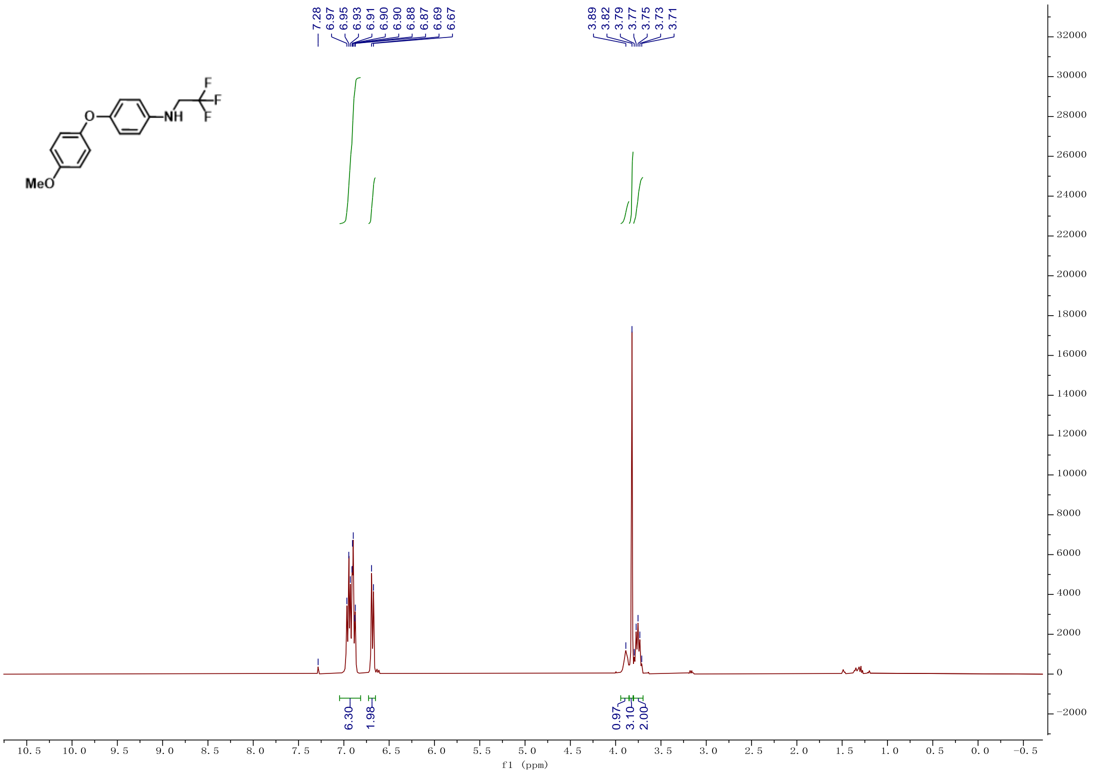

^{13}C NMR of Compound 11 (101 MHz, $CDCl_3$)

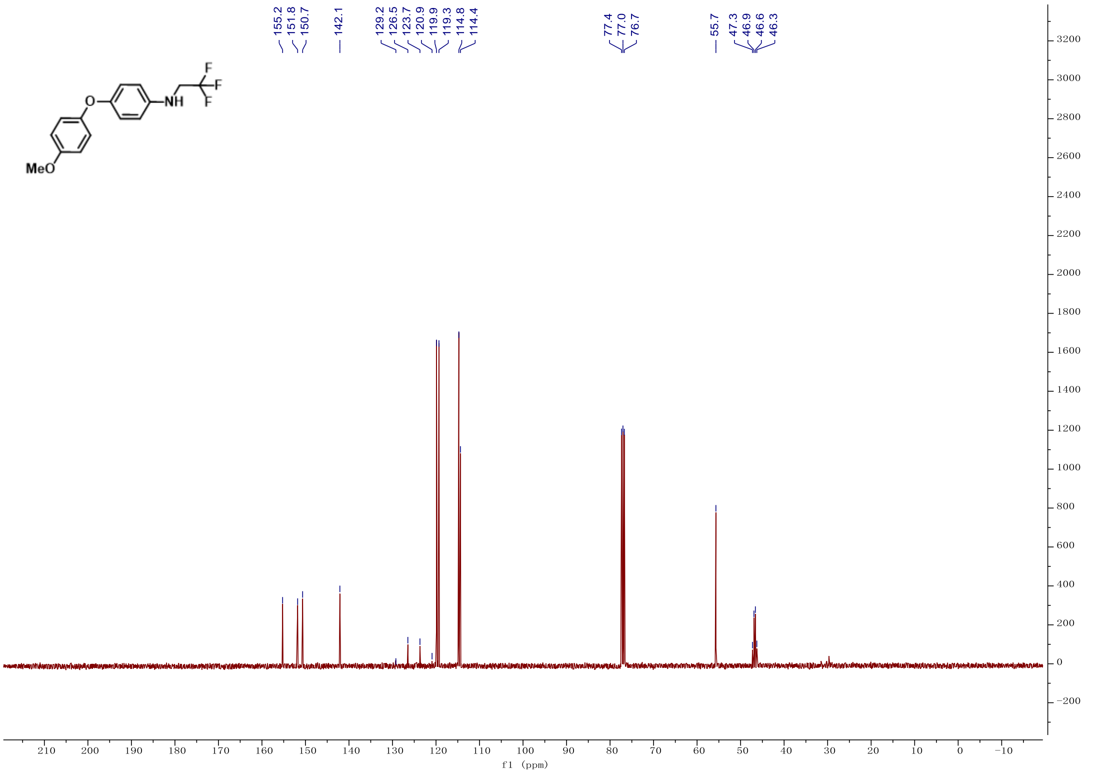

^{19}F NMR of Compound 11 (376 MHz, $CDCl_3$)

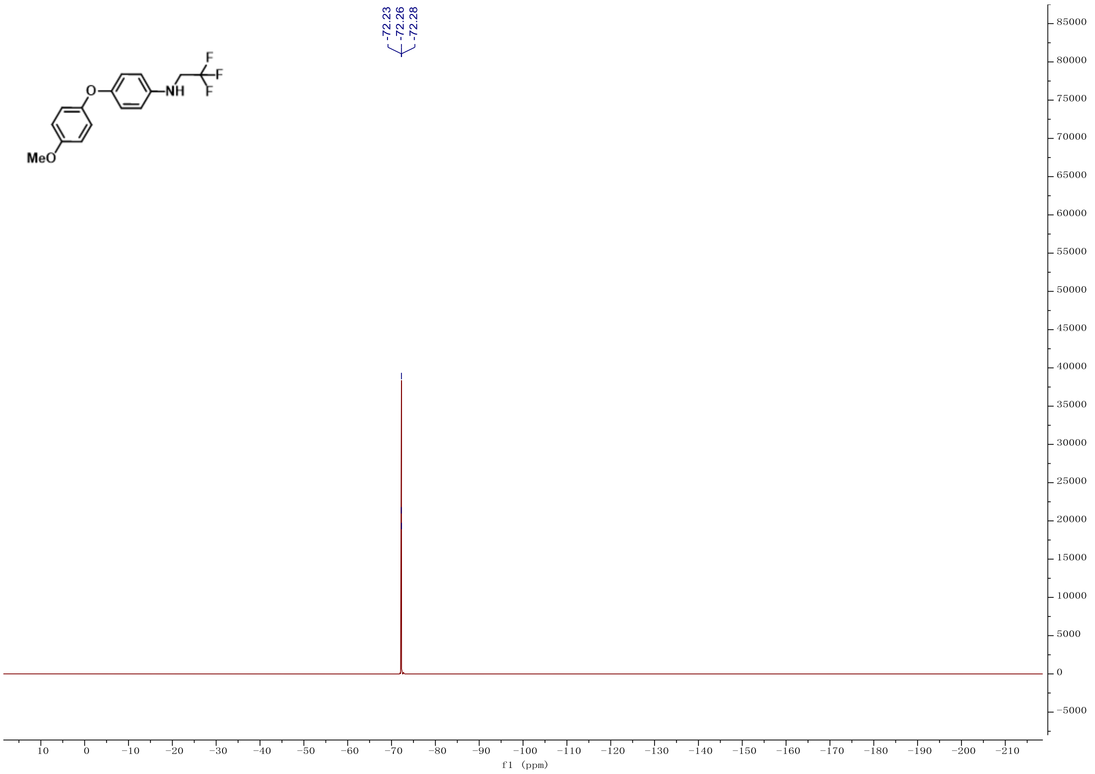

^{1}H NMR of Compound 12 (400 MHz, $CDCl_3$)

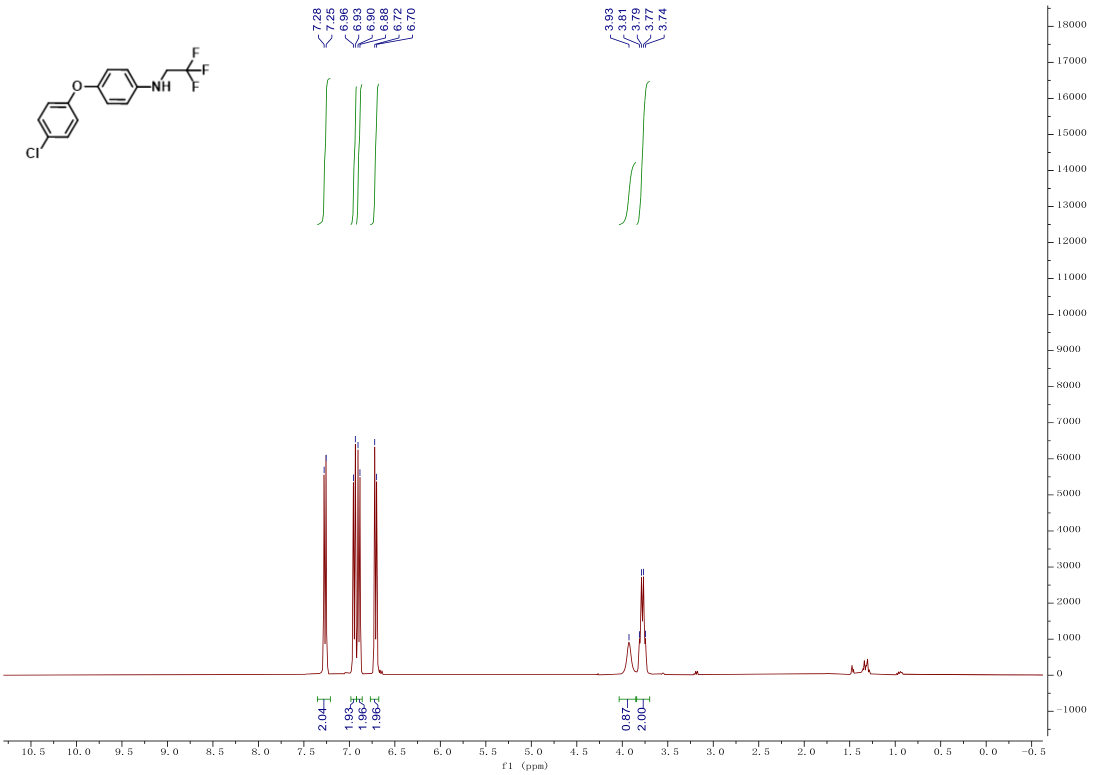

^{13}C NMR of Compound 12 (101 MHz, $CDCl_3$)

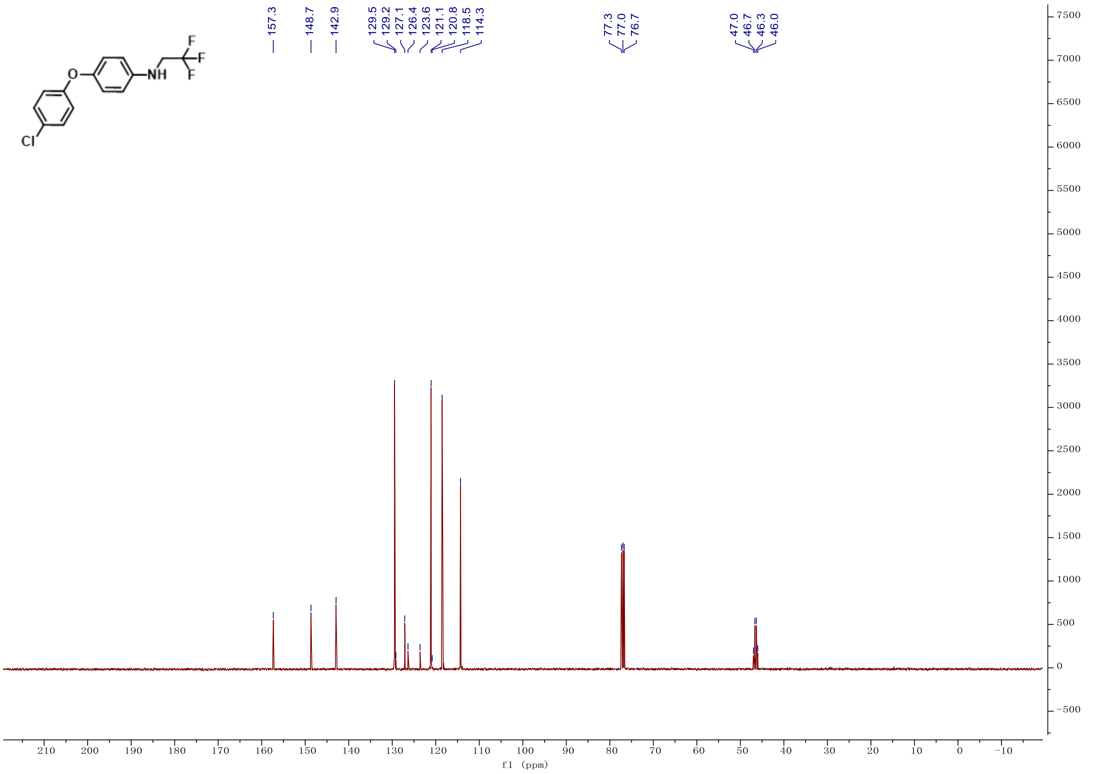

^{19}F NMR of Compound 12 (376 MHz, $CDCl_3$)

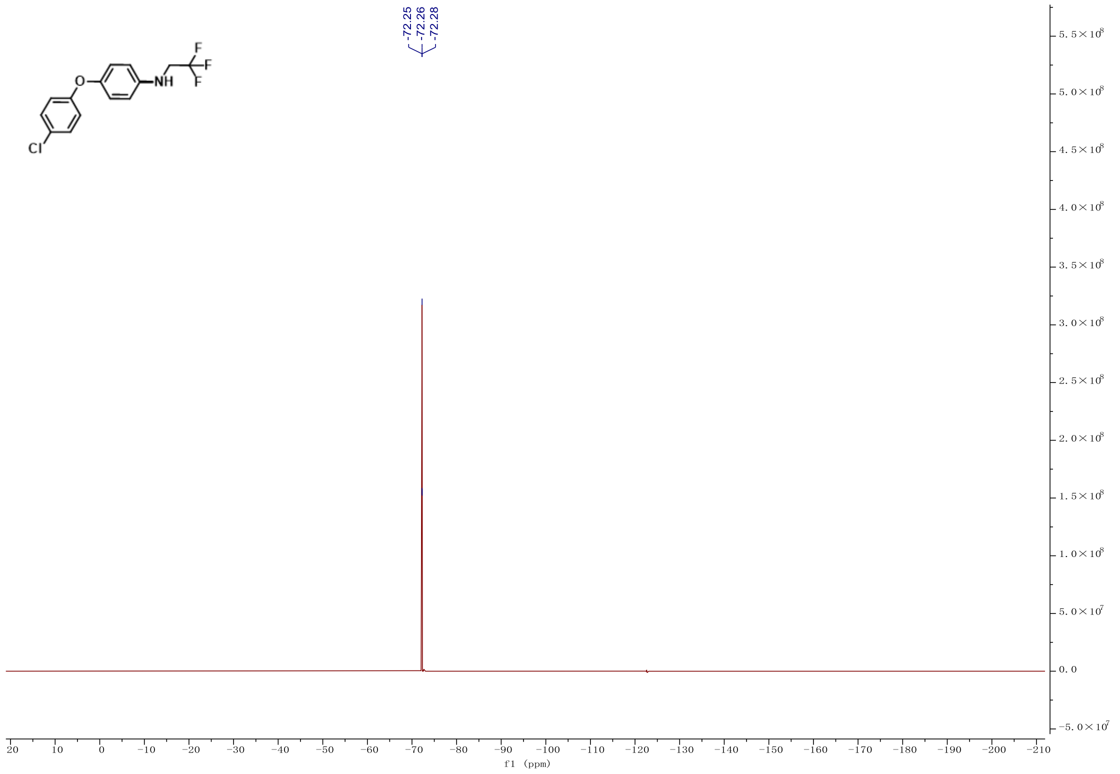

^1H NMR of Compound 13 (400 MHz, $CDCl_3$)

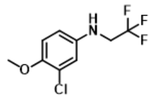

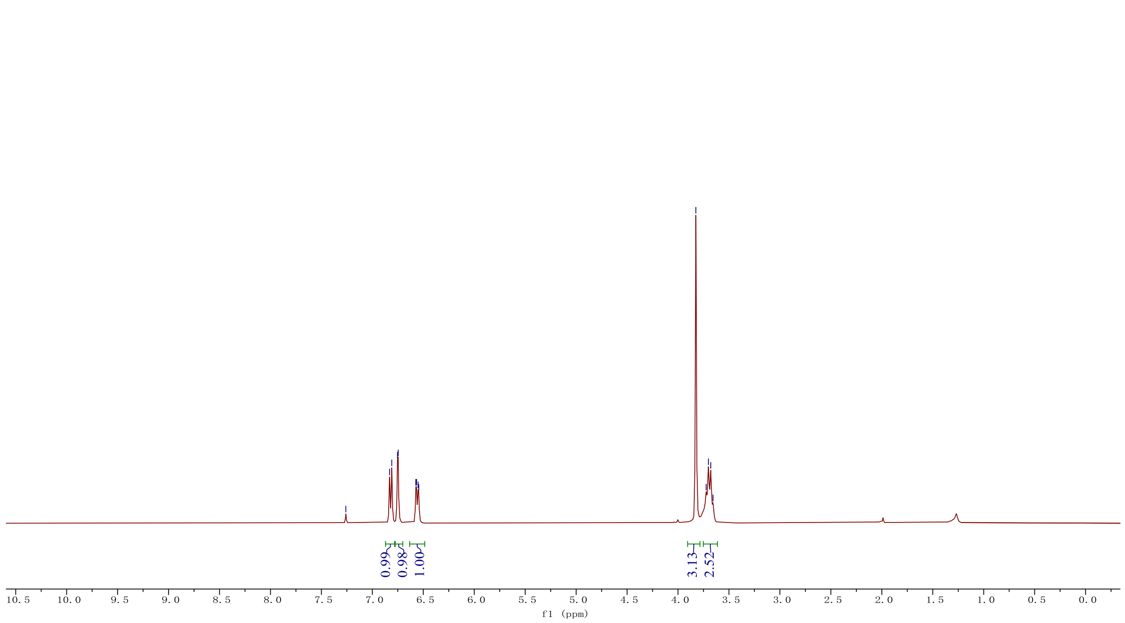

^{13}C NMR of Compound 13 (101 MHz, $CDCl_3$)

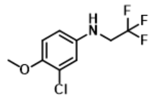

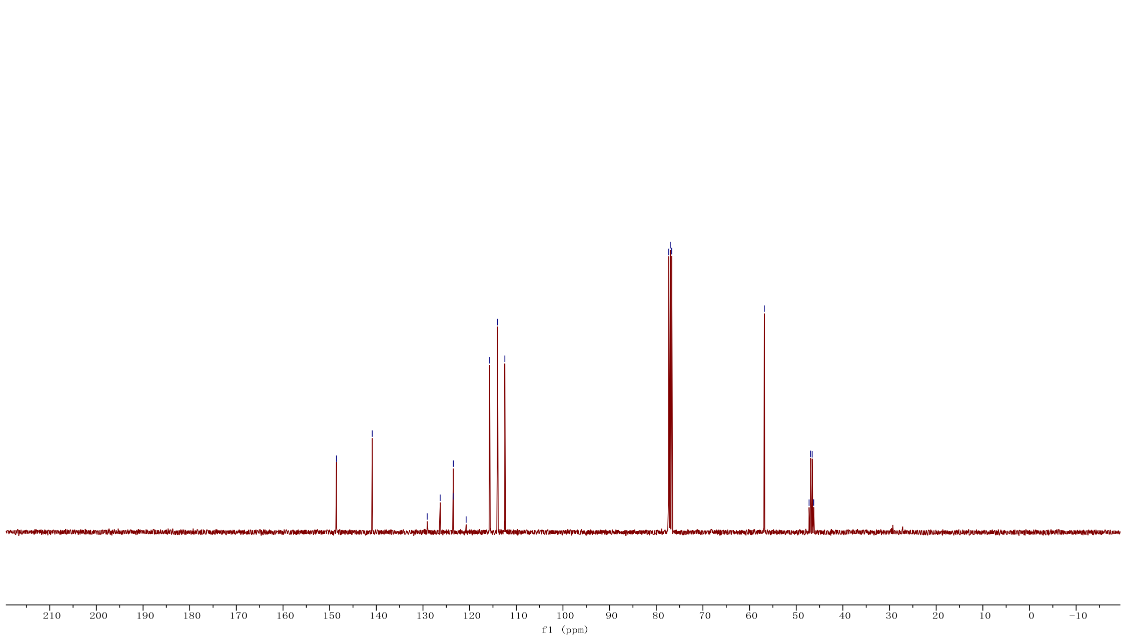

^{19}F NMR of Compound 13 (376 MHz, $CDCl_3$)

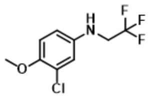

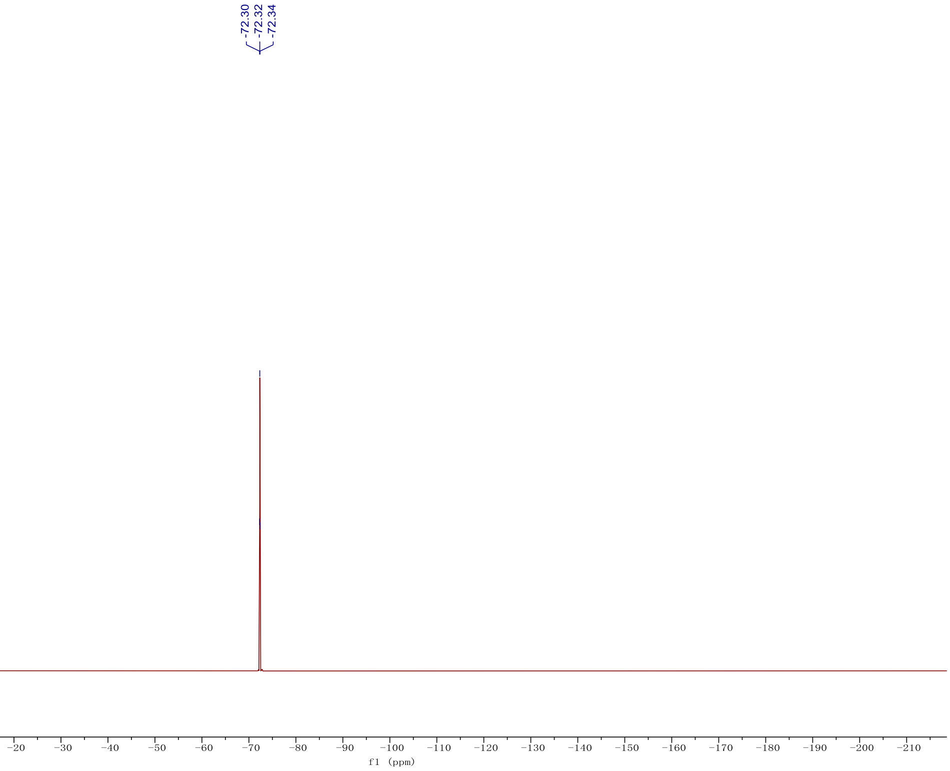

^{1}H NMR of Compound 14 (400 MHz, $CDCl_3$)

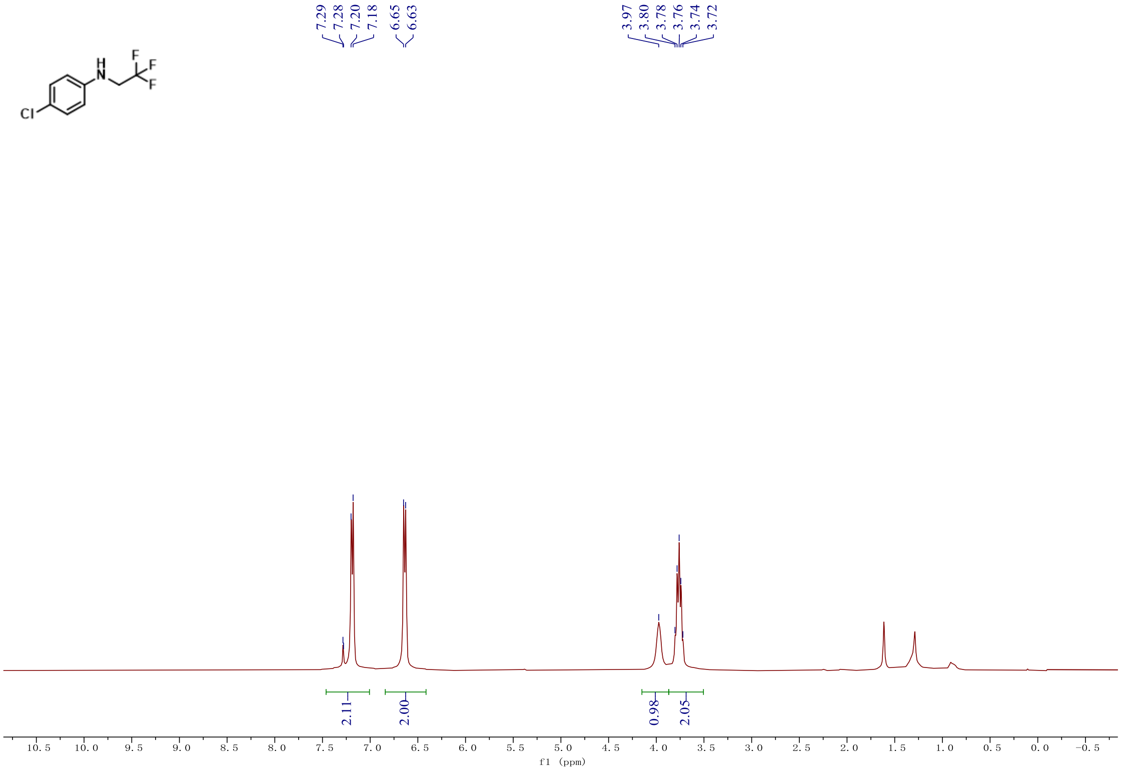

^{13}C NMR of Compound 14 (101 MHz, $CDCl_3$)

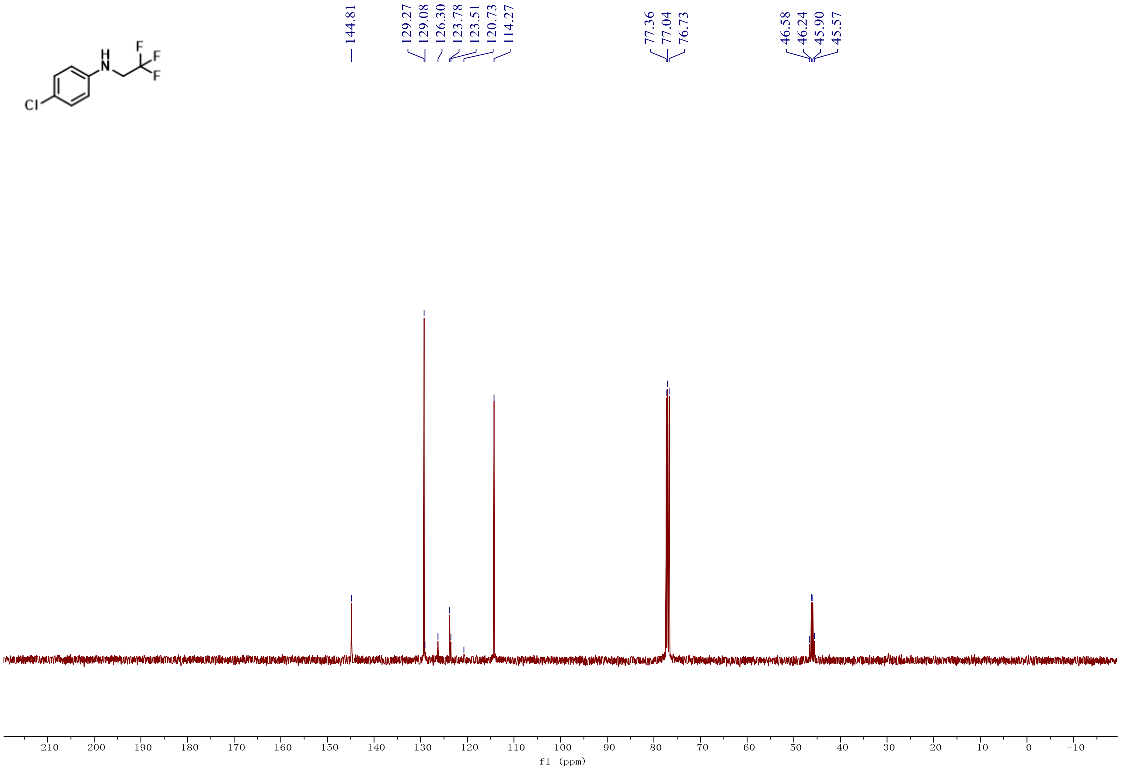

^{19}F NMR of Compound 14 (376 MHz, $CDCl_3$)

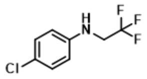

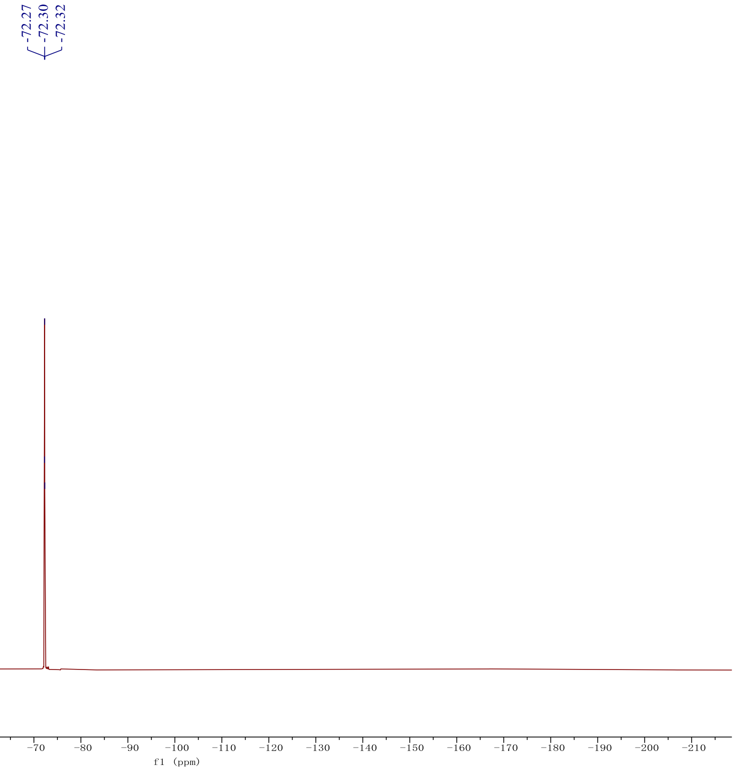

^{1}H NMR of Compound 15 (400 MHz, $CDCl_3$)

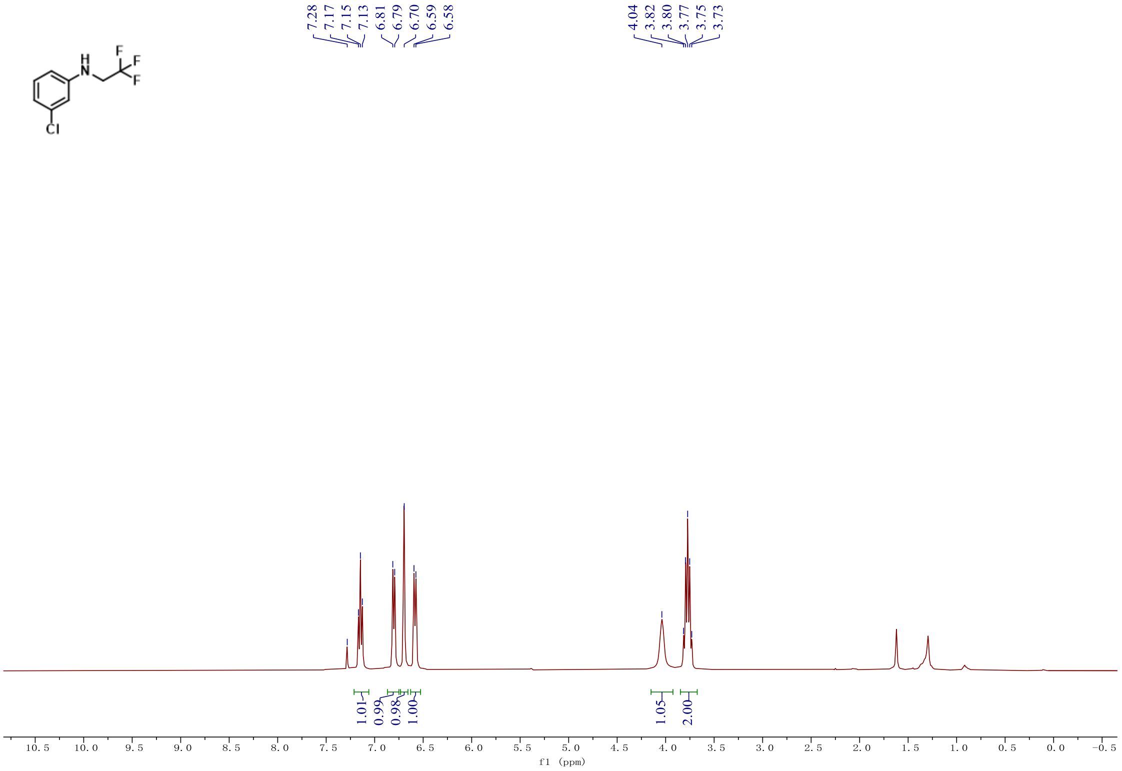

^{13}C NMR of Compound 15 (101 MHz, $CDCl_3$)

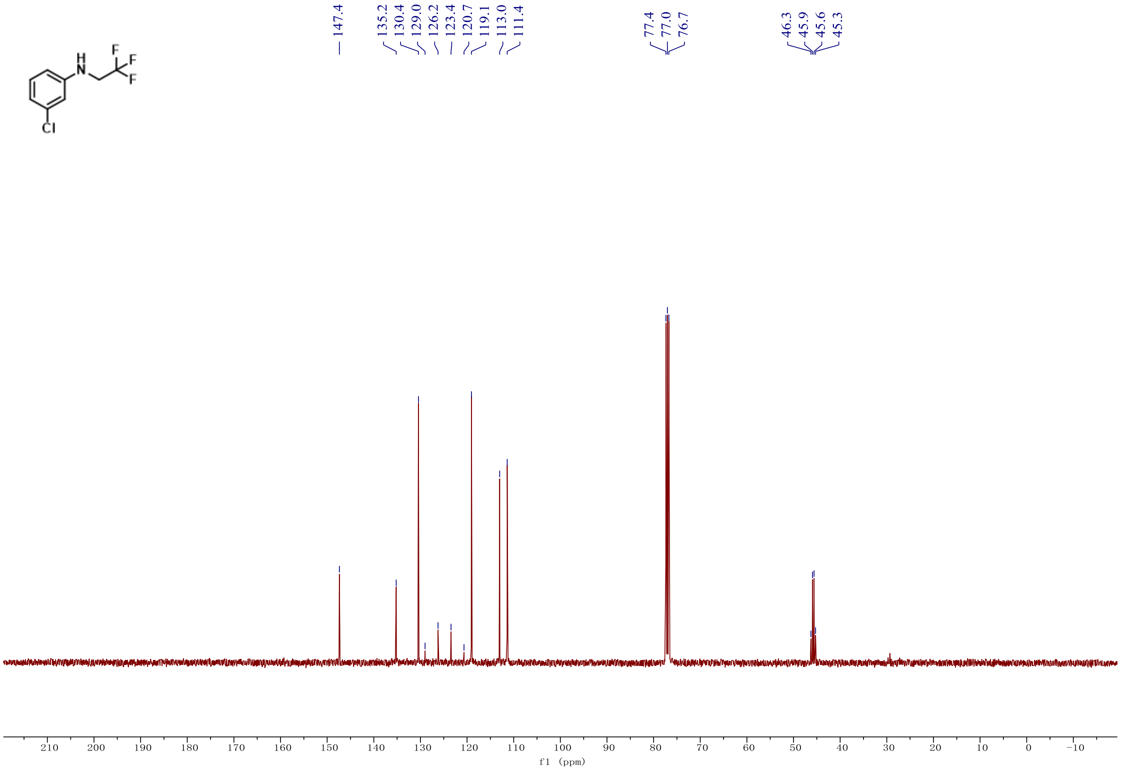

^{19}F NMR of Compound 15 (376 MHz, $CDCl_3$)

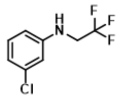

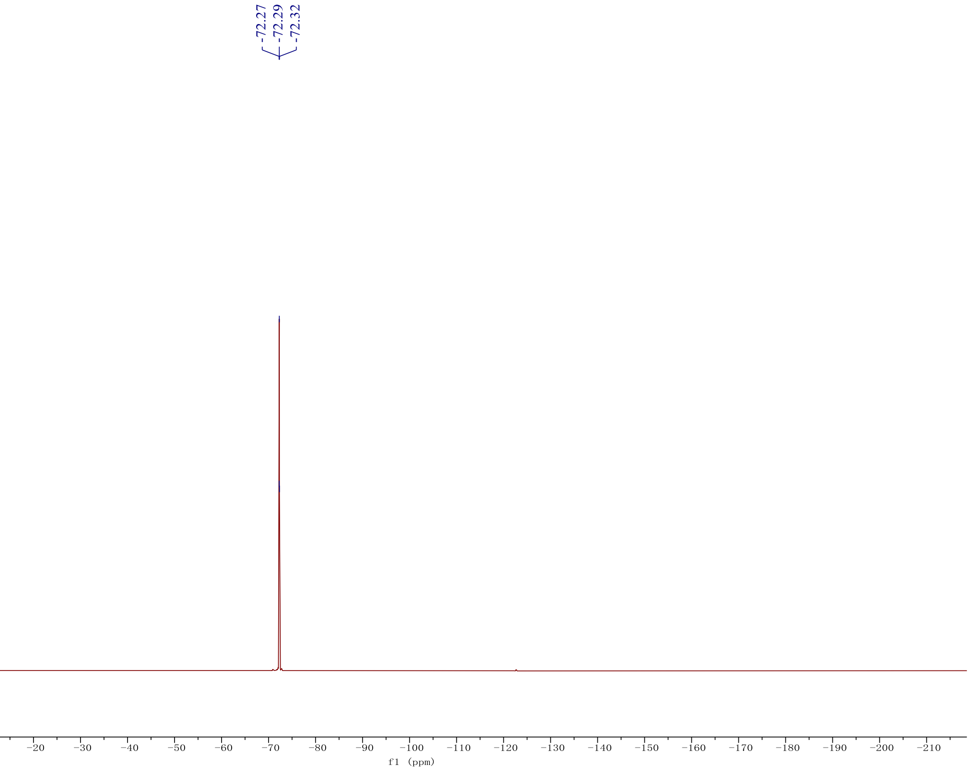

^{1}H NMR of Compound 16 (400 MHz, $CDCl_3$)

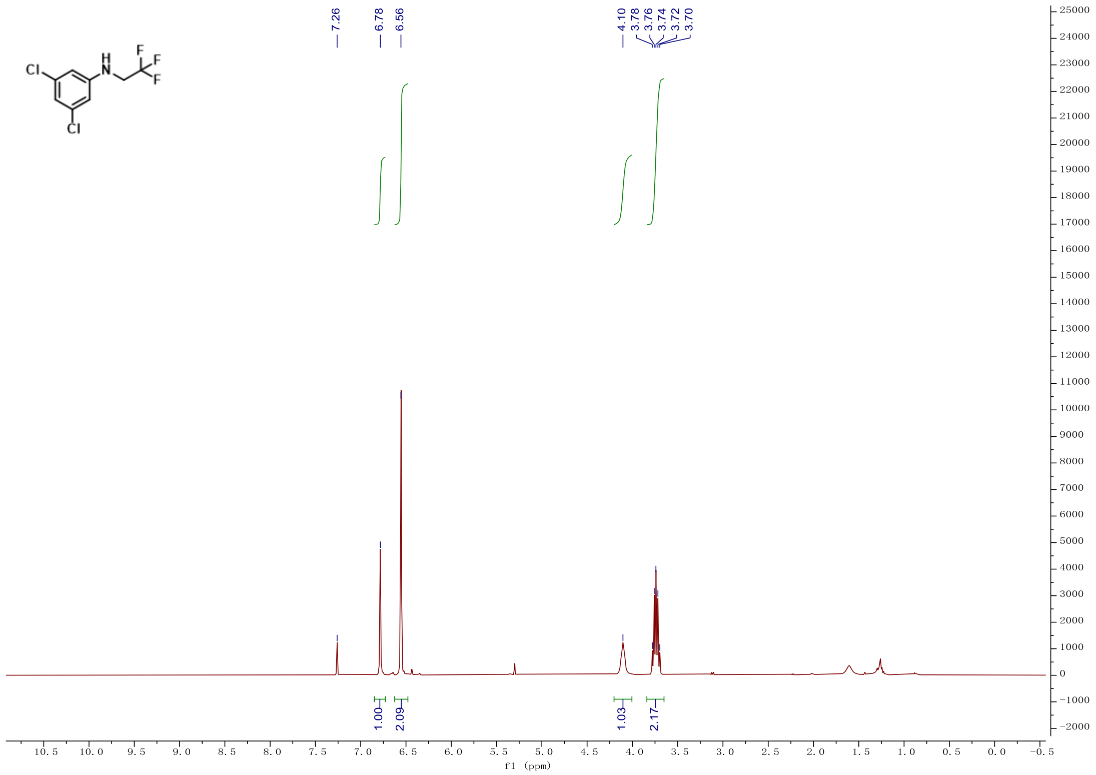

^{13}C NMR of Compound 16 (101 MHz, $CDCl_3$)

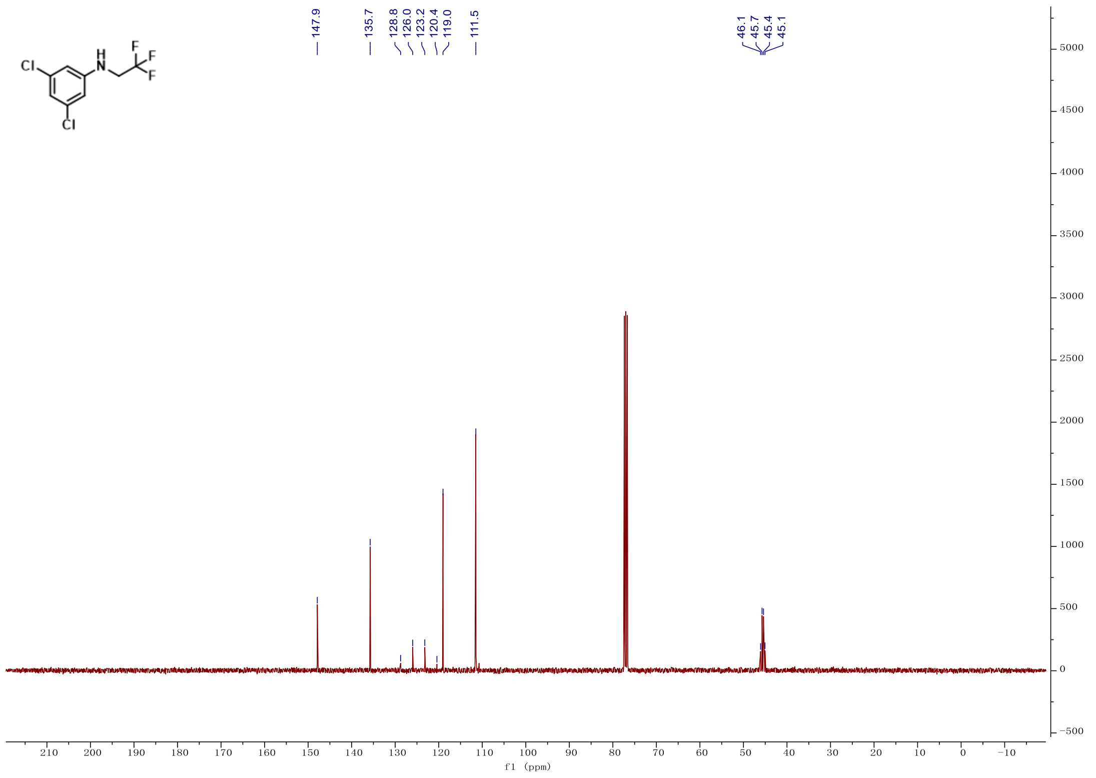

^{19}F NMR of Compound 16 (376 MHz, $CDCl_3$)

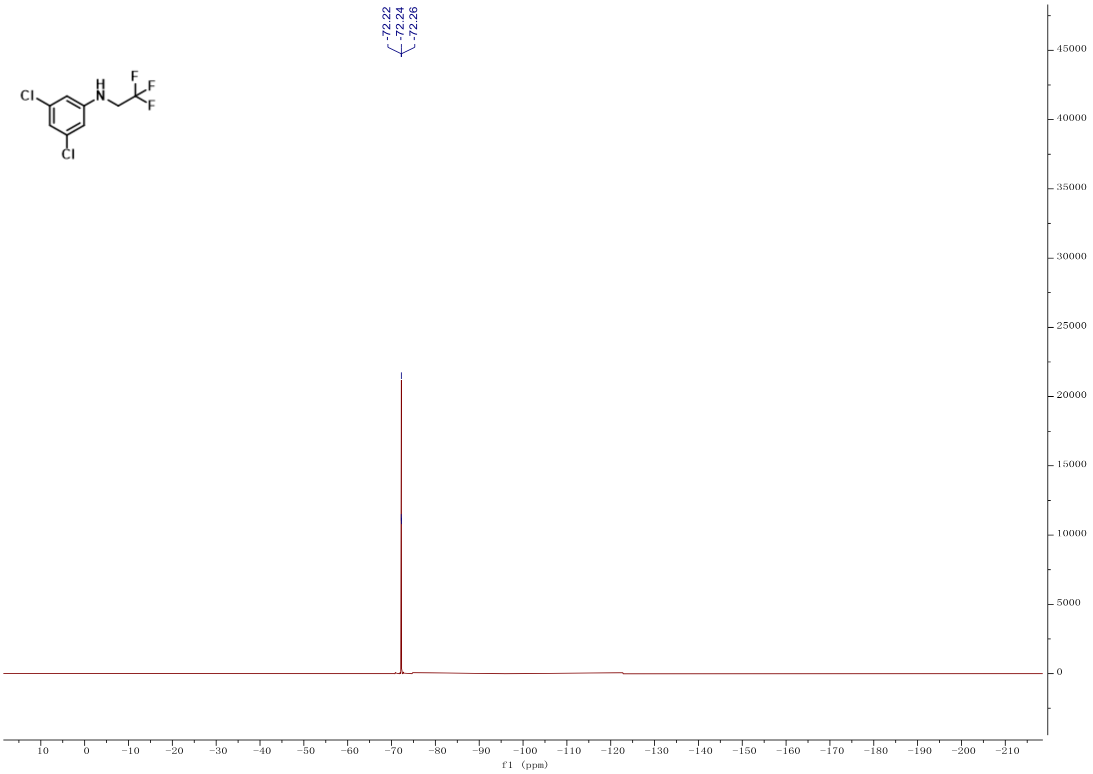

^{1}H NMR of Compound 17 (400 MHz, $CDCl_3$)

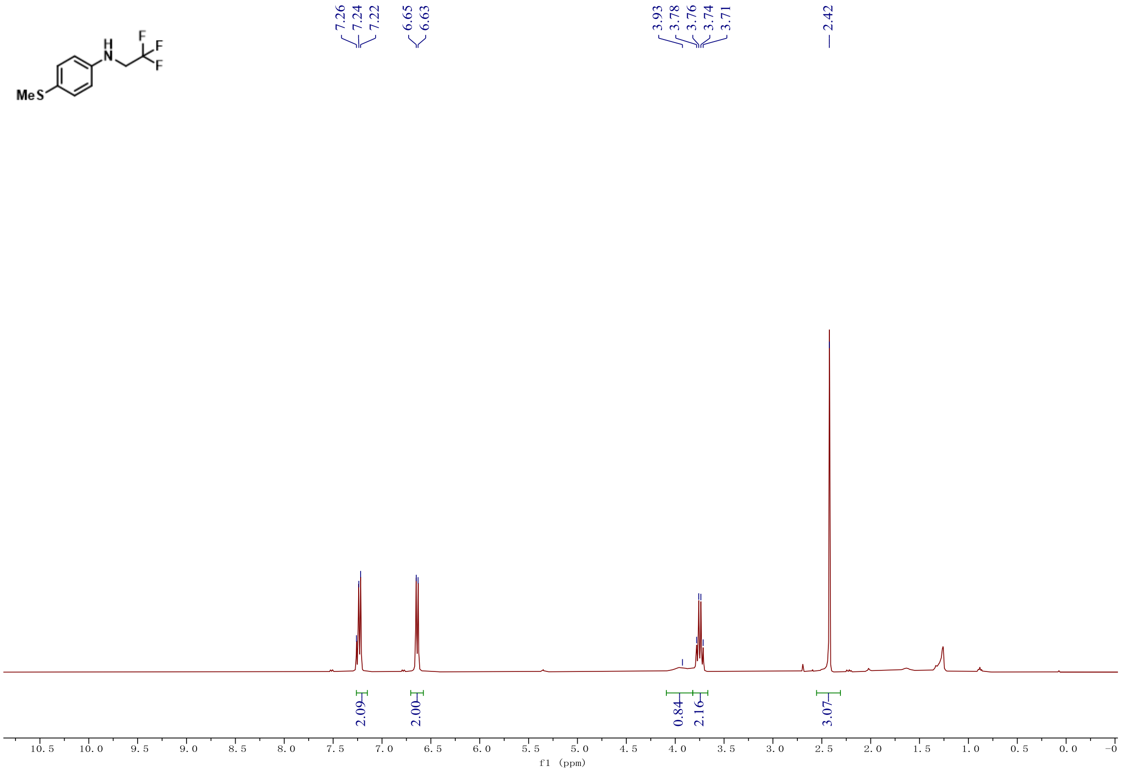

^{13}C NMR of Compound 17 (101 MHz, $CDCl_3$)

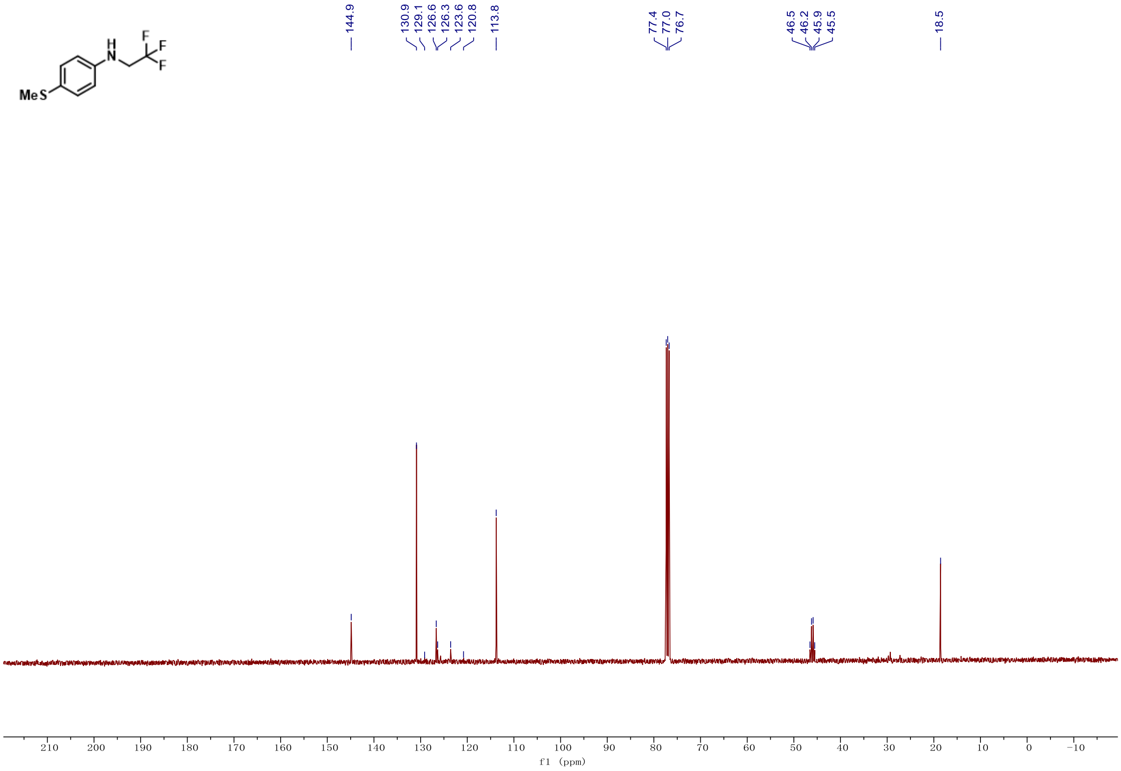

^{19}F NMR of Compound 17 (376 MHz, $CDCl_3$)

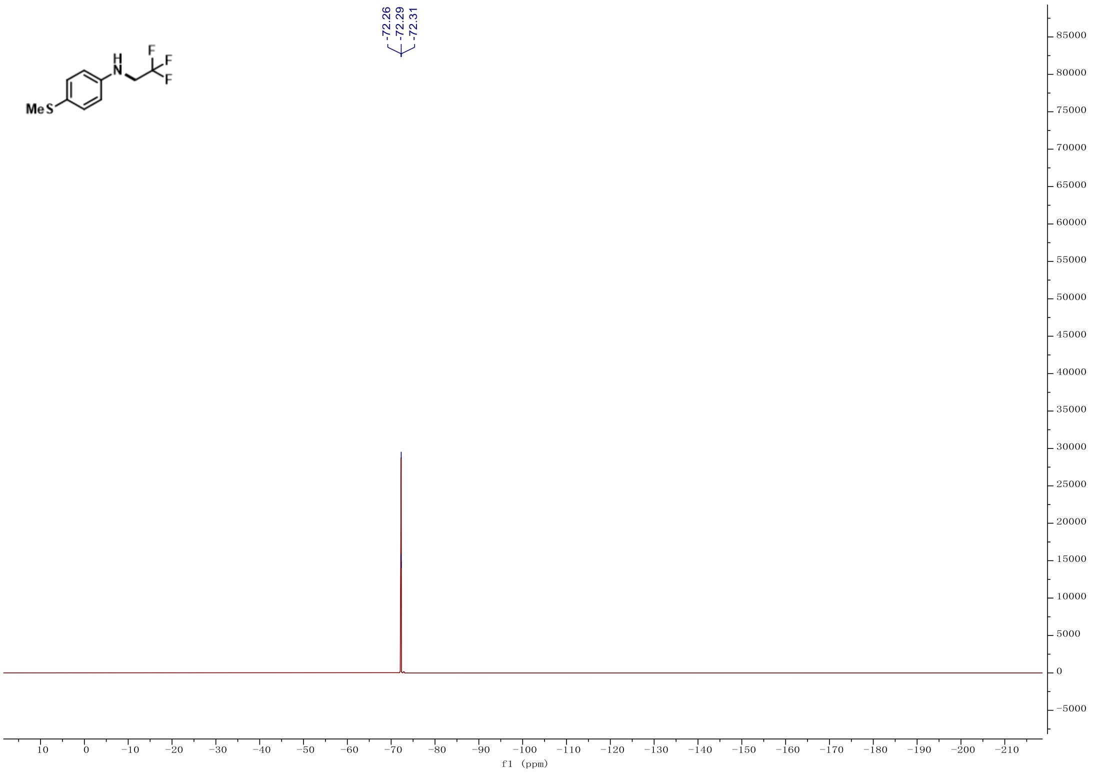

^{1}H NMR of Compound 18 (400 MHz, DMSO-d^{6})

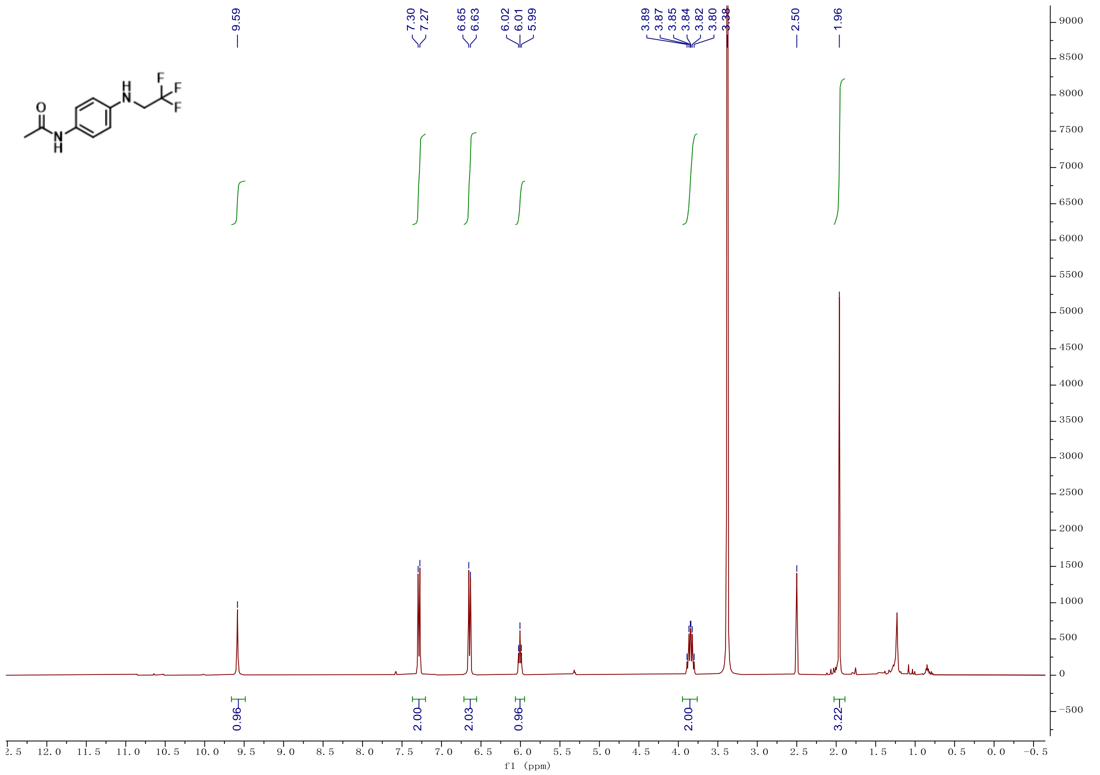

^{13}C NMR of Compound 18 (101 MHz,DMSO-d^{6})

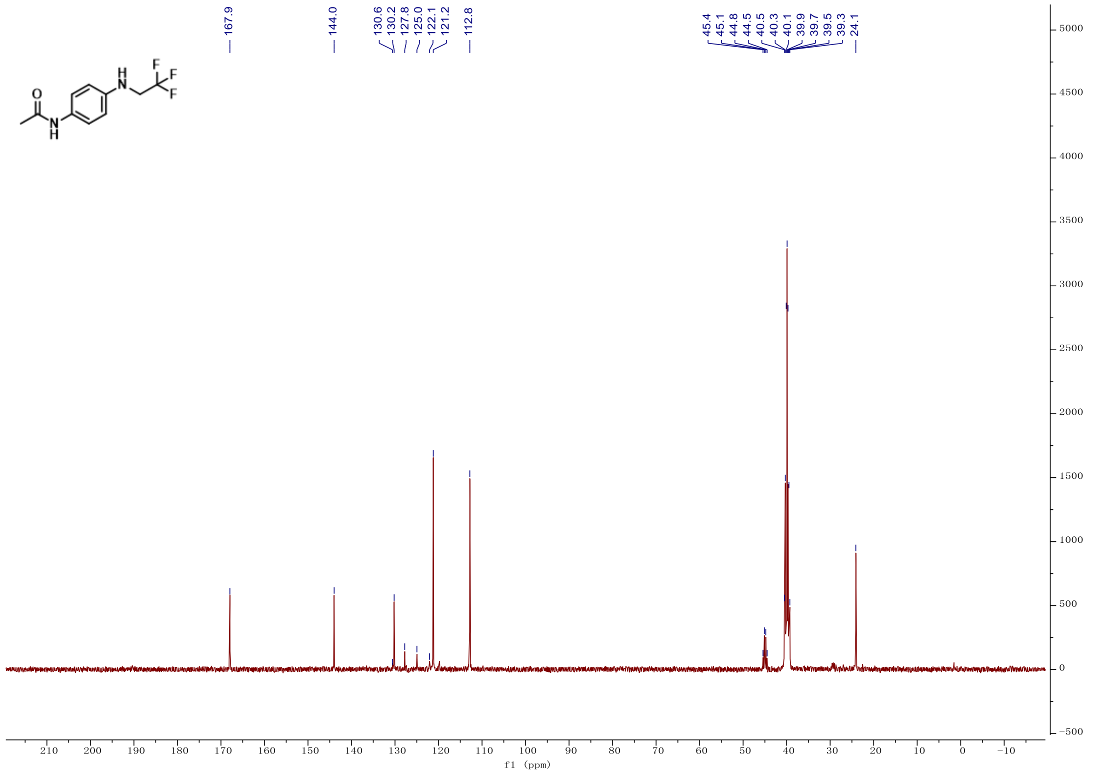

^{19}F NMR of Compound 18 (376 MHz, DMSO-d^6)

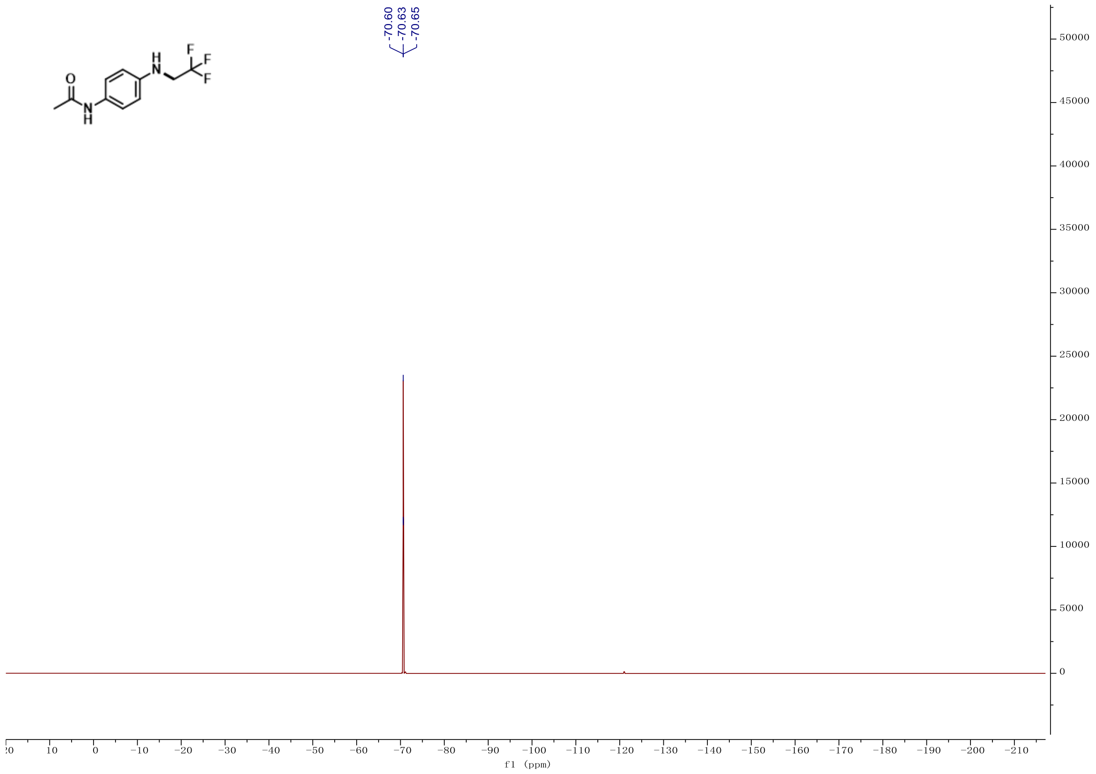

^{1}H NMR of Compound 19 (400 MHz, DMSO-d^{6})

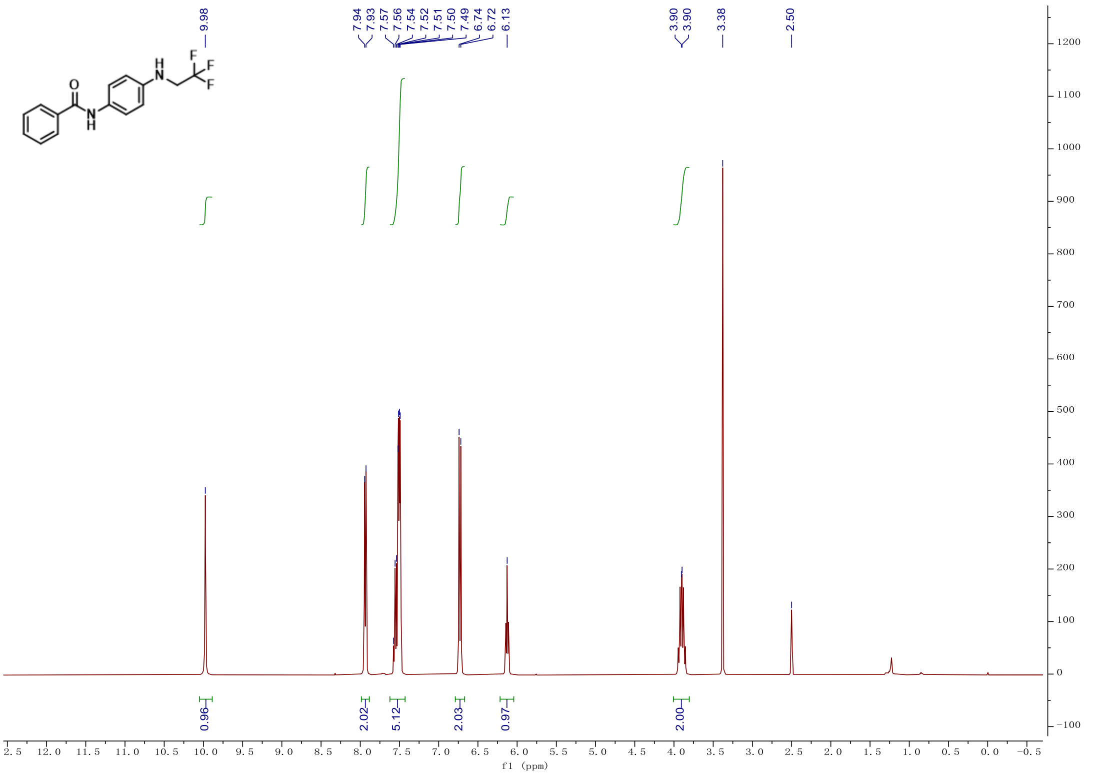

^{13}C NMR of Compound 19 (101 MHz, DMSO-d^{6})

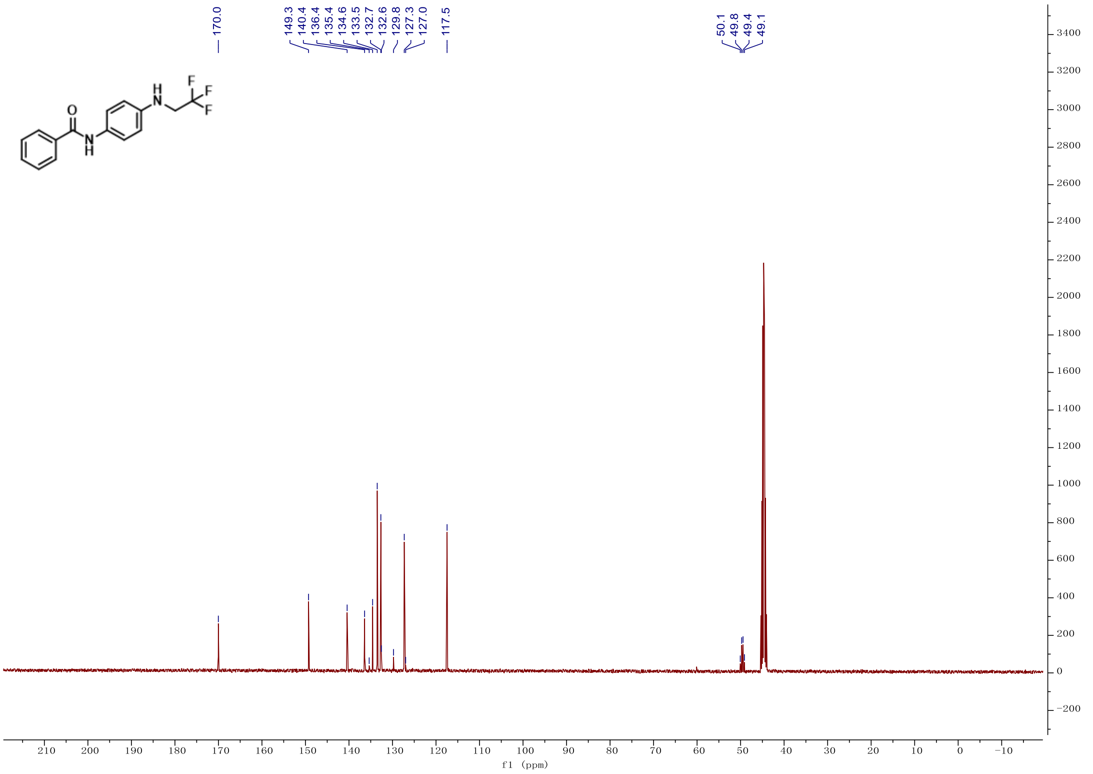

^{19}F NMR of Compound 19 (376 MHz, DMSO-d^6)

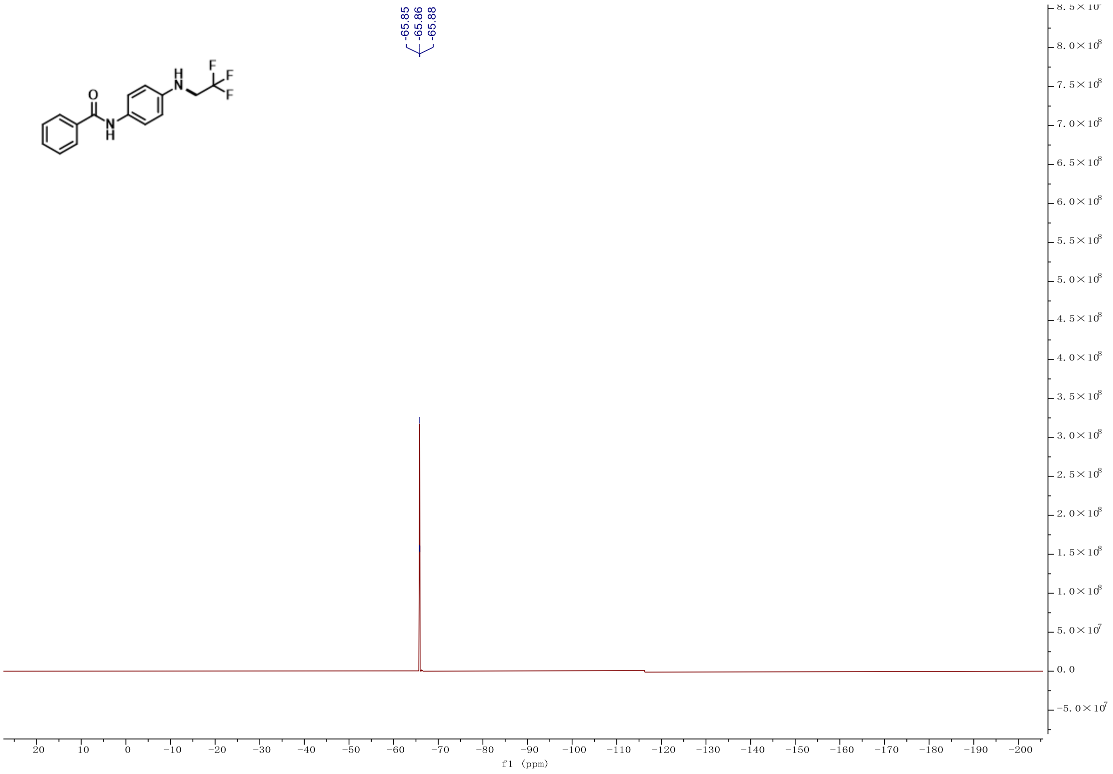

^{1}H NMR of Compound 20 (400 MHz, DMSO-d^{6})

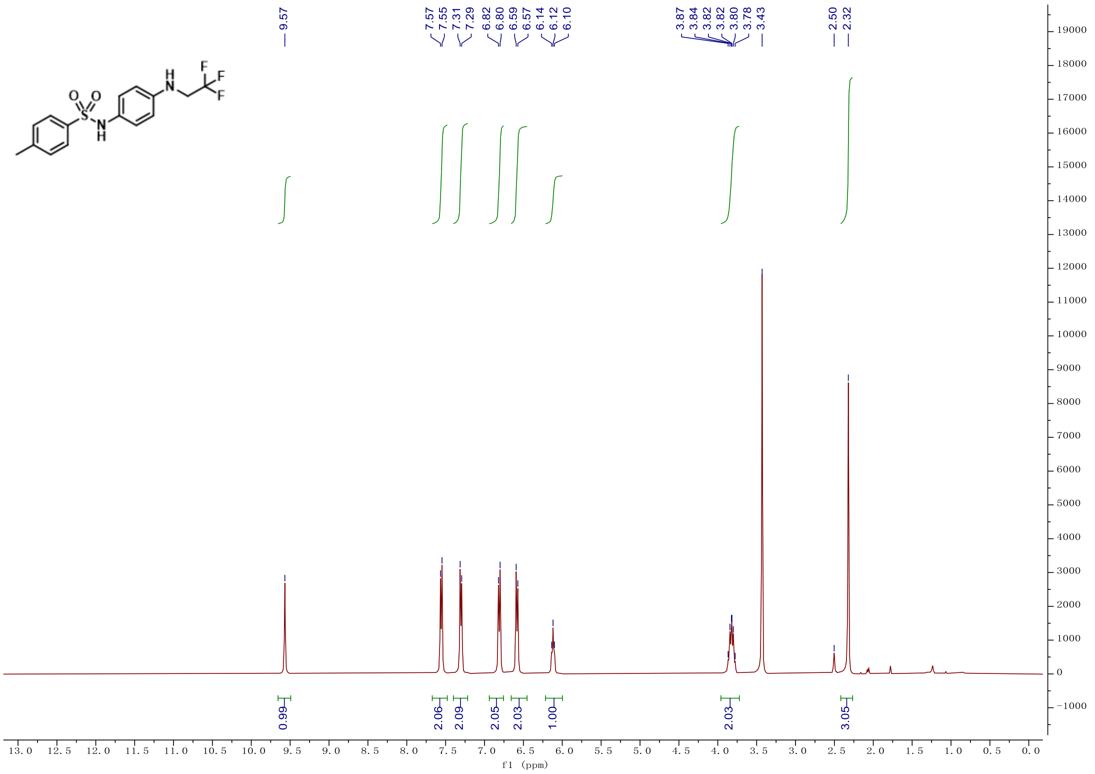

^{13}C NMR of Compound 20 (101 MHz, DMSO-d^{6})

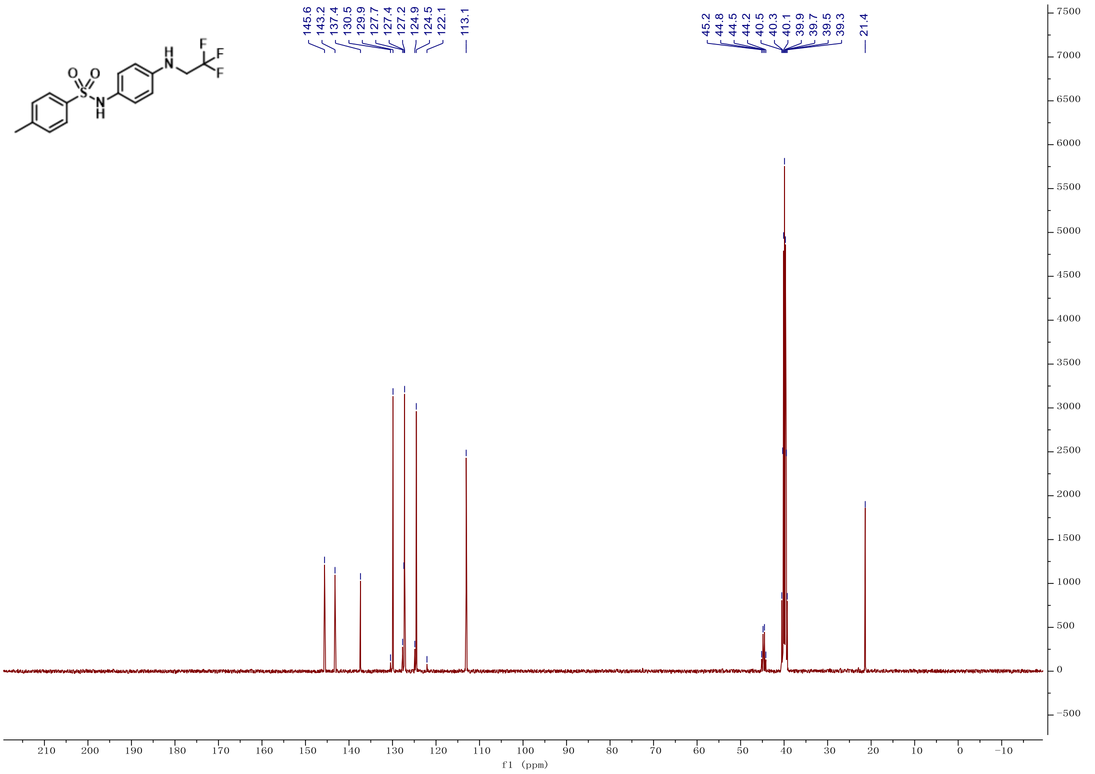

^{19}F NMR of Compound 20 (376 MHz, DMSO-d^6)

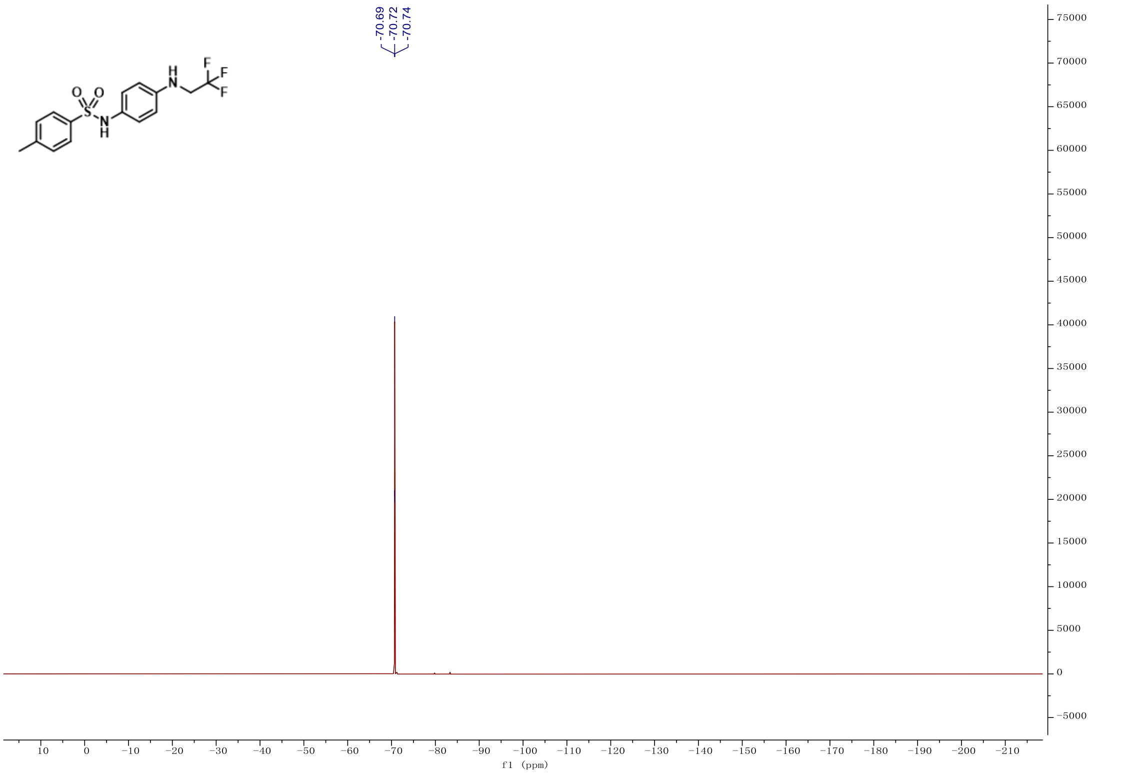

^{1}H NMR of Compound 21 (400 MHz, $CDCl_3$)

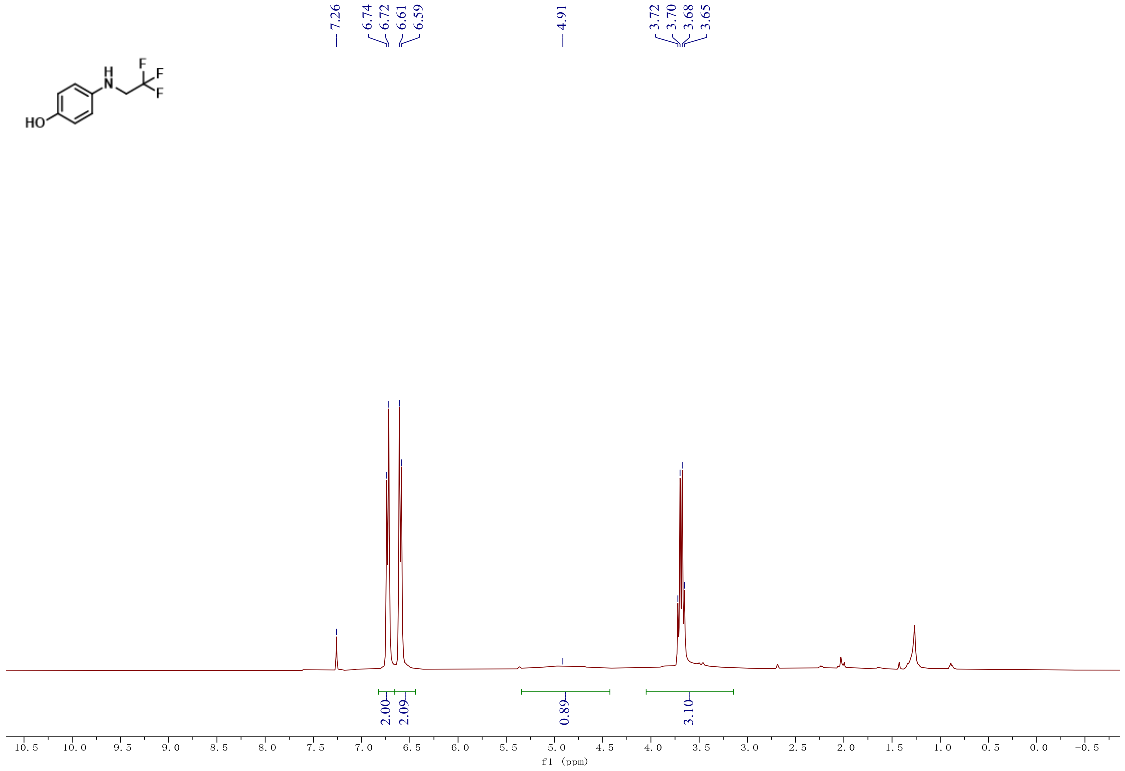

^{13}C NMR of Compound 21 (101 MHz, $CDCl_3$)

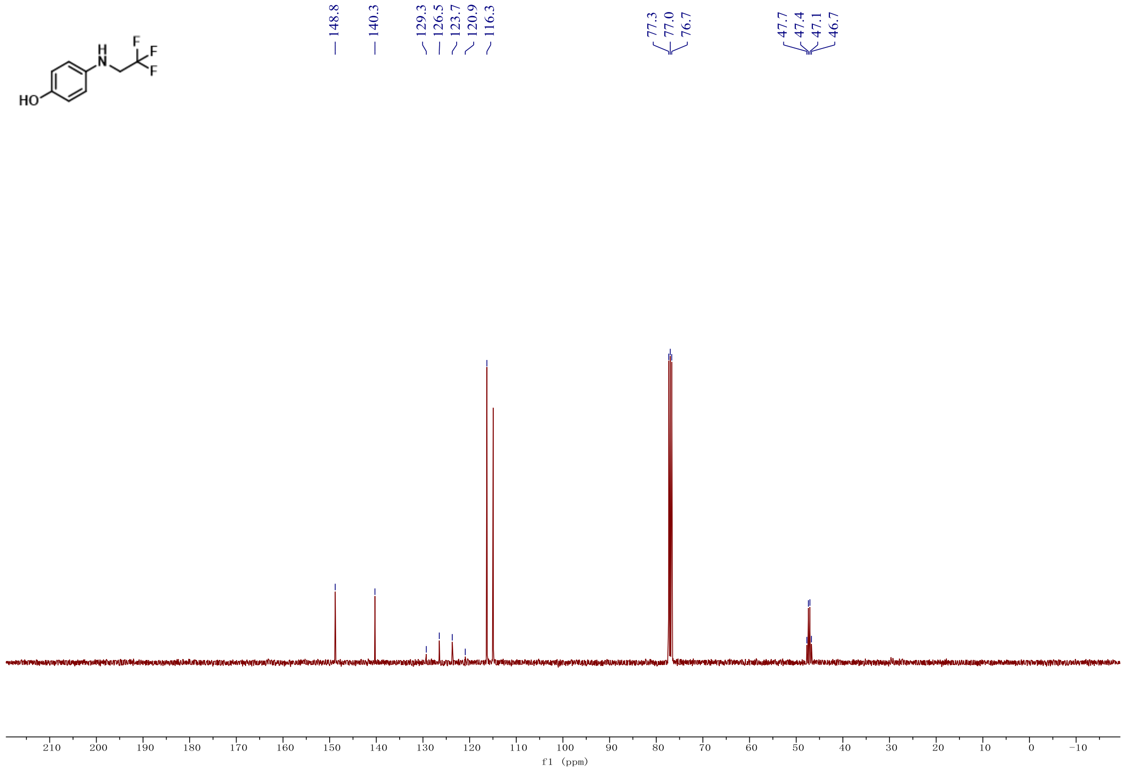

^{19}F NMR of Compound 21 (376 MHz, $CDCl_3$)

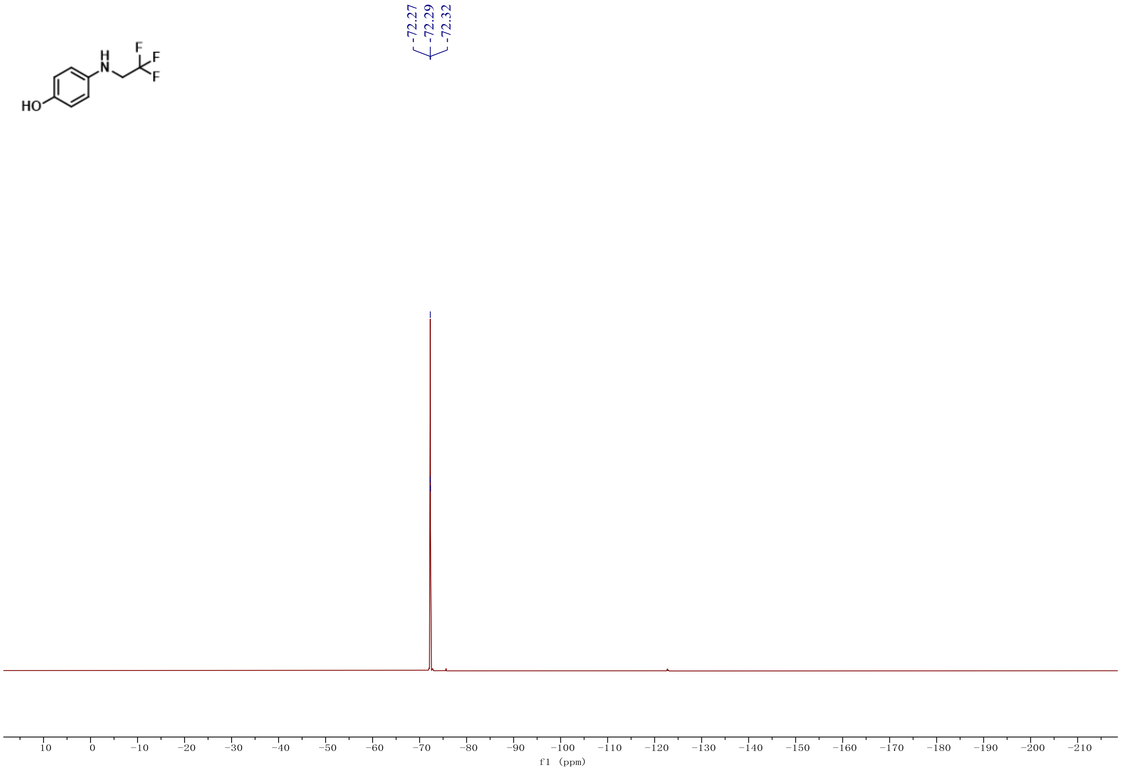

^{1}H NMR of Compound 22 (400 MHz, CD_3OD)

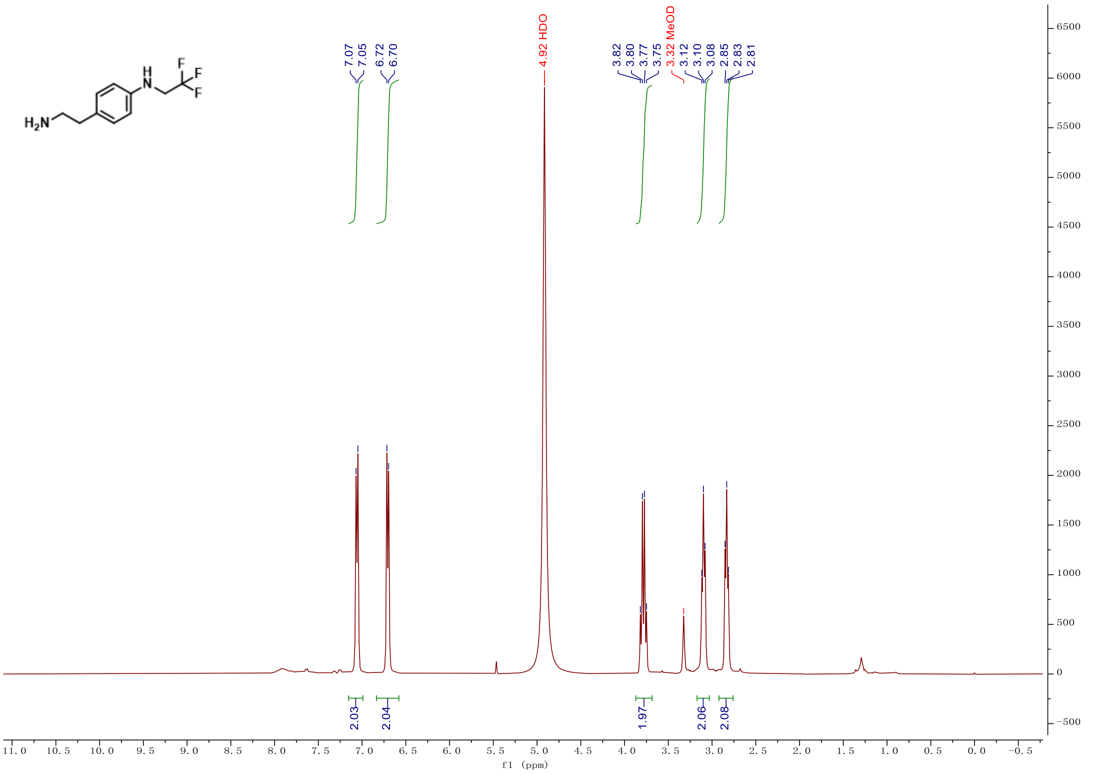

^{13}C NMR of Compound 22 (101 MHz, CD_3OD)

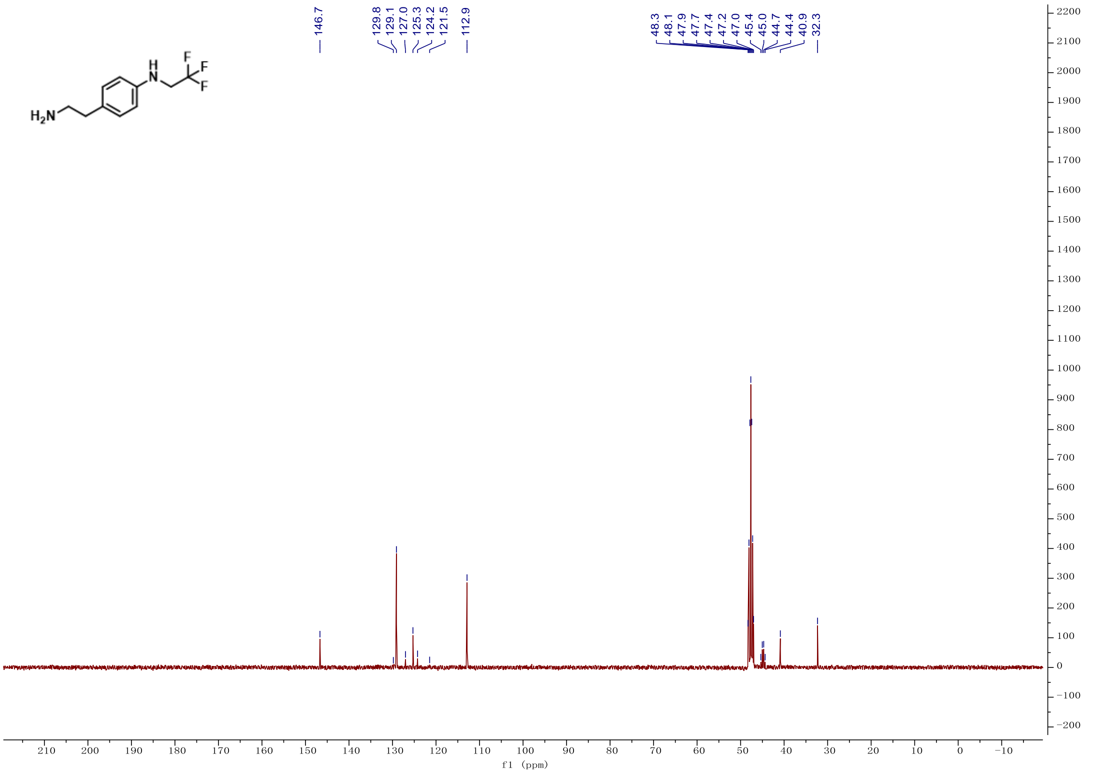

^{19}F NMR of Compound 22 (376 MHz, CD_3OD)

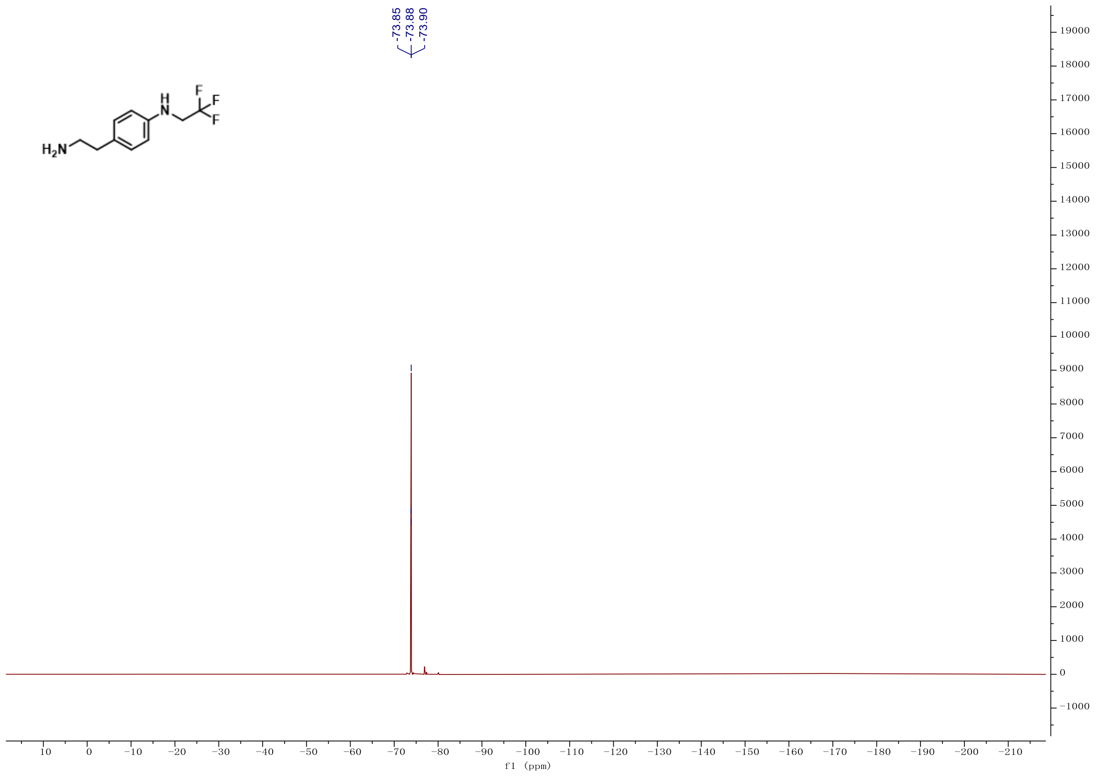

^{1}H NMR of Compound 23 (400 MHz, $CDCl_3$)

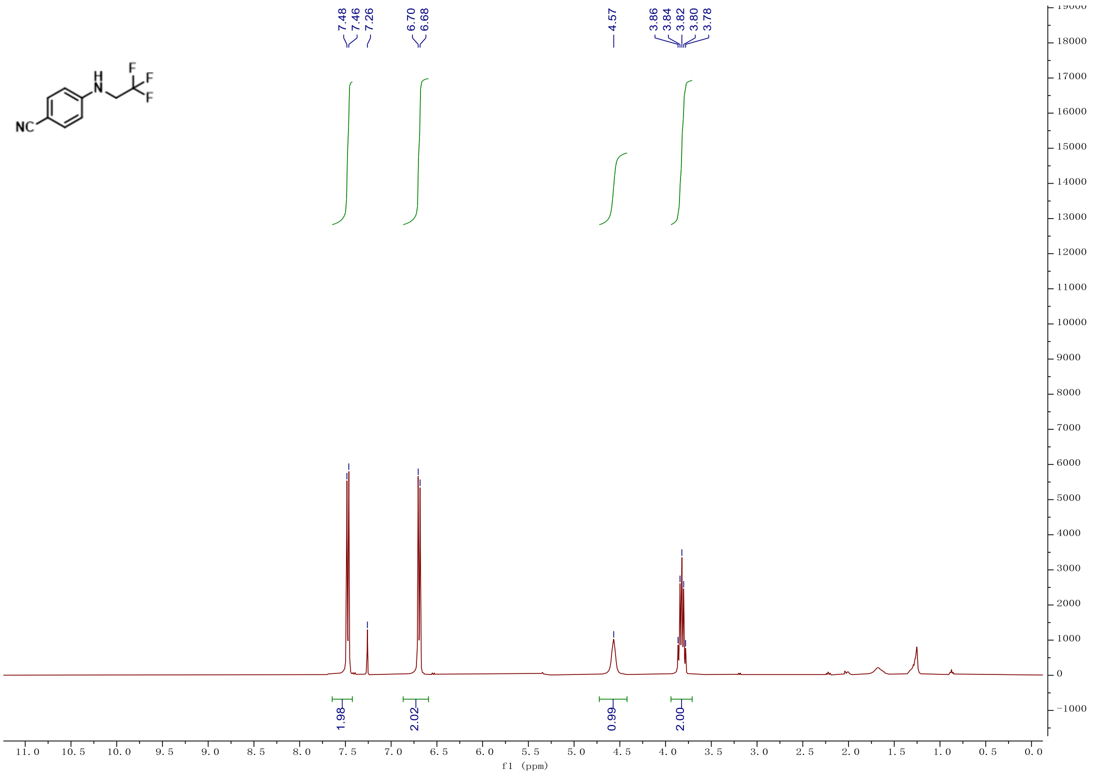

^{13}C NMR of Compound 23 (101 MHz, $CDCl_3$)

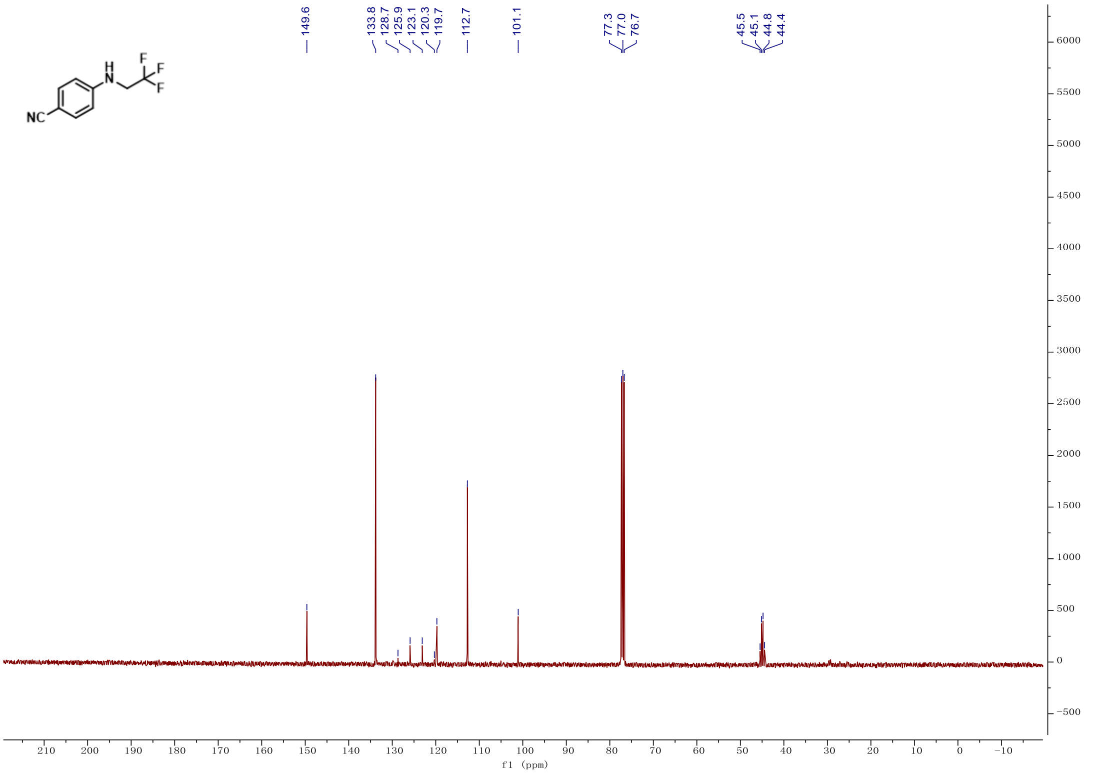

^{19}F NMR of Compound 23 (376 MHz, $CDCl_3$)

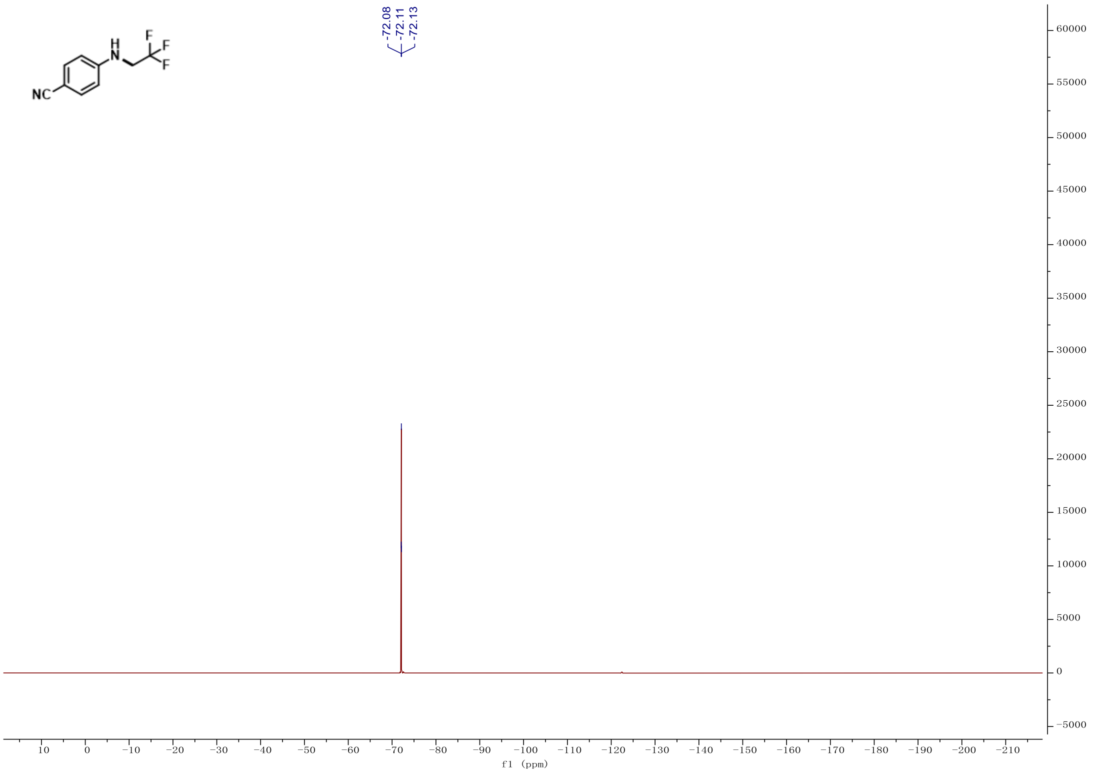

^{1}H NMR of Compound 24 (400 MHz, DMSO-d^{6})

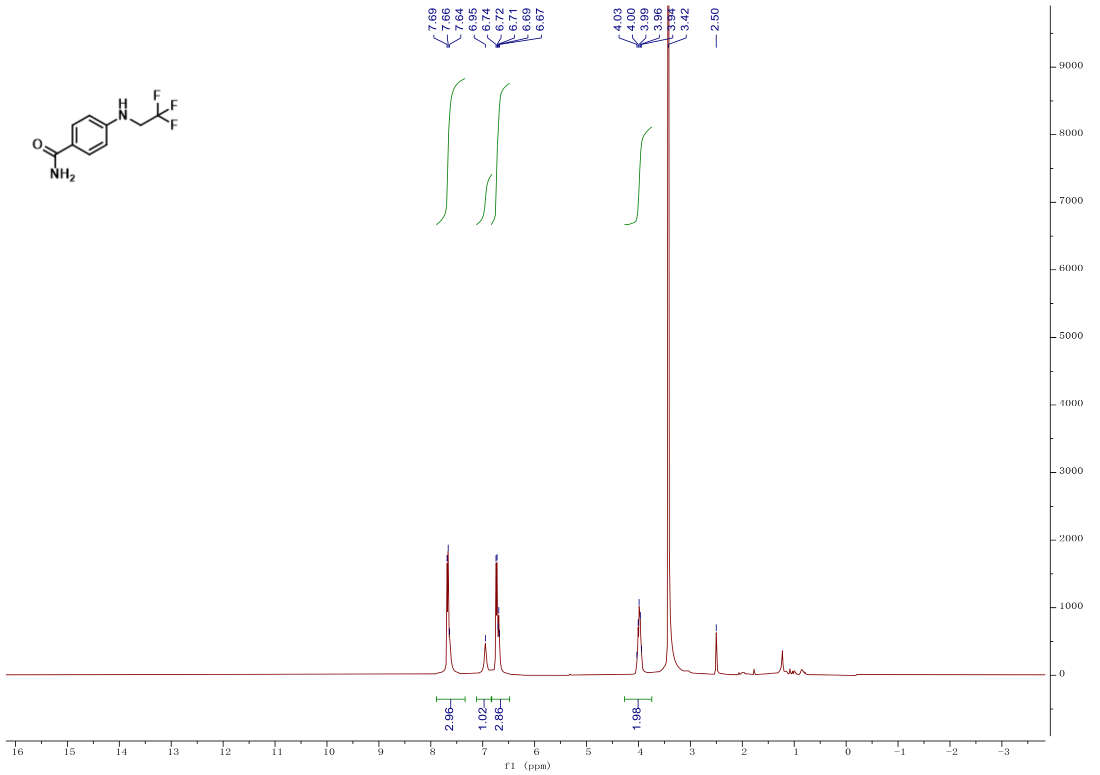

^{13}C NMR of Compound 24 (101 MHz, DMSO-d^{6})

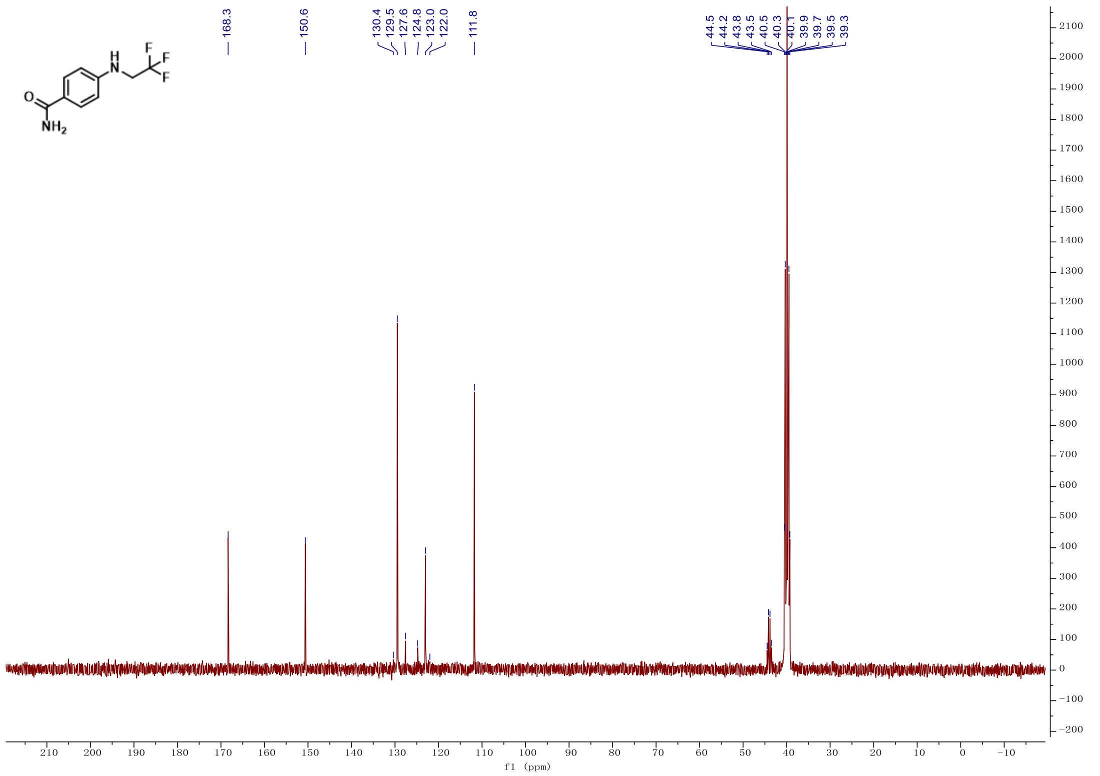

^{19}F NMR of Compound 24 (376 MHz, DMSO-d^6)

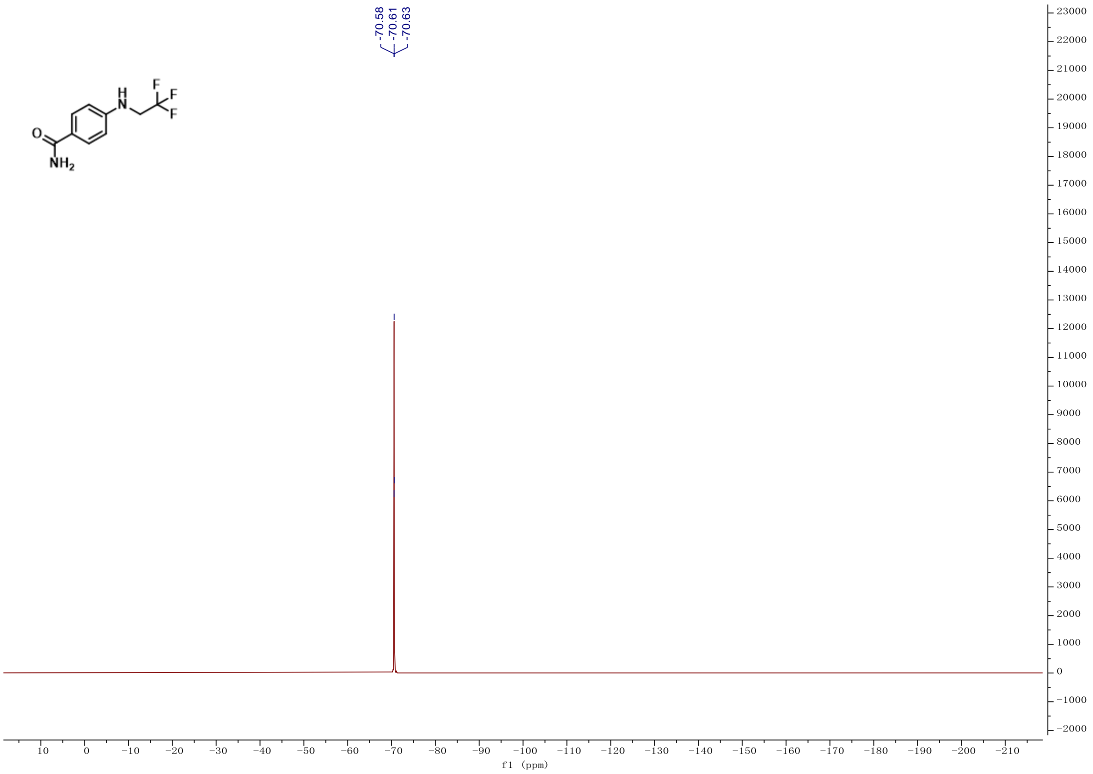

^{1}H NMR of Compound 25 (400 MHz, $CDCl_3$)

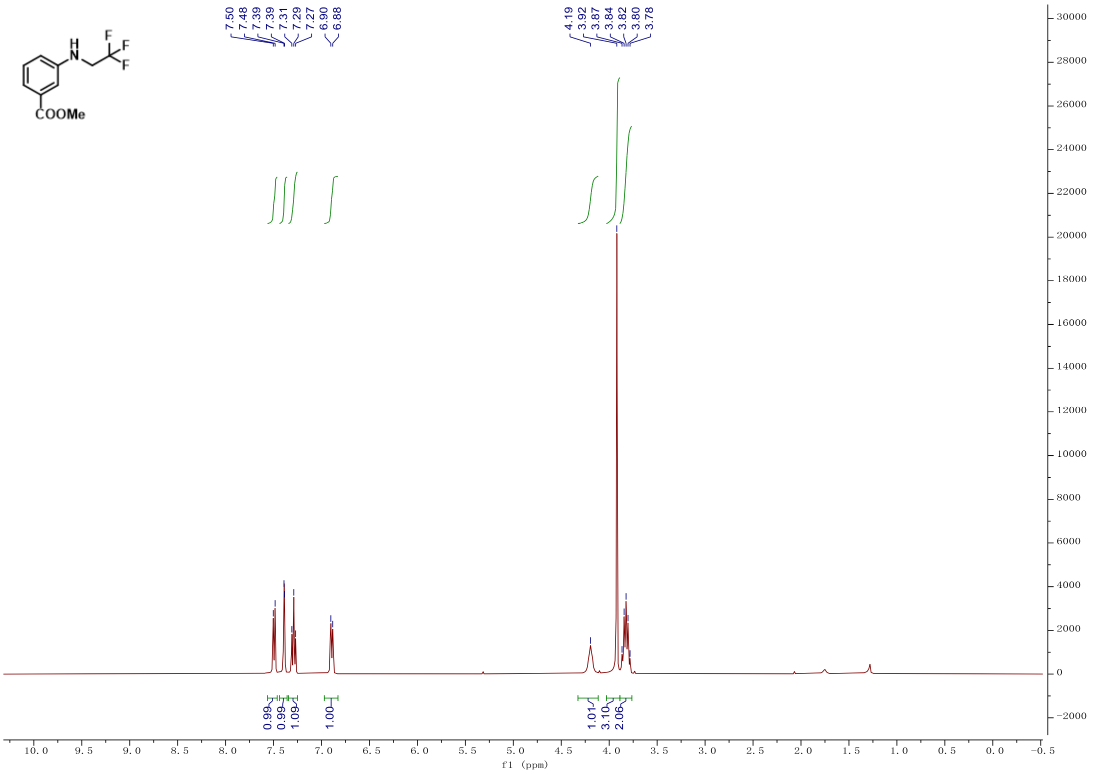

^{13}C NMR of Compound 25 (101 MHz, $CDCl_3$)

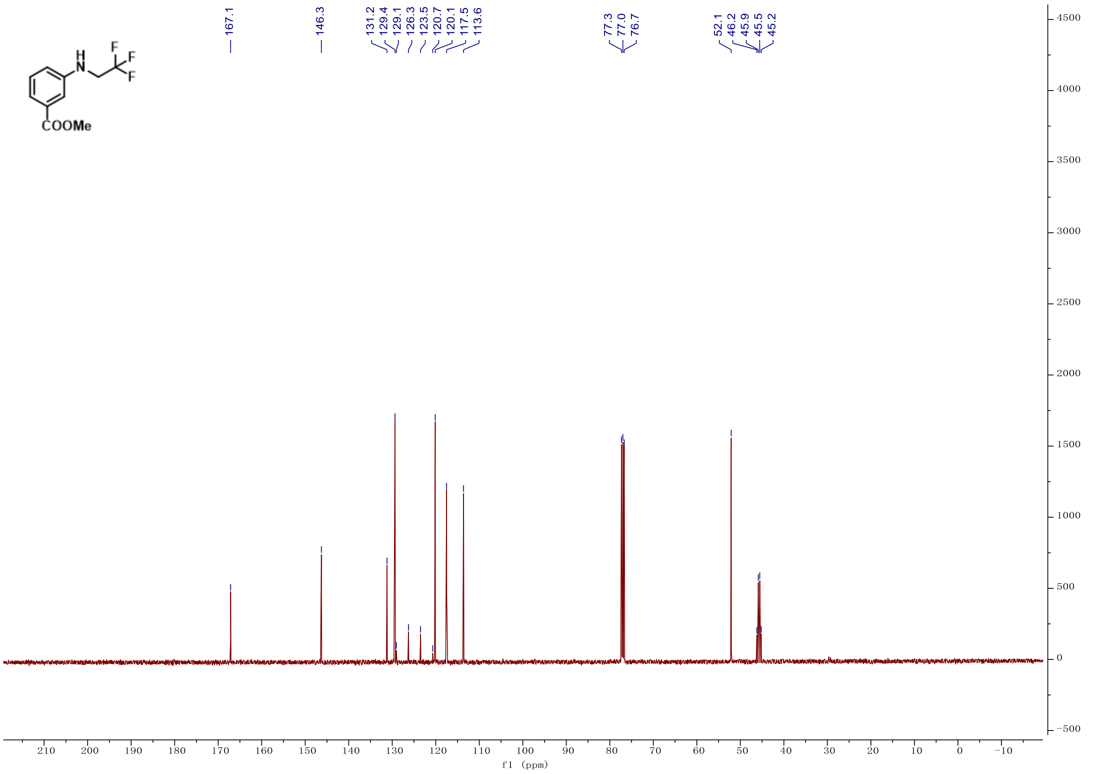

^{19}F NMR of Compound 25 (376 MHz, $CDCl_3$)

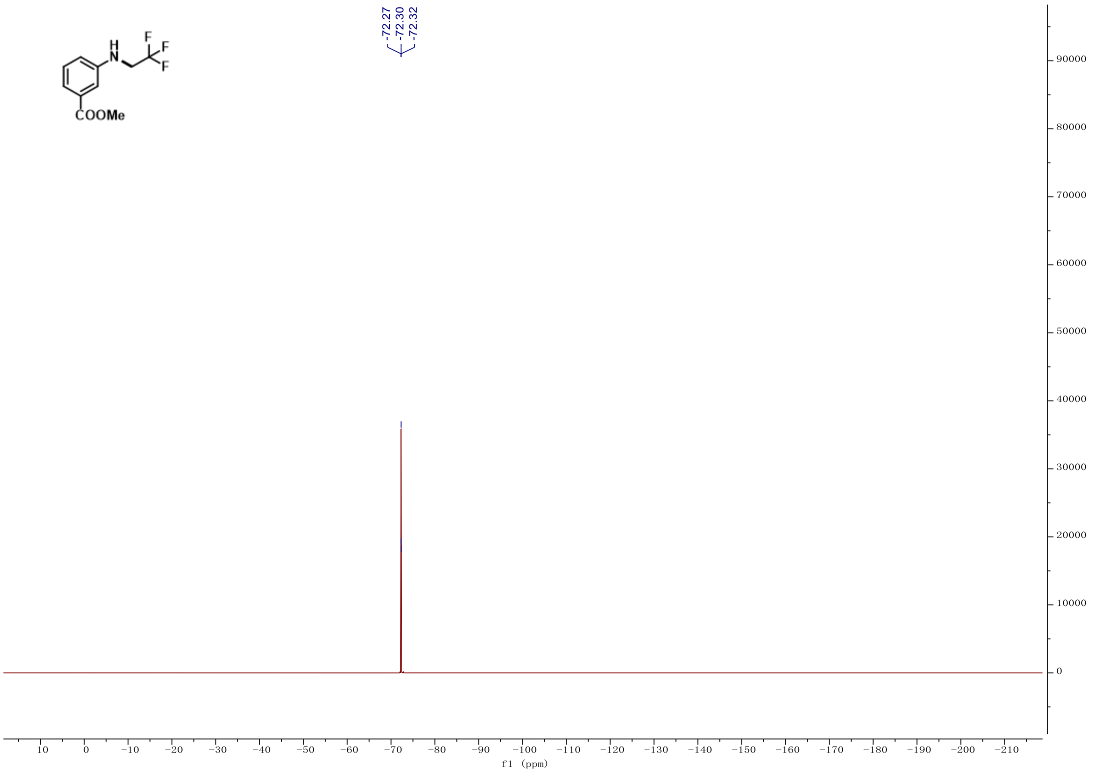

^{1}H NMR of Compound 26 (400 MHz, DMSO-d^{6})

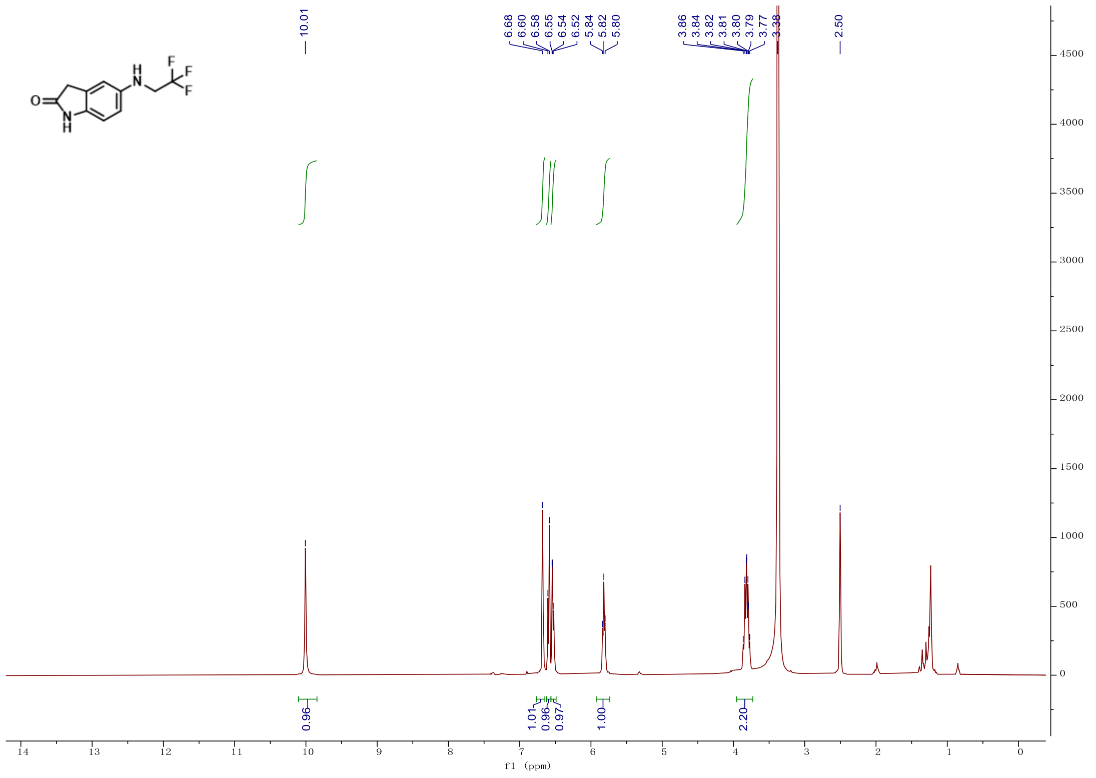

^{13}C NMR of Compound 26 (101 MHz, DMSO-d^{6})

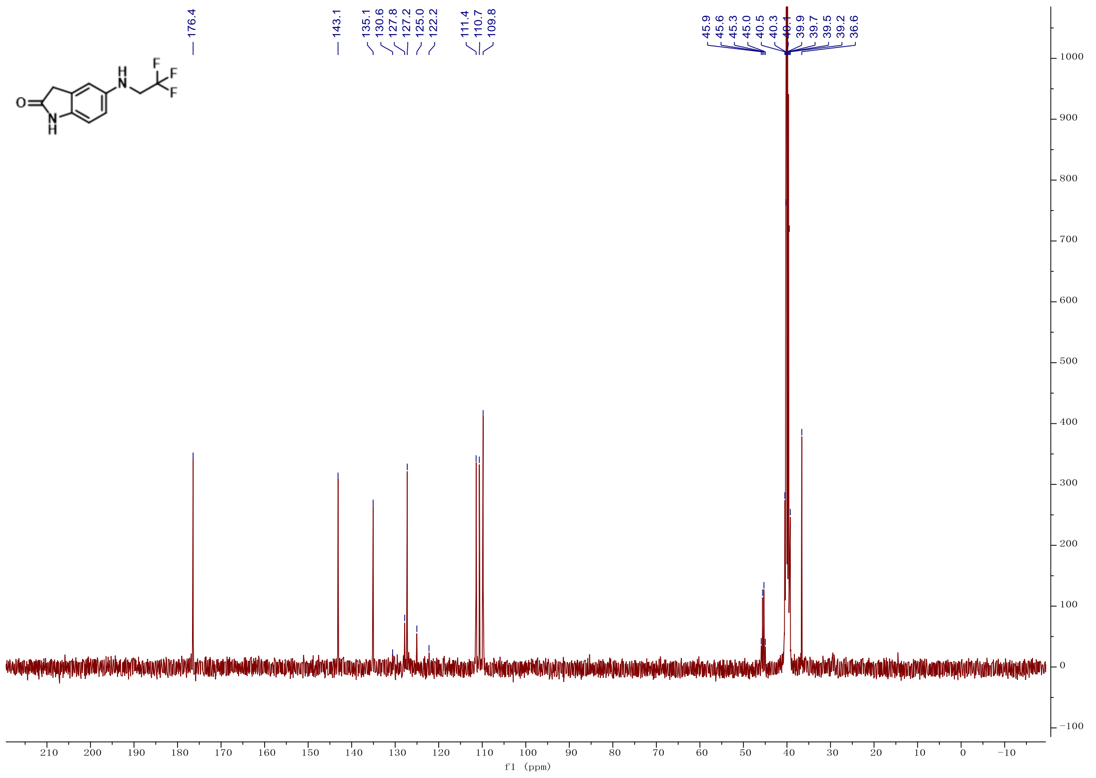

^{19}F NMR of Compound 26 (376 MHz, DMSO-d^{6})

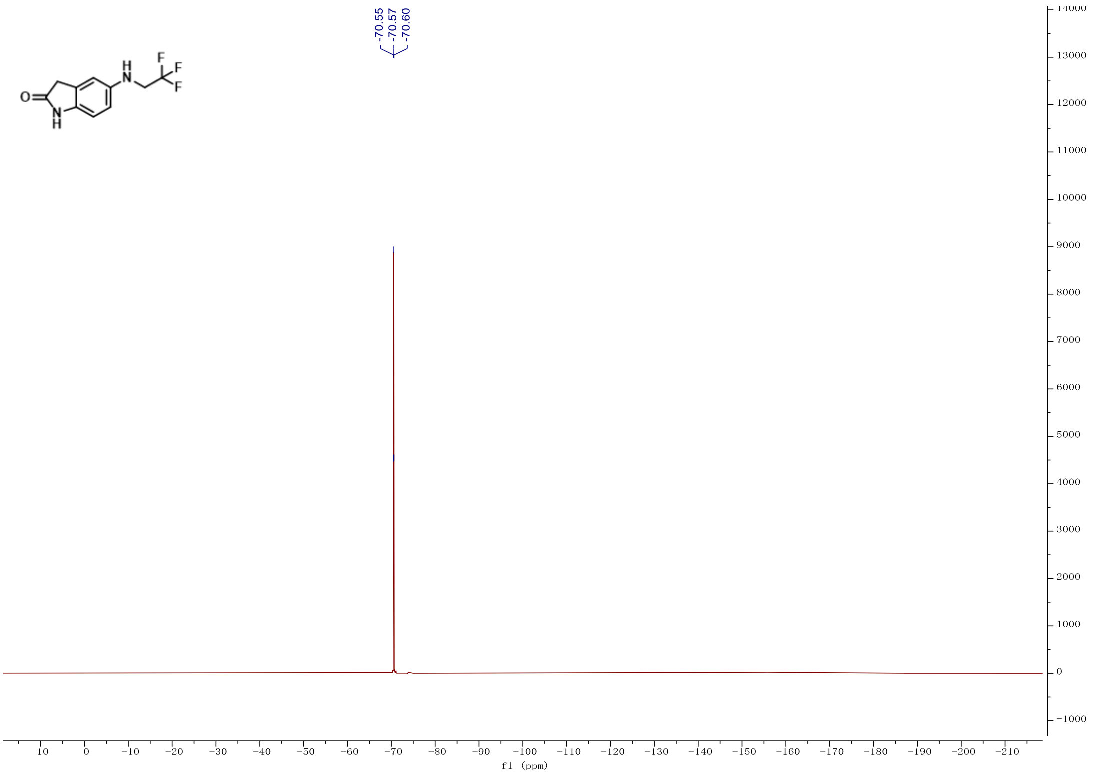

^{1}H NMR of Compound 27 (400 MHz, DMSO-d^{6})

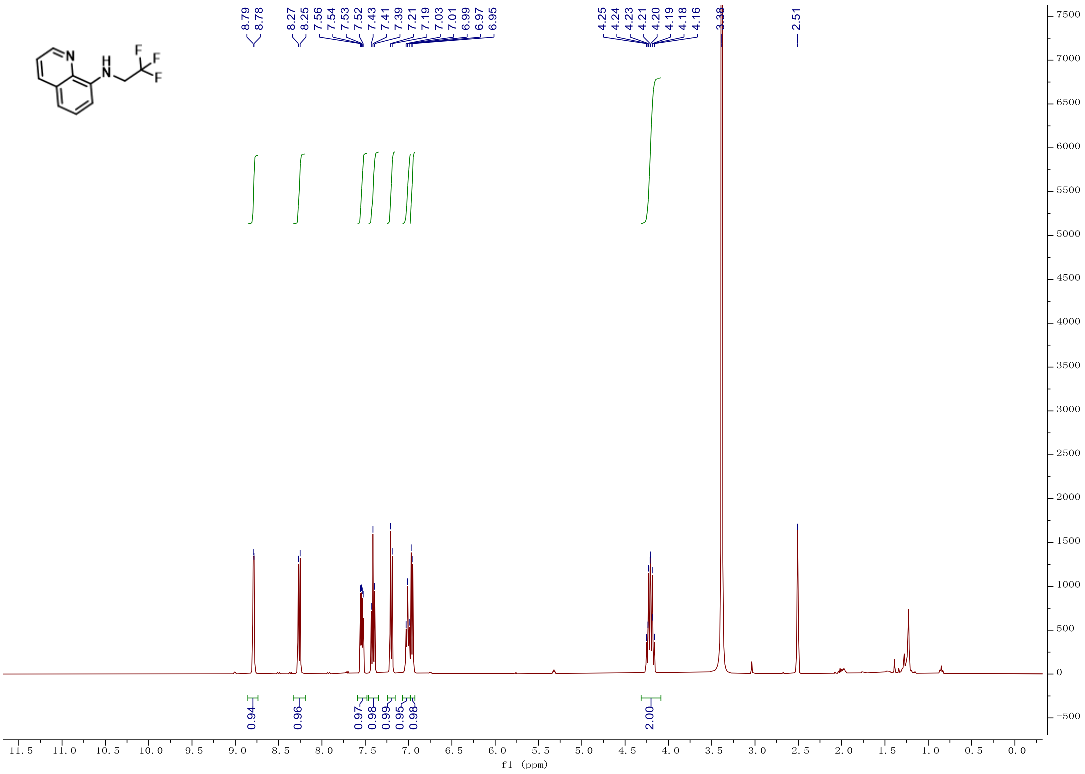

^{13}C NMR of Compound 27 (101 MHz, DMSO-d^{6})

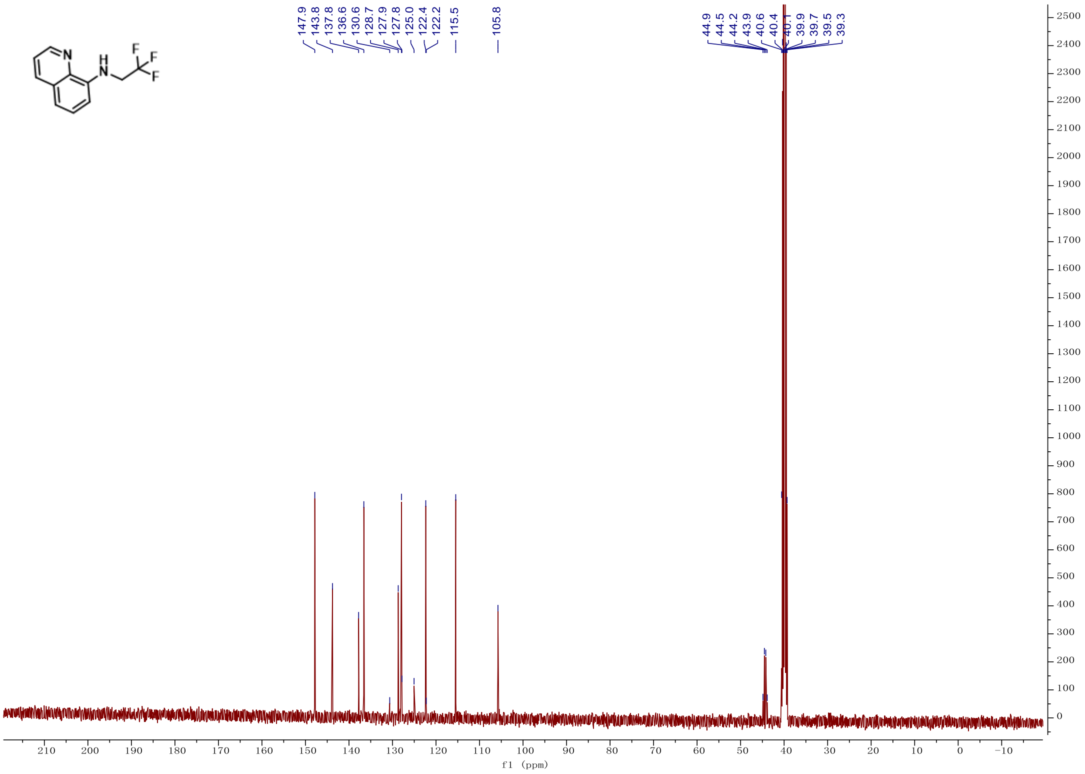

^{19}F NMR of Compound 27 (376 MHz, DMSO-d^6)

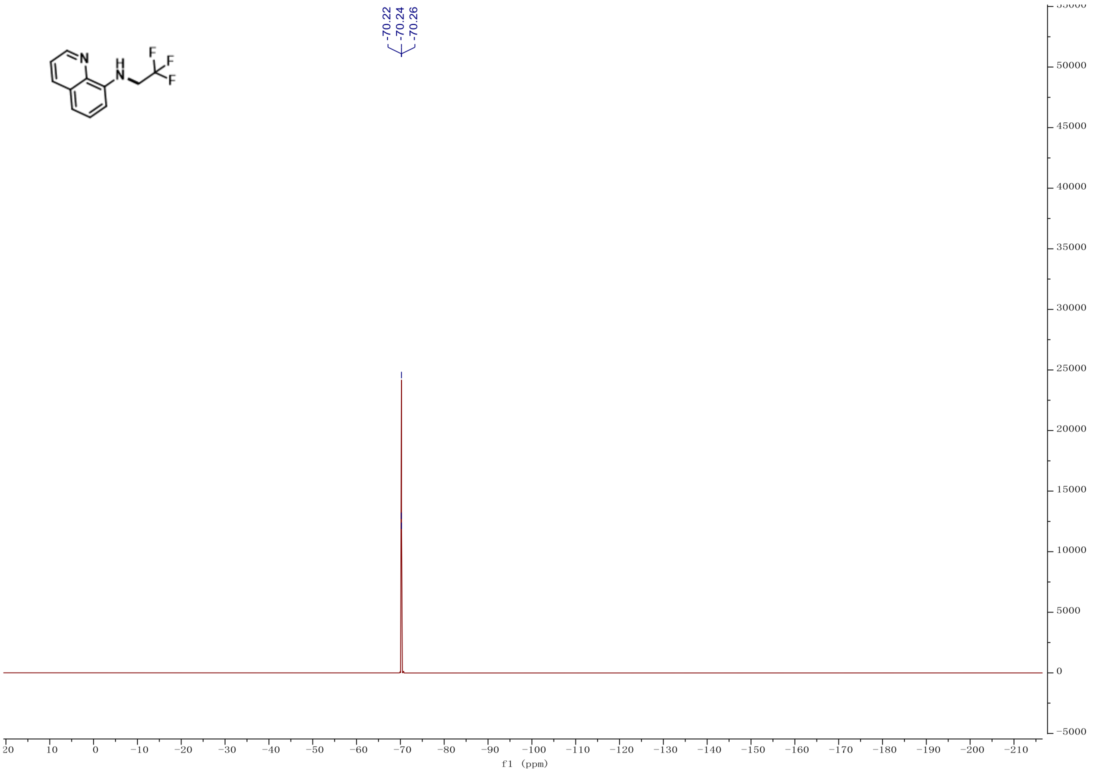

^{1}H NMR of Compound 28 (400 MHz, $CDCl_3$)

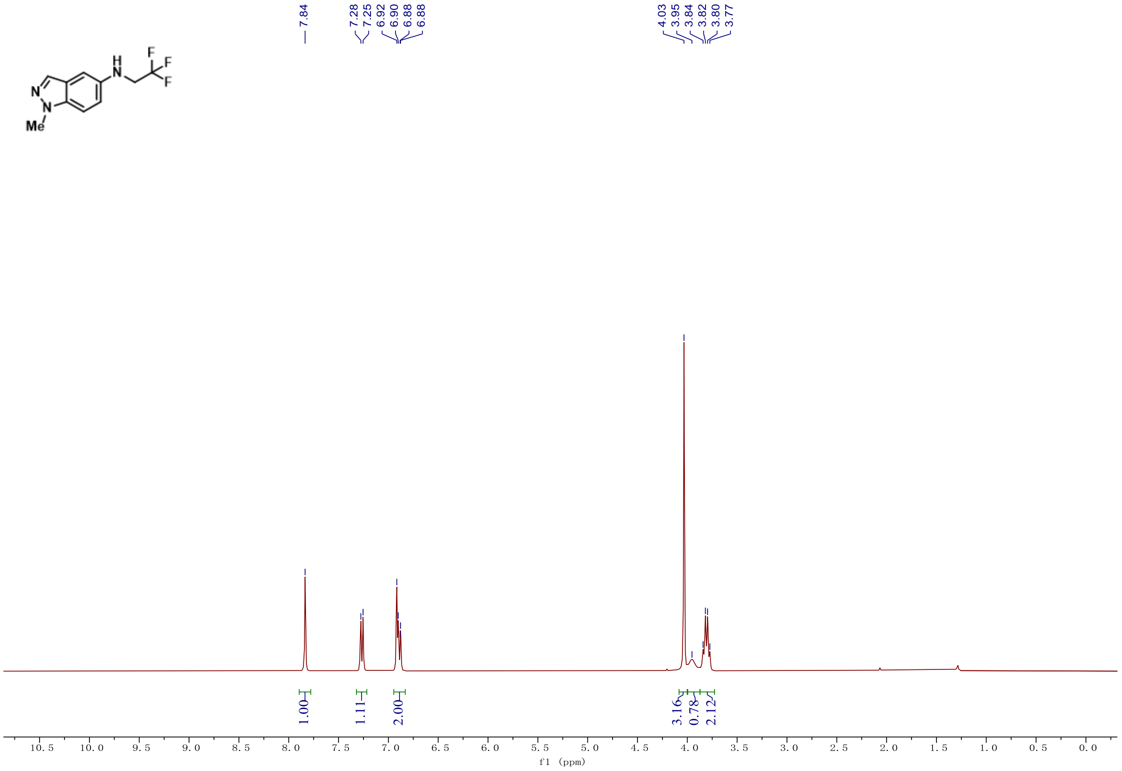

^{13}C NMR of Compound 28 (101 MHz, $CDCl_3$)

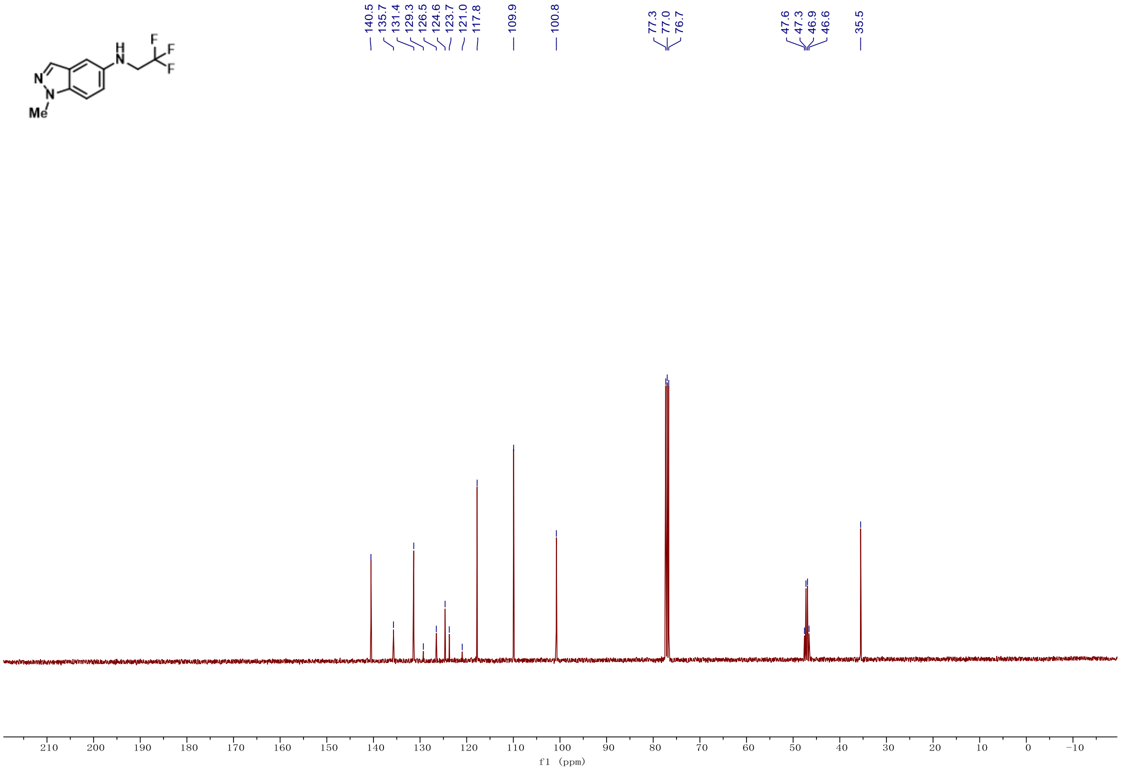

^{19}F NMR of Compound 28 (376 MHz, $CDCl_3$)

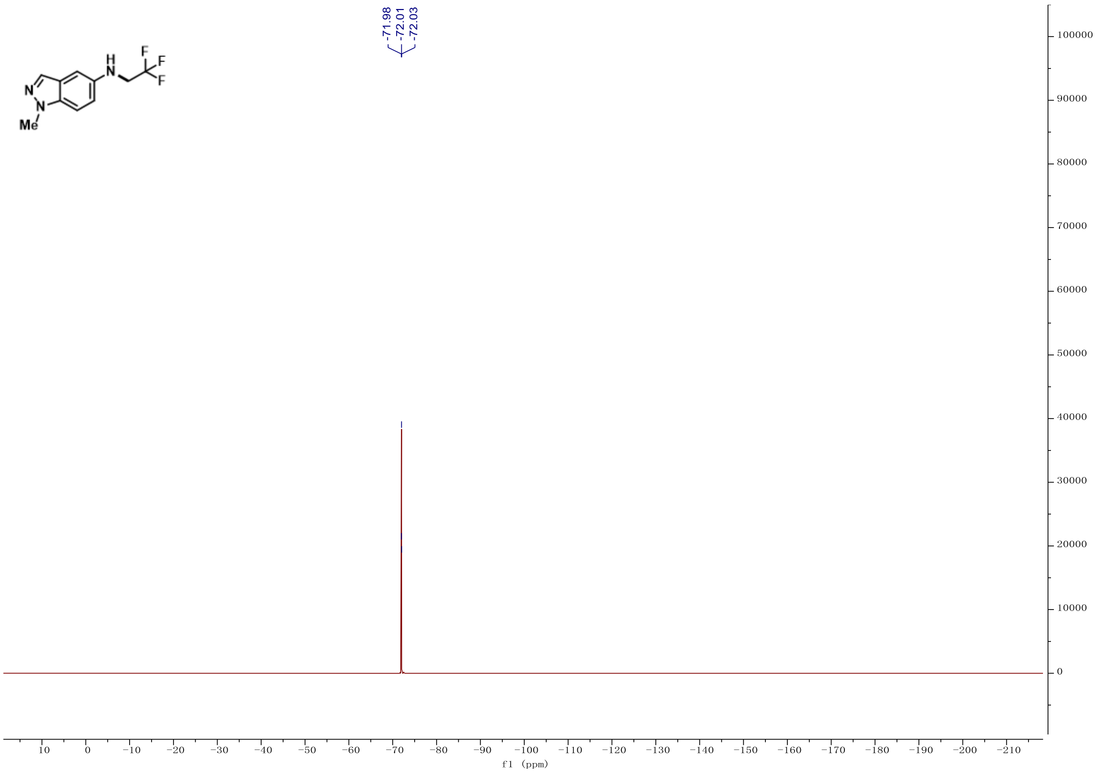

^{1}H NMR of Compound 29 (400 MHz, $CDCl_3$)

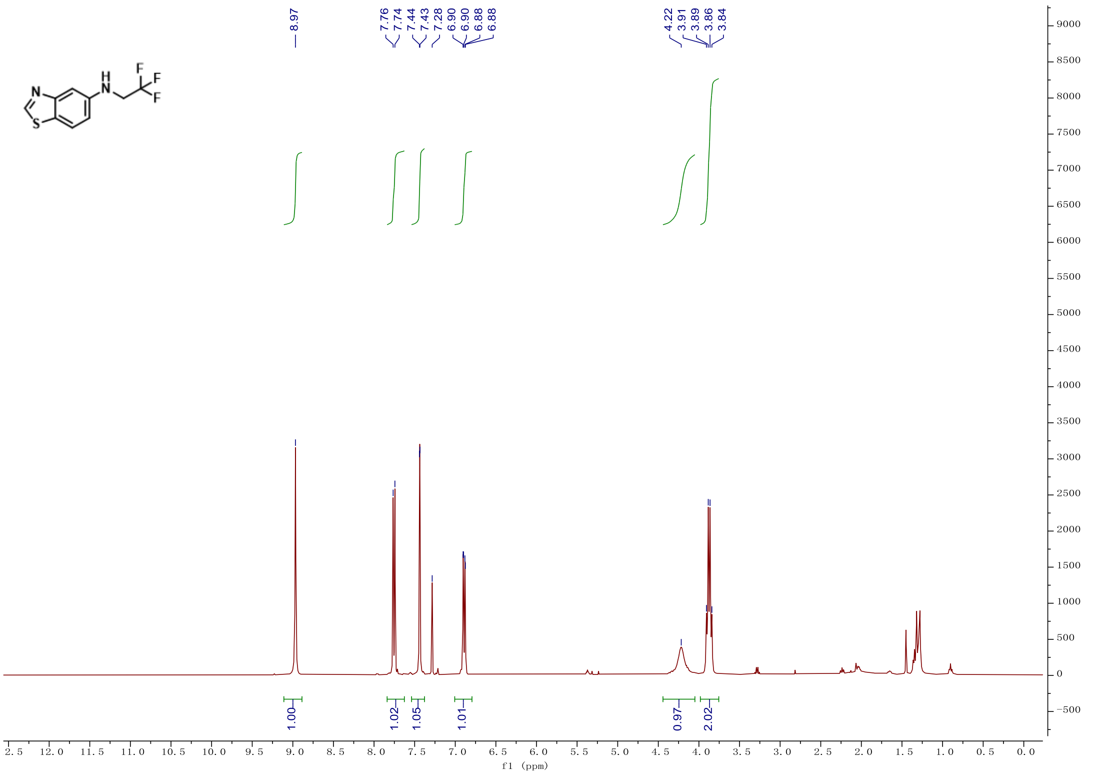

^{13}C NMR of Compound 29 (101 MHz, $CDCl_3$)

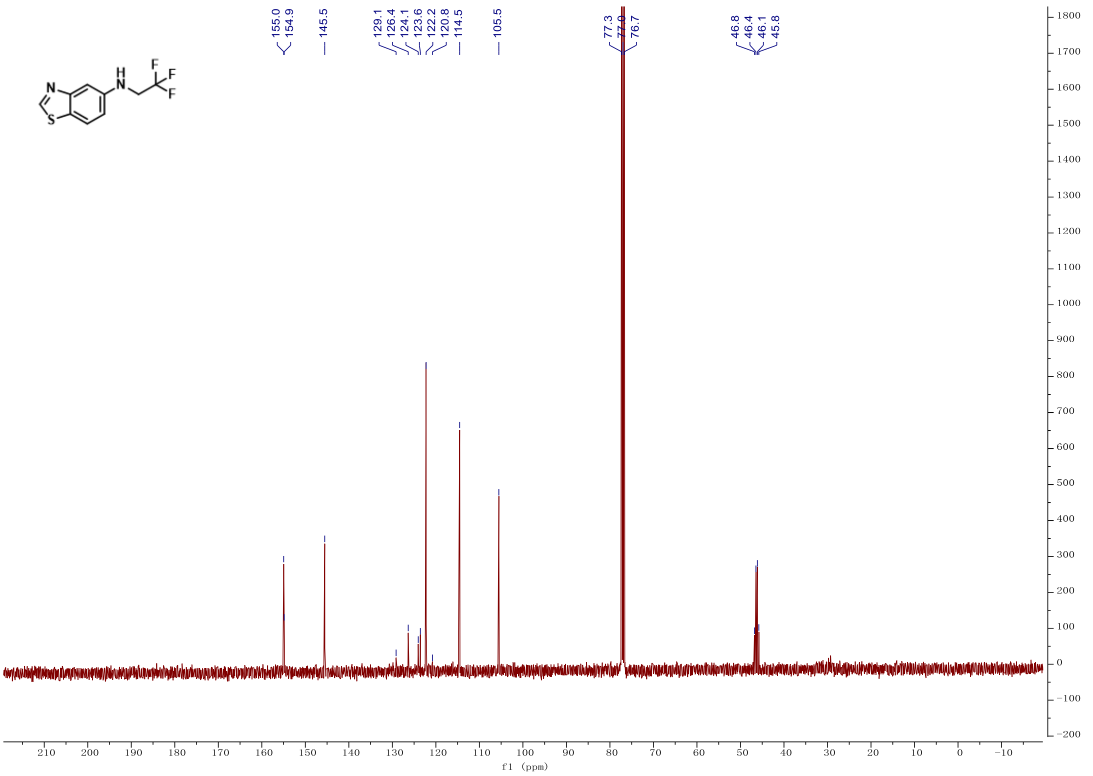

^{19}F NMR of Compound 29 (376 MHz, $CDCl_3$)

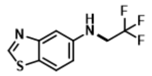

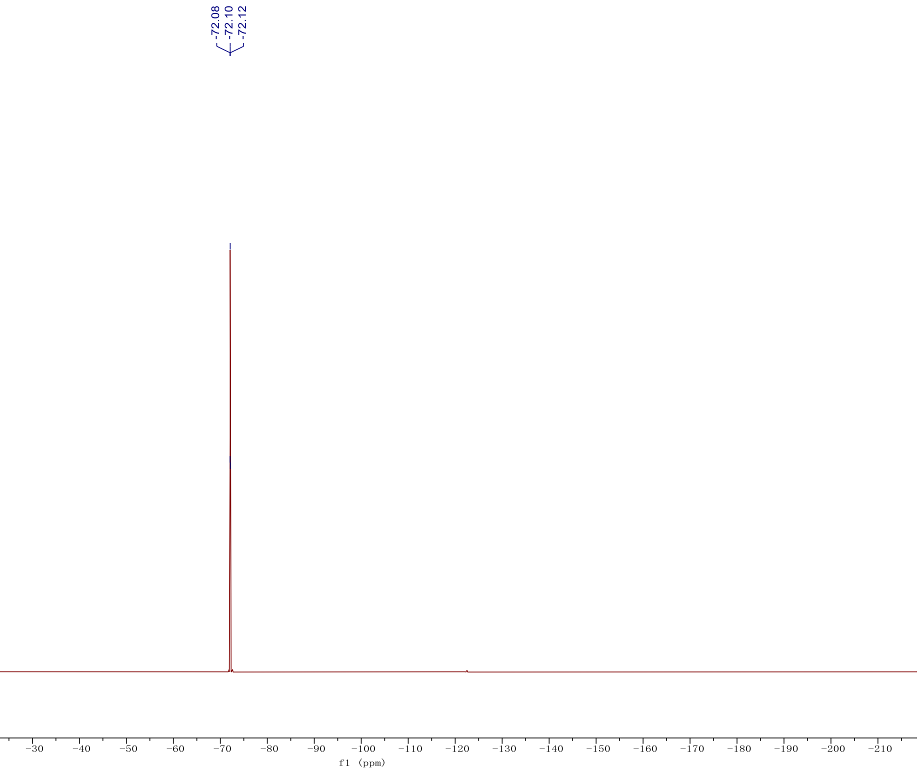

^{1}H NMR of Compound 30 (400 MHz, $CDCl_3$)

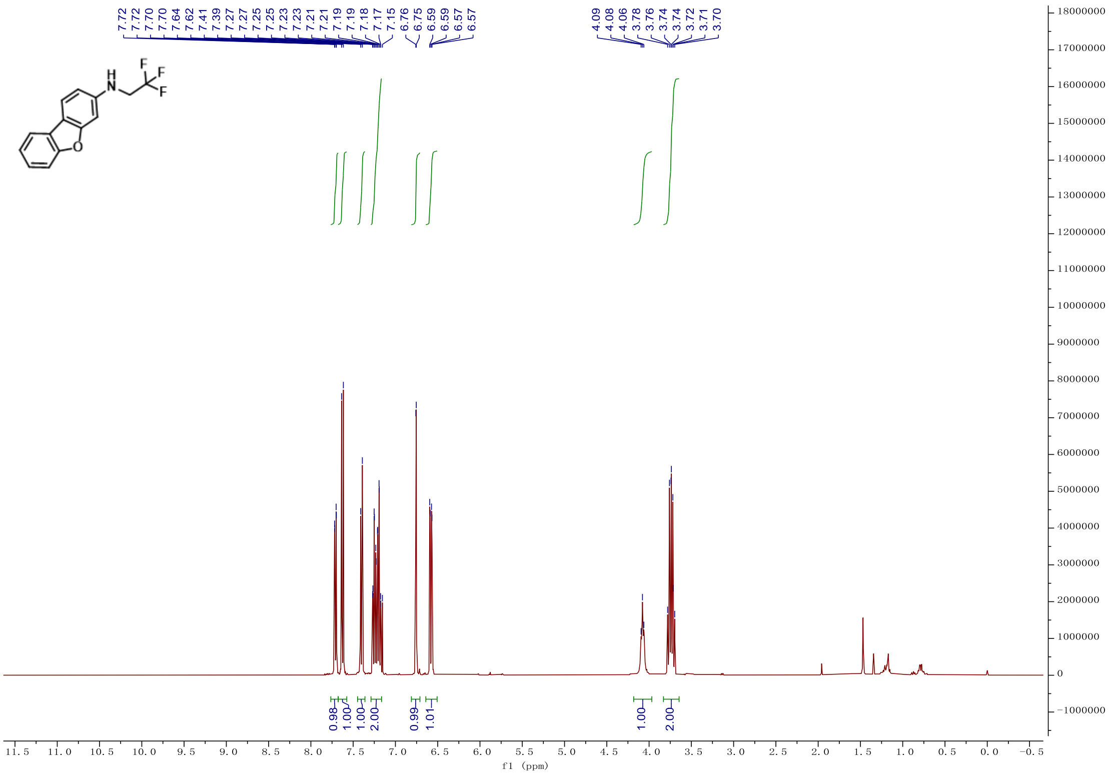

^{13}C NMR of Compound 30 (101 MHz, $CDCl_3$)

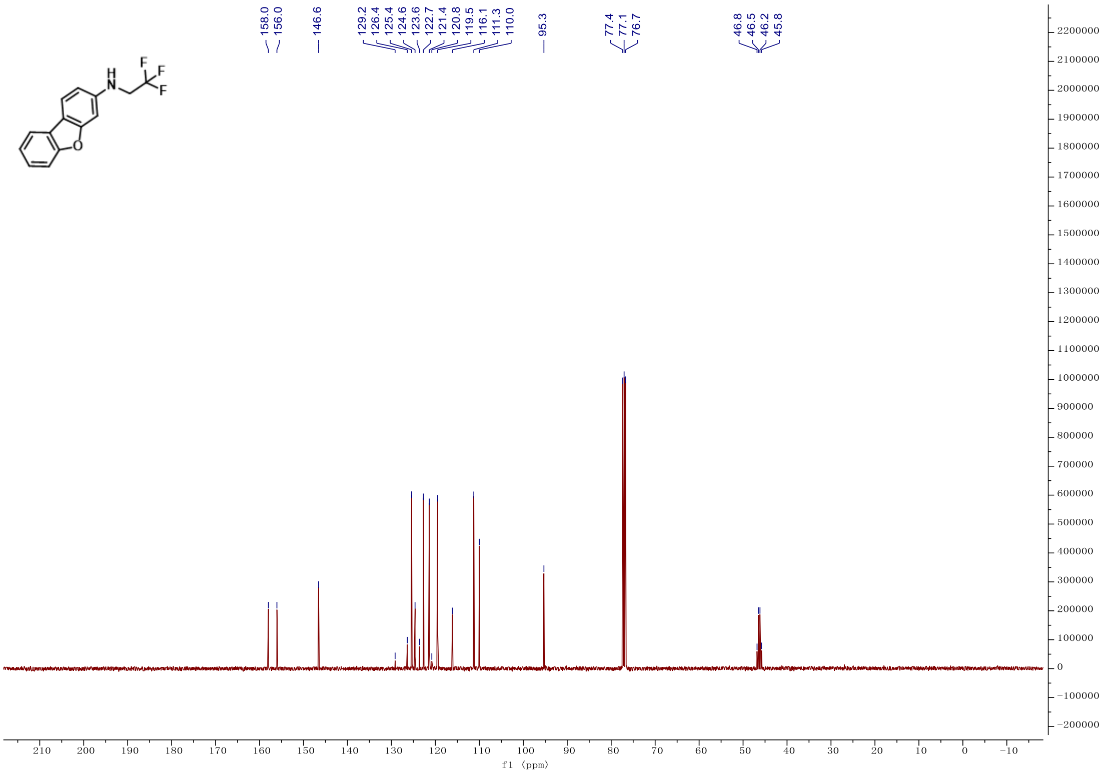

^{19}F NMR of Compound 30 (376 MHz, $CDCl_3$)

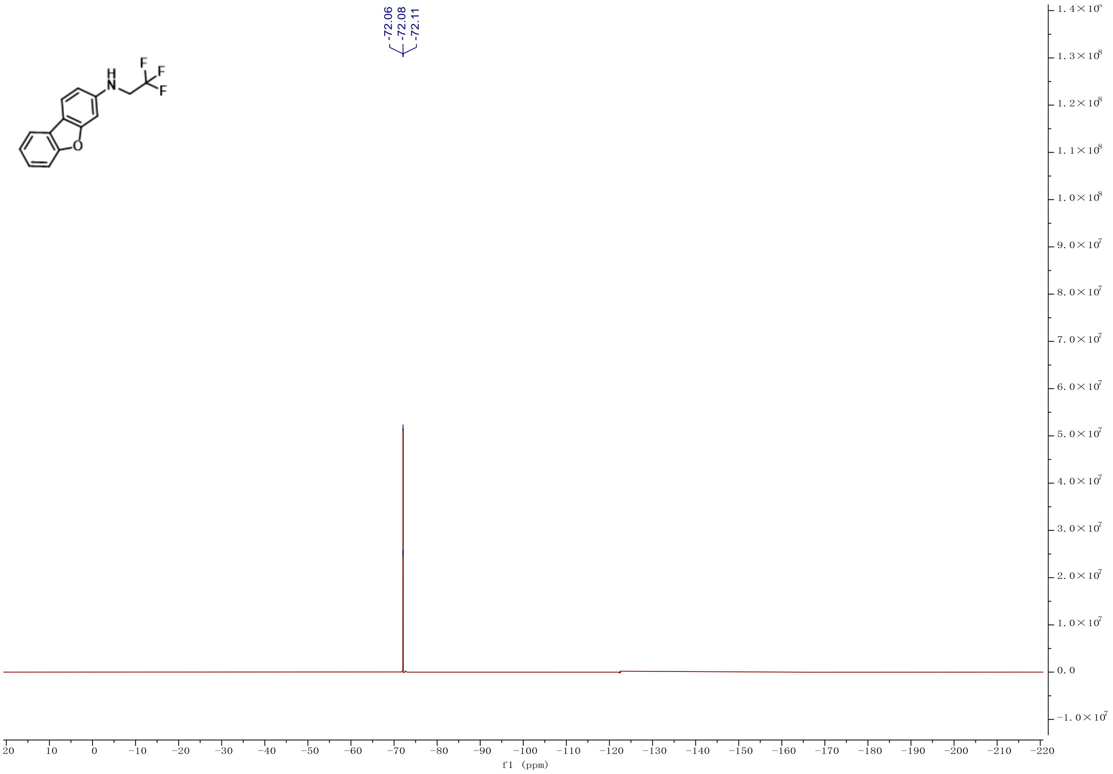

^{1}H NMR of Compound 31 (400 MHz, DMSO-d^{6})

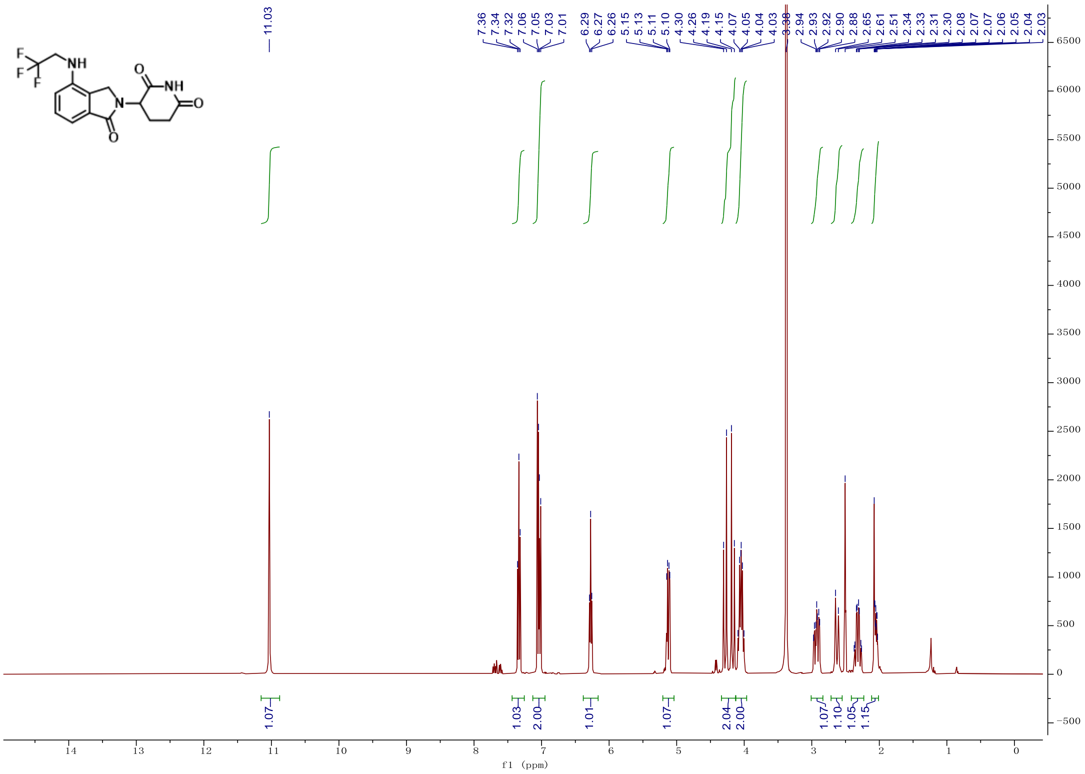

^{13}C NMR of Compound 31 (101 MHz, DMSO-d^{6})

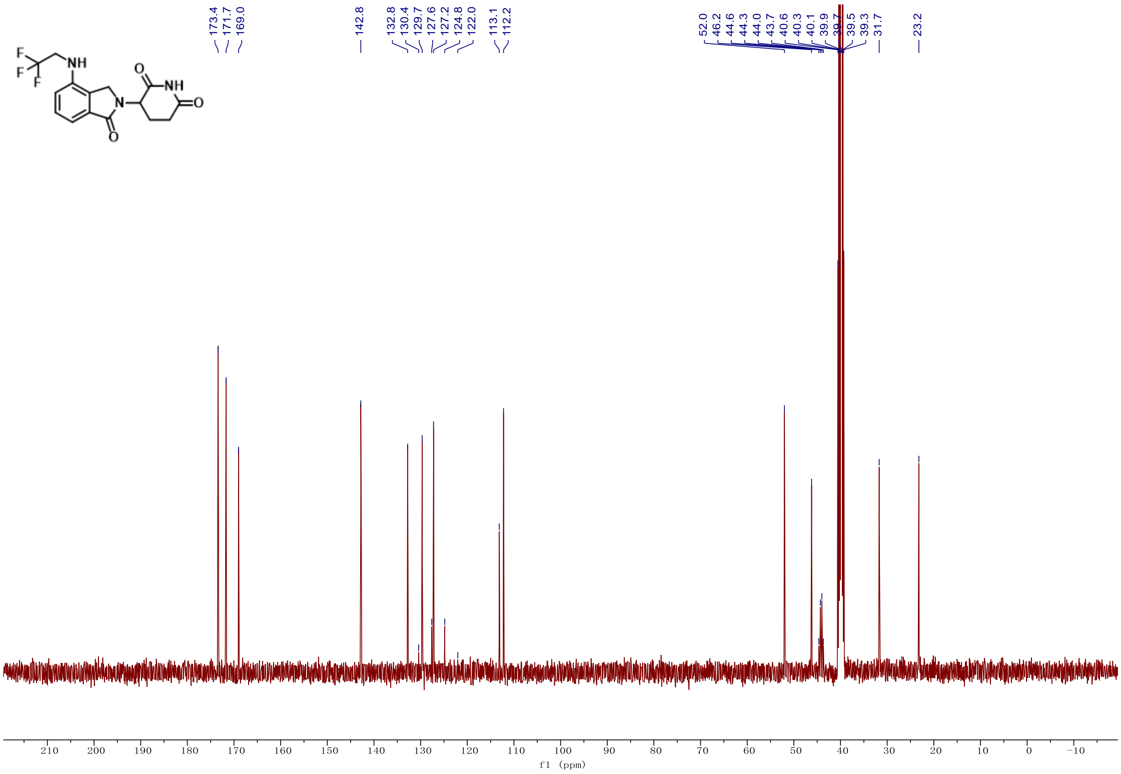

^{19}F NMR of Compound 31 (376 MHz, DMSO-d^{6})

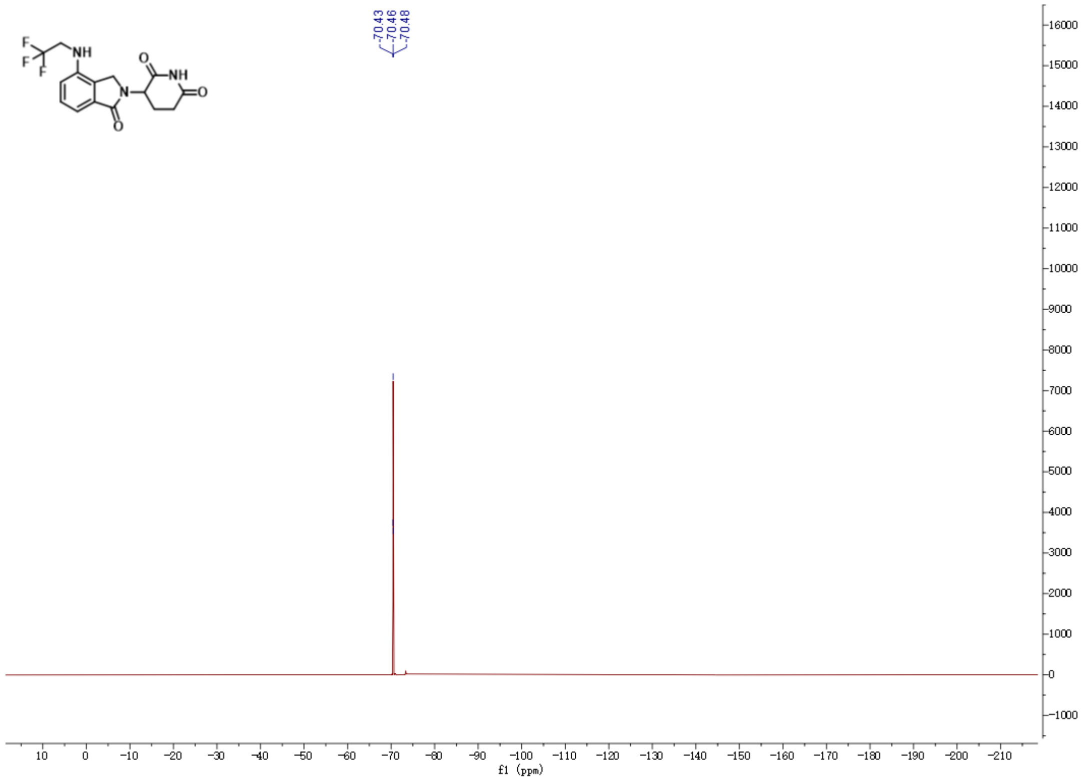

^{1}H NMR of Compound 32 (400 MHz, $CDCl_3$)

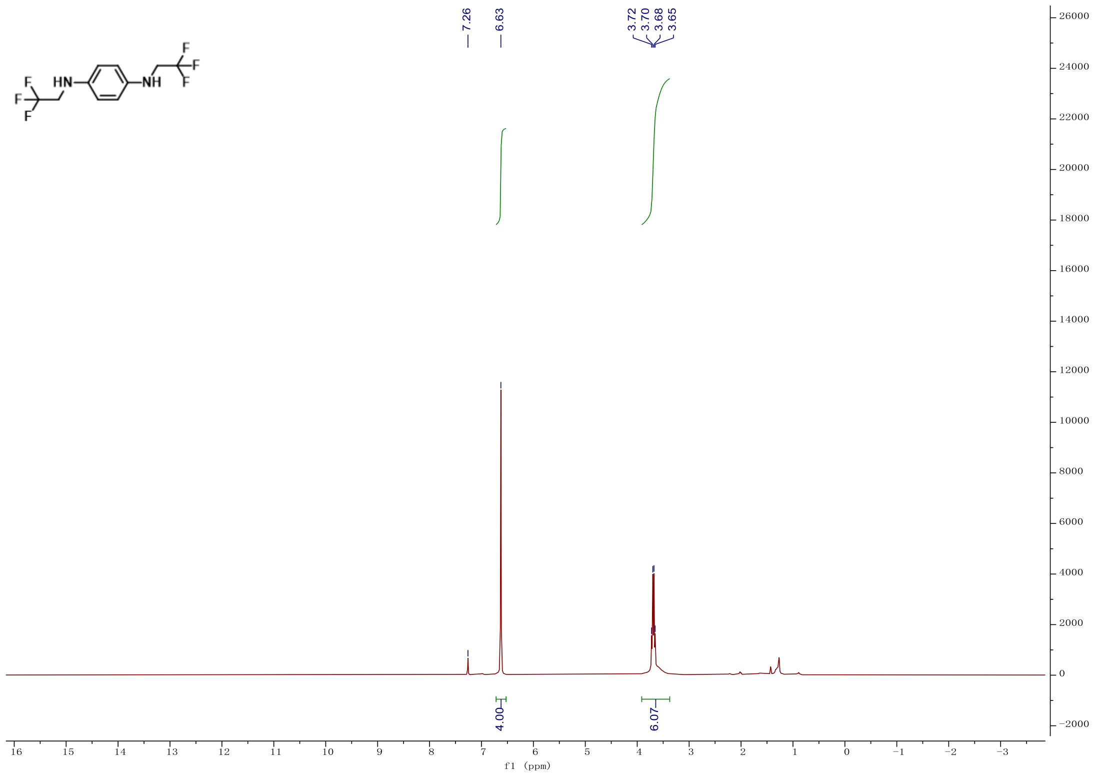

^{13}C NMR of Compound 32 (101 MHz, $CDCl_3$)

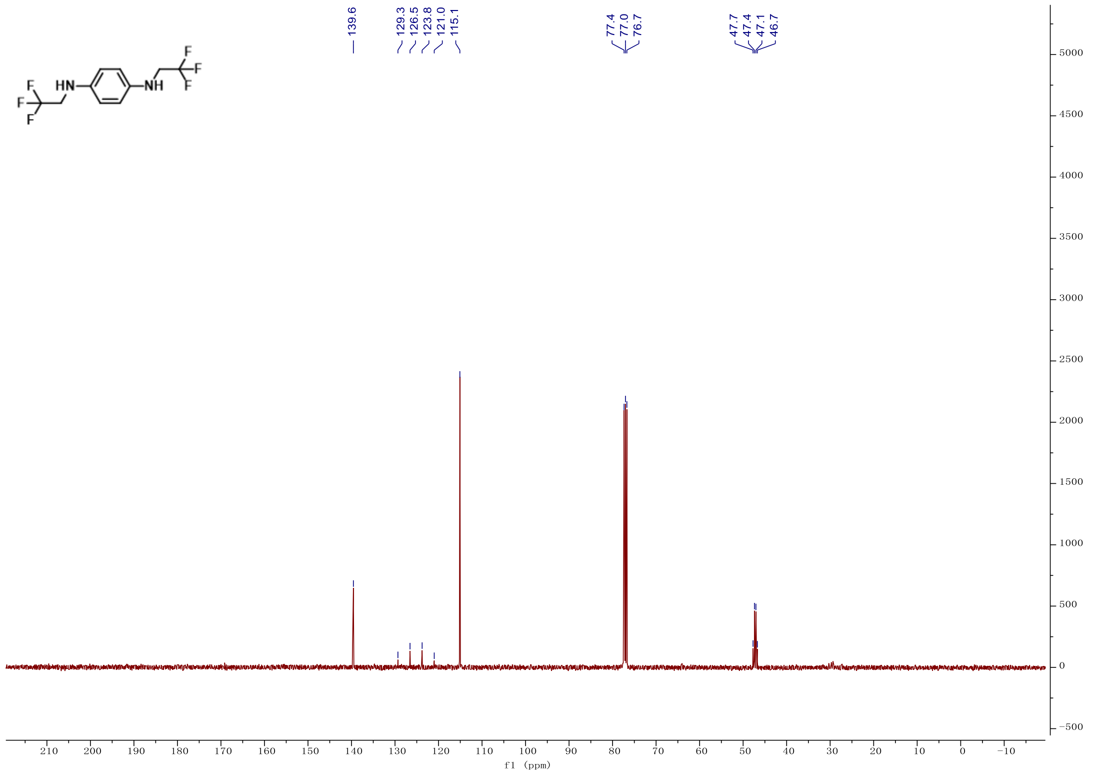

^{19}F NMR of Compound 32 (376 MHz, $CDCl_3$)

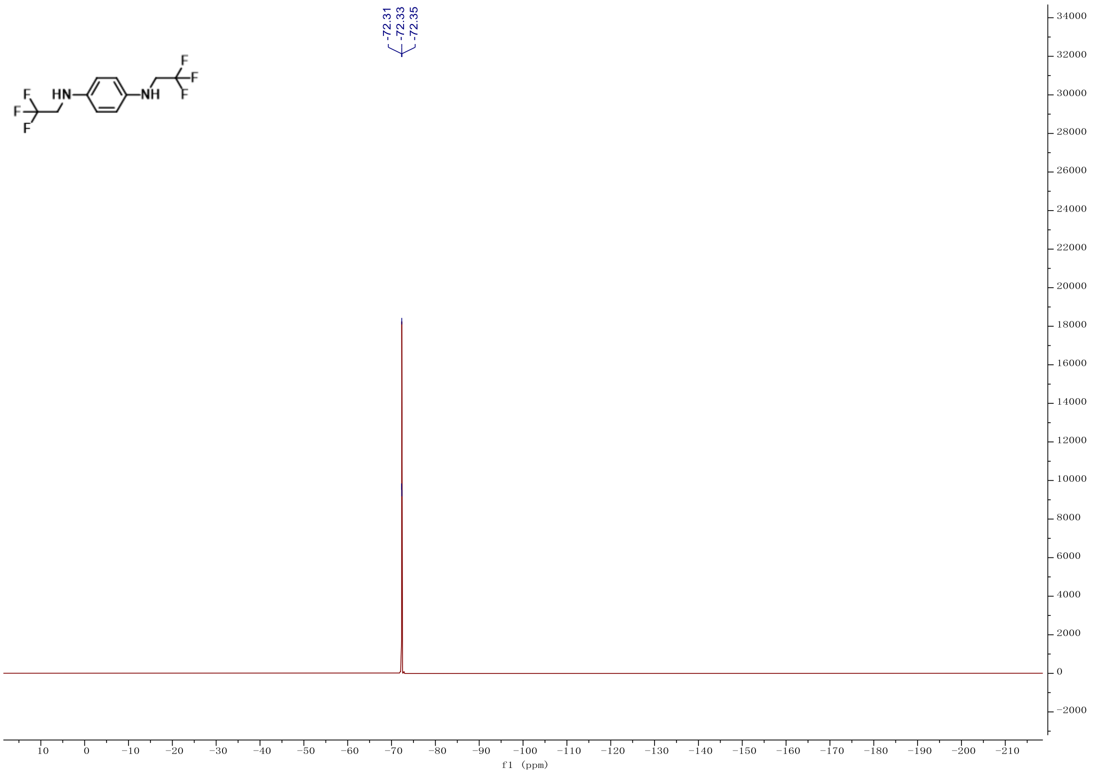

^{1}H NMR of Compound 33 (400 MHz, $CDCl_3$)

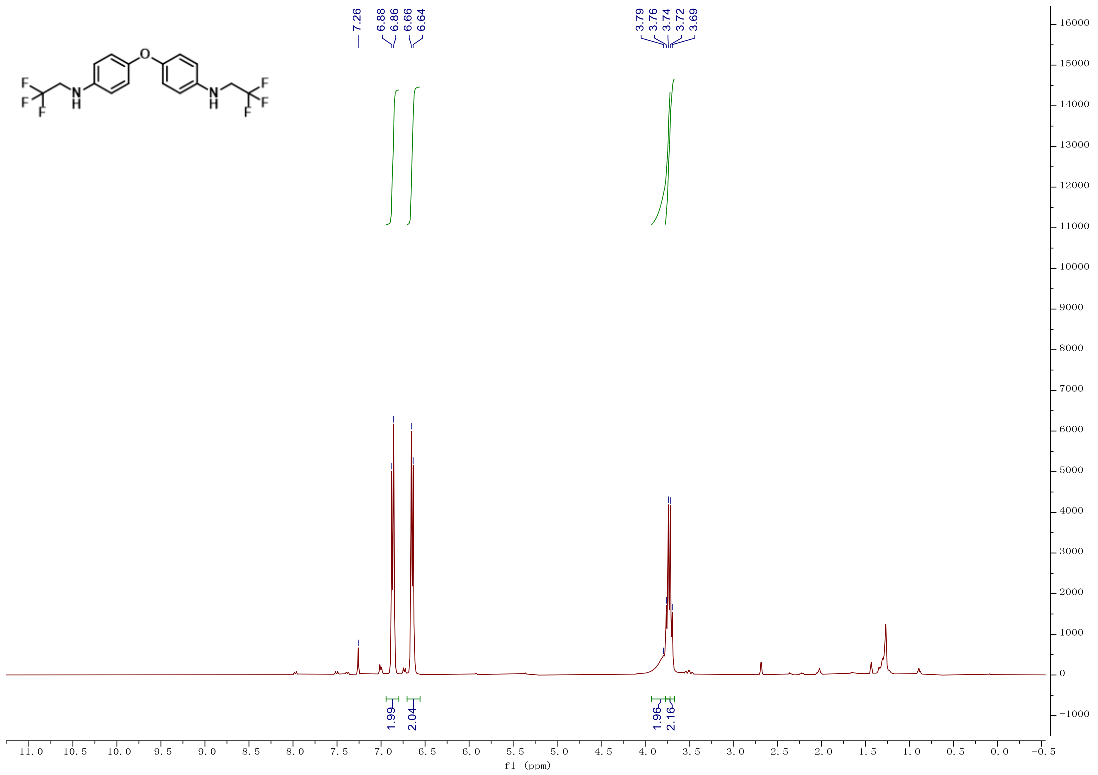

^{13}C NMR of Compound 33 (101 MHz, $CDCl_3$)

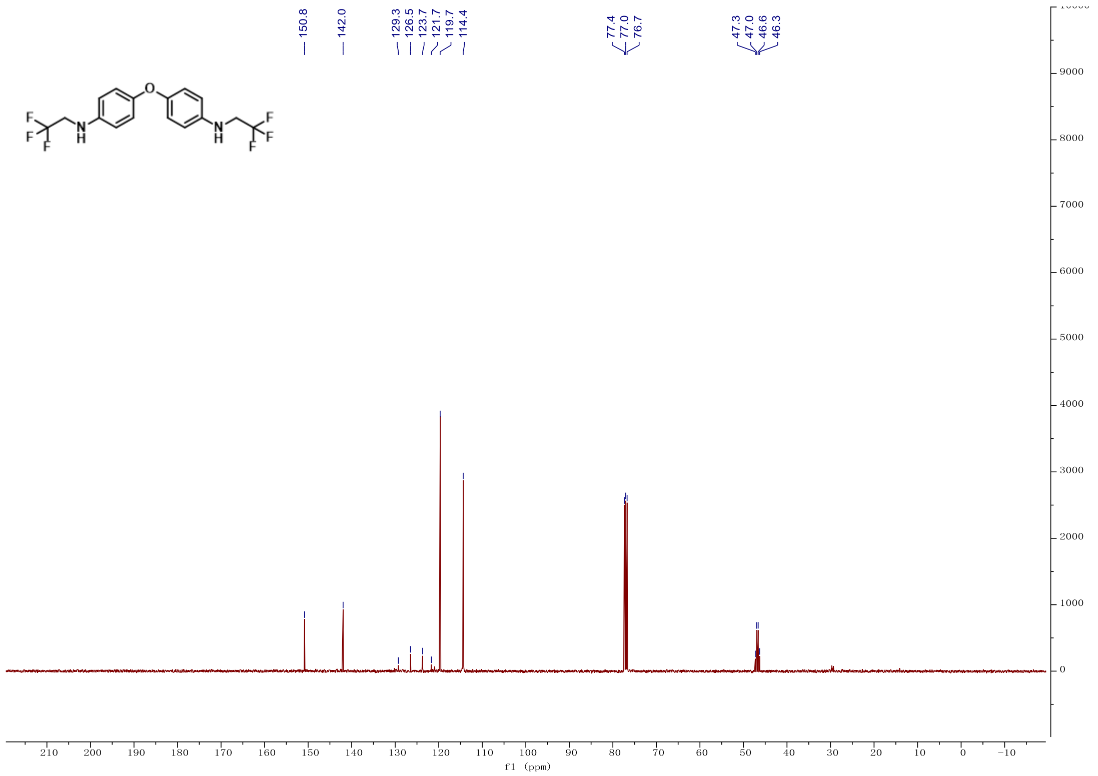

^{19}F NMR of Compound 33 (376 MHz, $CDCl_3$)

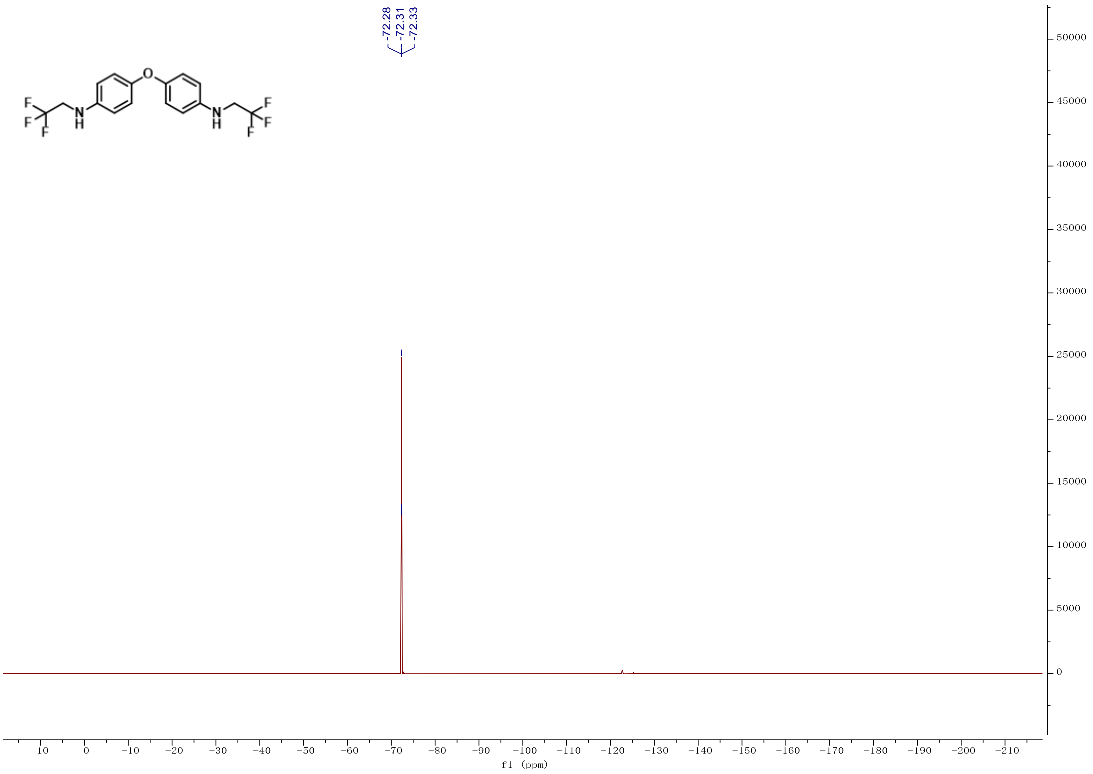

^{1}H NMR of Compound 3-d (400 MHz, DMSO-d^{6})

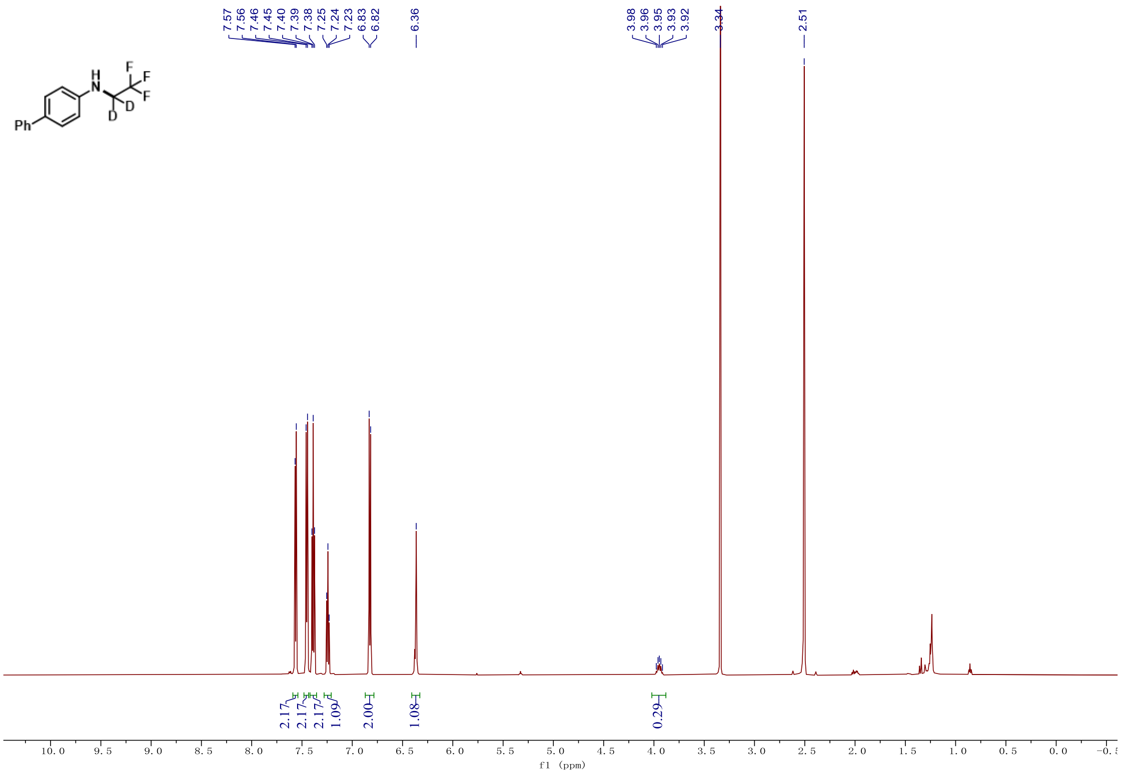

^{13}C NMR of Compound 3-d (101 MHz, DMSO-d^{6})

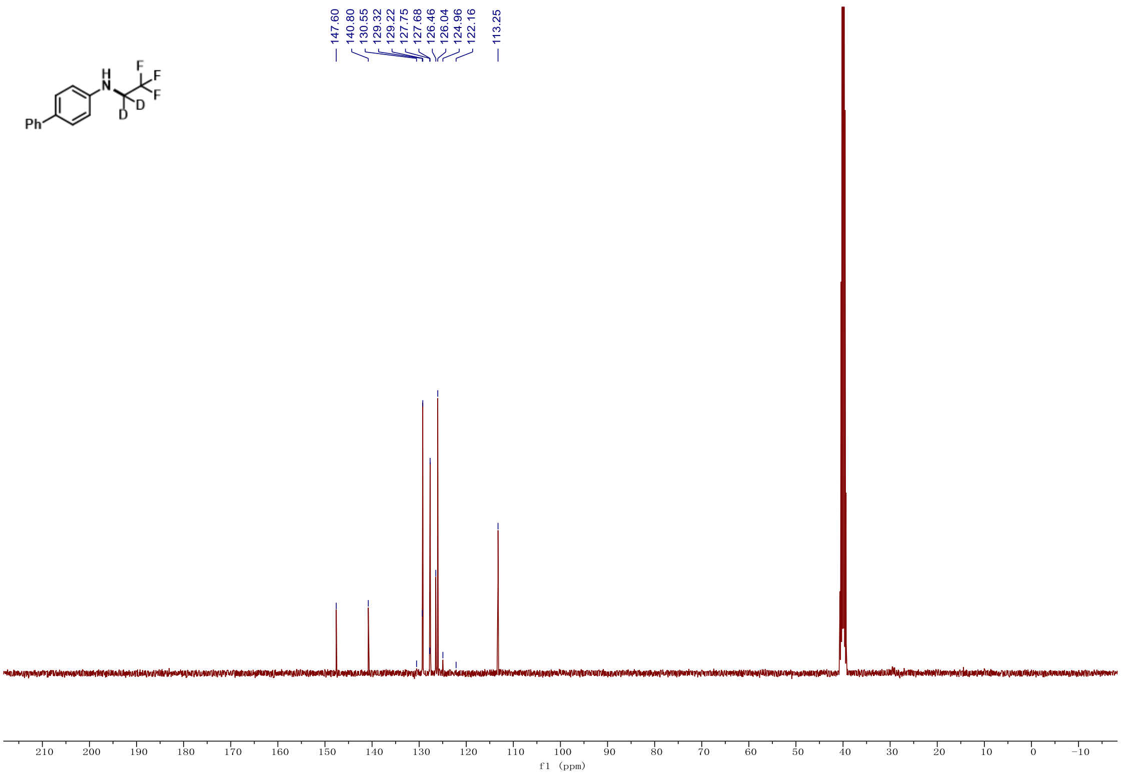

^{19}F NMR of Compound 3-d (376 MHz, DMSO-d6)

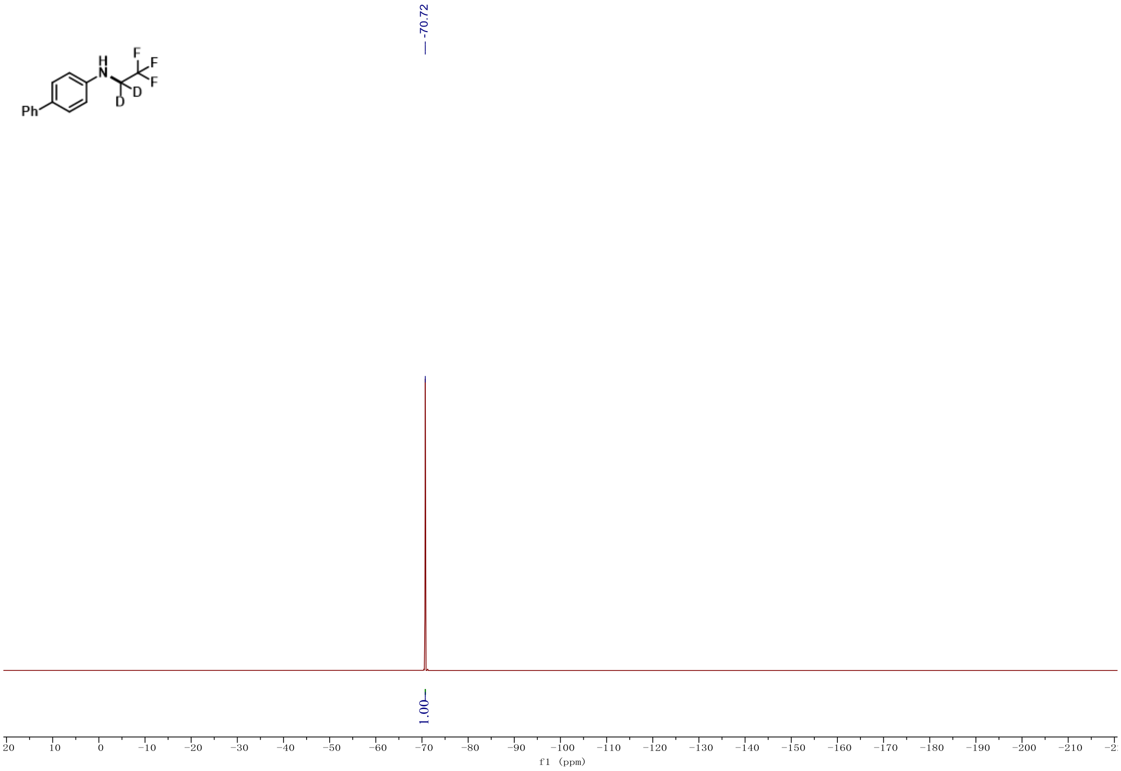

^{1}H NMR of Compound 34 (400 MHz, $CDCl_3$)

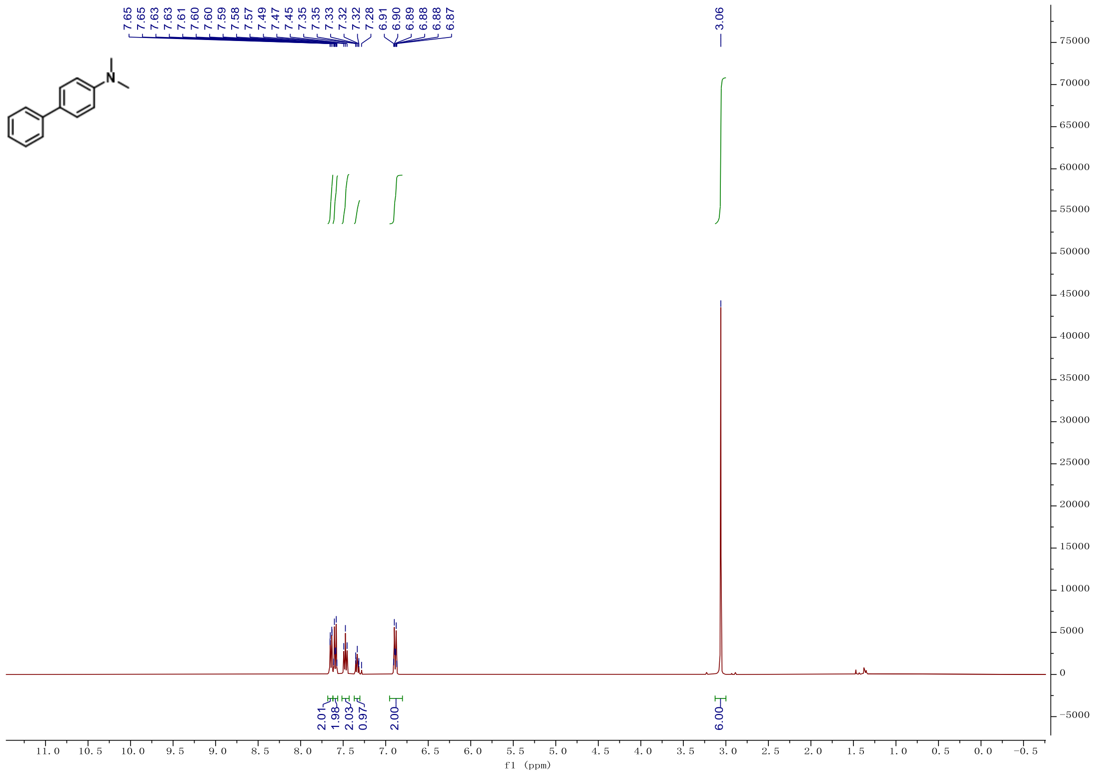

^{13}C NMR of Compound 34 (101 MHz, $CDCl_3$)

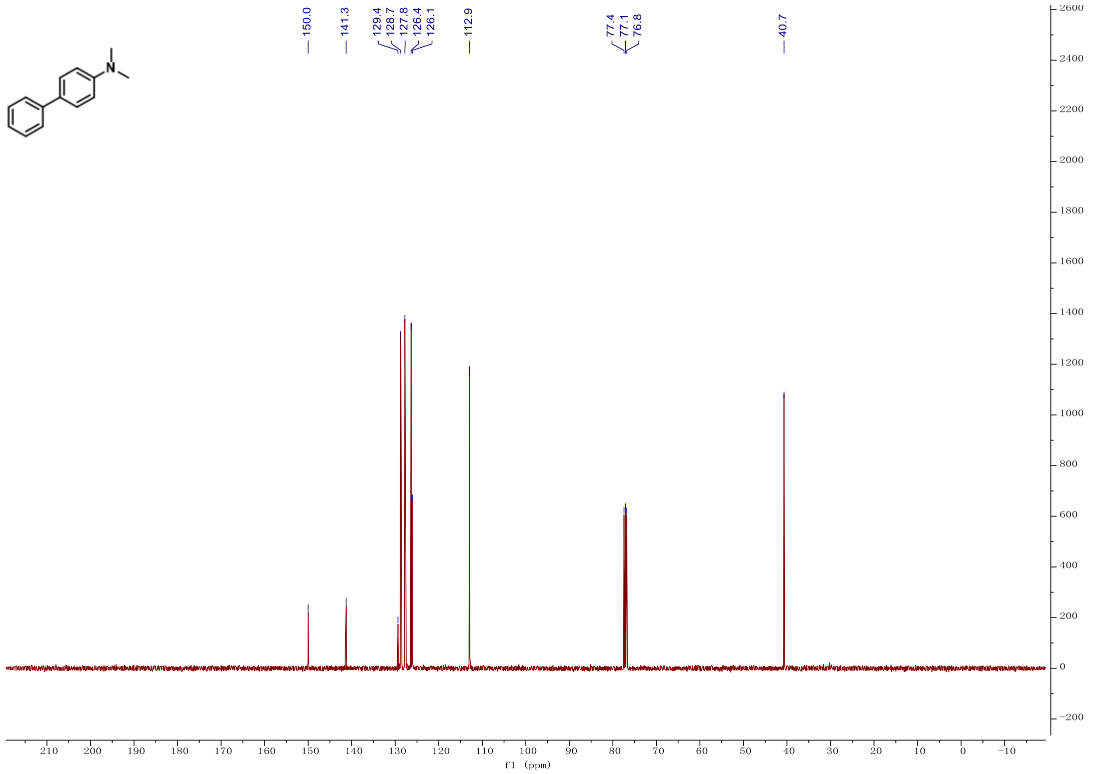

^{1}H NMR of Compound 35 (400 MHz, $CDCl_3$)

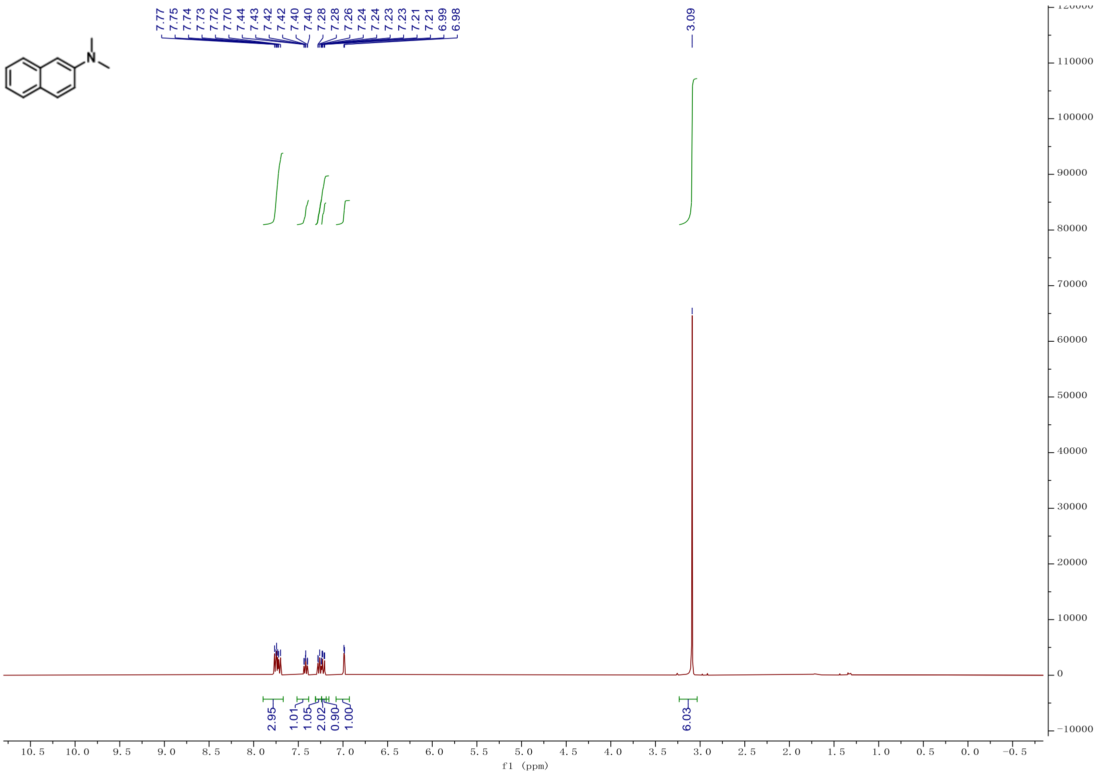

^{13}C NMR of Compound 35 (101 MHz, $CDCl_3$)

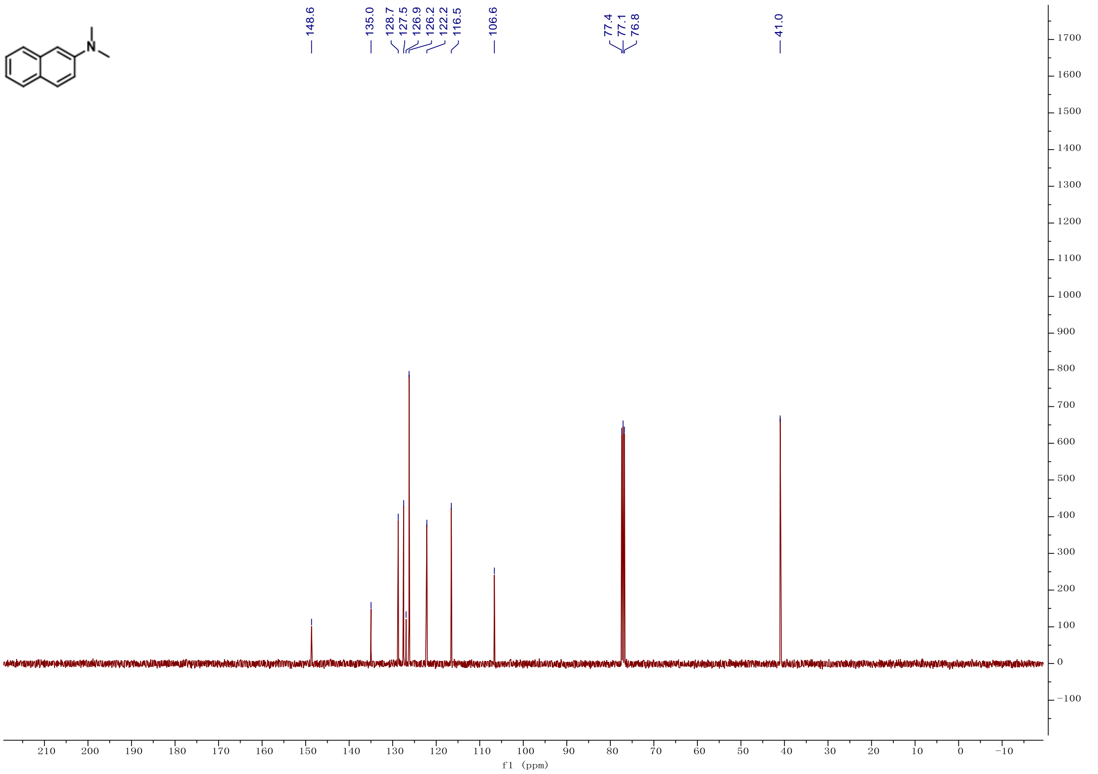

^{1}H NMR of Compound 36 (400 MHz, $CDCl_3$)

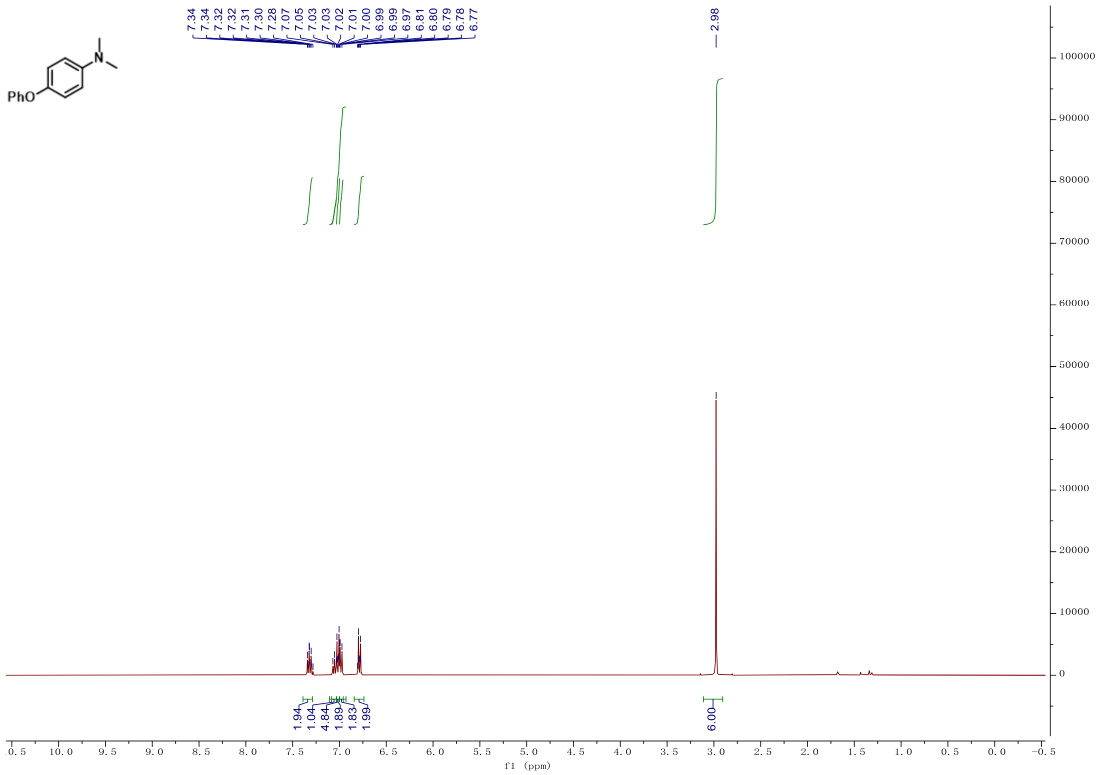

^{13}C NMR of Compound 36 (101 MHz, $CDCl_3$)

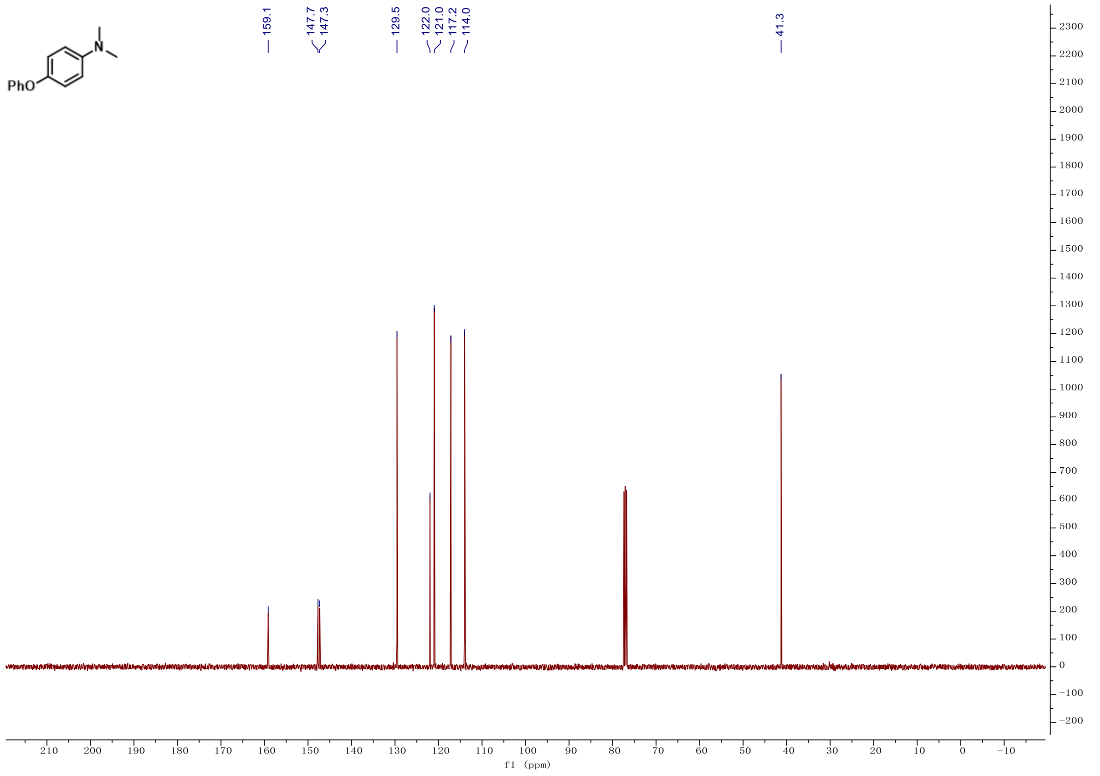

^{1}H NMR of Compound 37 (400 MHz, $CDCl_3$)

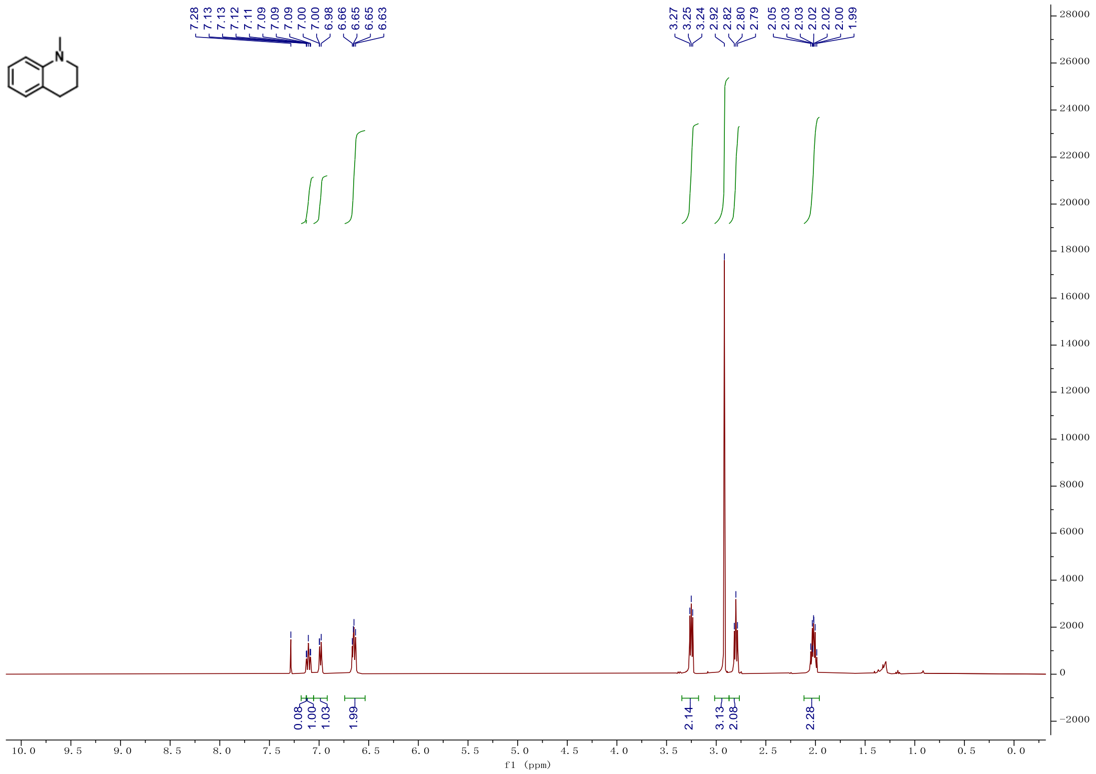

^{13}C NMR of Compound 37 (101 MHz, $CDCl_3$)

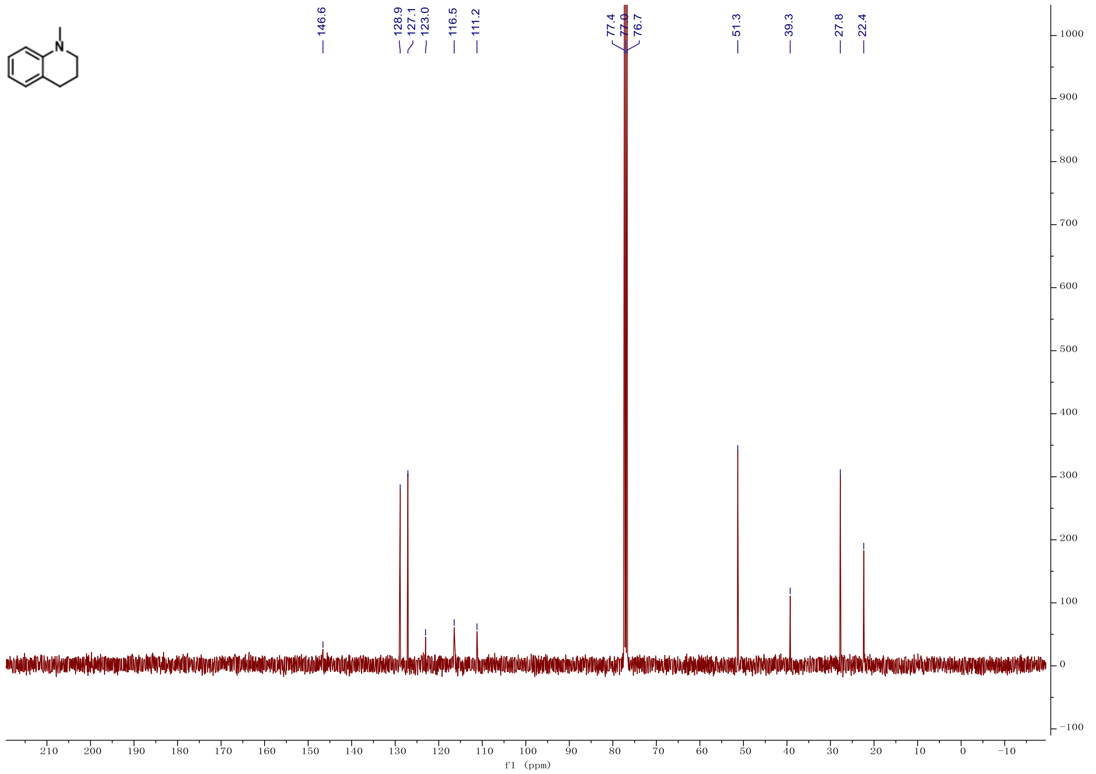

^{1}H NMR of Compound 38 (400 MHz, $CDCl_3$)

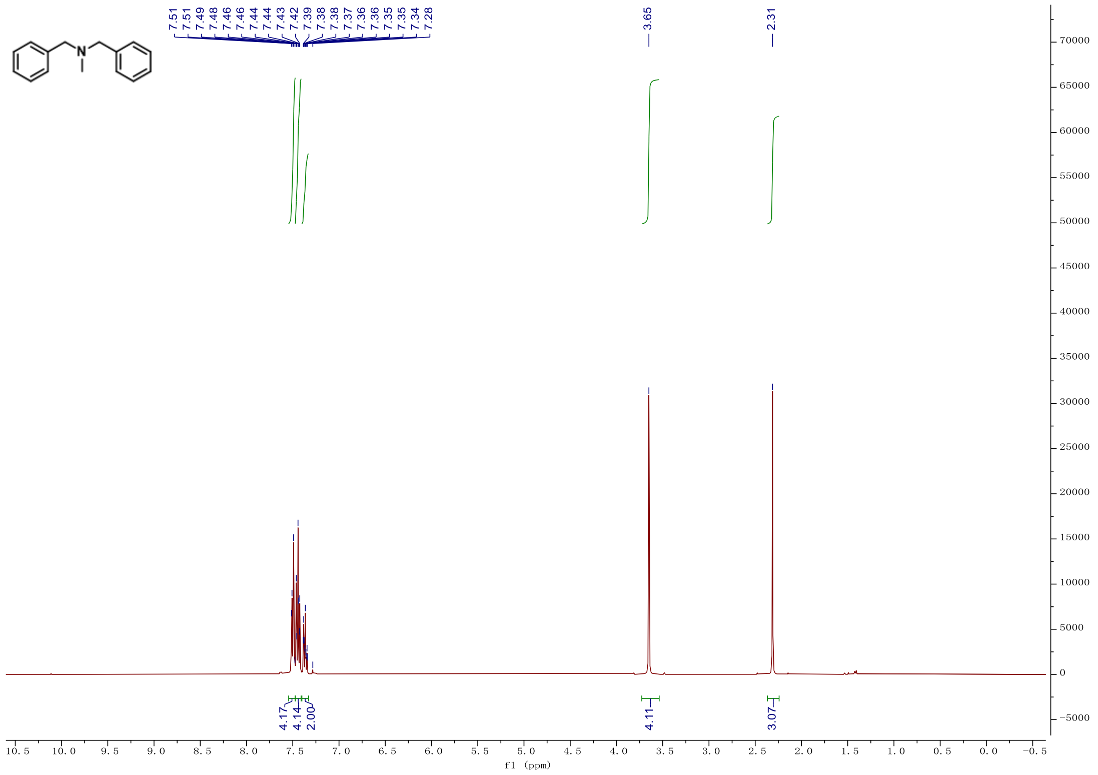

^{13}C NMR of Compound 38 (101 MHz, $CDCl_3$)

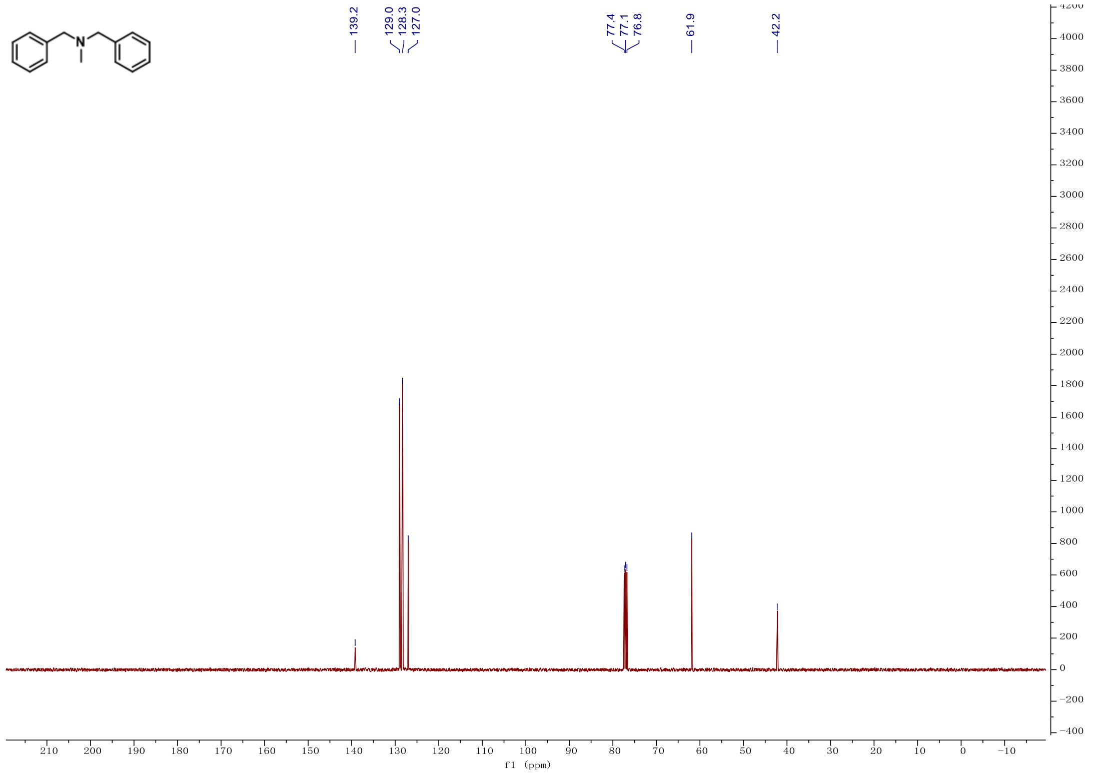

^{1}H NMR of Compound 39 (400 MHz, $CDCl_3$)

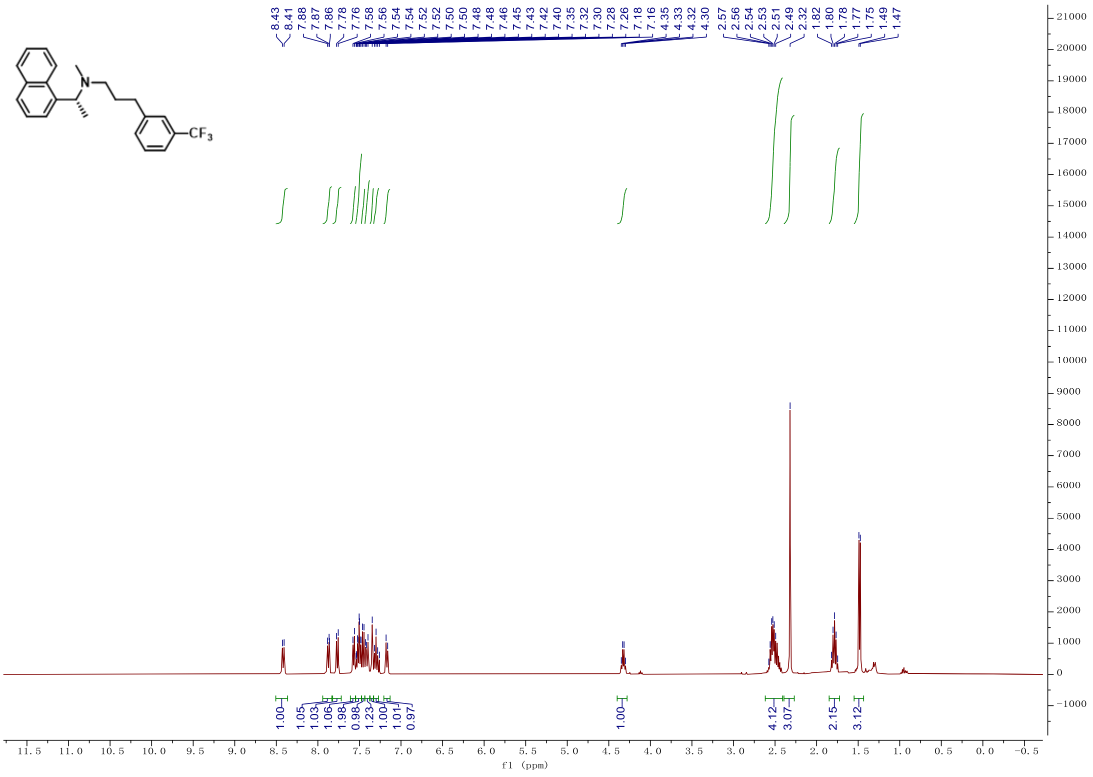

^{13}C NMR of Compound 39 (101 MHz, $CDCl_3$)

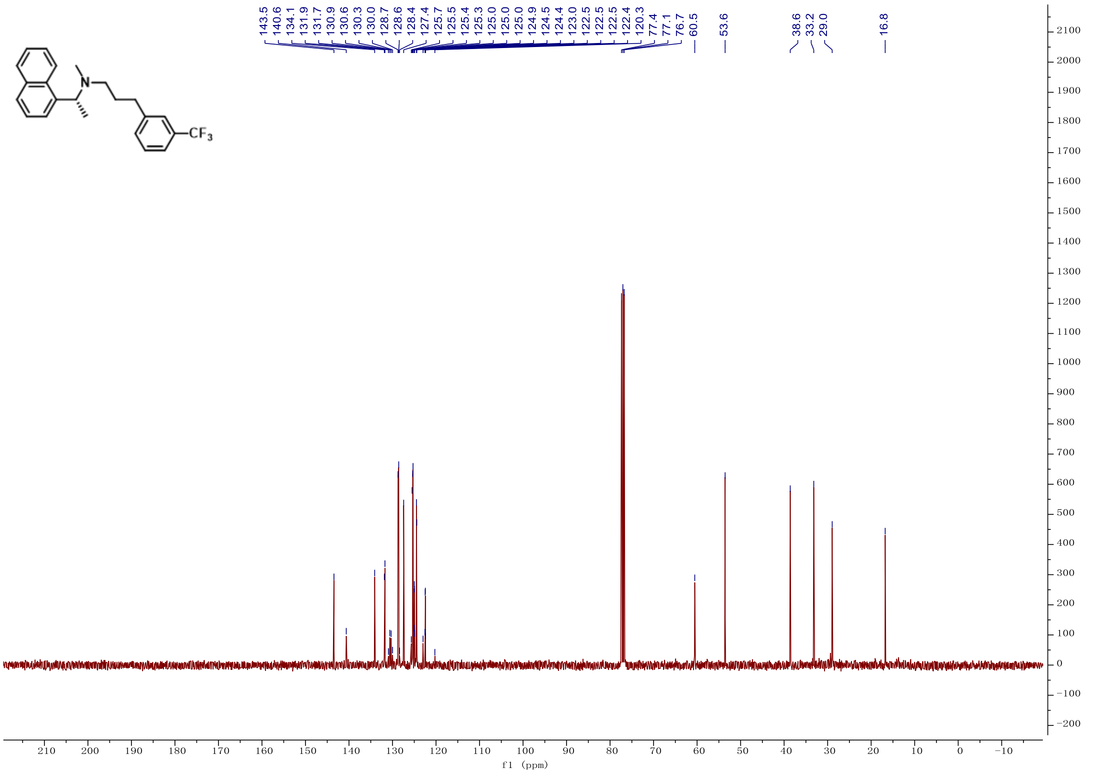

^{19}F NMR of Compound 39 (376 MHz, $CDCl_3$)

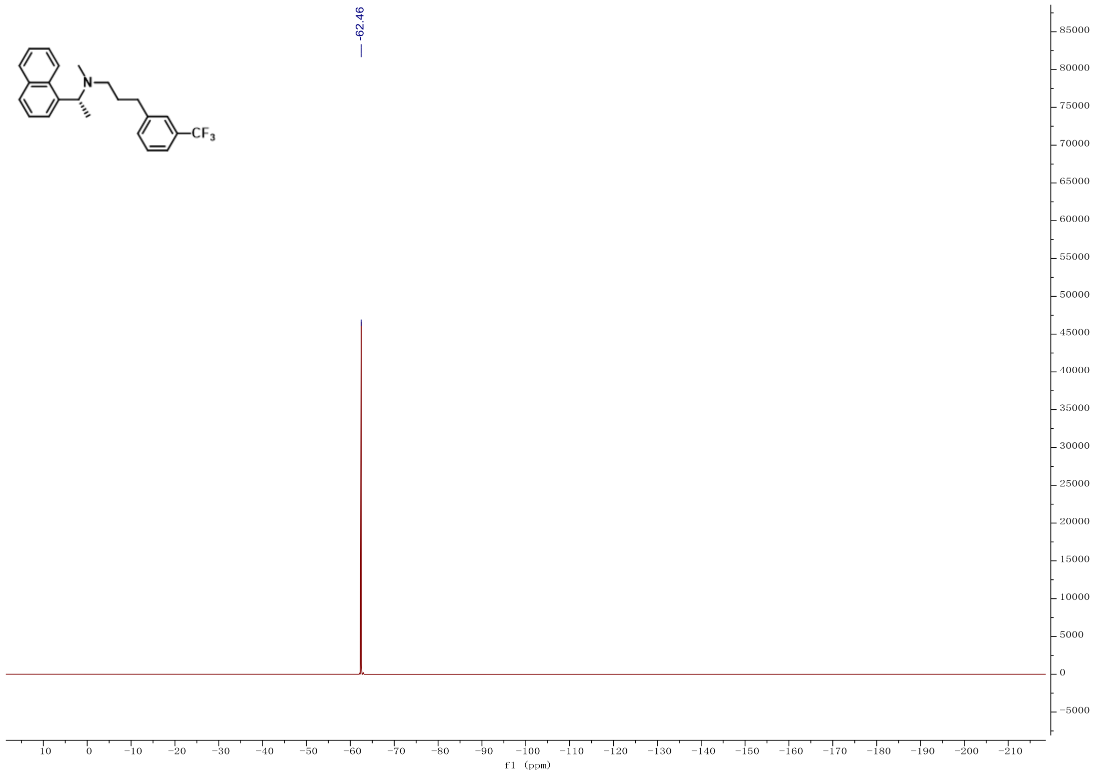

^{1}H NMR of Compound 40 (400 MHz, $CDCl_3$)

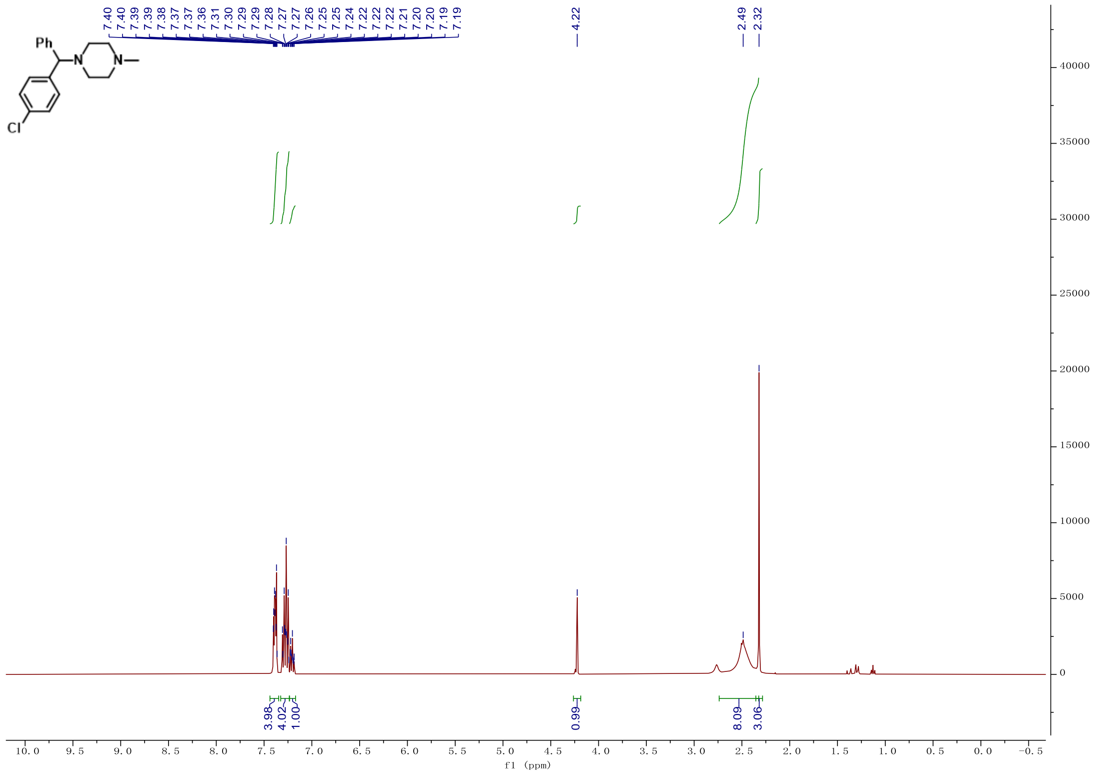

^{13}C NMR of Compound 40 (101 MHz, $CDCl_3$)

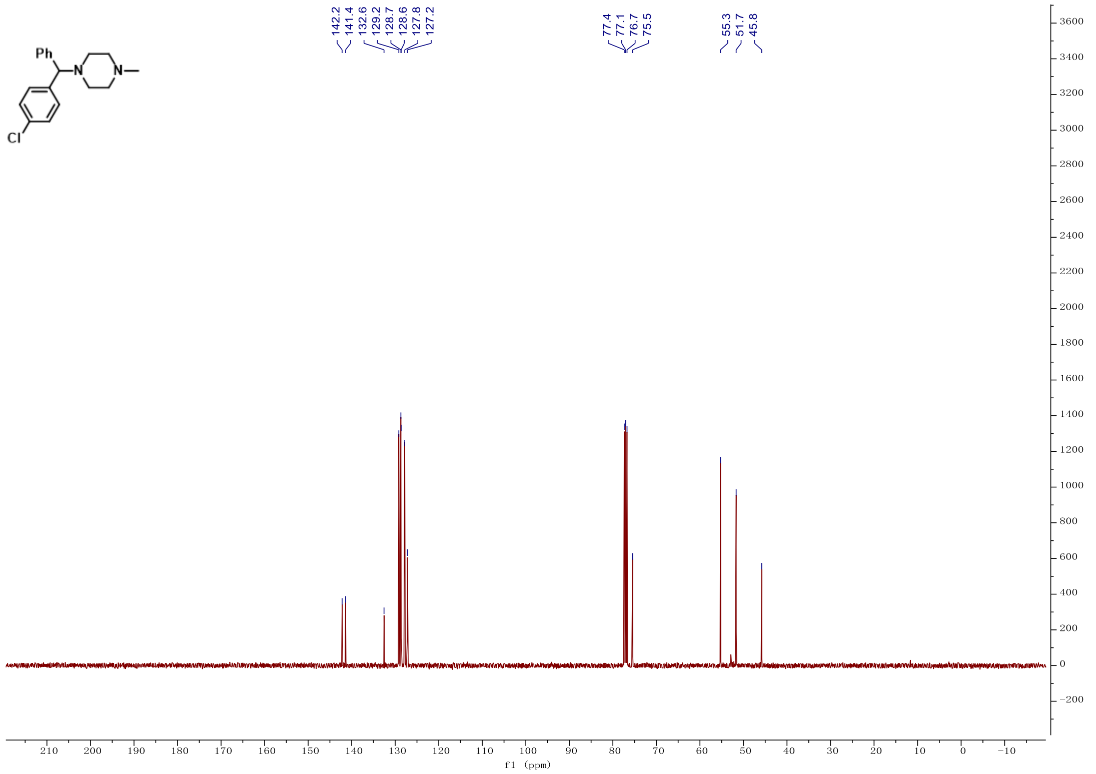

^{1}H NMR of Compound 41 (400 MHz, $CDCl_3$)

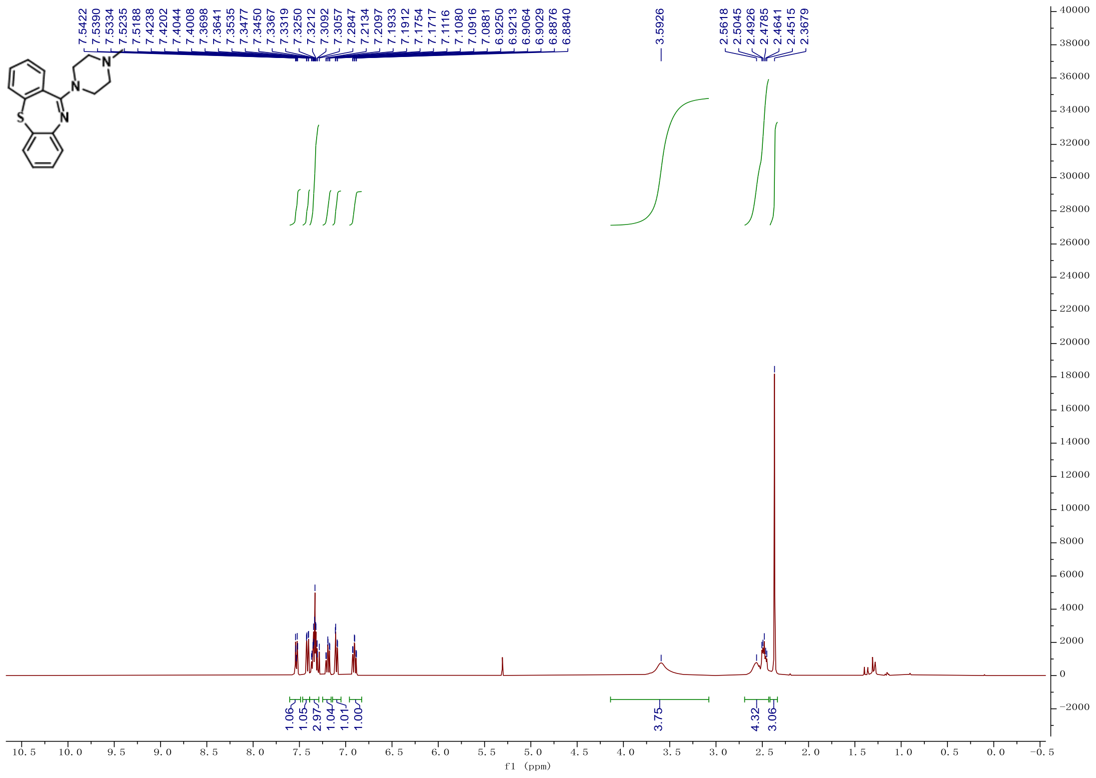

^{13}C NMR of Compound 41 (101 MHz, $CDCl_3$)

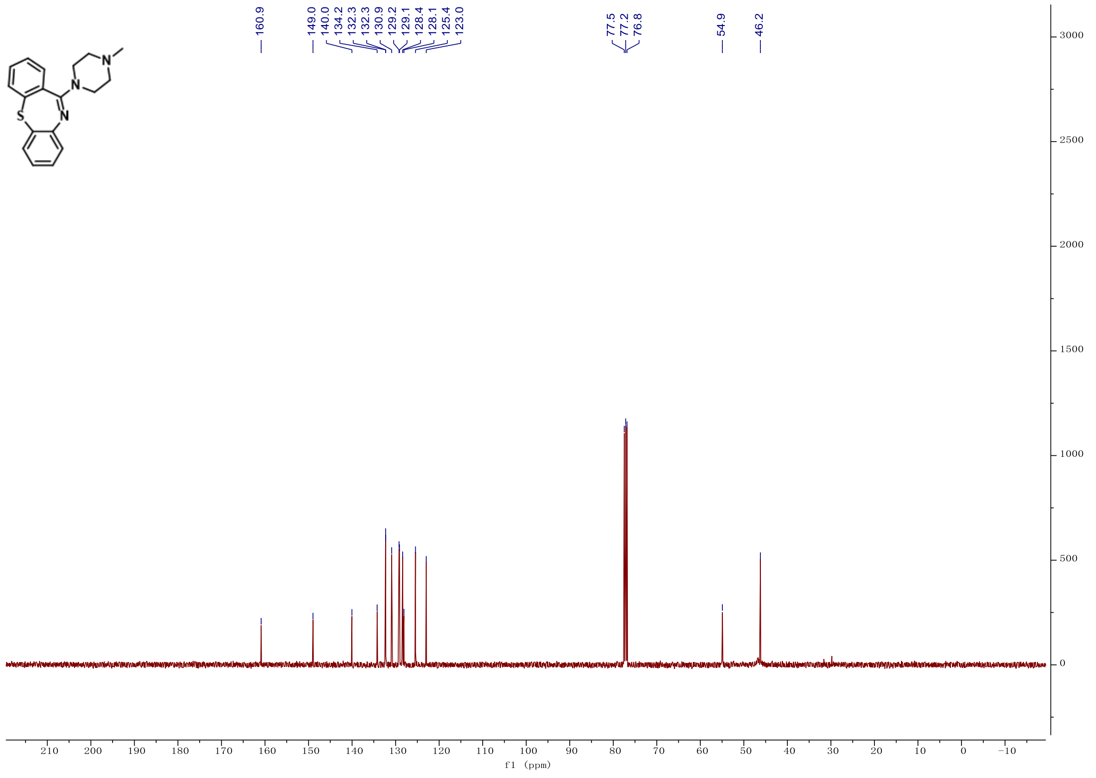

^{1}H NMR of Compound 42 (400 MHz, $CDCl_3$)

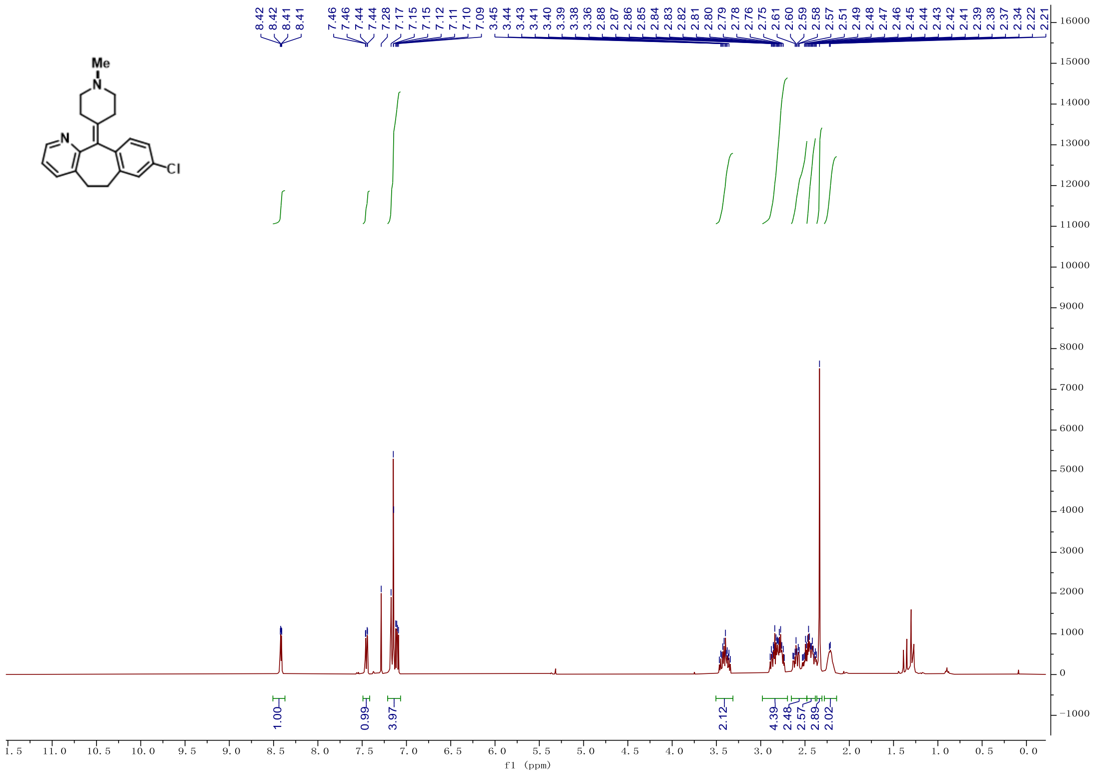

^{13}C NMR of Compound 42 (101 MHz, $CDCl_3$)

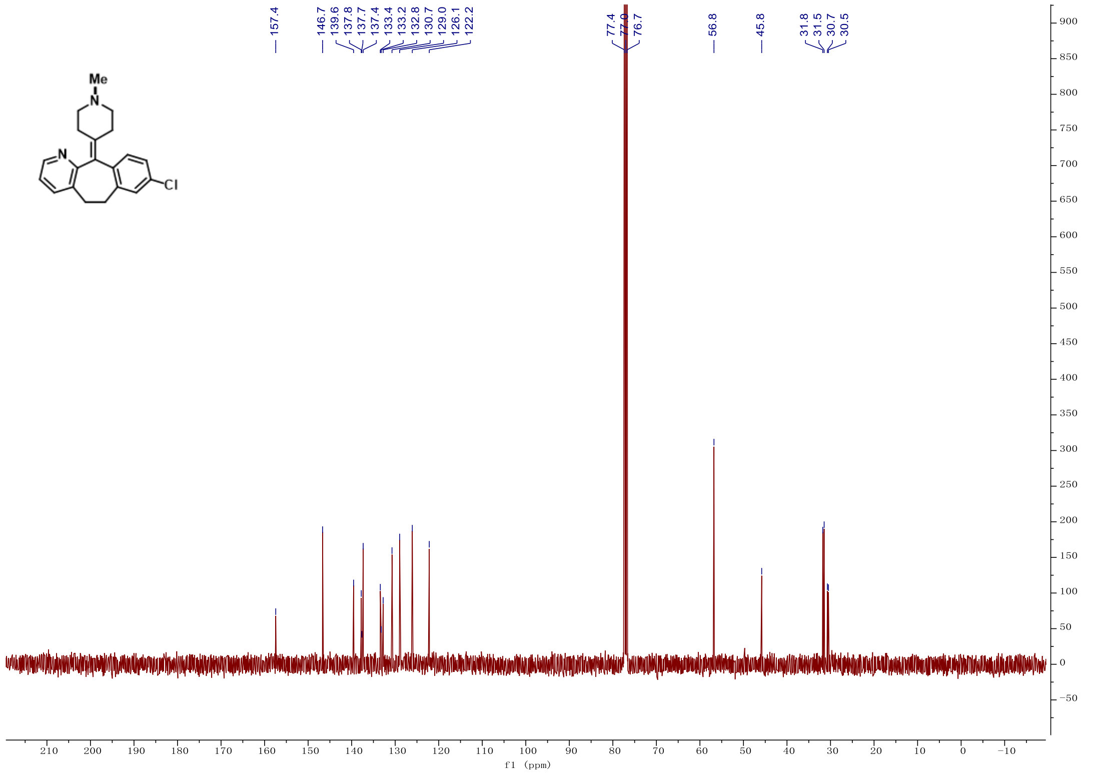

^{1}H NMR of Compound 43 (400 MHz, $CDCl_3$)

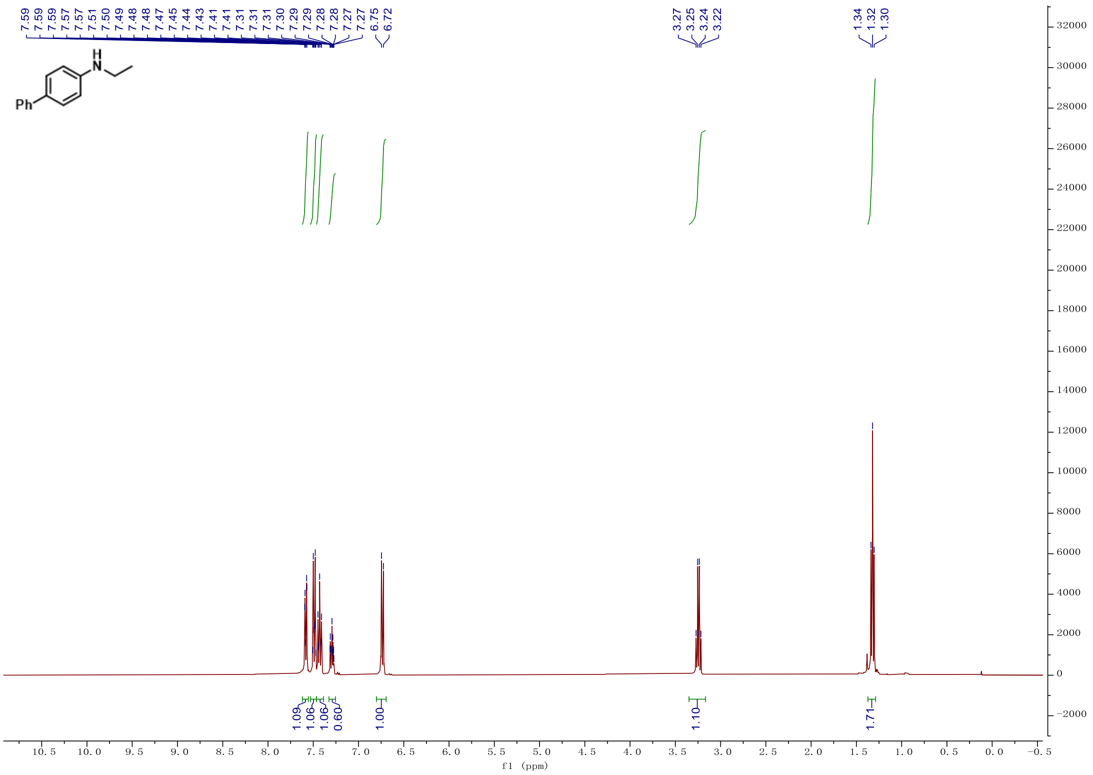

^{13}C NMR of Compound 43 (101 MHz, $CDCl_3$)

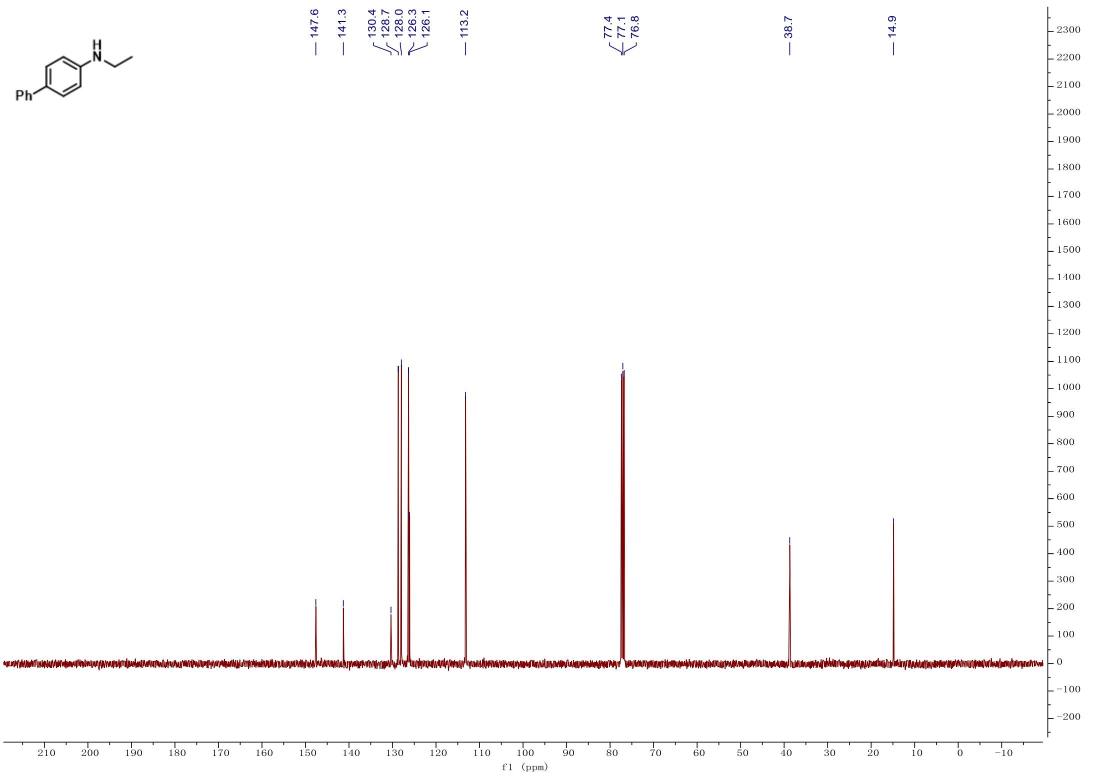

^{1}H NMR of Compound 44 (400 MHz, $CDCl_3$)

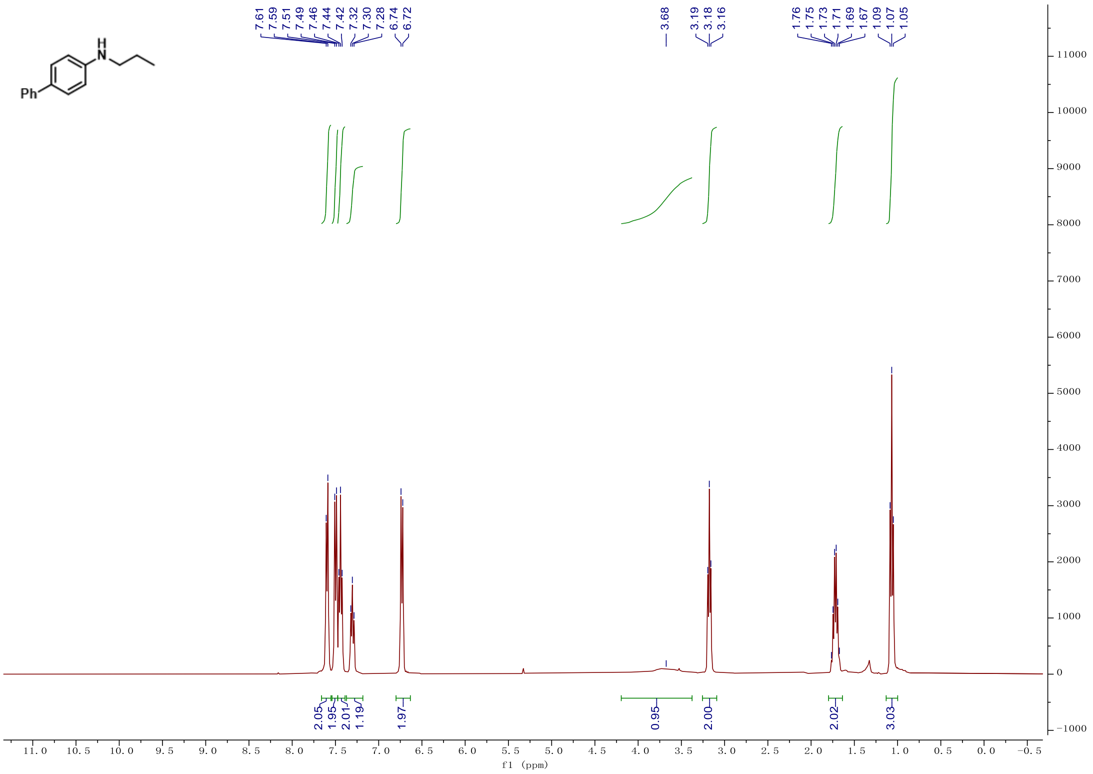

^{13}C NMR of Compound 44 (101 MHz, $CDCl_3$)

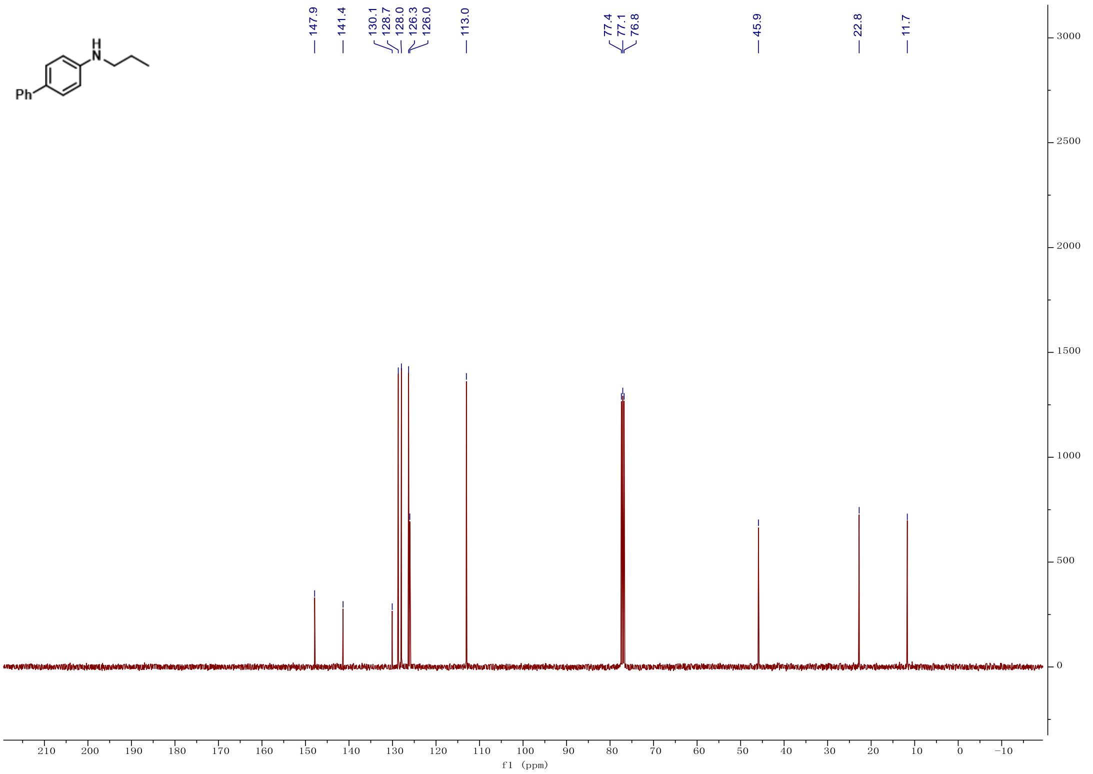

^{1}H NMR of Compound 45 (400 MHz, $CDCl_3$)

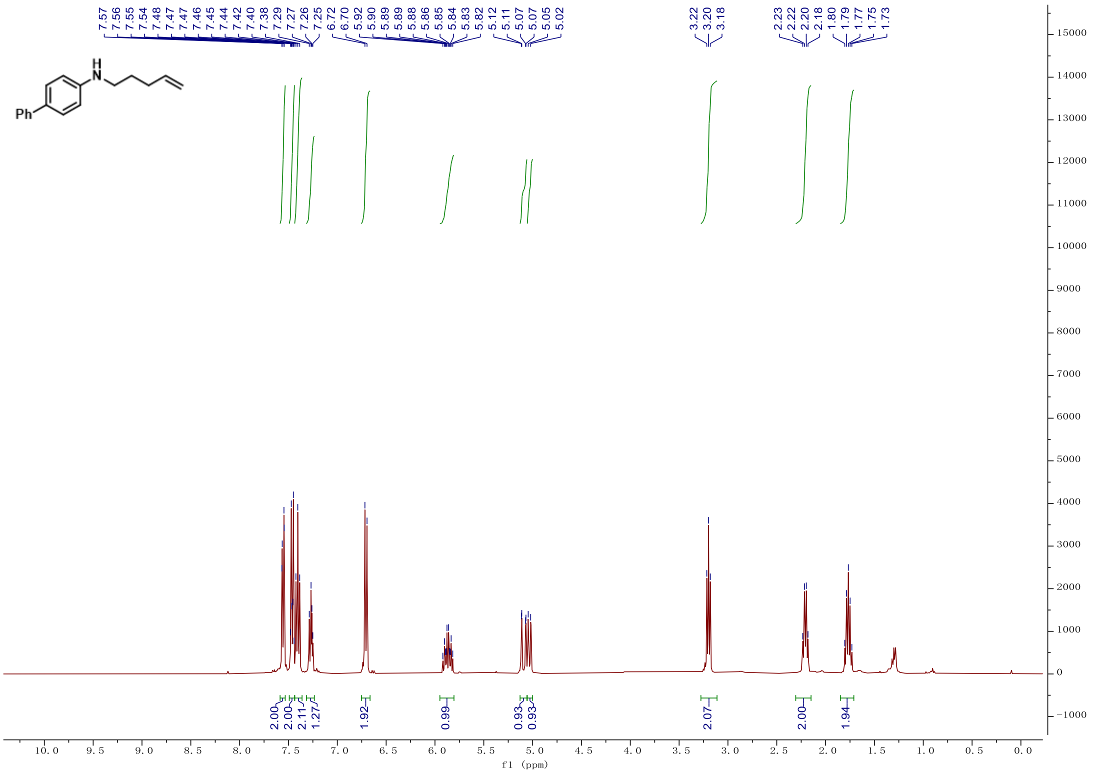

^{13}C NMR of Compound 45 (101 MHz, $CDCl_3$)

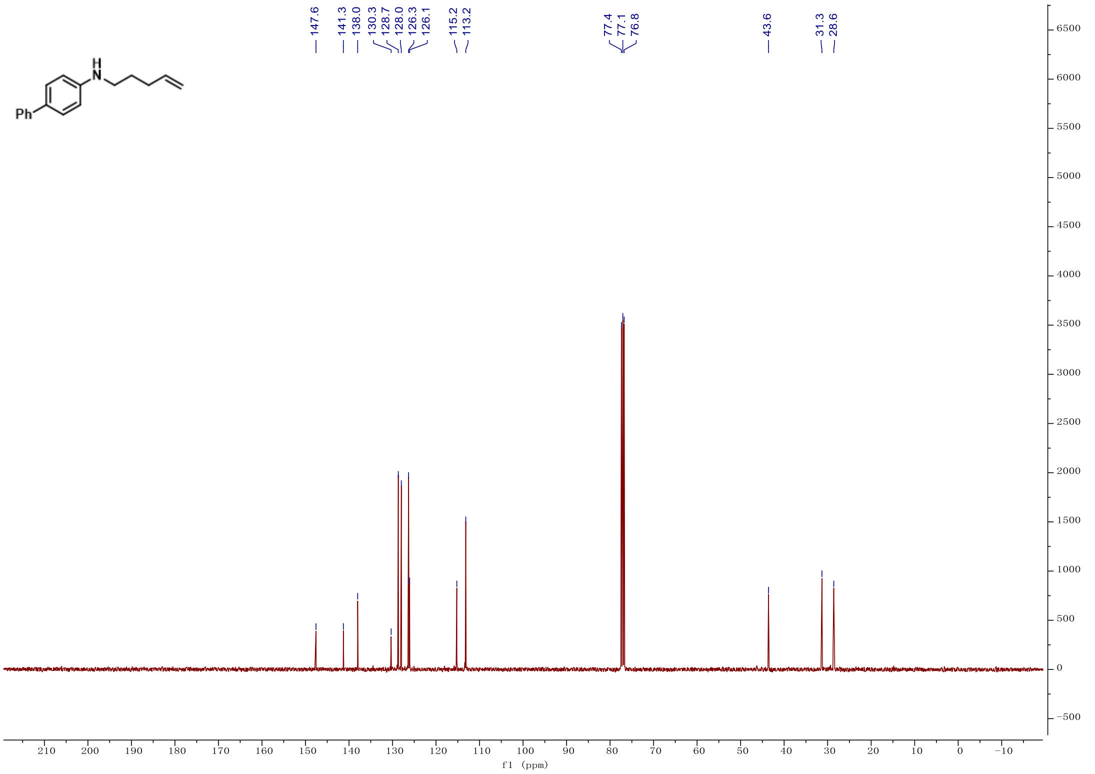

^{1}H NMR of Compound 46 (400 MHz, $CDCl_3$)

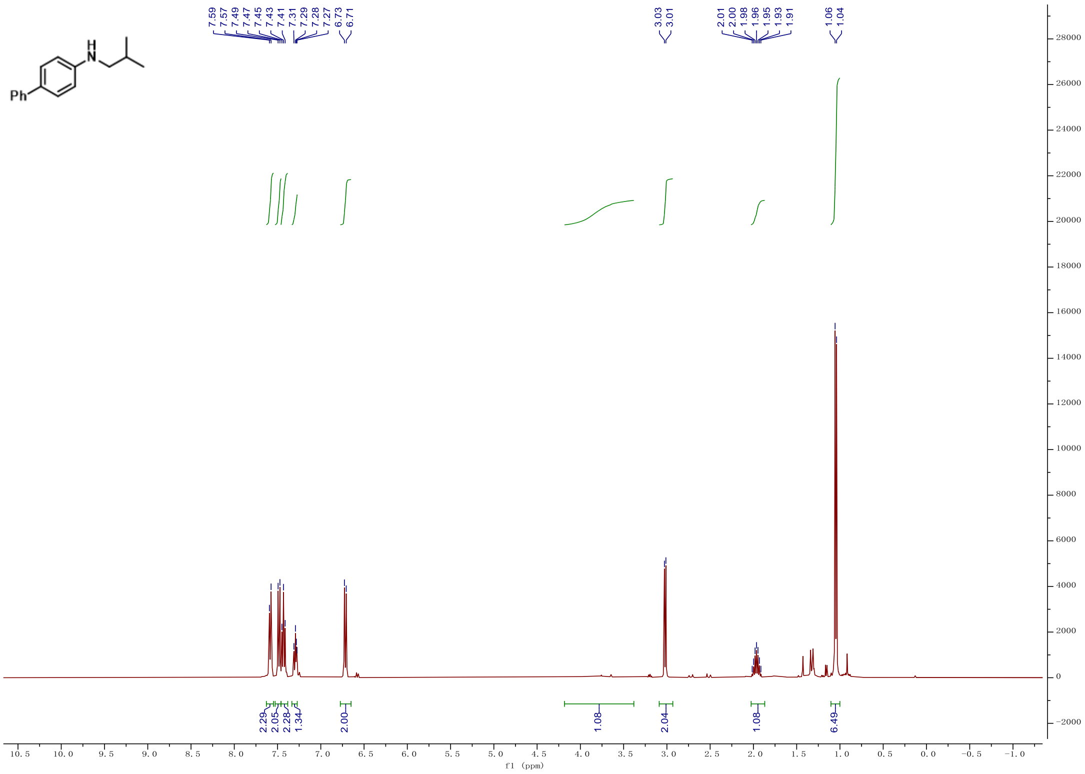

^{13}C NMR of Compound 46 (101 MHz, $CDCl_3$)

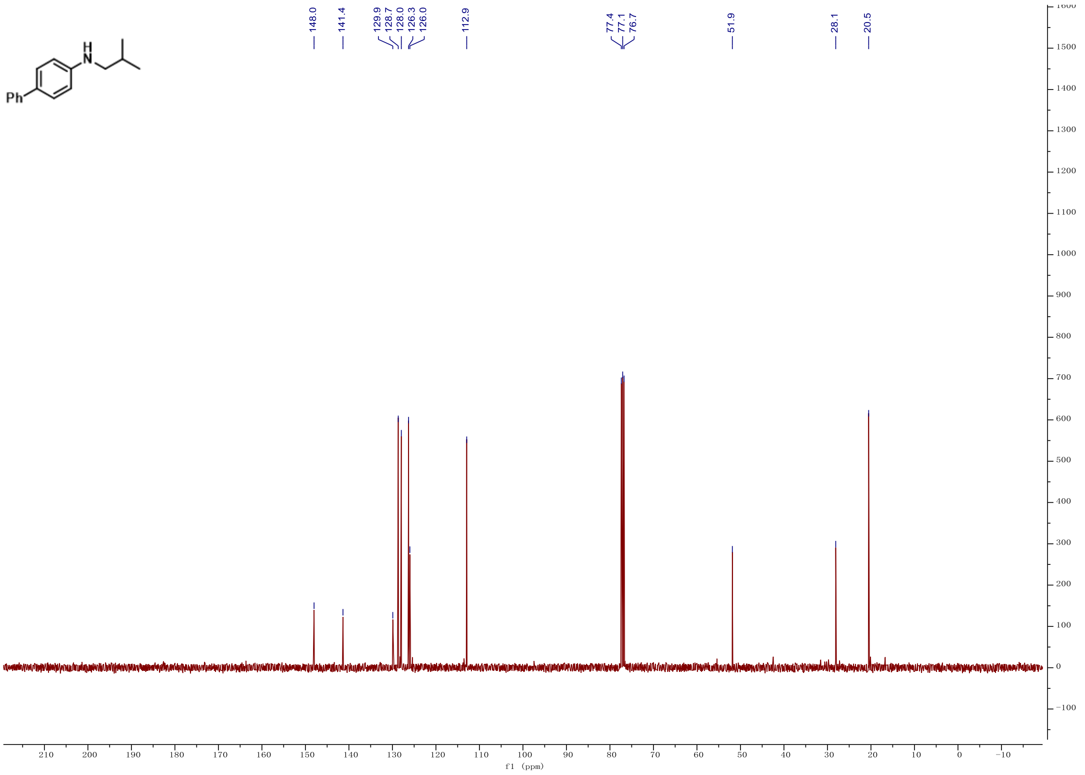

^{1}H NMR of Compound 47 (400 MHz, $CDCl_3$)

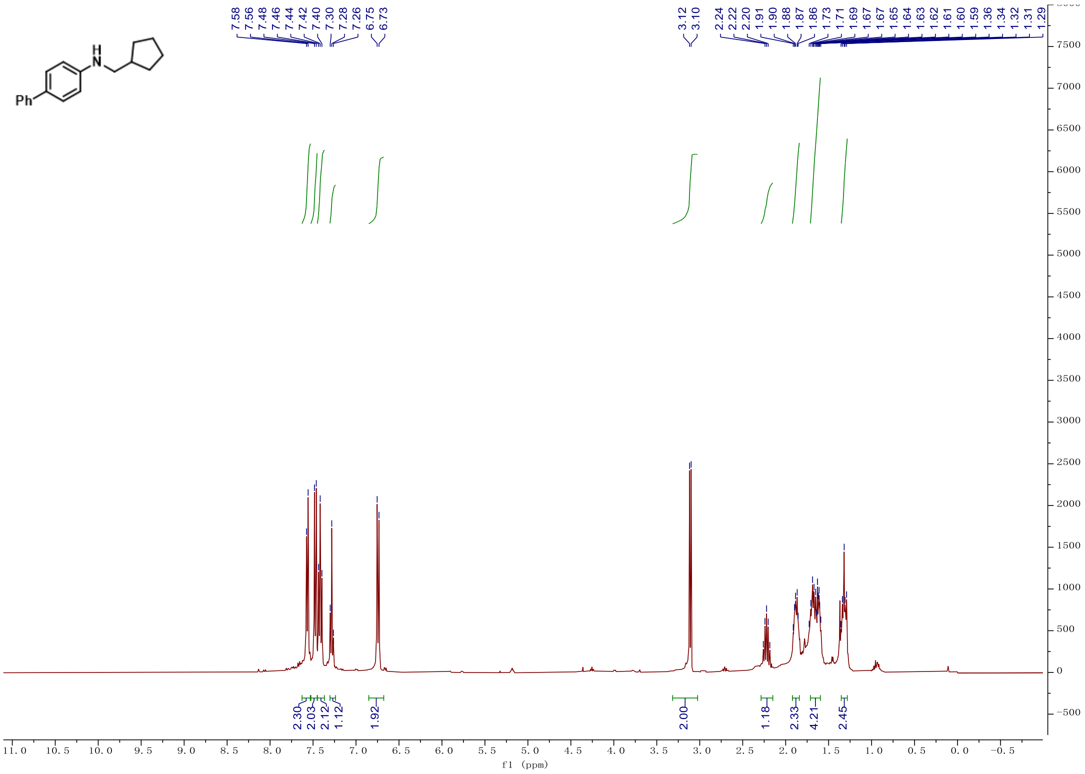

^{13}C NMR of Compound 47 (101 MHz, $CDCl_3$)

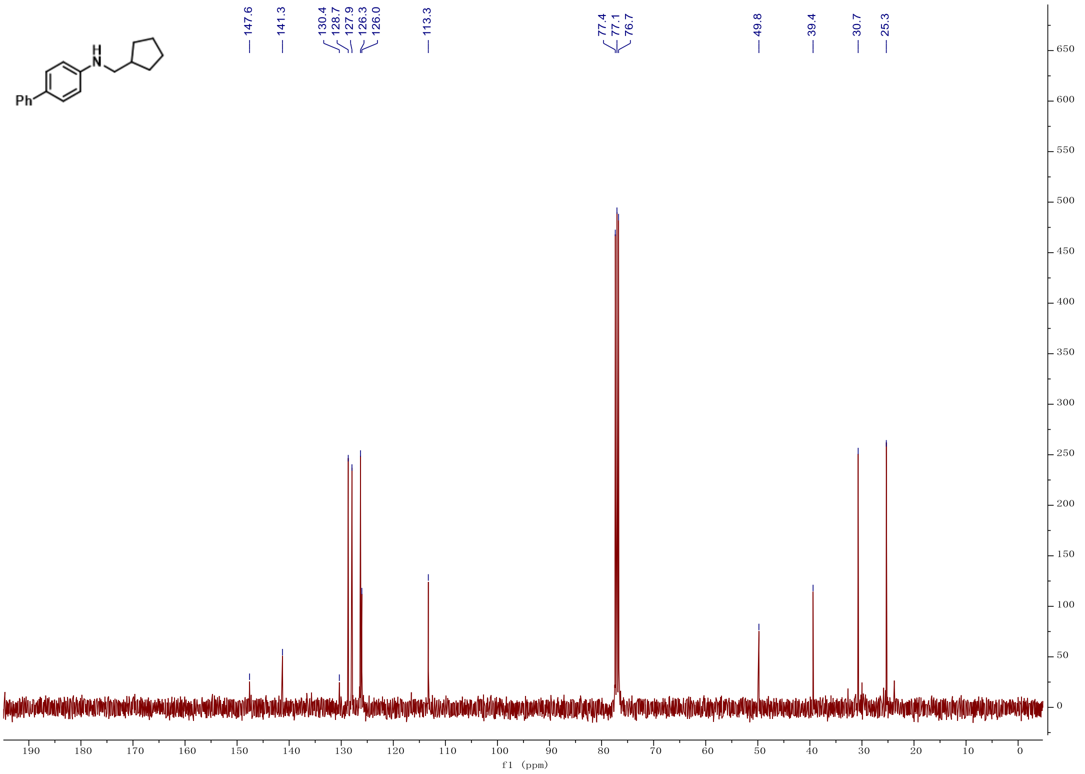

^{1}H NMR of Compound 48 (400 MHz, $CDCl_3$)

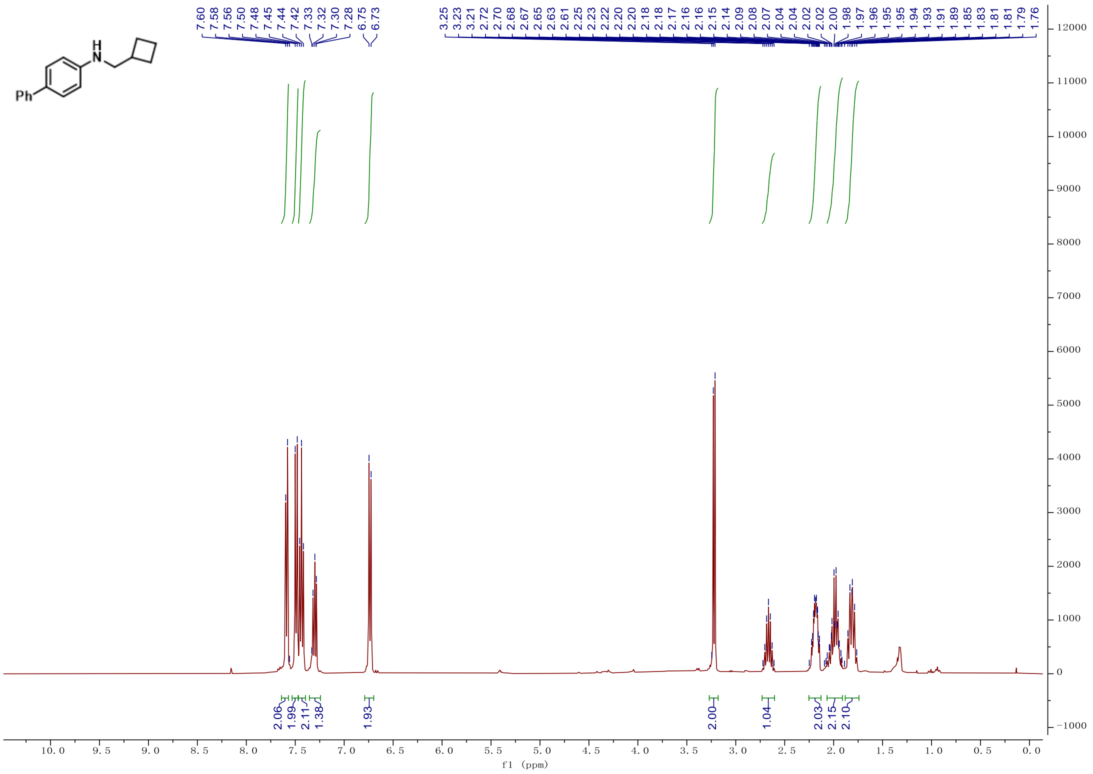

^{13}C NMR of Compound 48 (101 MHz, $CDCl_3$)

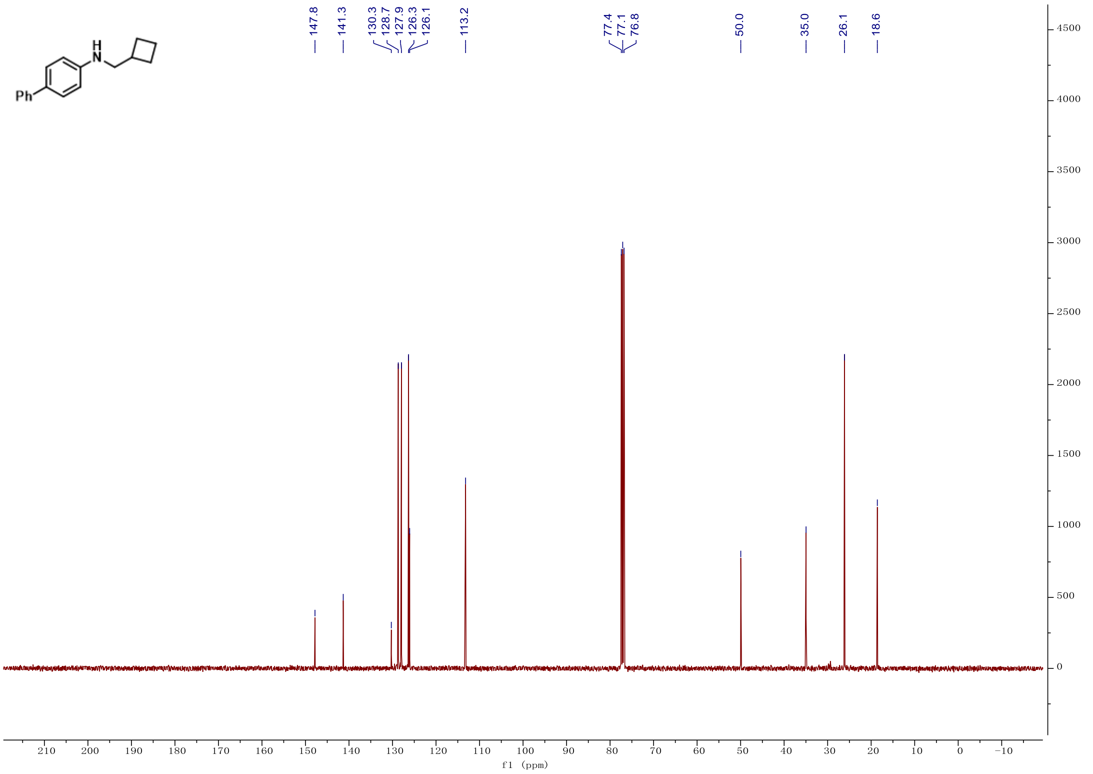

^{1}H NMR of Compound 49 (400 MHz, $CDCl_3$)

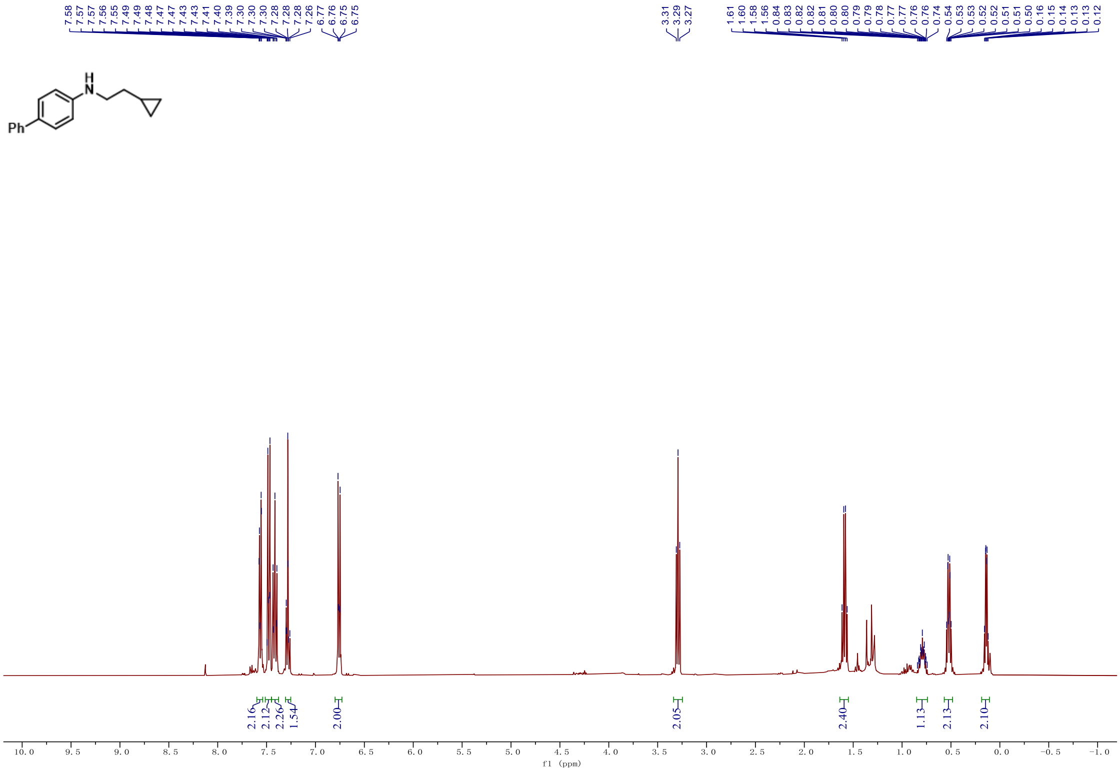

^{13}C NMR of Compound 49 (101 MHz, $CDCl_3$)

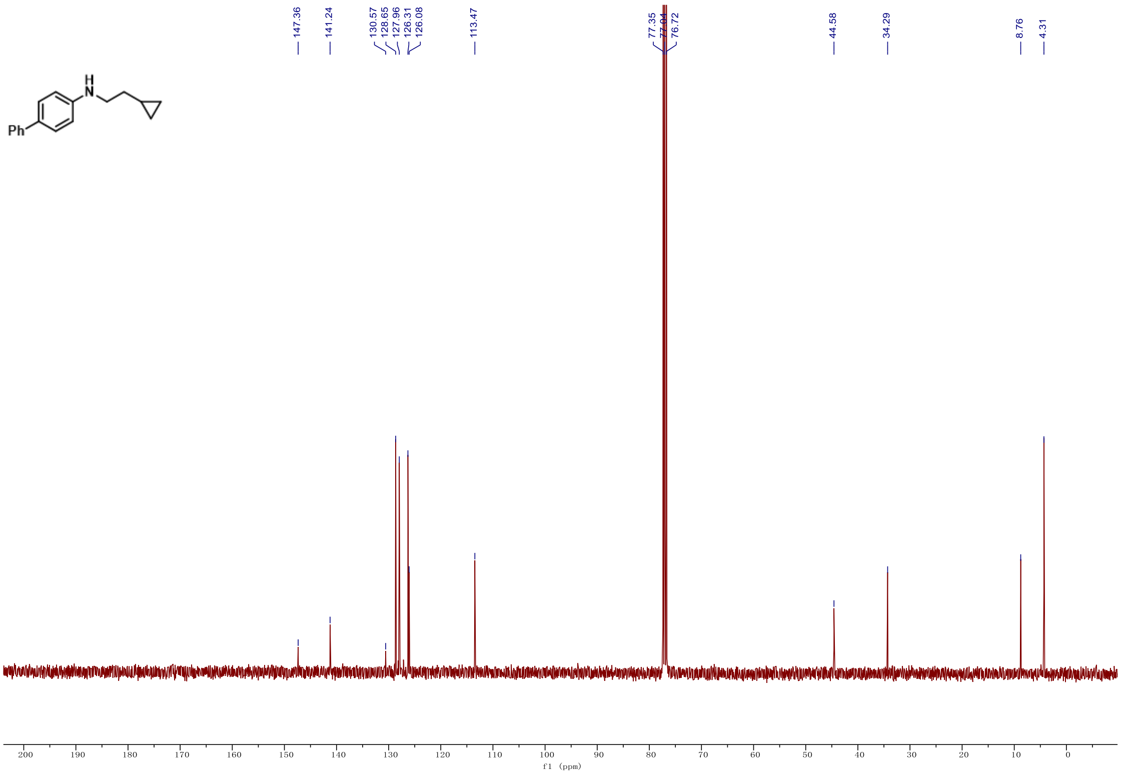

^{1}H NMR of Compound 50 (400 MHz, $CDCl_3$)

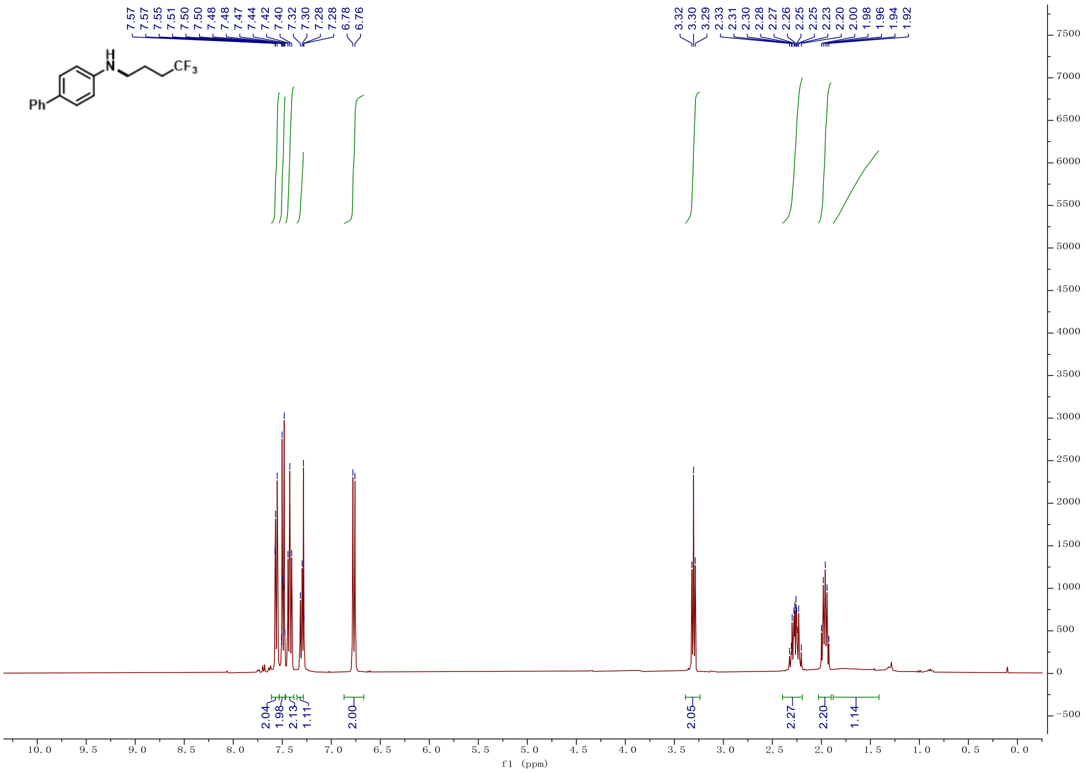

^{13}C NMR of Compound 50 (101 MHz, $CDCl_3$)

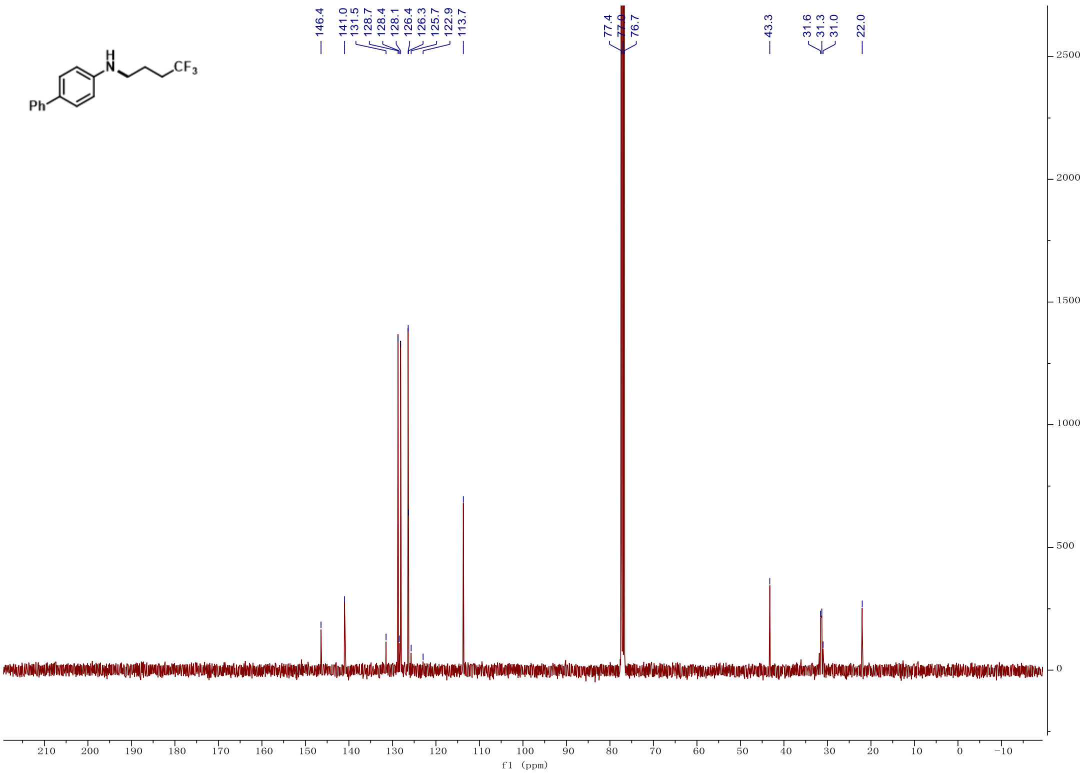

^{19}F NMR of Compound 50 (376 MHz, $CDCl_3$)

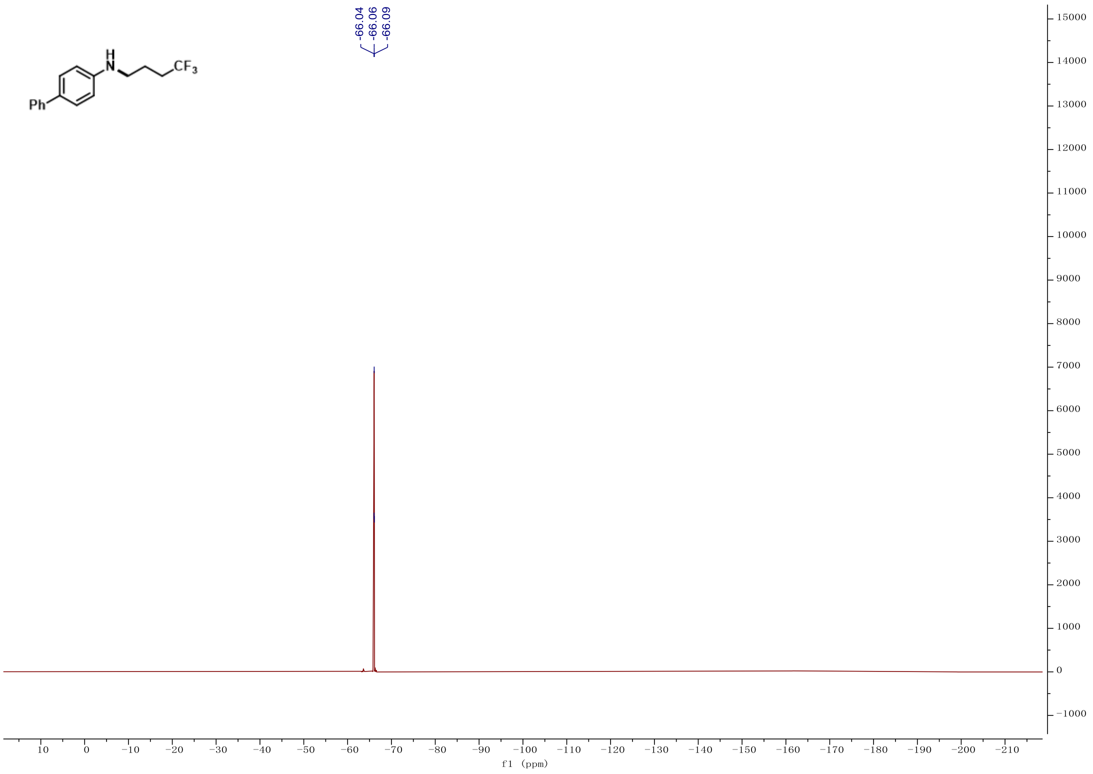

^{1}H NMR of Compound 51 (400 MHz, $CDCl_3$)

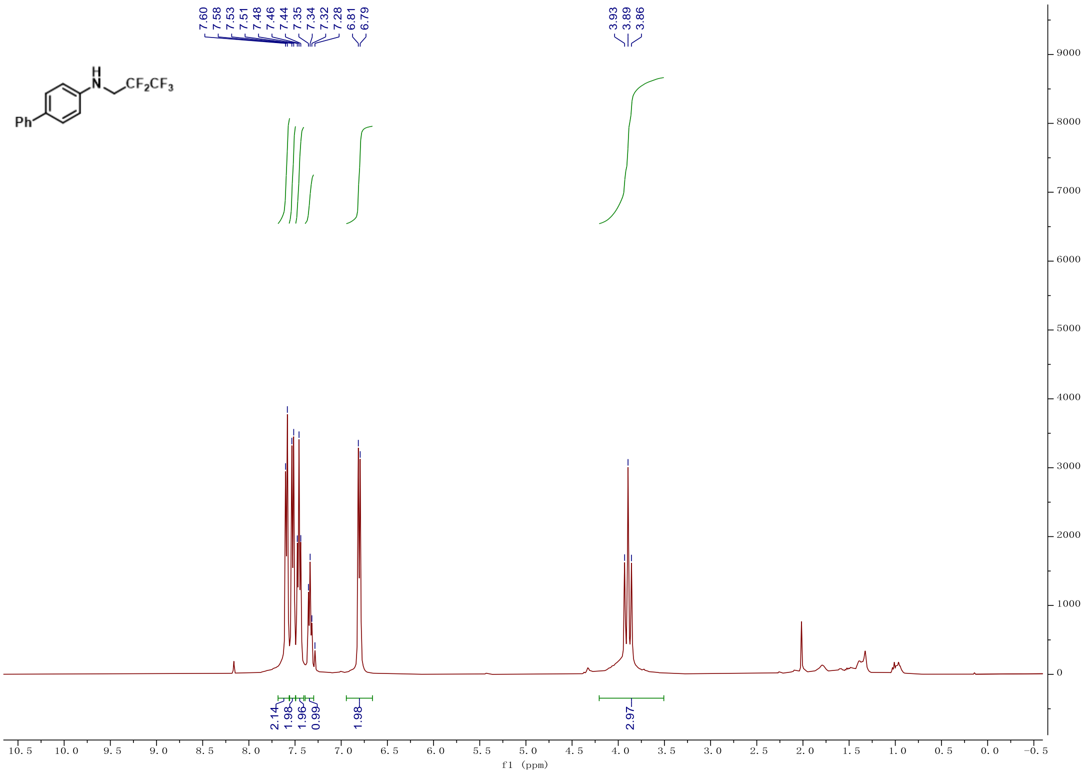

^{13}C NMR of Compound 51 (101 MHz, $CDCl_3$)

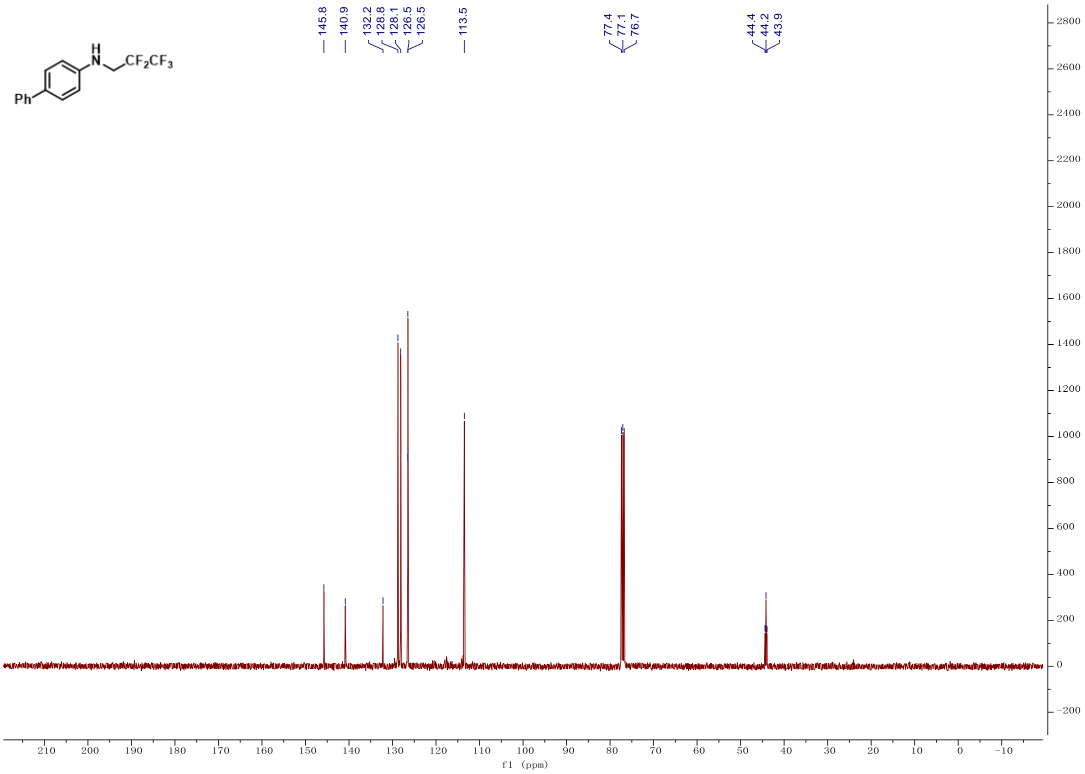

^{19}F NMR of Compound 51 (376 MHz, $CDCl_3$)

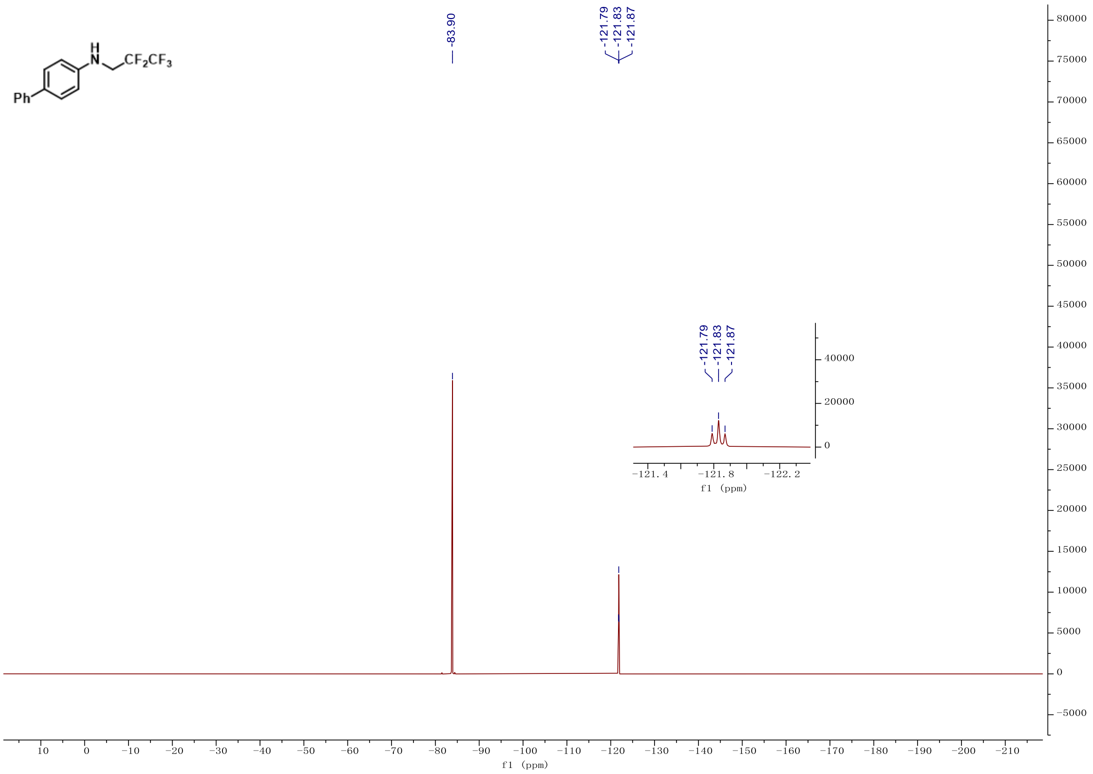

^{1}H NMR of Compound 52 (400 MHz, $CDCl_3$)

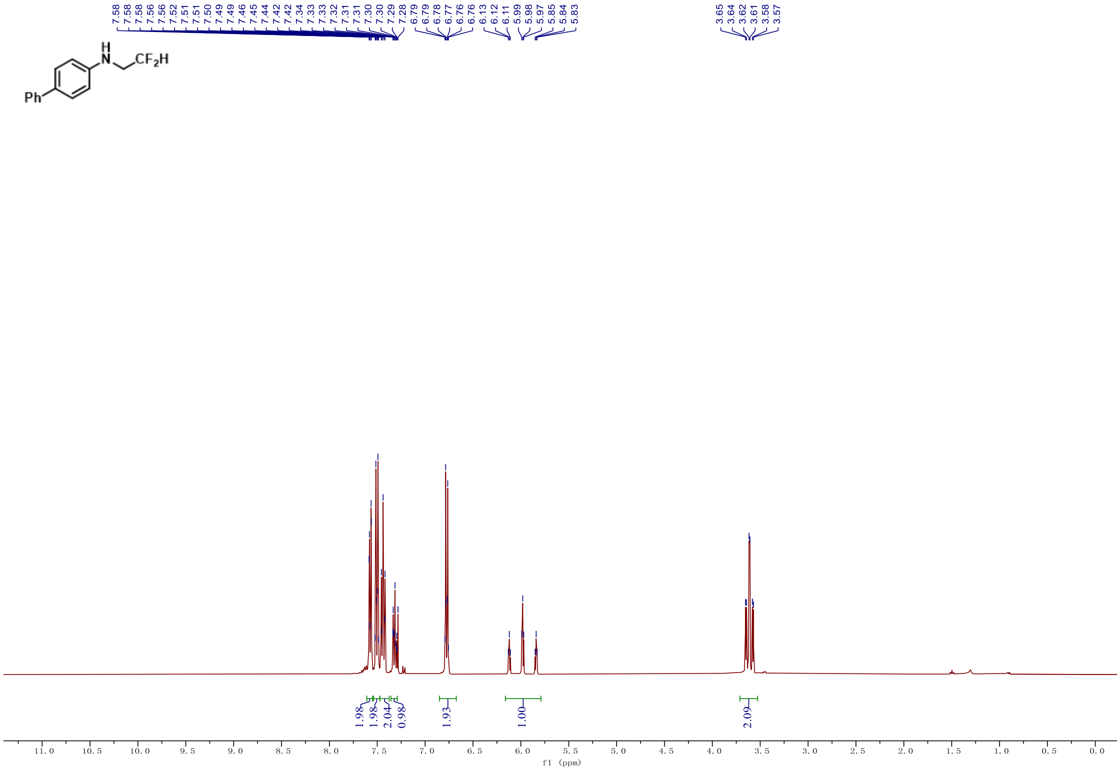

^{13}C NMR of Compound 52 (101 MHz, $CDCl_3$)

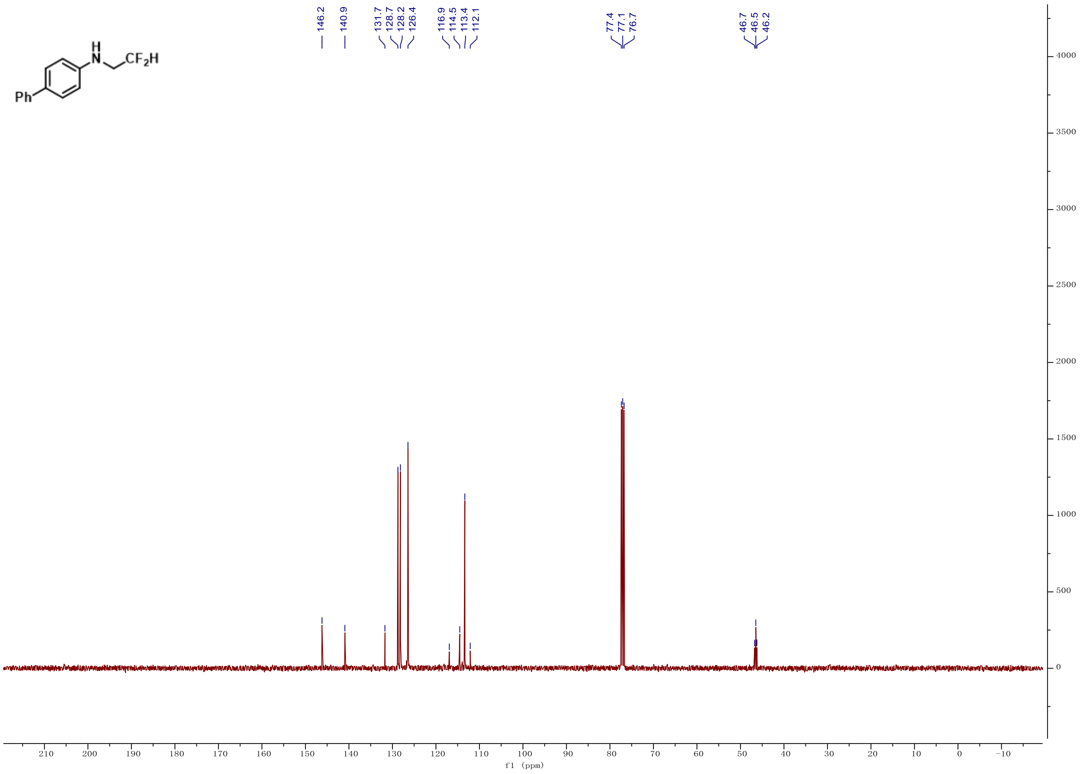

^{19}F NMR of Compound 52 (376 MHz, $CDCl_3$)

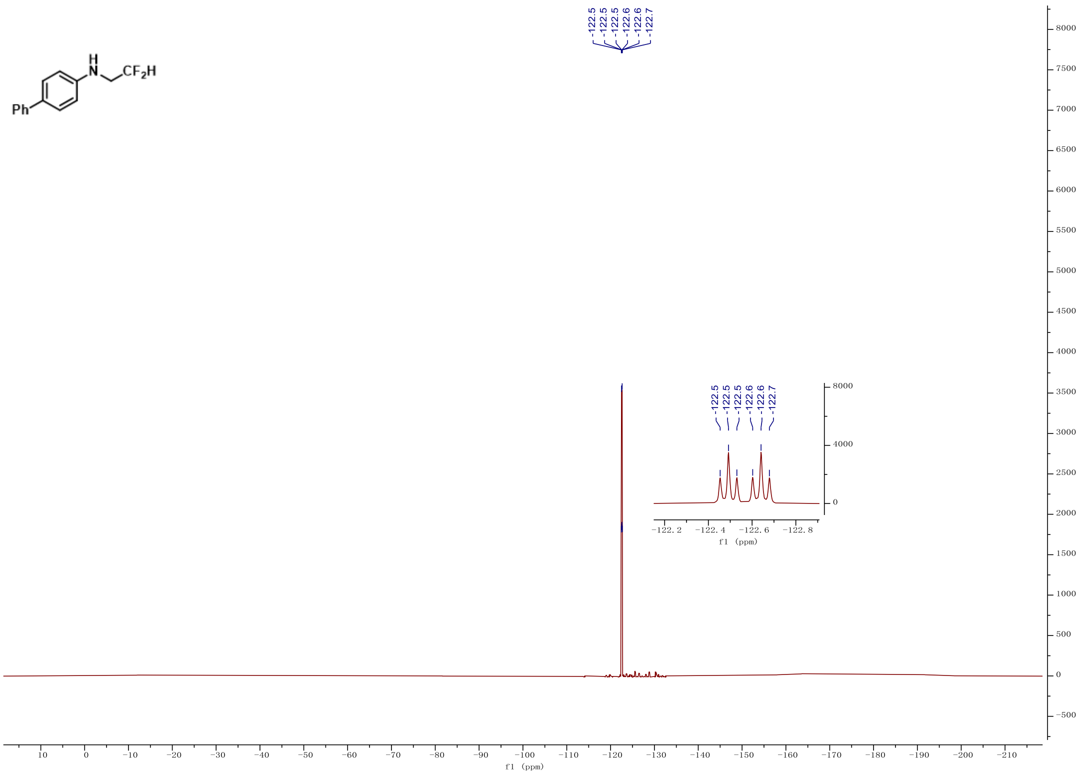

^{1}H NMR of Compound 53 (400 MHz, $CDCl_3$)

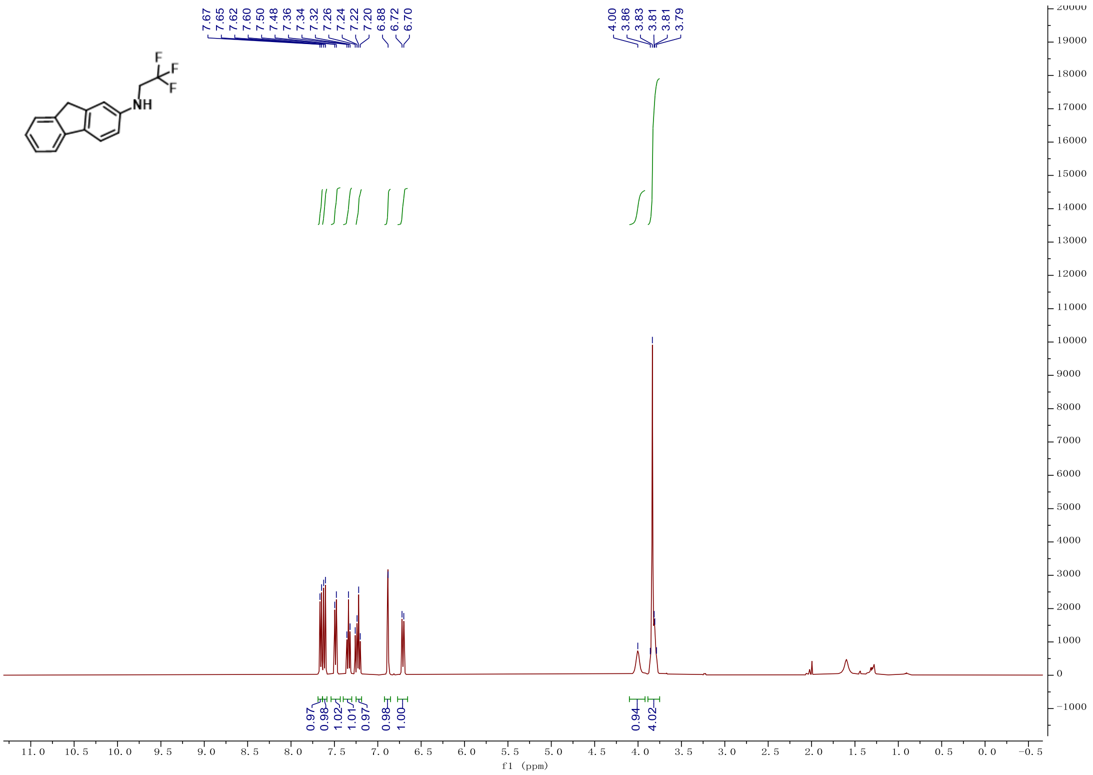

^{13}C NMR of Compound 53 (101 MHz, $CDCl_3$)

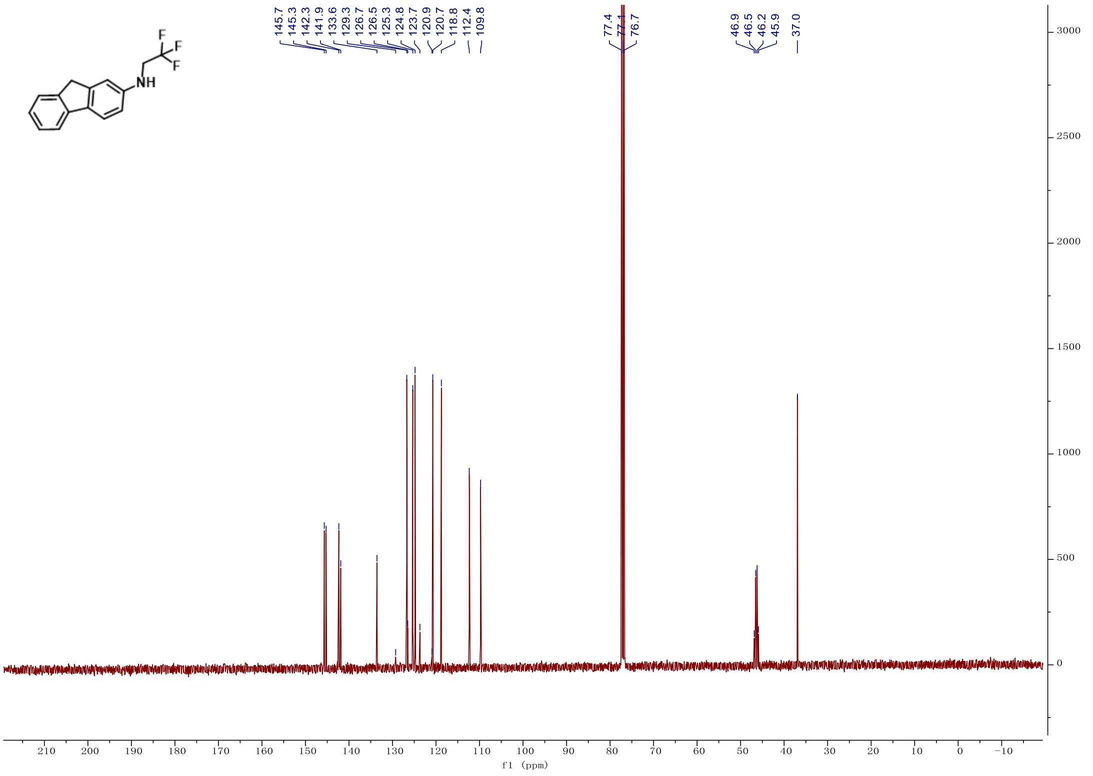

^{19}F NMR of Compound 53 (376 MHz, $CDCl_3$)

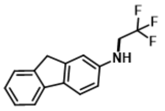

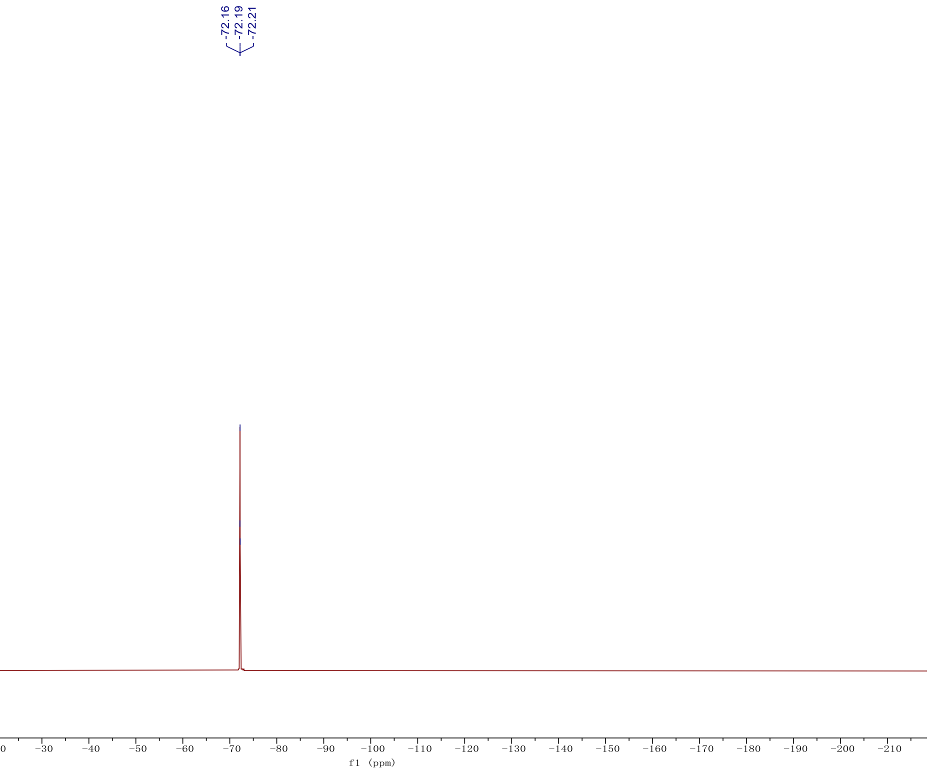

^{1}H NMR of Compound 54 (400 MHz, $CDCl_3$)

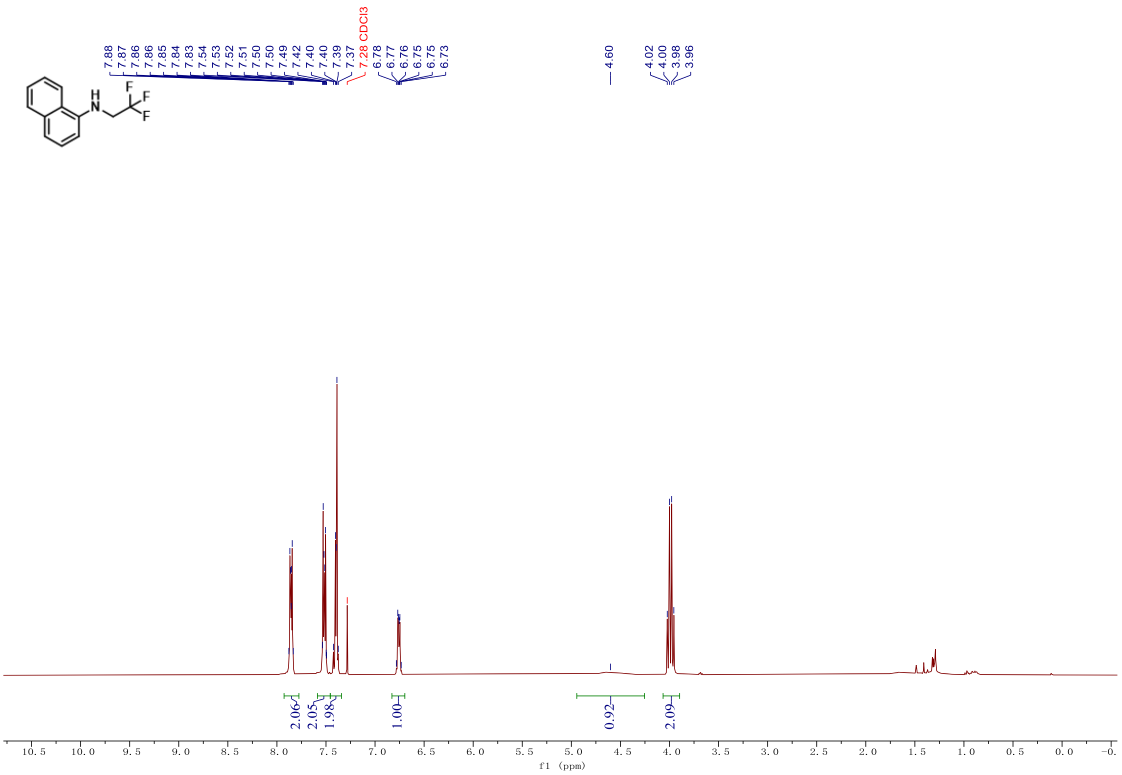

^{13}C NMR of Compound 54 (101 MHz, $CDCl_3$)

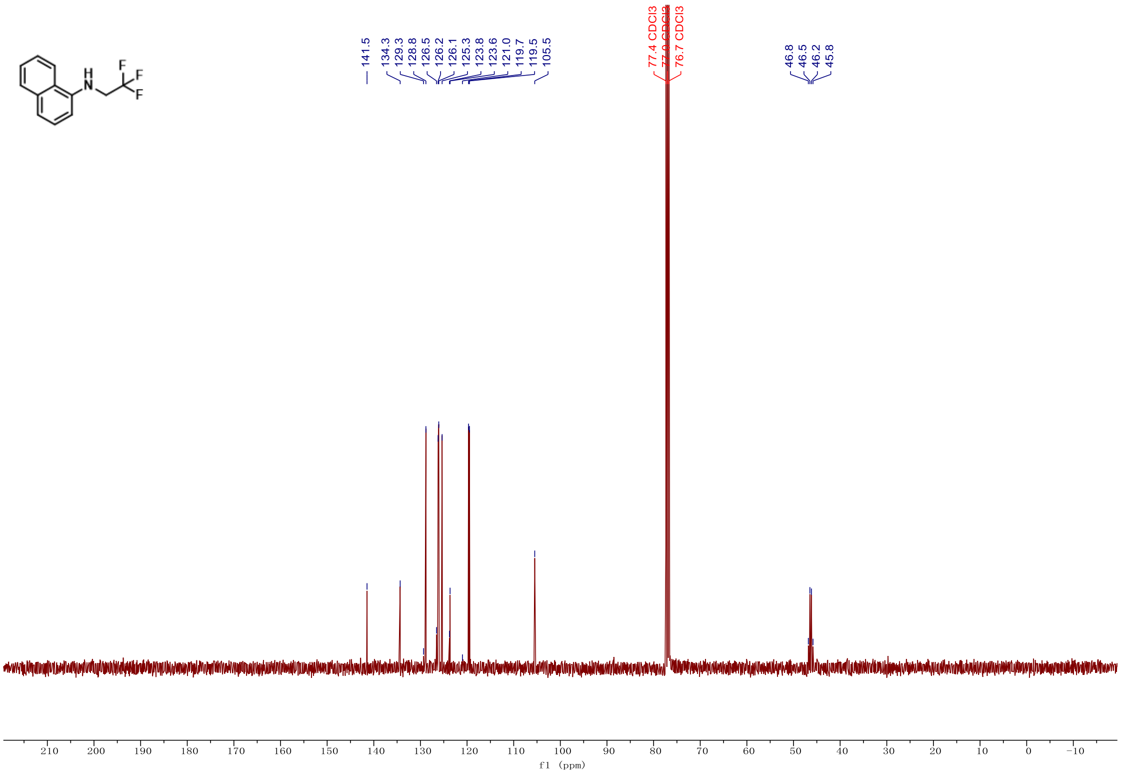

\

^{19}F NMR of Compound 54 (376 MHz, $CDCl_3$)

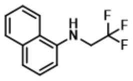

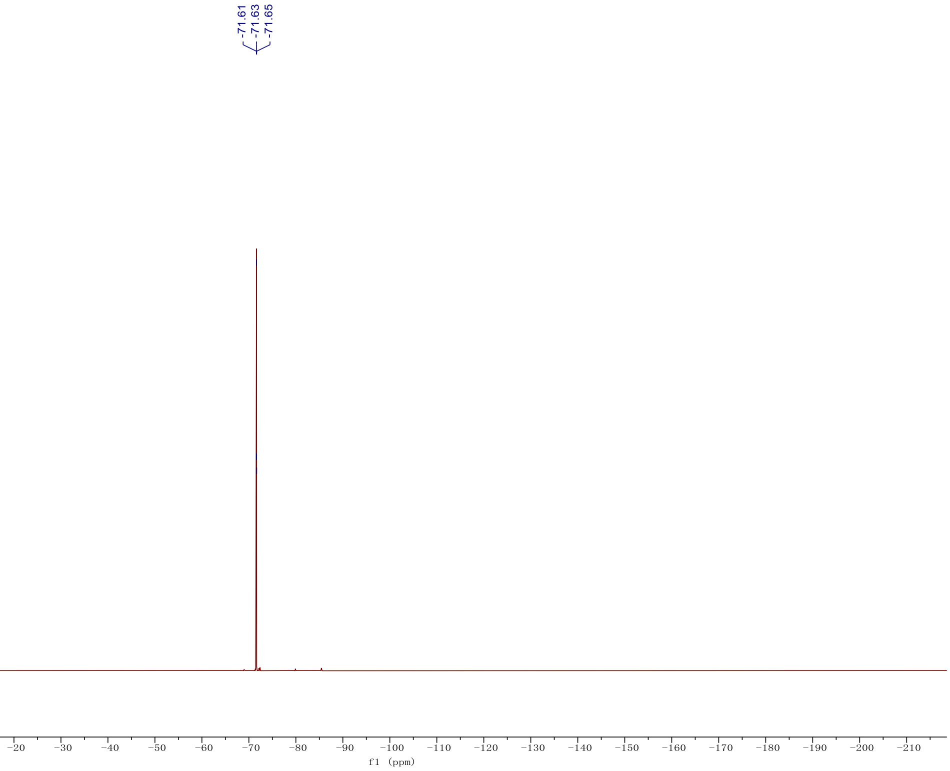

^{1}H NMR of Compound 55 (400 MHz, $CDCl_3$)

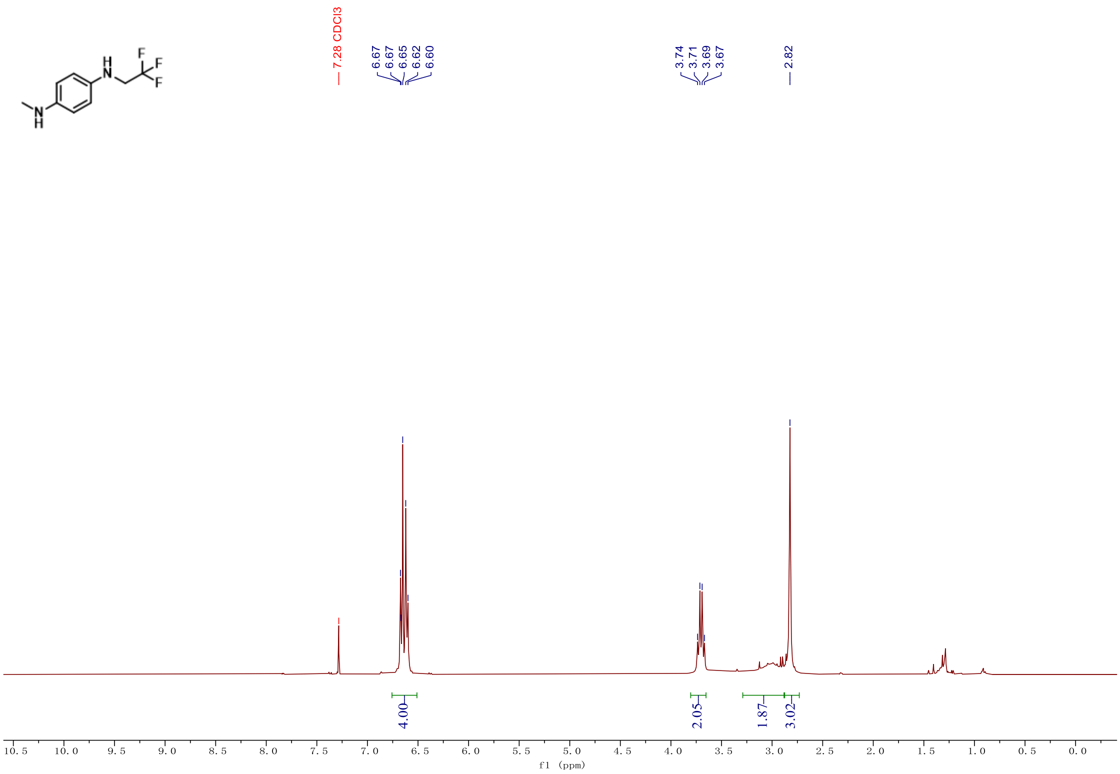

^{13}C NMR of Compound 55 (101 MHz, $CDCl_3$)

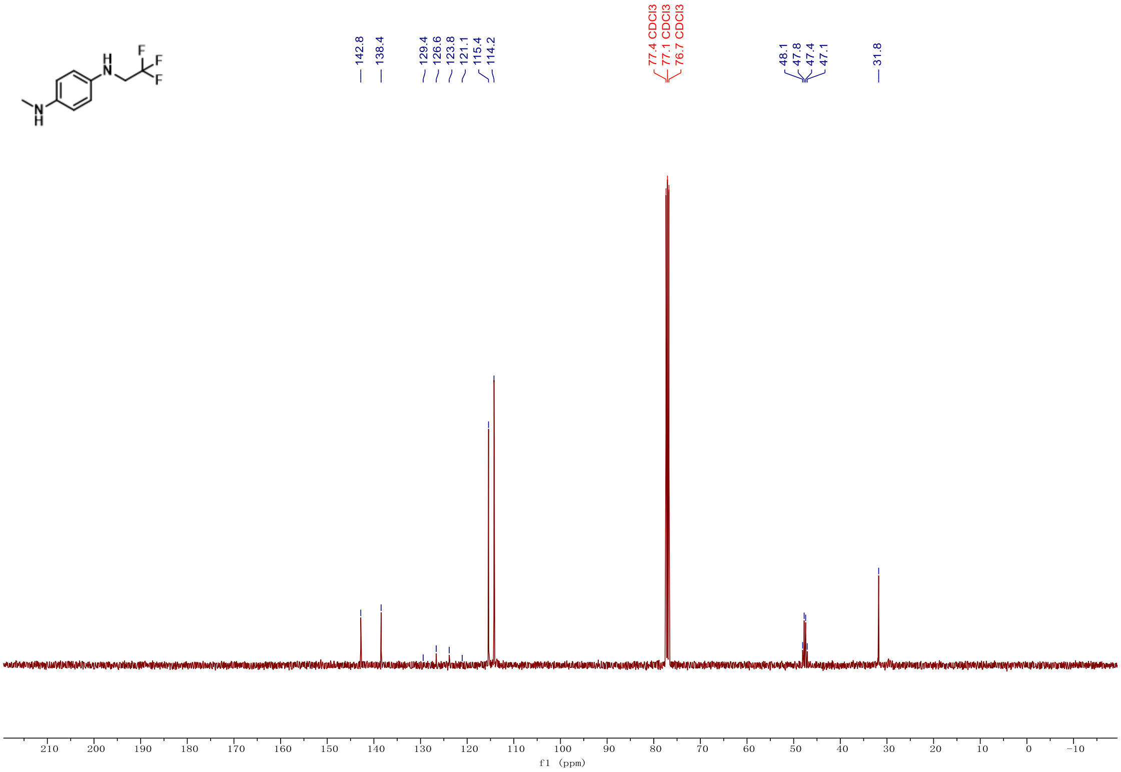

^{19}F NMR of Compound 55 (376 MHz, $CDCl_3$)

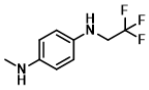

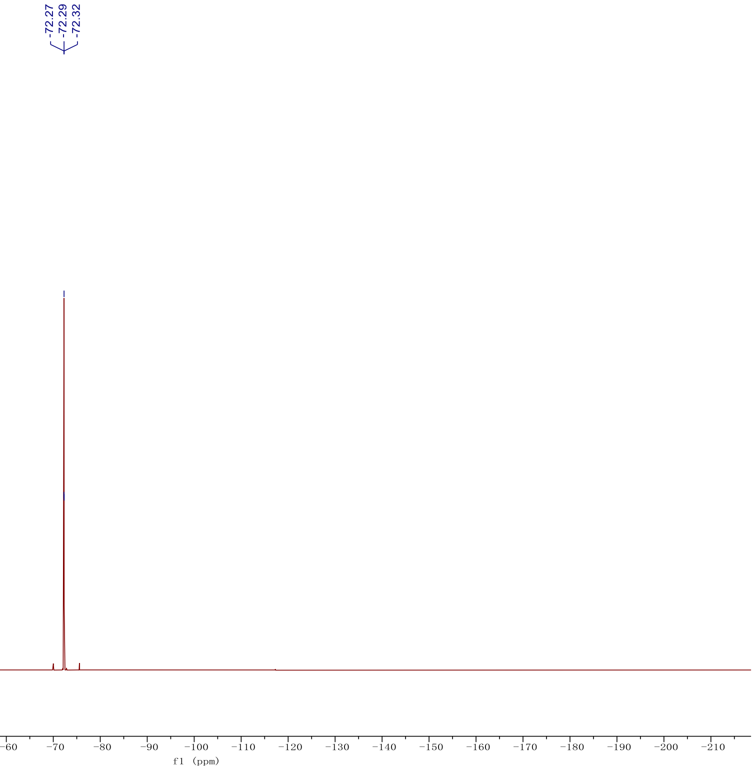

^{1}H NMR of Compound 56 (400 MHz, $CDCl_3$)

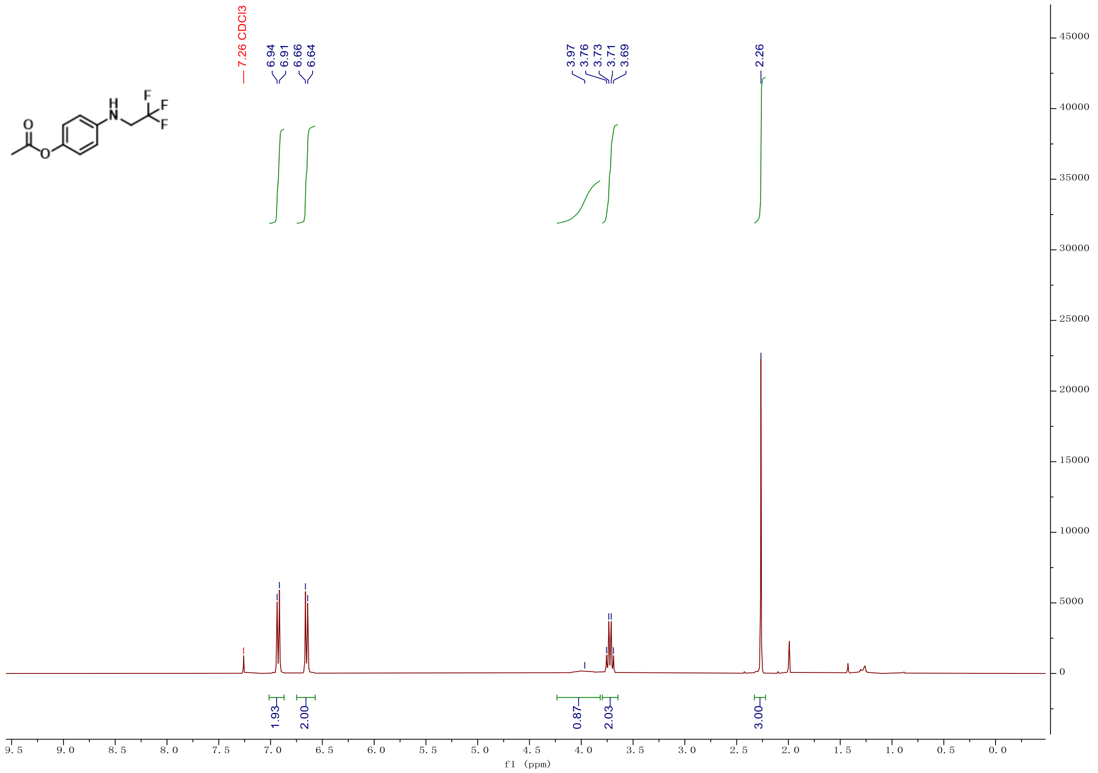

^{13}C NMR of Compound 56 (101 MHz, $CDCl_3$)

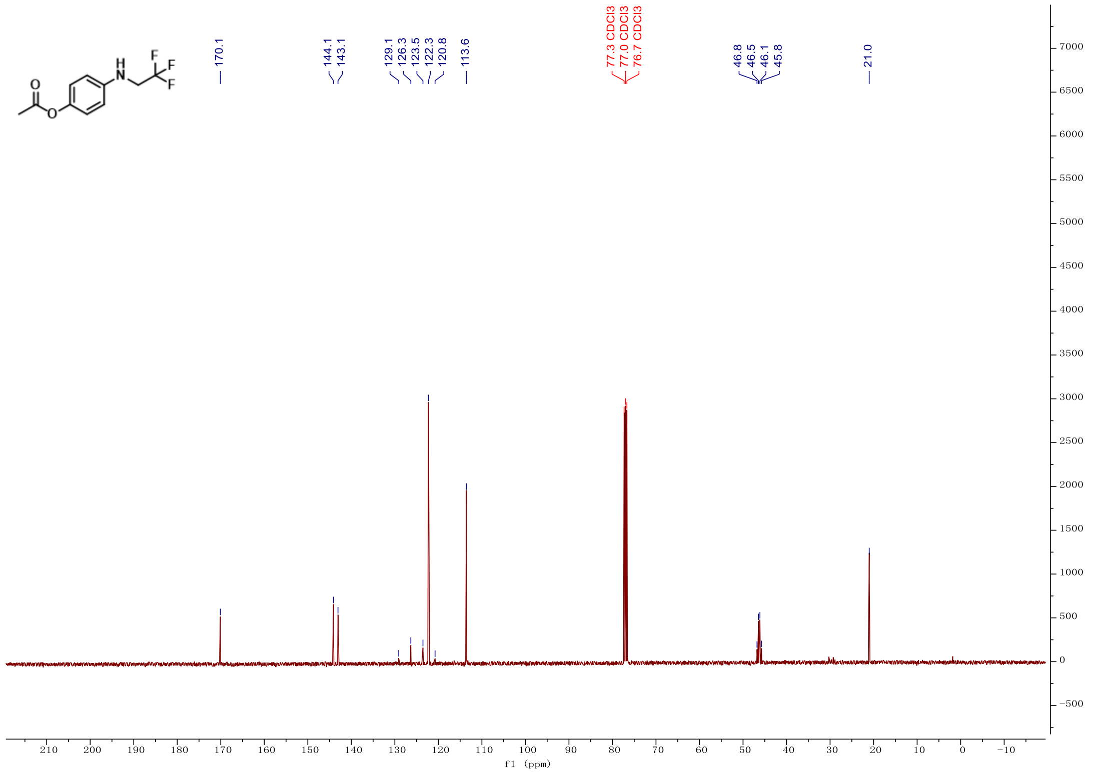

^{19}F NMR of Compound 56 (376 MHz, $CDCl_3$)

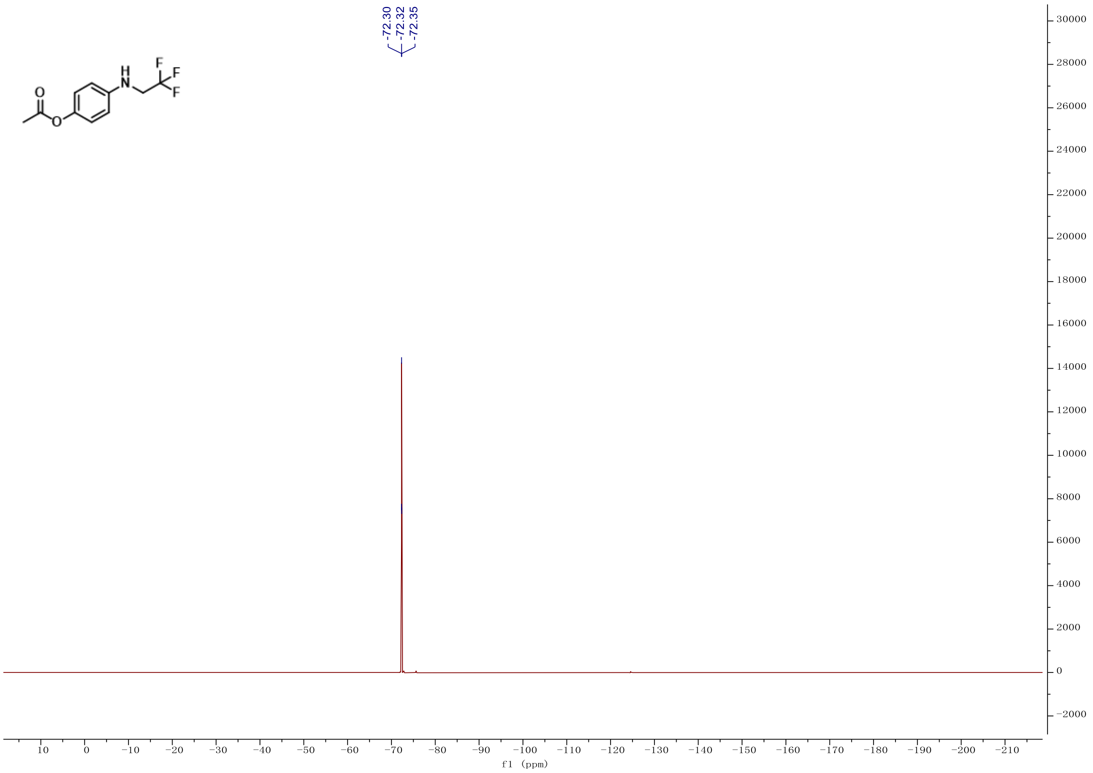

^{1}H NMR of Compound 57 (400 MHz, $CDCl_3$)

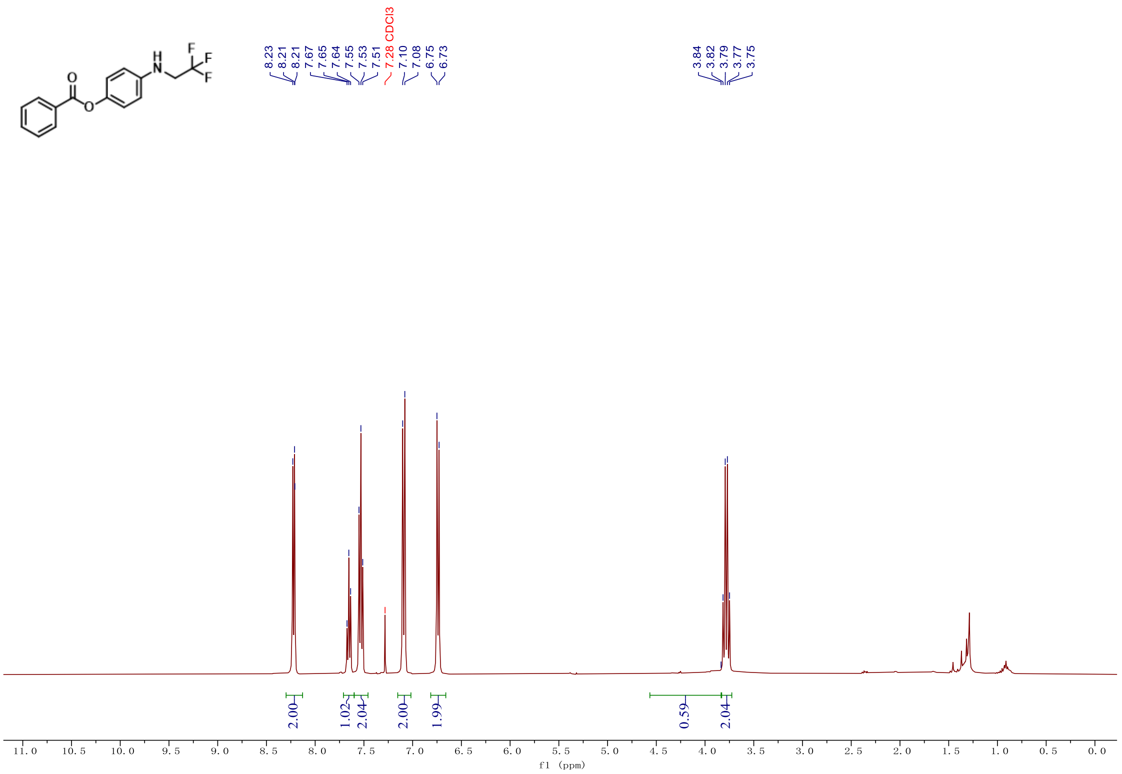

^{13}C NMR of Compound 57 (101 MHz, $CDCl_3$)

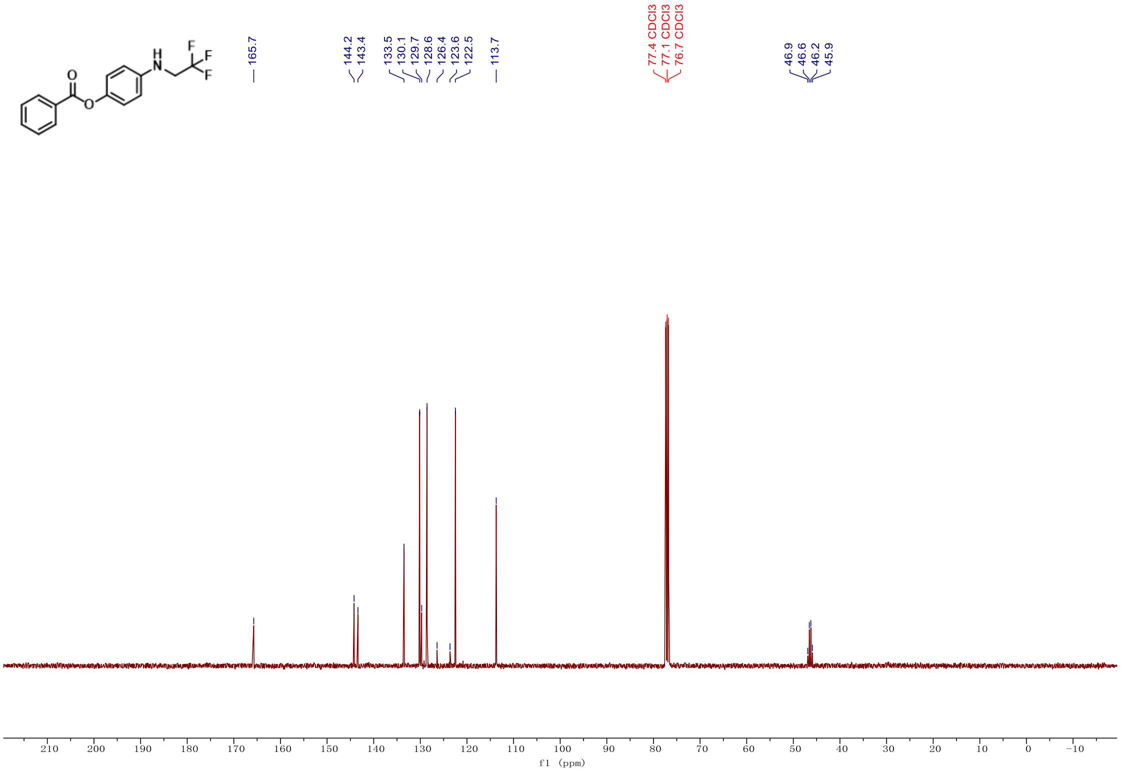

^{19}F NMR of Compound 57 (376 MHz, $CDCl_3$)

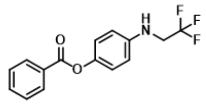

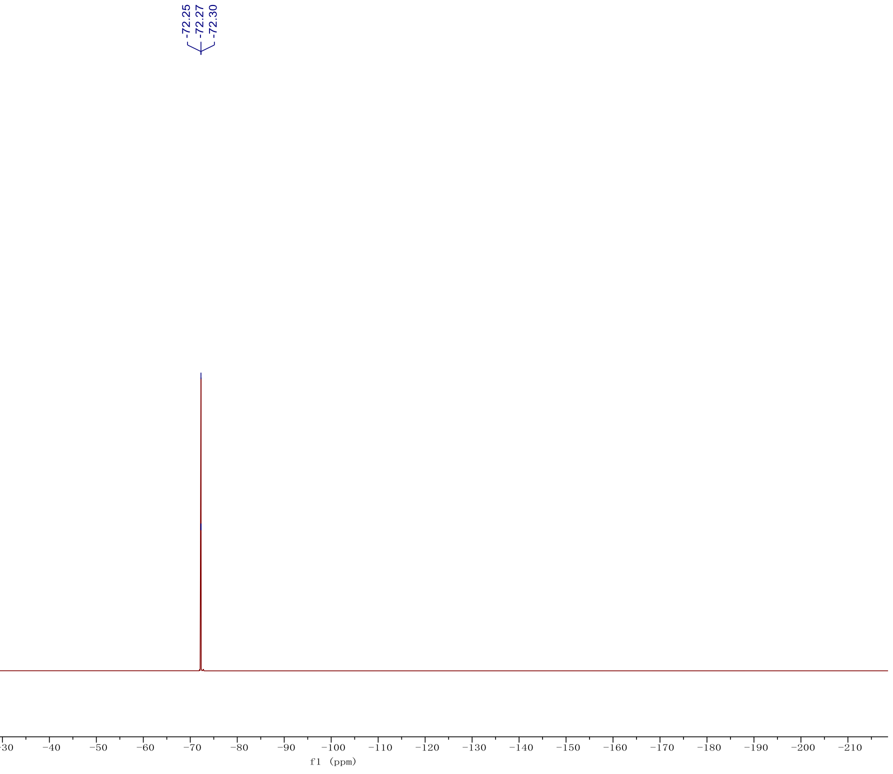

^{1}H NMR of Compound 58 (400 MHz, DMSO-d^{6})

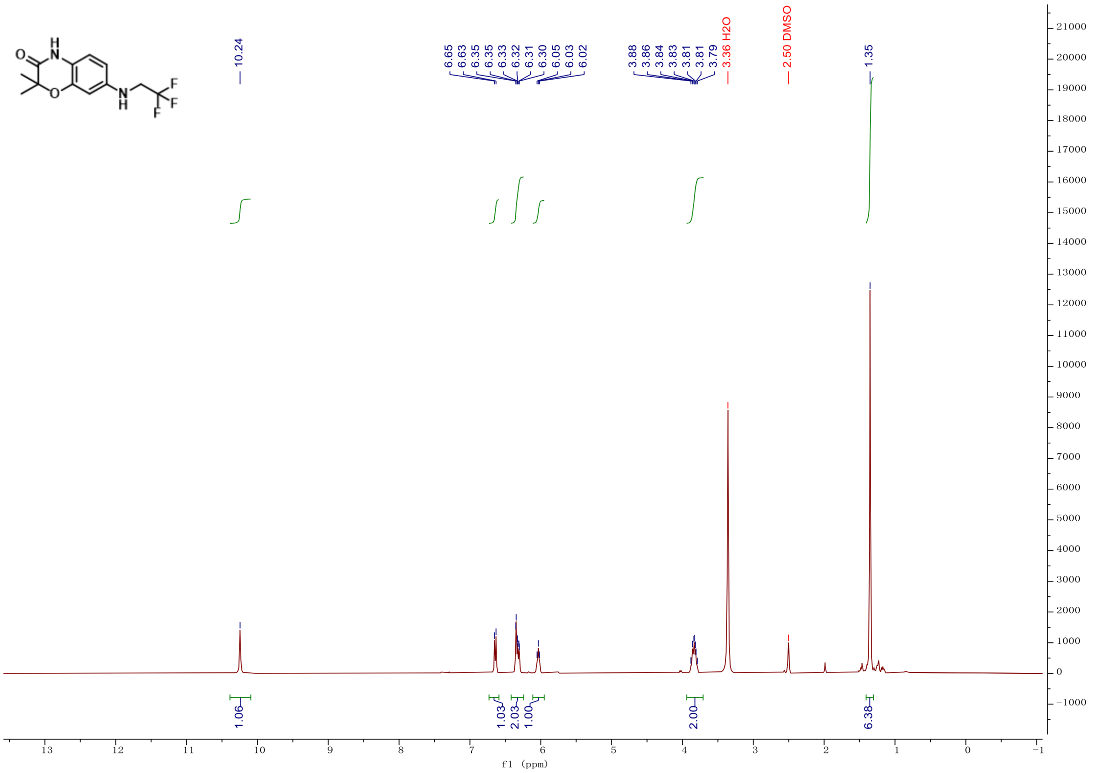

^{13}C NMR of Compound 58 (101 MHz, DMSO-d^{6})

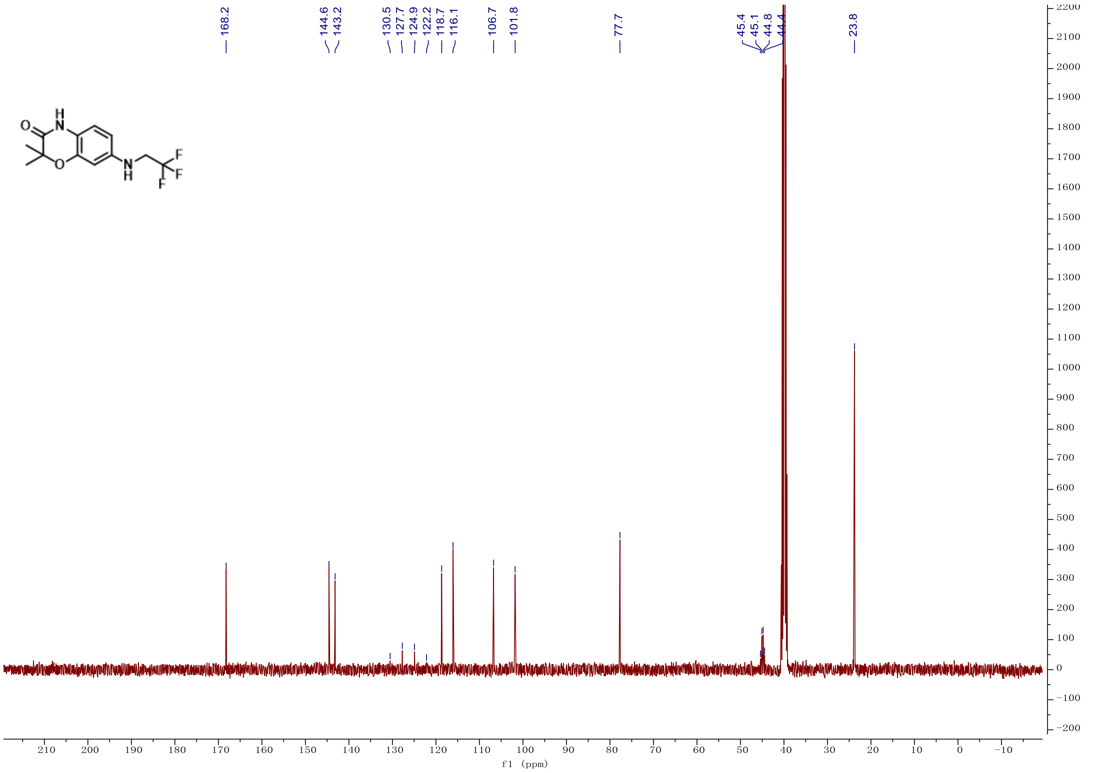

^{19}F NMR of Compound 58 (376 MHz, DMSO-d^6)

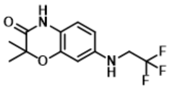

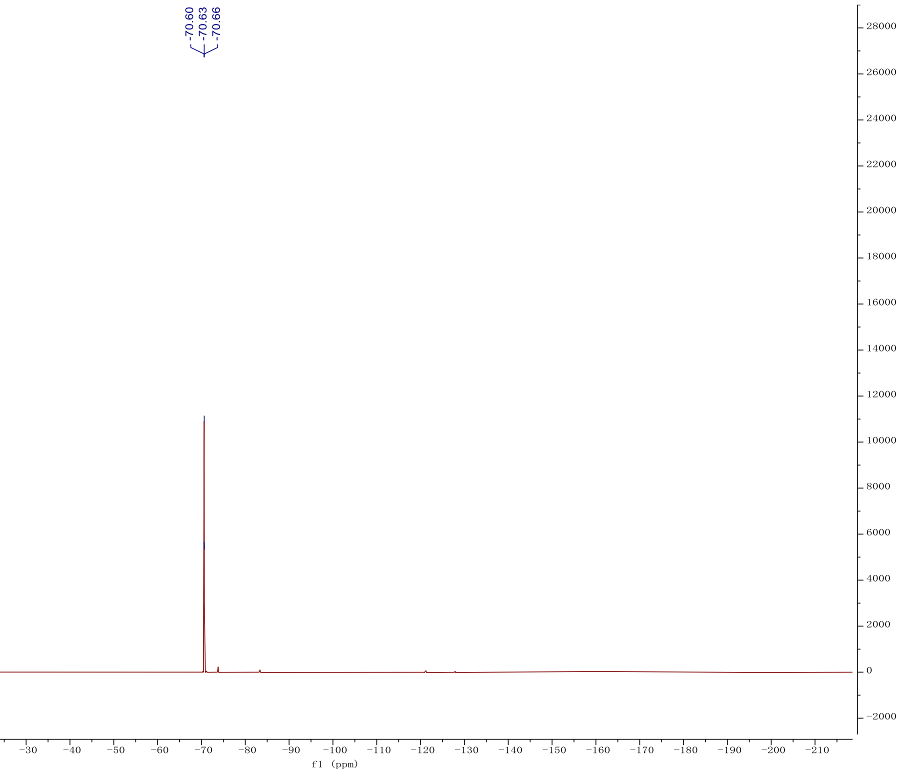

^{1}H NMR of Compound 59 (400 MHz, DMSO-d^{6})

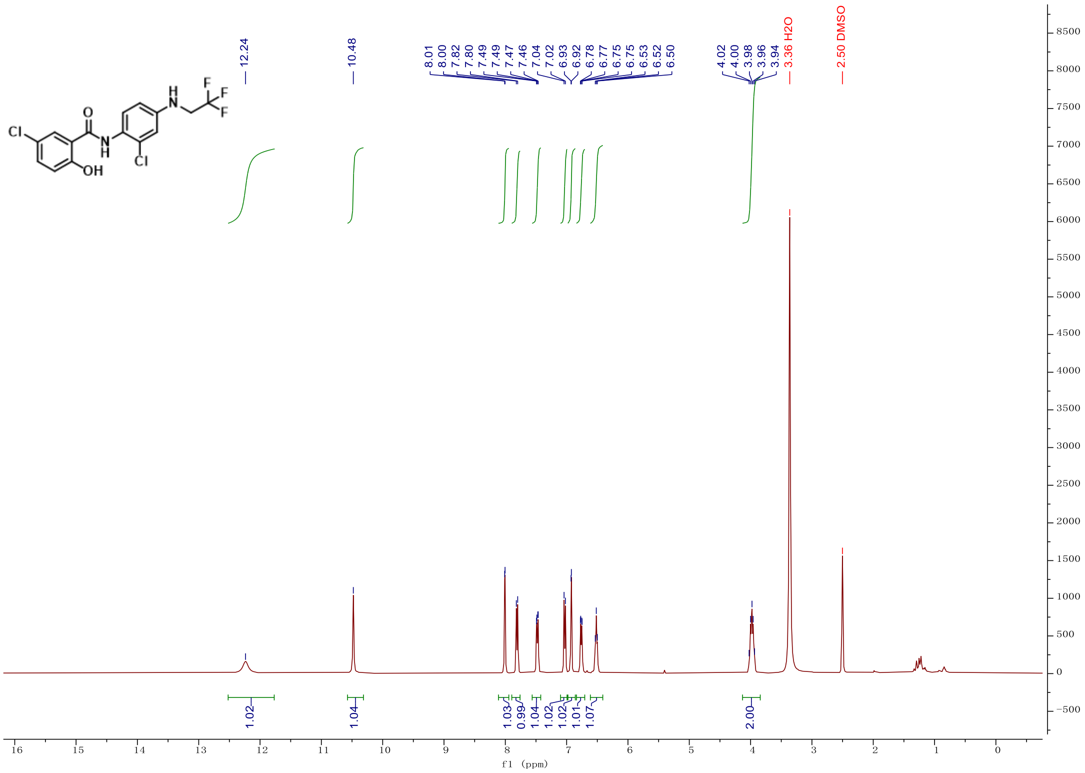

^{13}C NMR of Compound 59 (101 MHz,DMSO-d^{6})

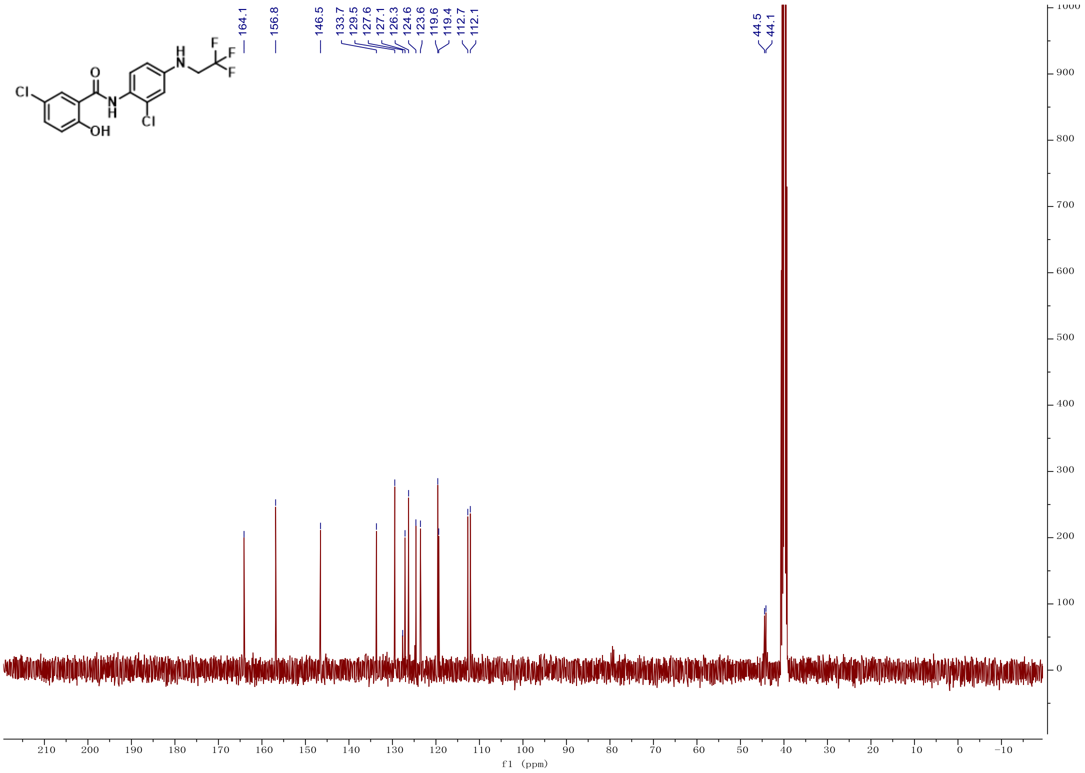

^{19}F NMR of Compound 59 (376 MHz, DMSO-d^6)

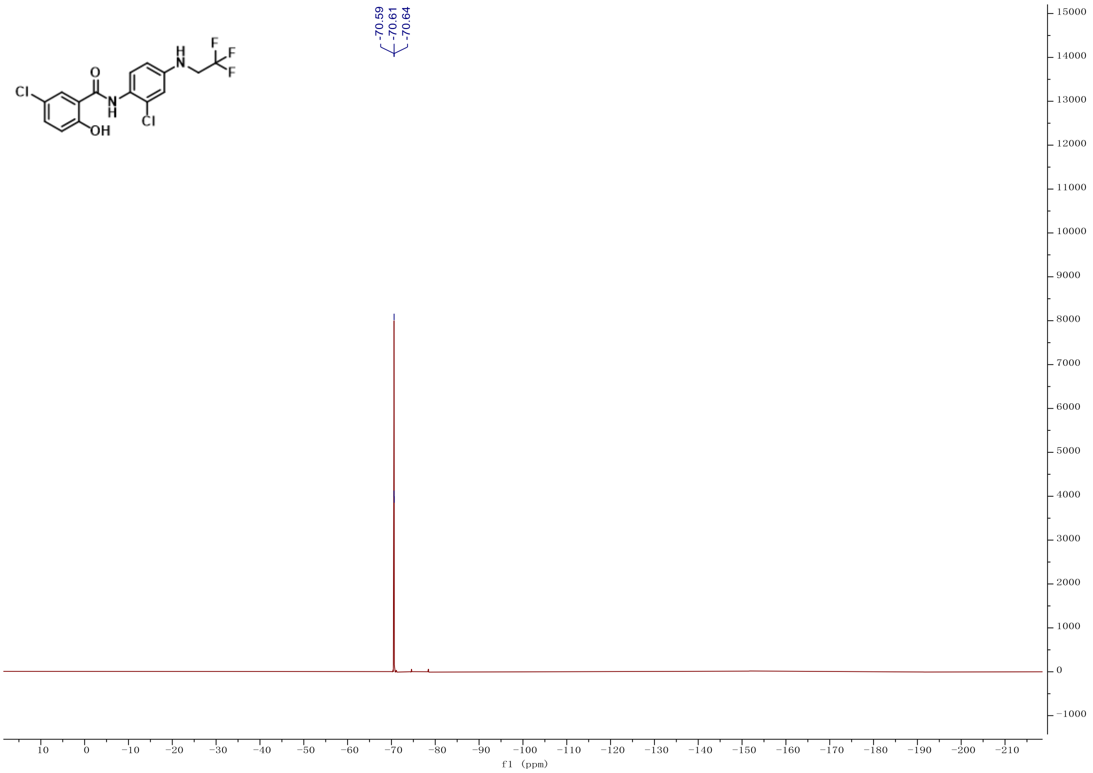

^{1}H NMR of Compound 60 (400 MHz, DMSO-d^{6})

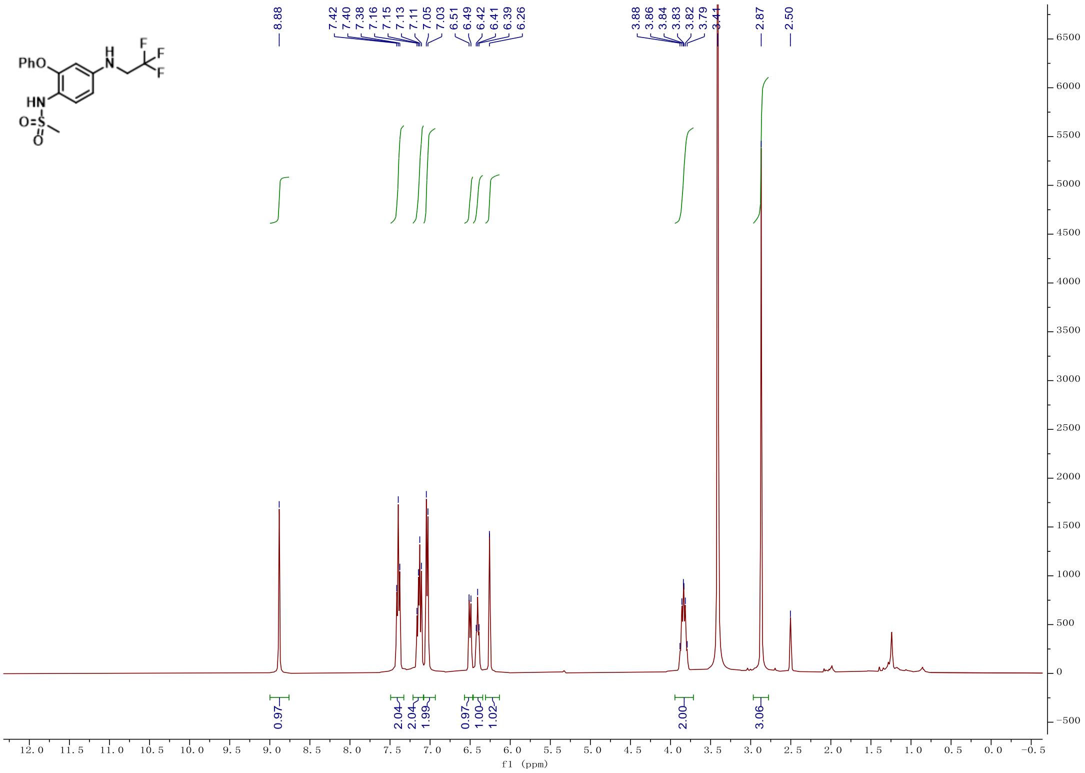

^{13}C NMR of Compound 60(101 MHz, DMSO-d^{6})

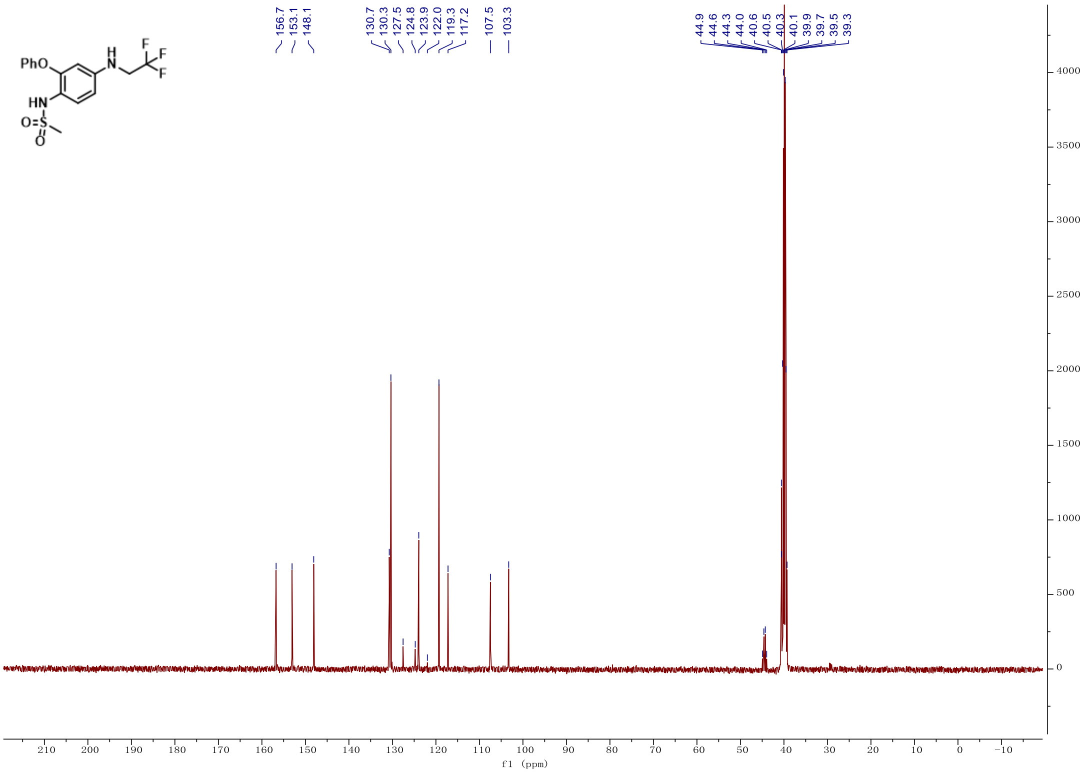

^{19}F NMR of Compound 60(376 MHz, DMSO-d^6)

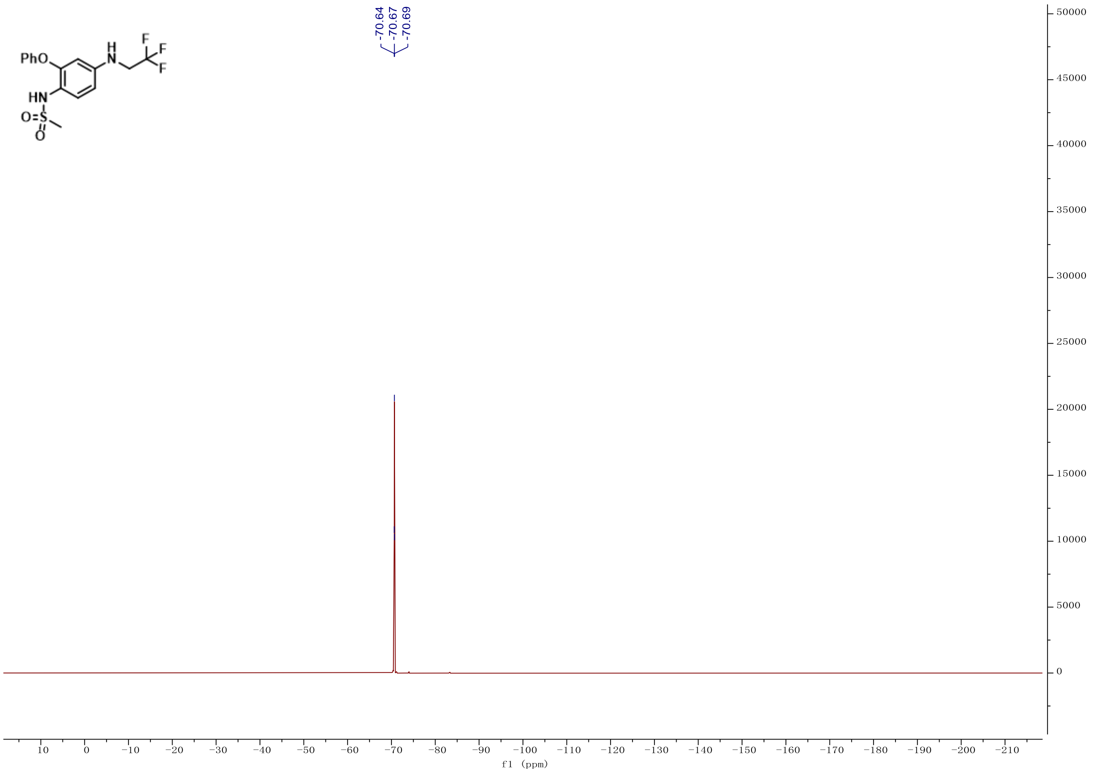

^{1}H NMR of Compound 61(400 MHz, $CDCl_3$)

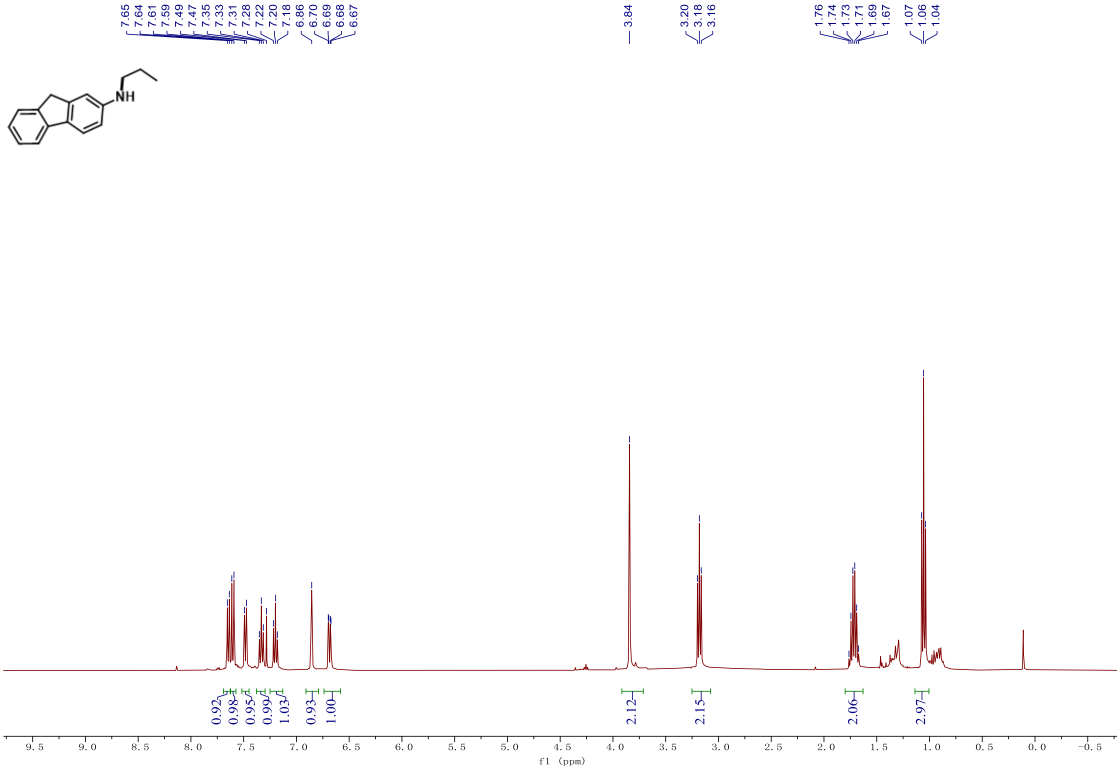

^{13}C NMR of Compound 61(101 MHz, $CDCl_3$)

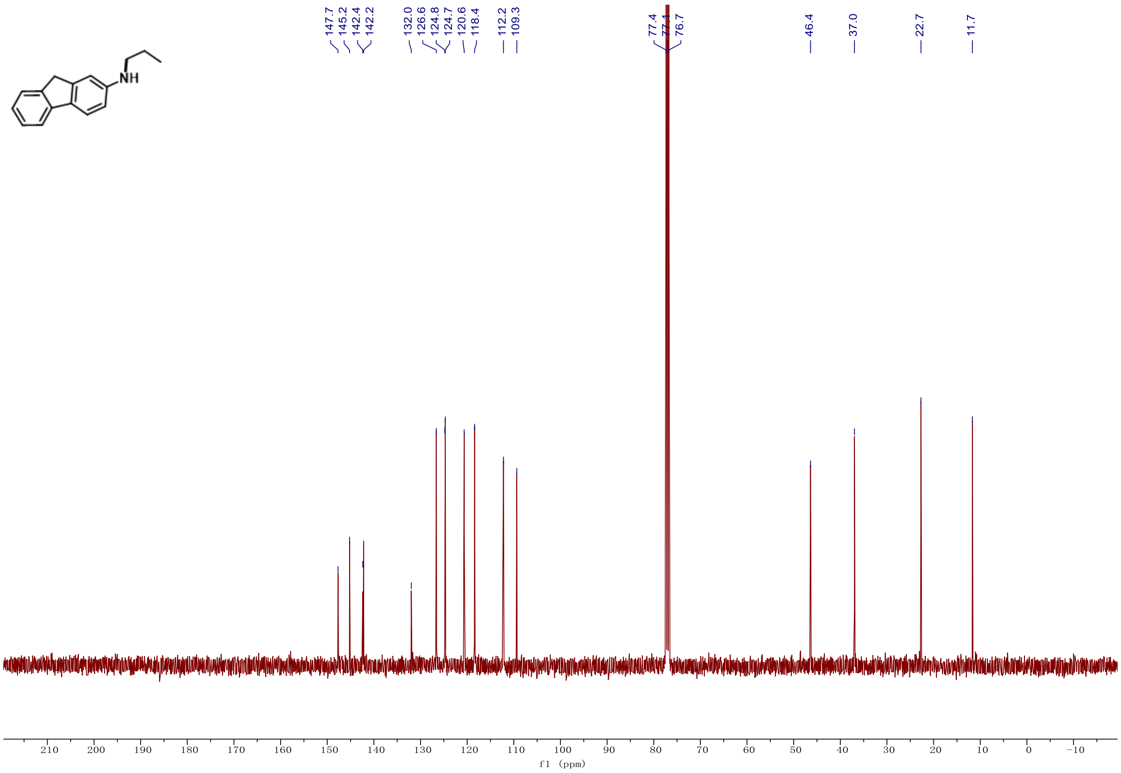

^{1}H NMR of Compound 62(400 MHz, $CDCl_3$)

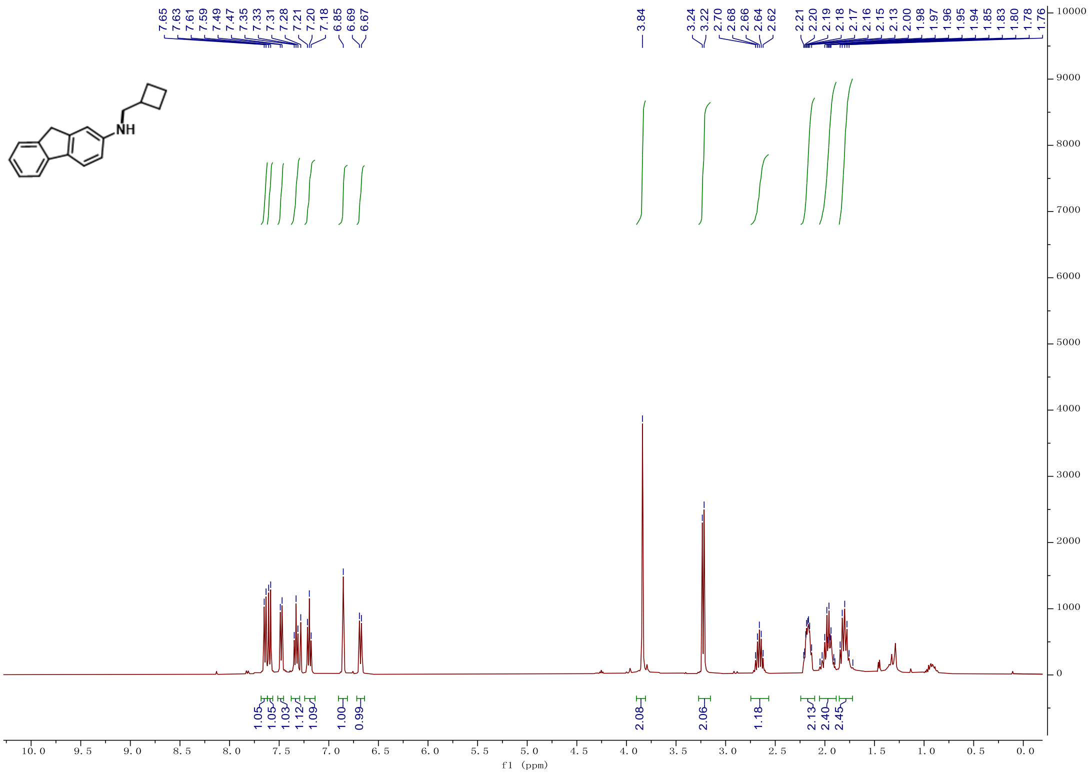

^{13}C NMR of Compound 62(101 MHz, $CDCl_3$)

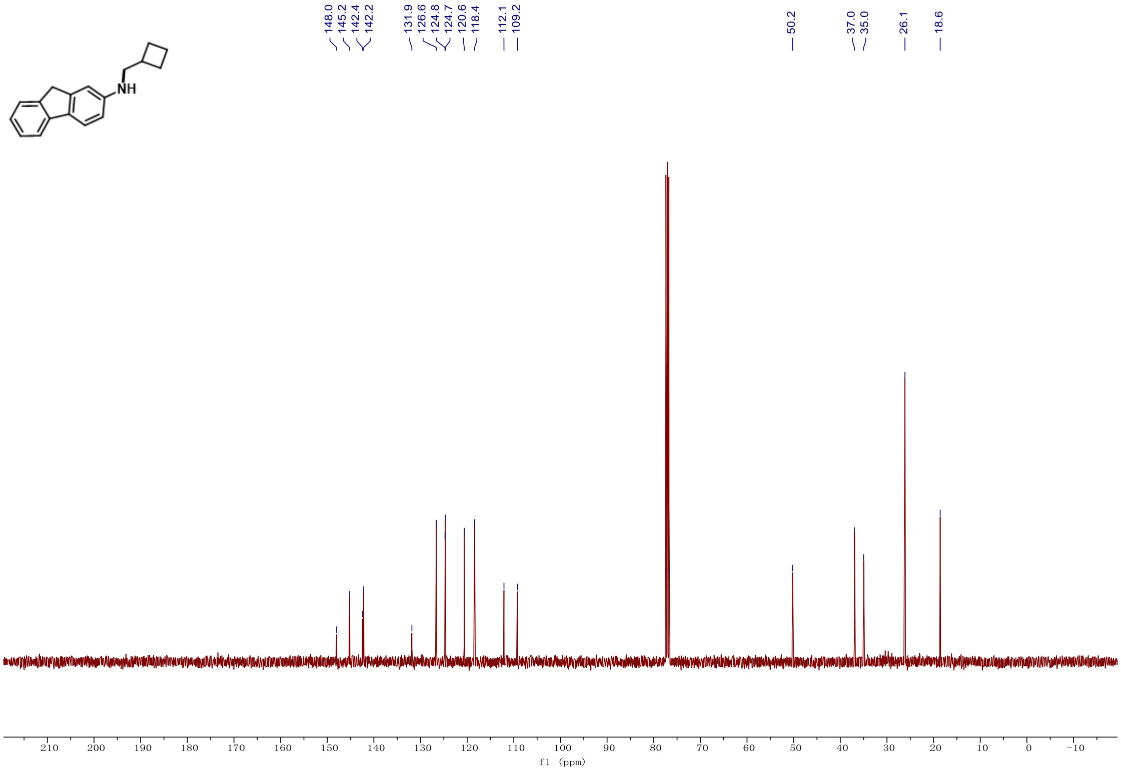

^{1}H NMR of Compound 63(400 MHz, $CDCl_3$)

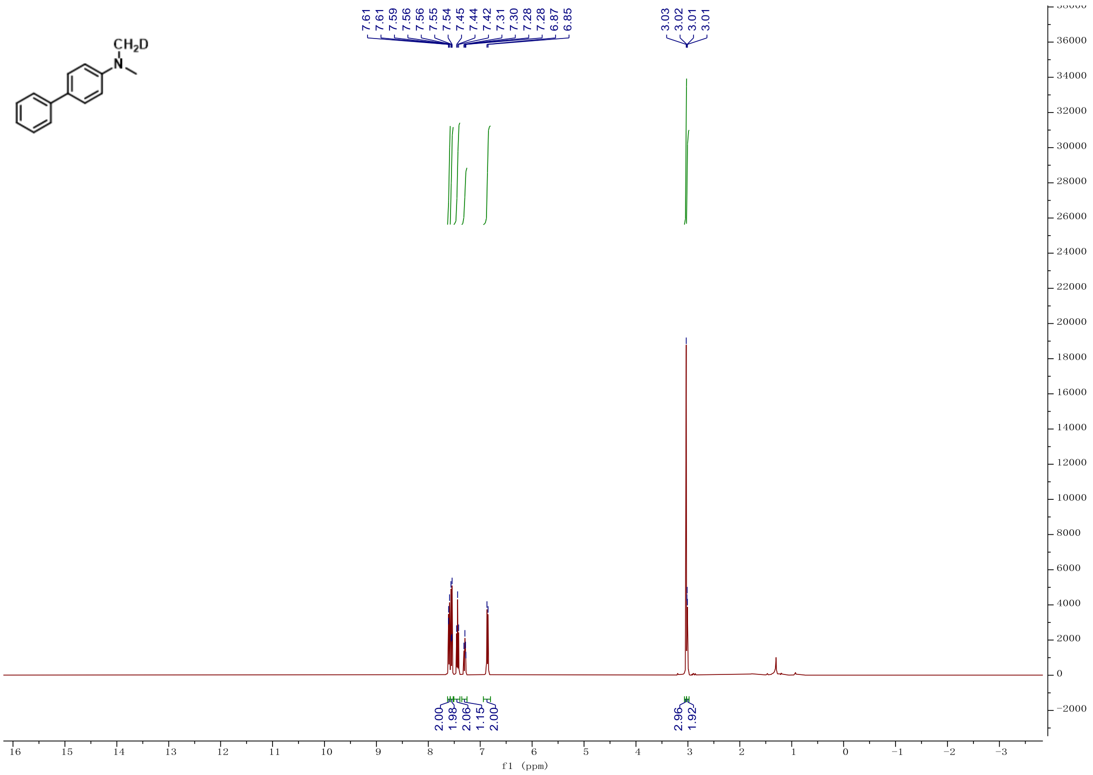

^{13}C NMR of Compound 63(101 MHz, $CDCl_3$)

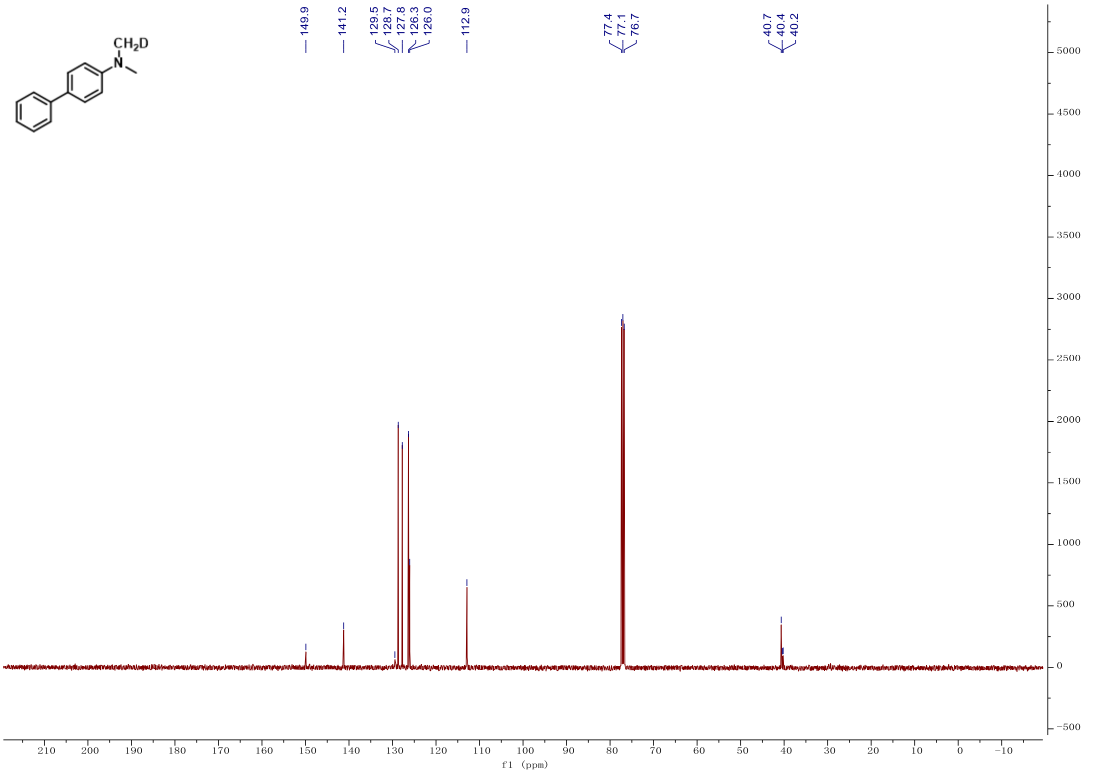

^{1}H NMR of Compound 64 (400 MHz, $CDCl_3$)

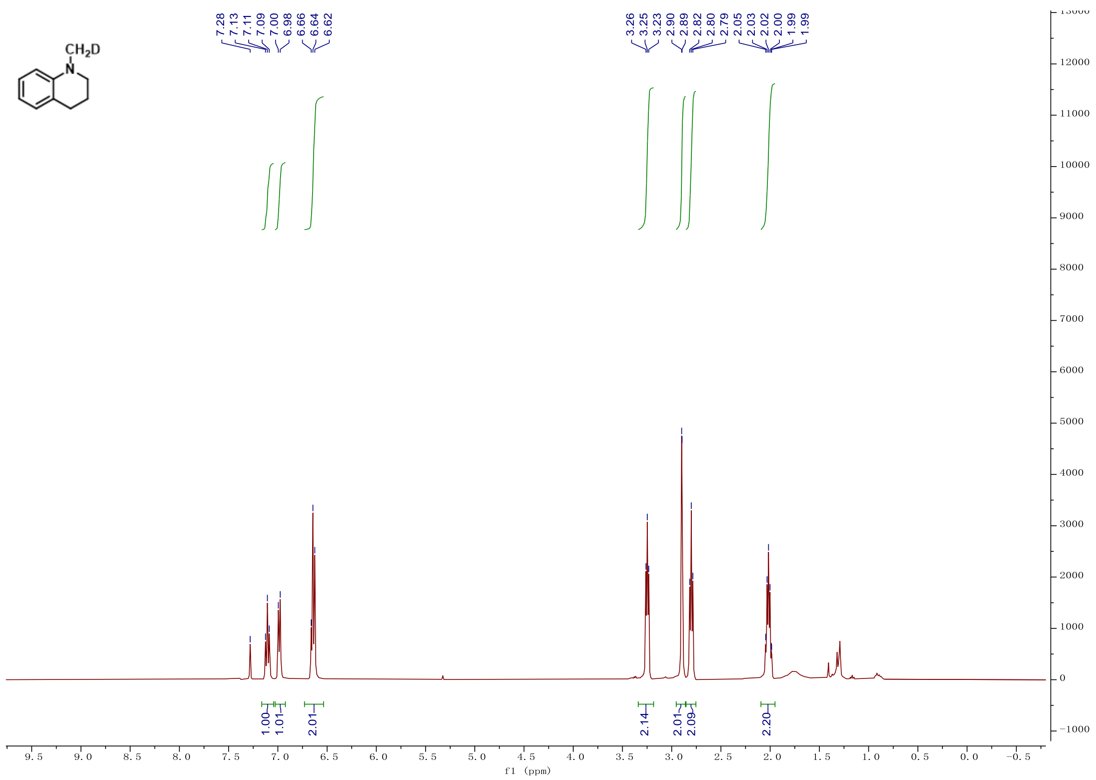

^{13}C NMR of Compound 64 (101 MHz, $CDCl_3$)

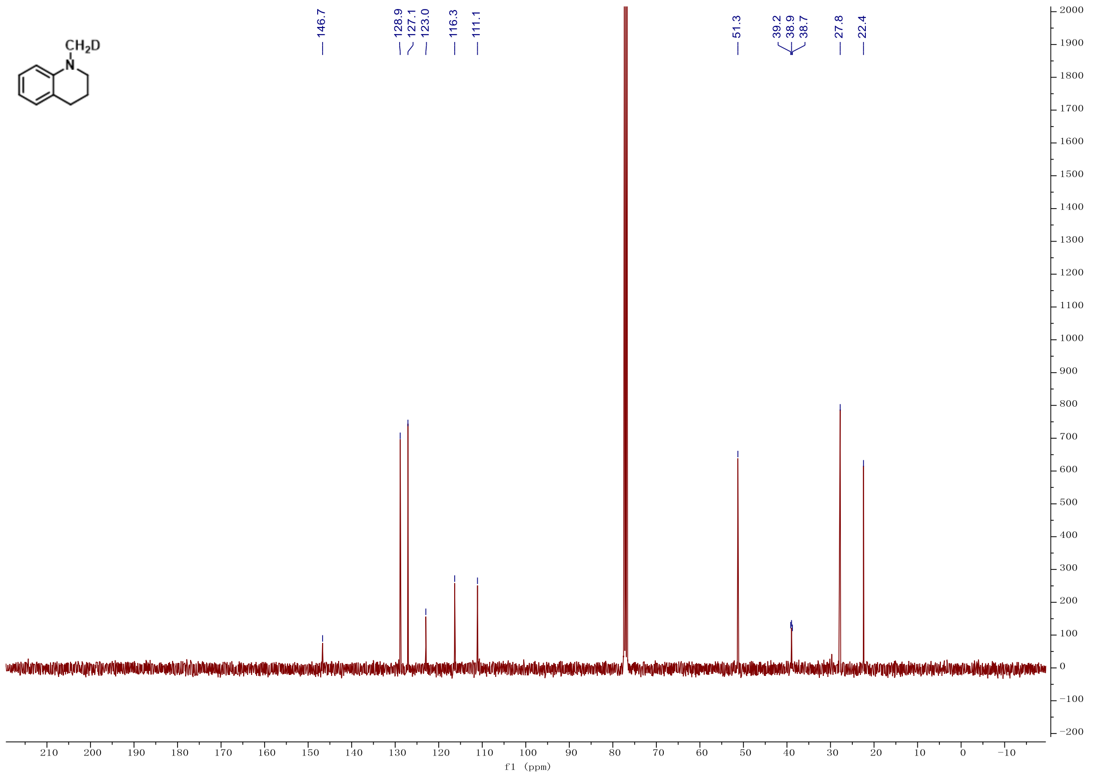

^{1}H NMR of Compound 65 (400 MHz, $CDCl_3$)

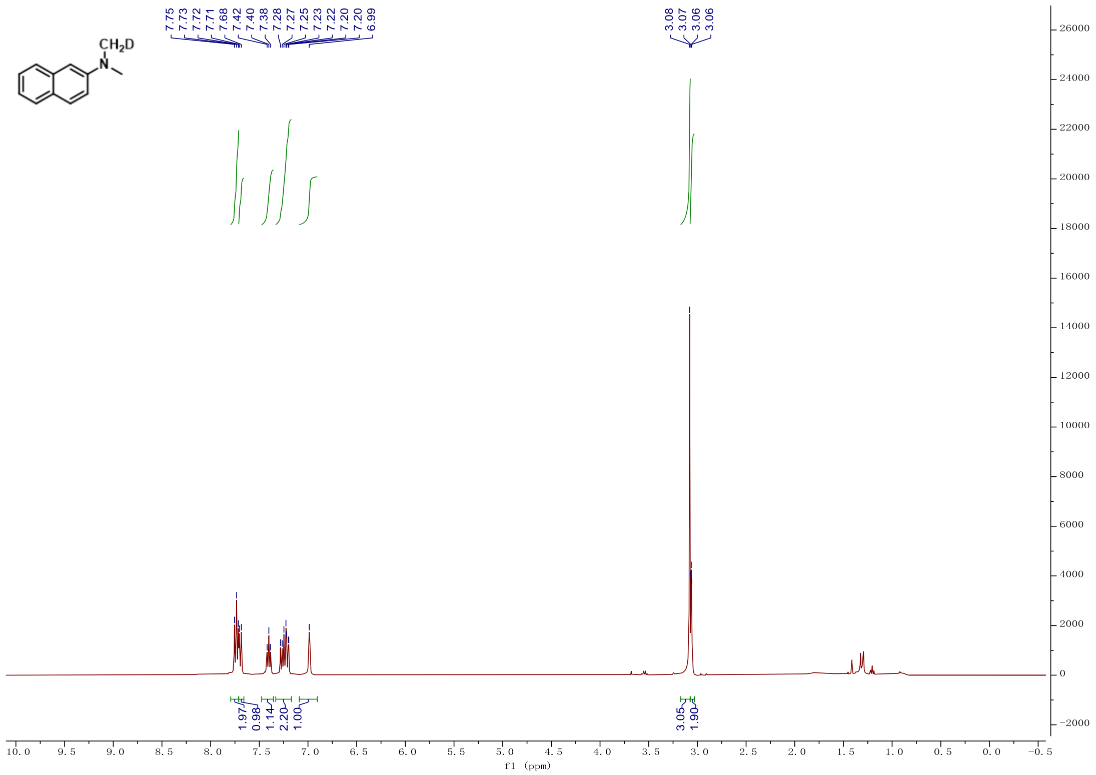

^{13}C NMR of Compound 65 (101 MHz, $CDCl_3$)

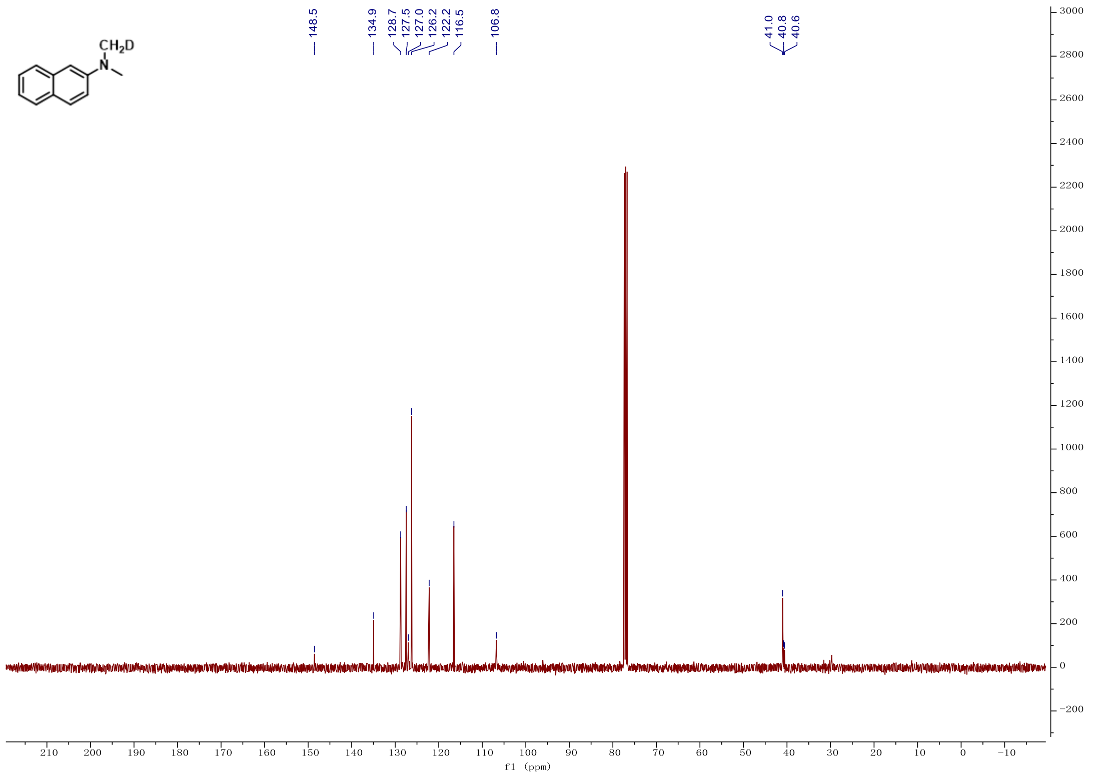

^{1}H NMR of Compound 66 (400 MHz, $CDCl_3$)

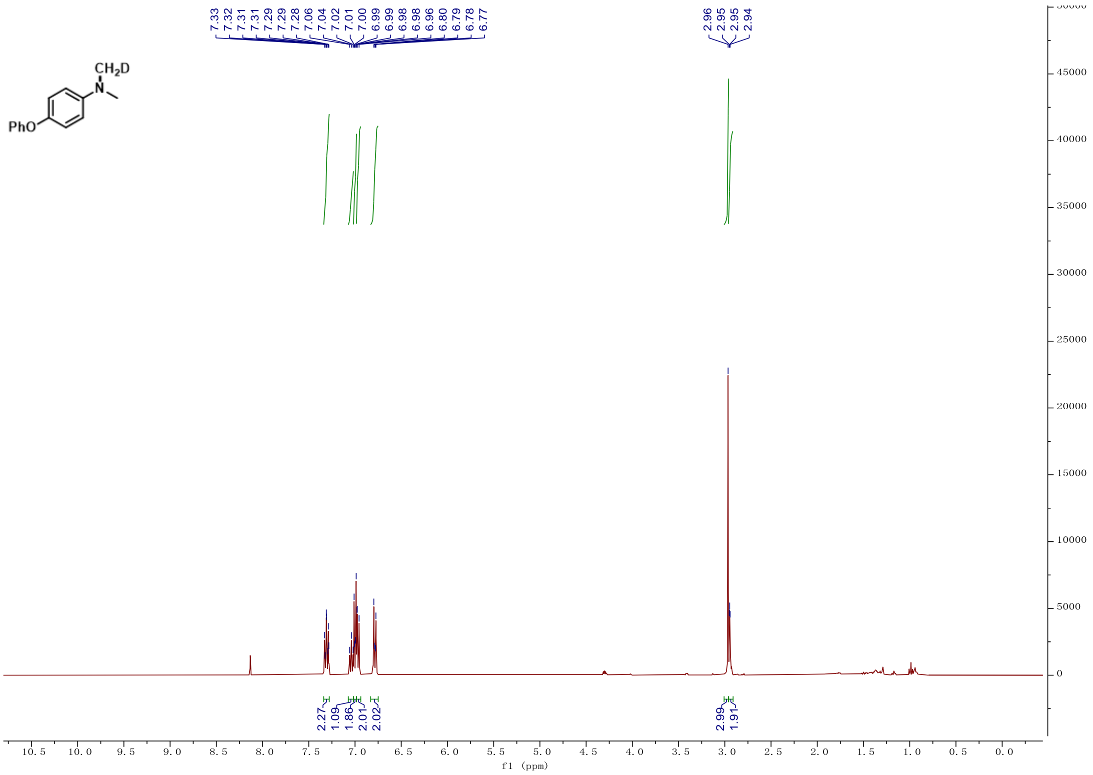

^{13}C NMR of Compound 66 (101 MHz, $CDCl_3$)

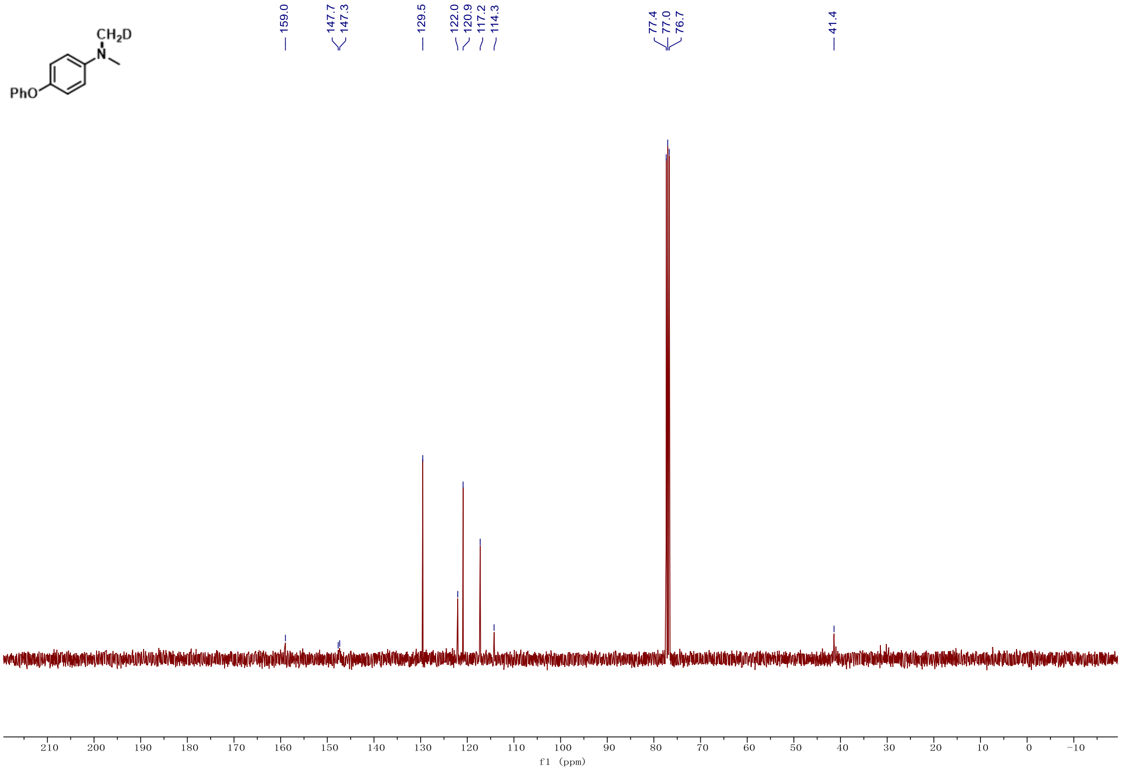

^{1}H NMR of Compound 67 (400 MHz, $CDCl_3$)

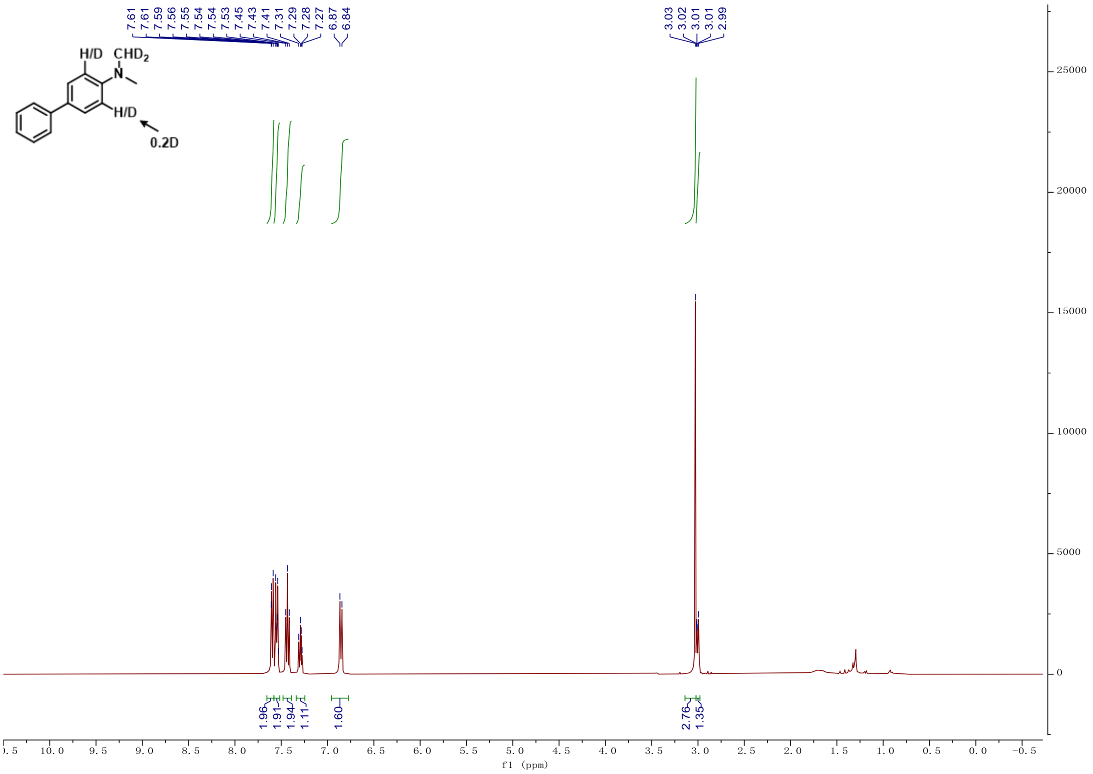

^{13}C NMR of Compound 67 (101 MHz, $CDCl_3$)

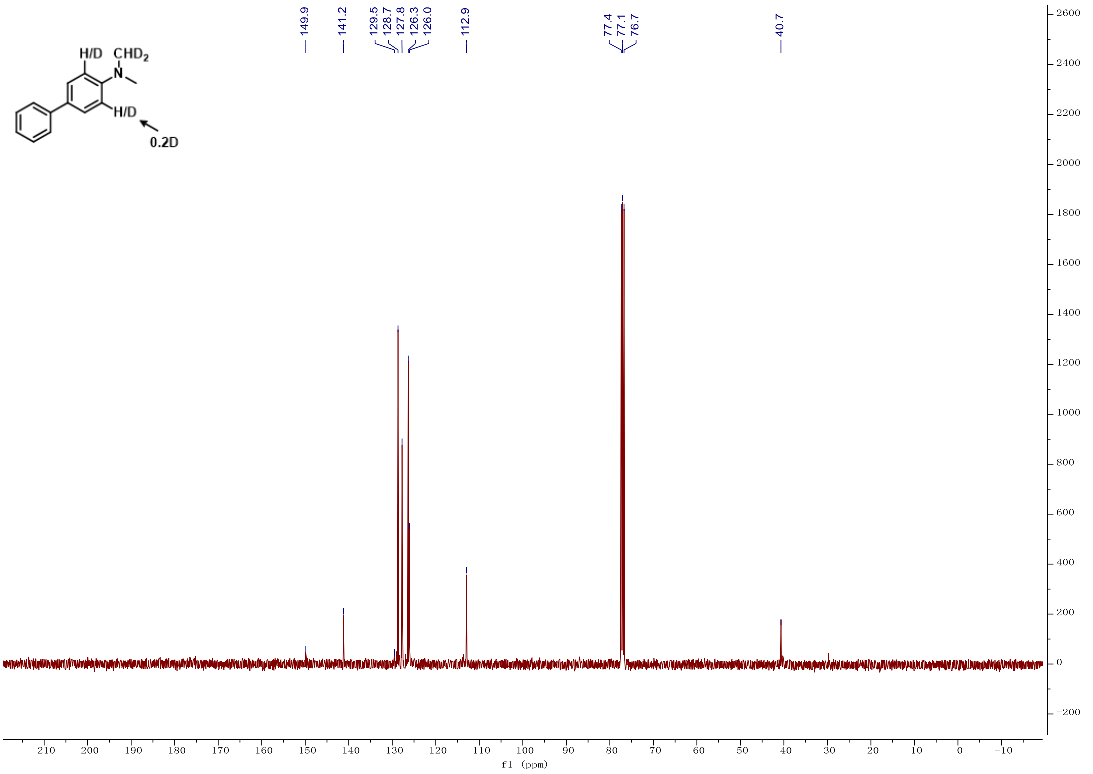

^{1}H NMR of Compound 68 (400 MHz, $CDCl_3$)

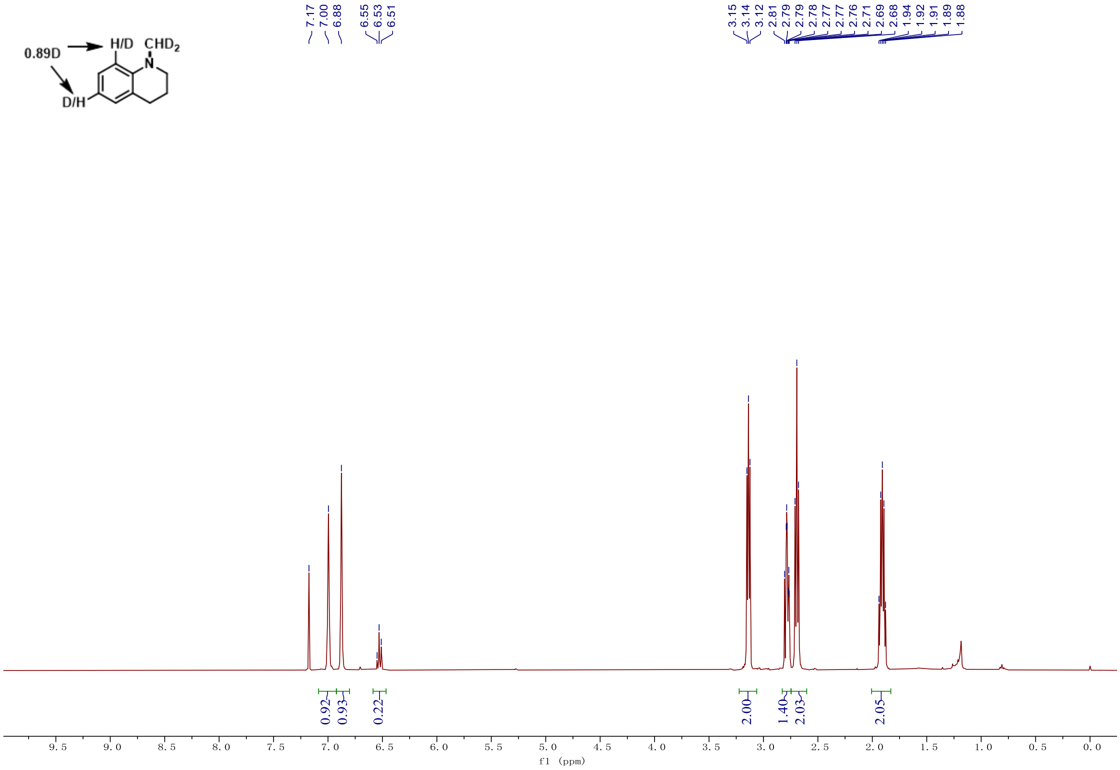

^{13}C NMR of Compound 68 (101 MHz, $CDCl_3$)

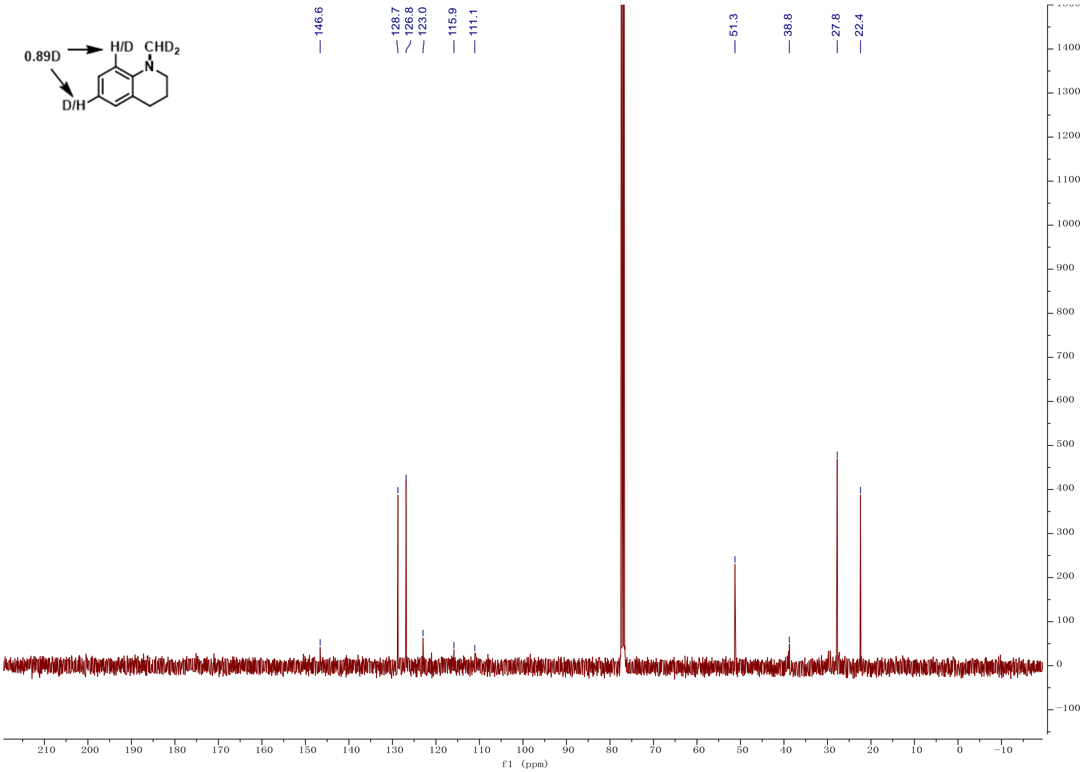

^{1}H NMR of Compound 69 (400 MHz, $CDCl_3$)

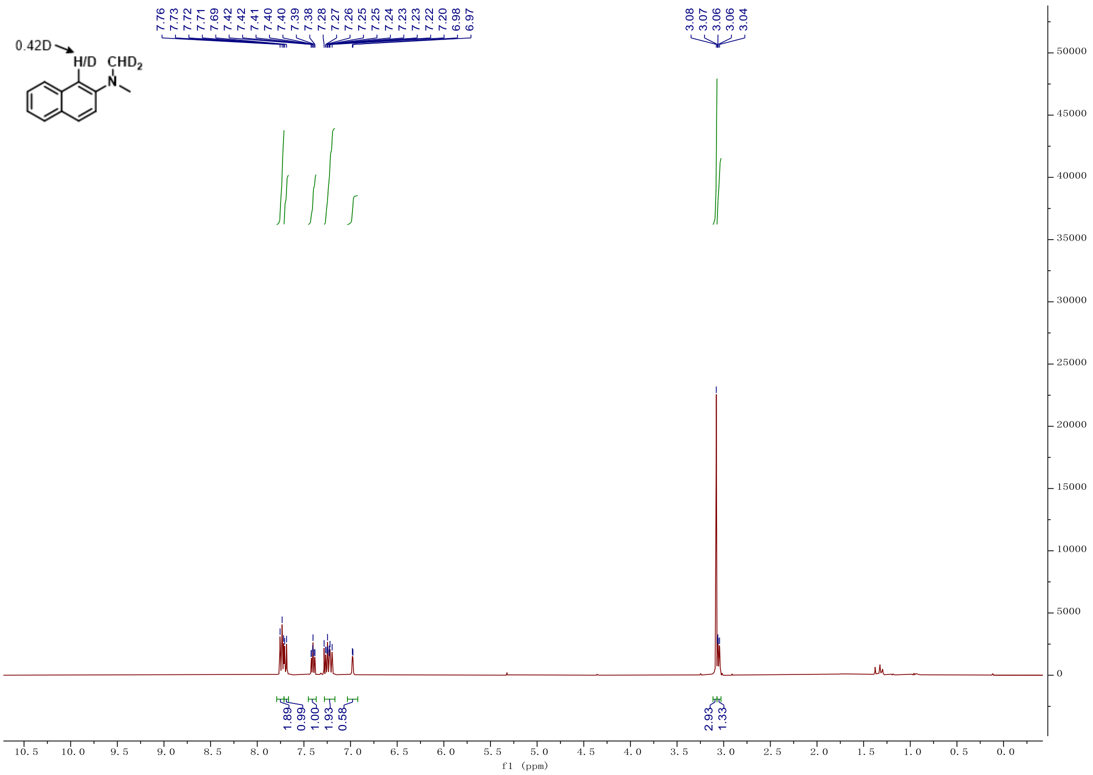

^{13}C NMR of Compound 69 (101 MHz, $CDCl_3$)

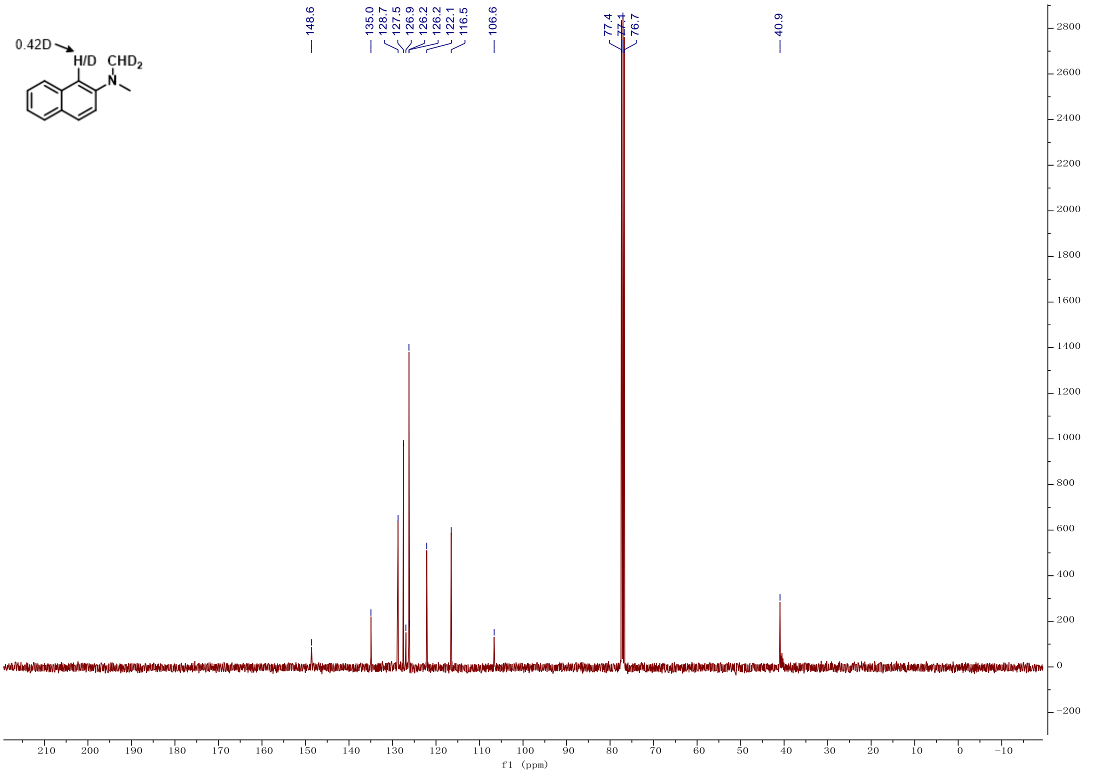

^{1}H NMR of Compound 70 (400 MHz, $CDCl_3$)

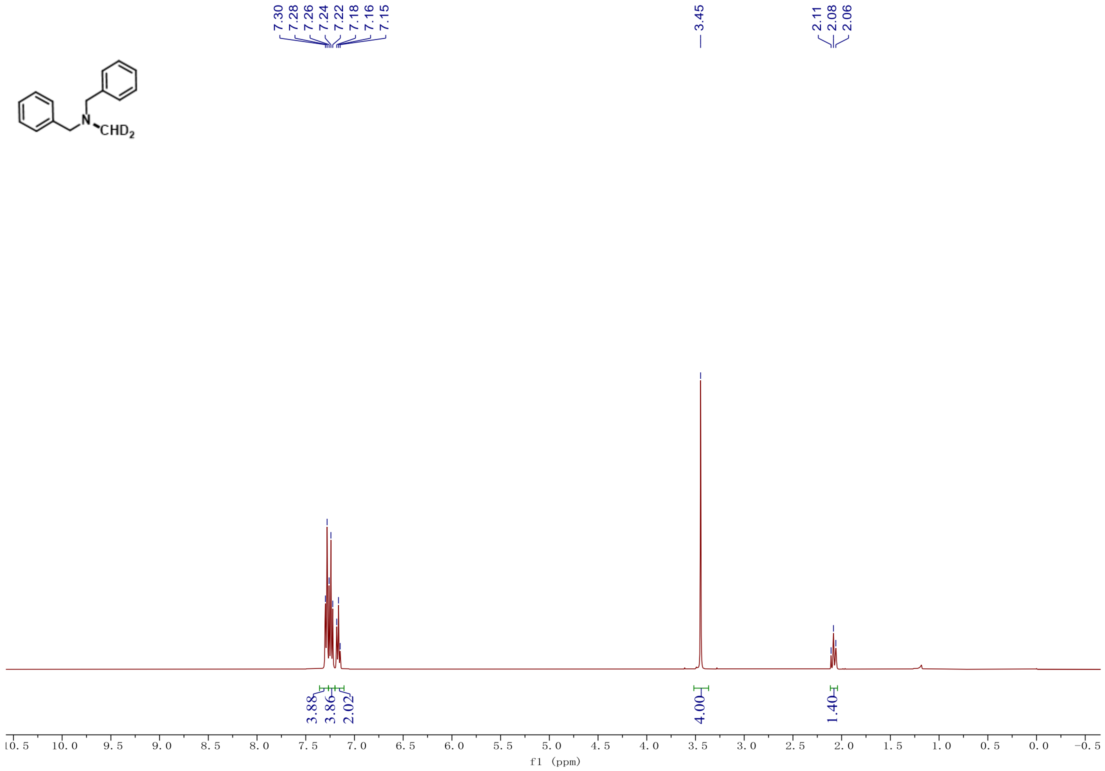

^{13}C NMR of Compound 70 (101 MHz, $CDCl_3$)

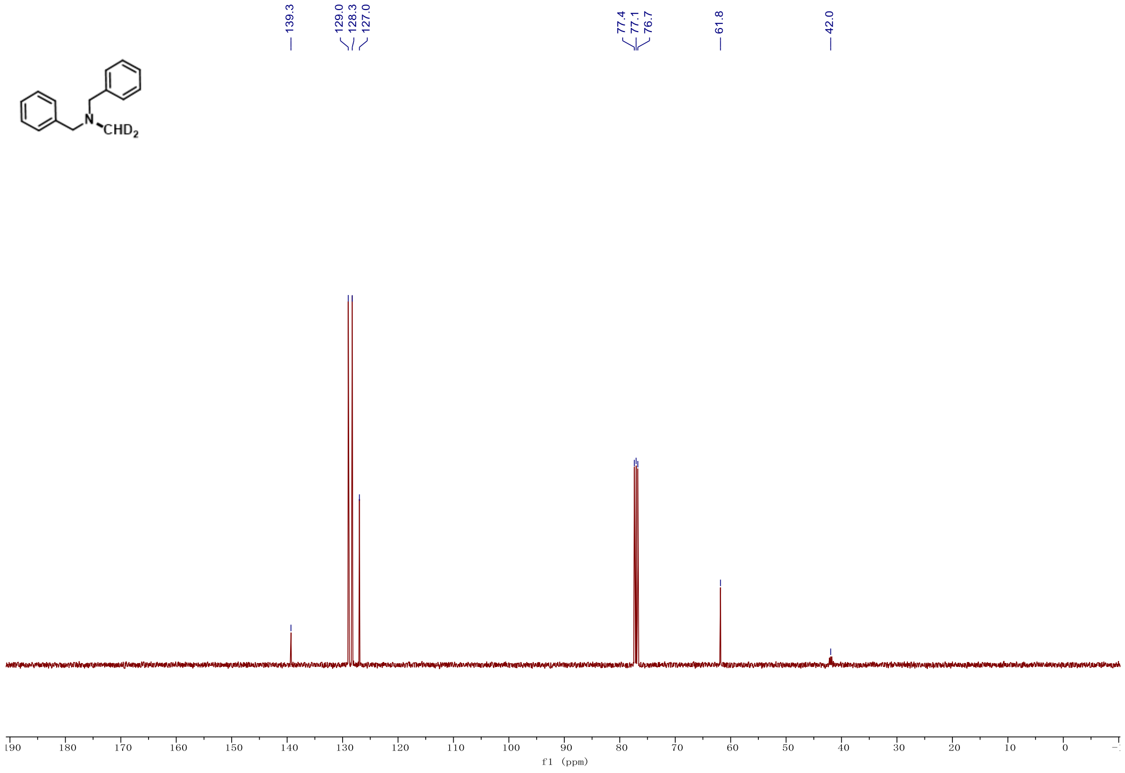

^{1}H NMR of Compound 71 (400 MHz, $CDCl_3$)

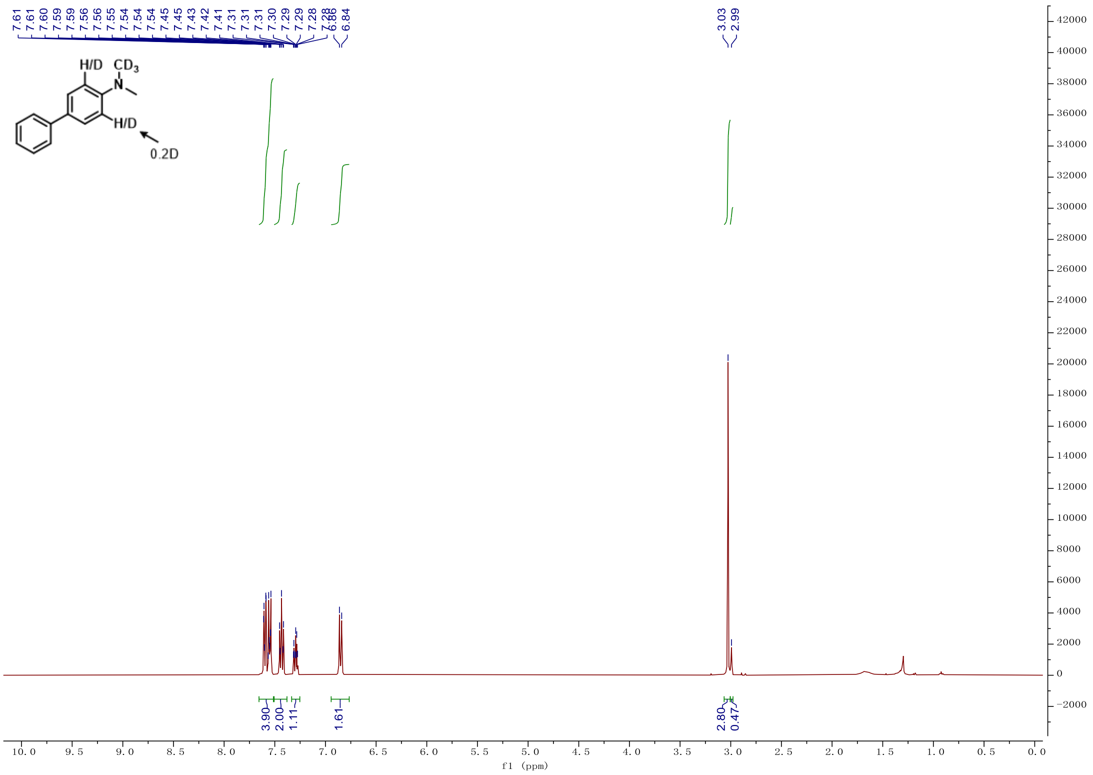

^{13}C NMR of Compound 71 (101 MHz, $CDCl_3$)

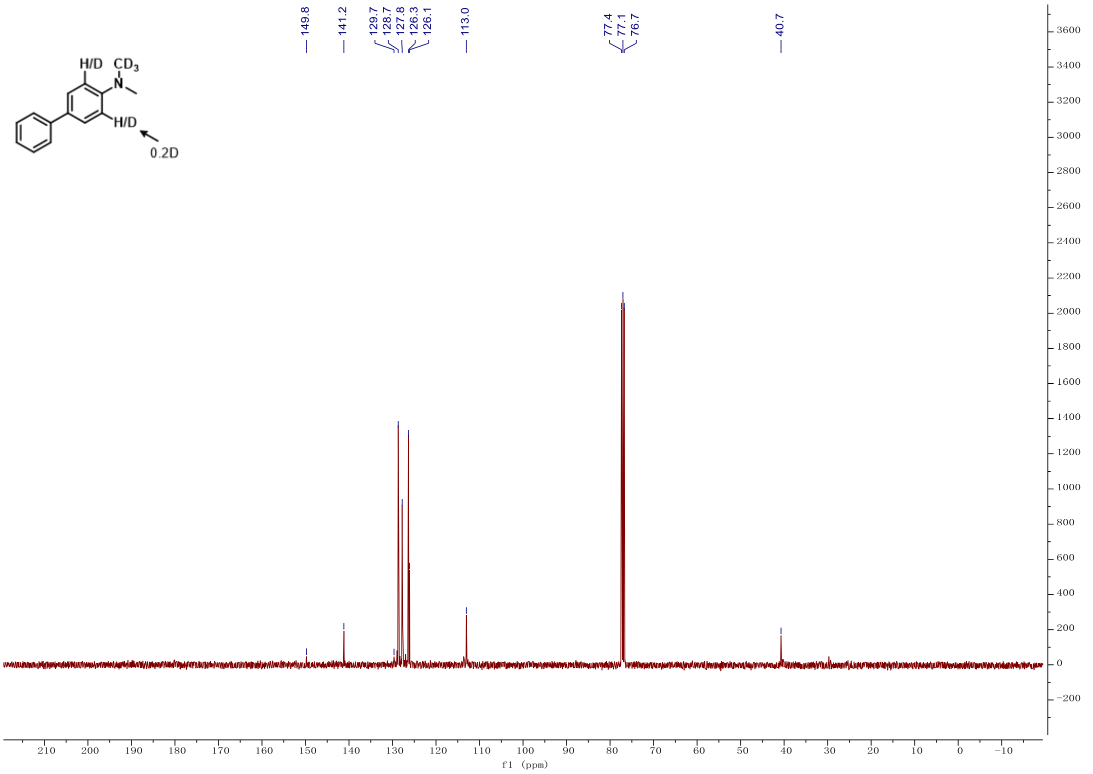

^{1}H NMR of Compound 72 (400 MHz, $CDCl_3$)

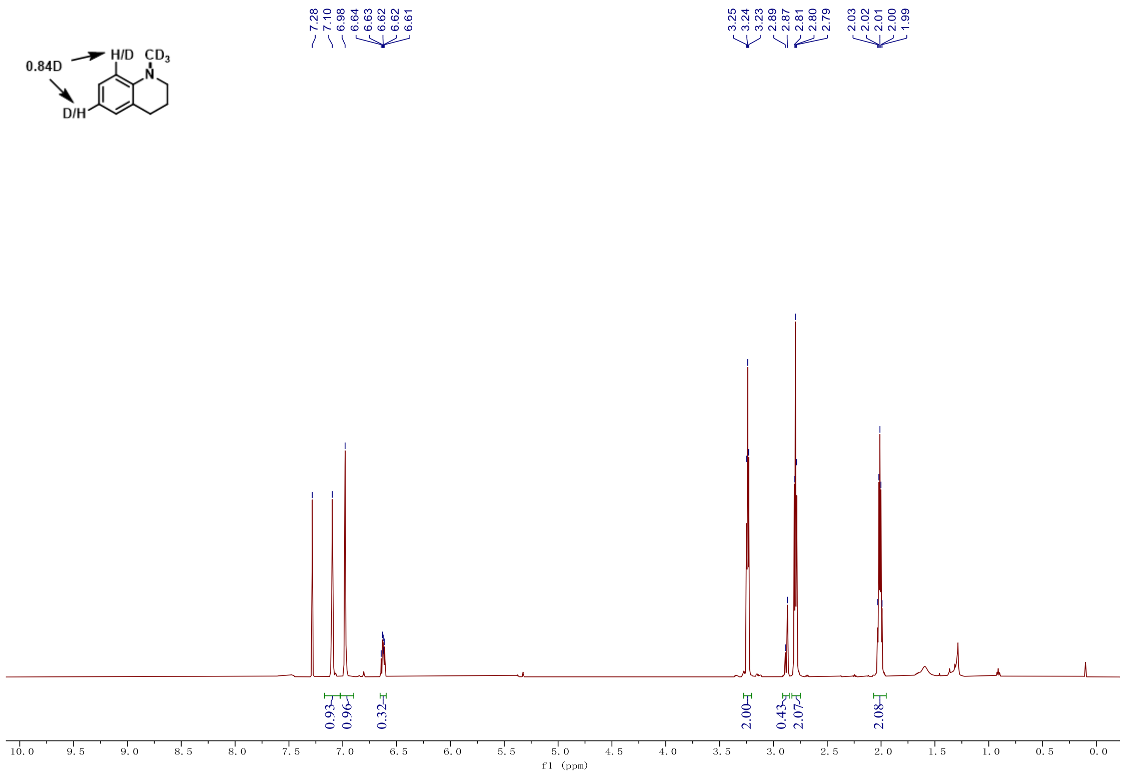

^{13}C NMR of Compound 72 (101 MHz, $CDCl_3$)

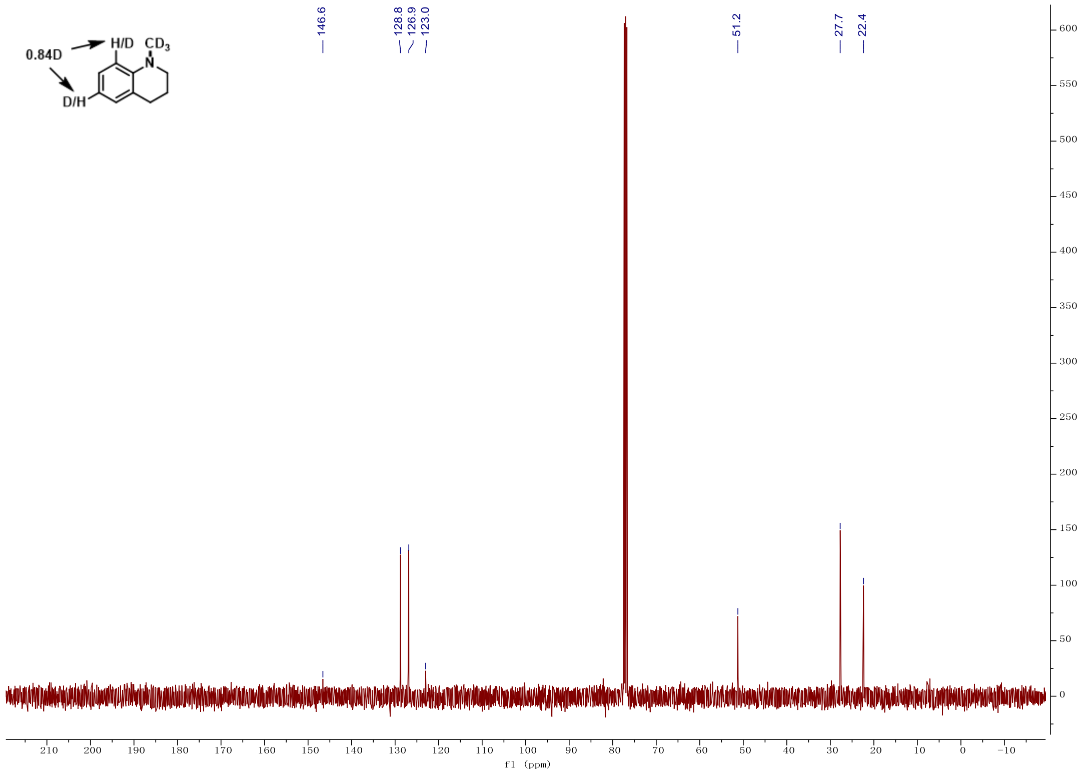

^{1}H NMR of Compound 73 (400 MHz, $CDCl_3$)

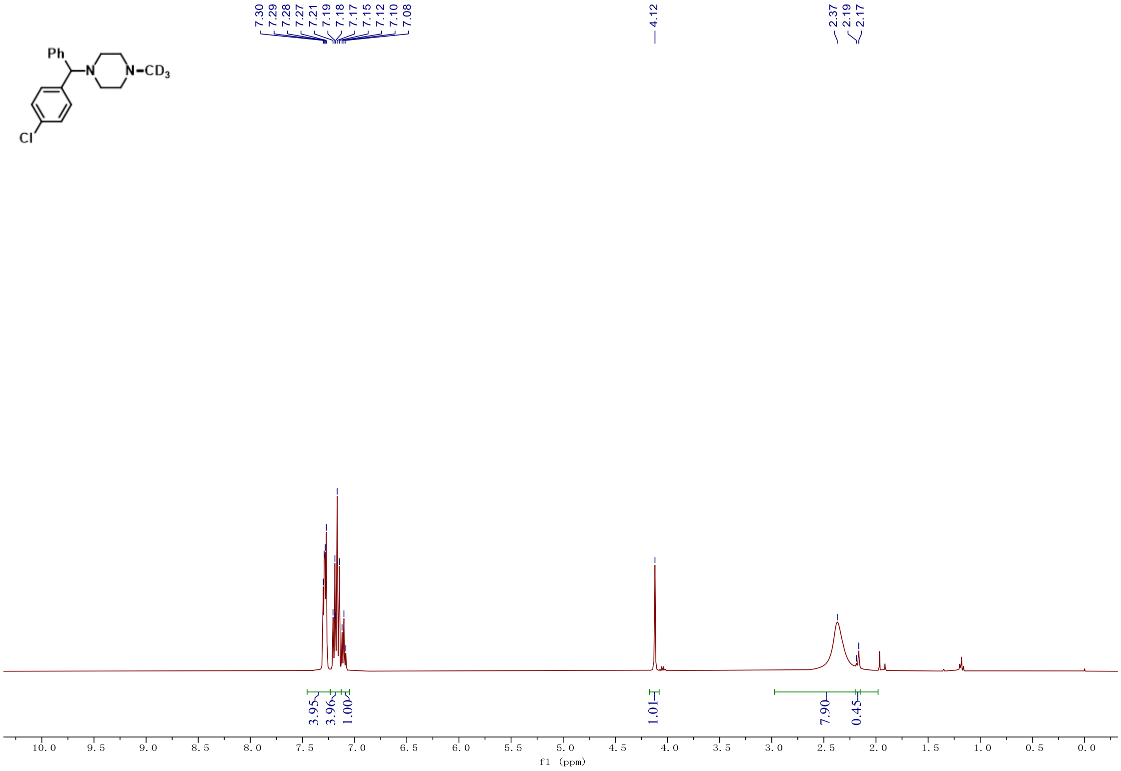

^{13}C NMR of Compound 73 (101 MHz, $CDCl_3$)

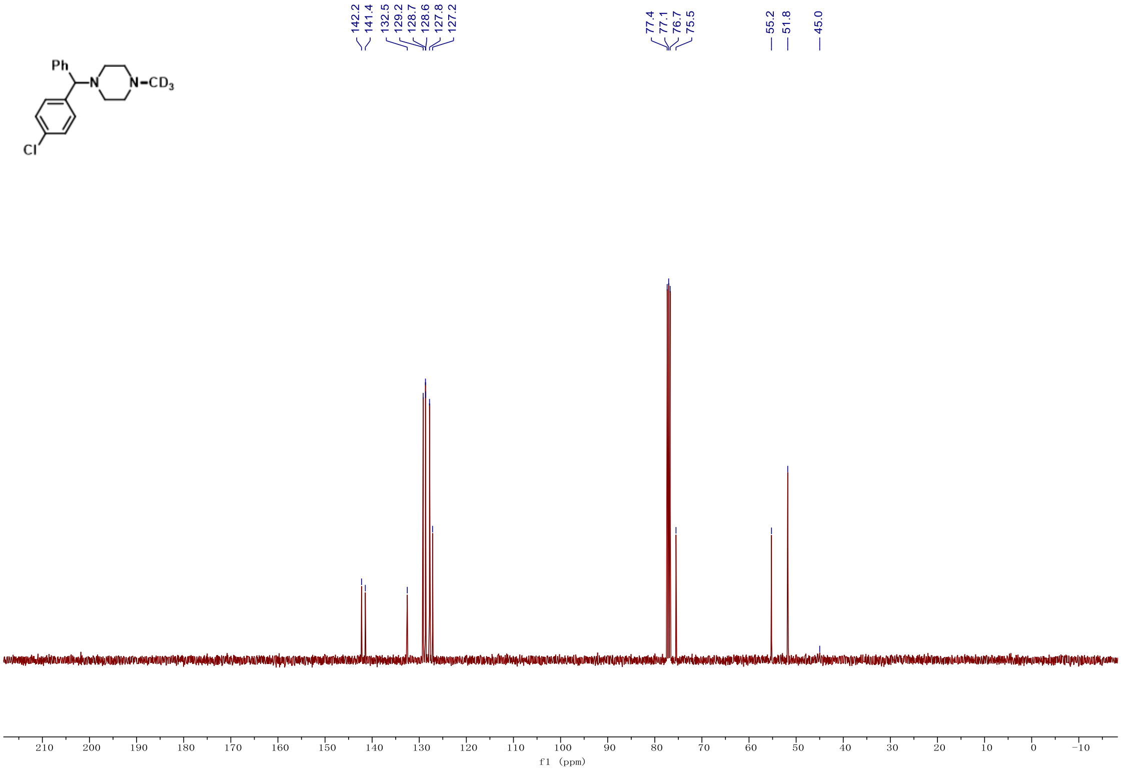

^1H NMR of Compound 74 (400 MHz, $CDCl_3$)

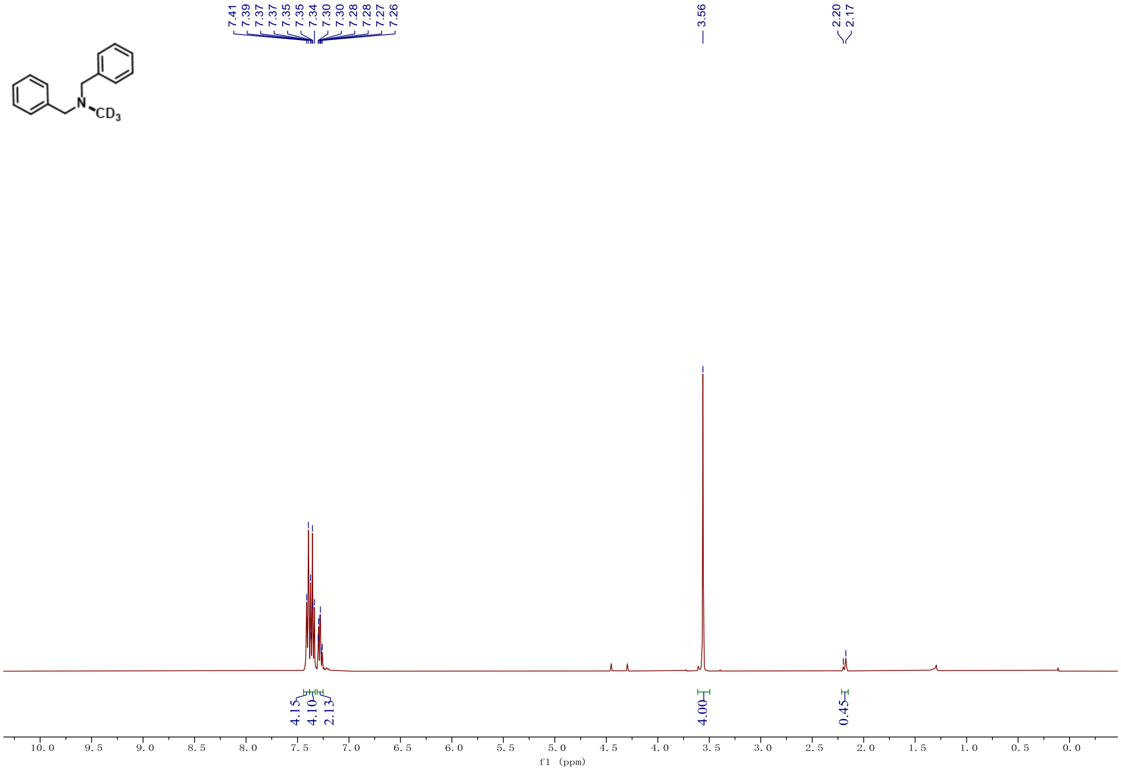

^{13}C NMR of Compound 74 (101 MHz, $CDCl_3$)

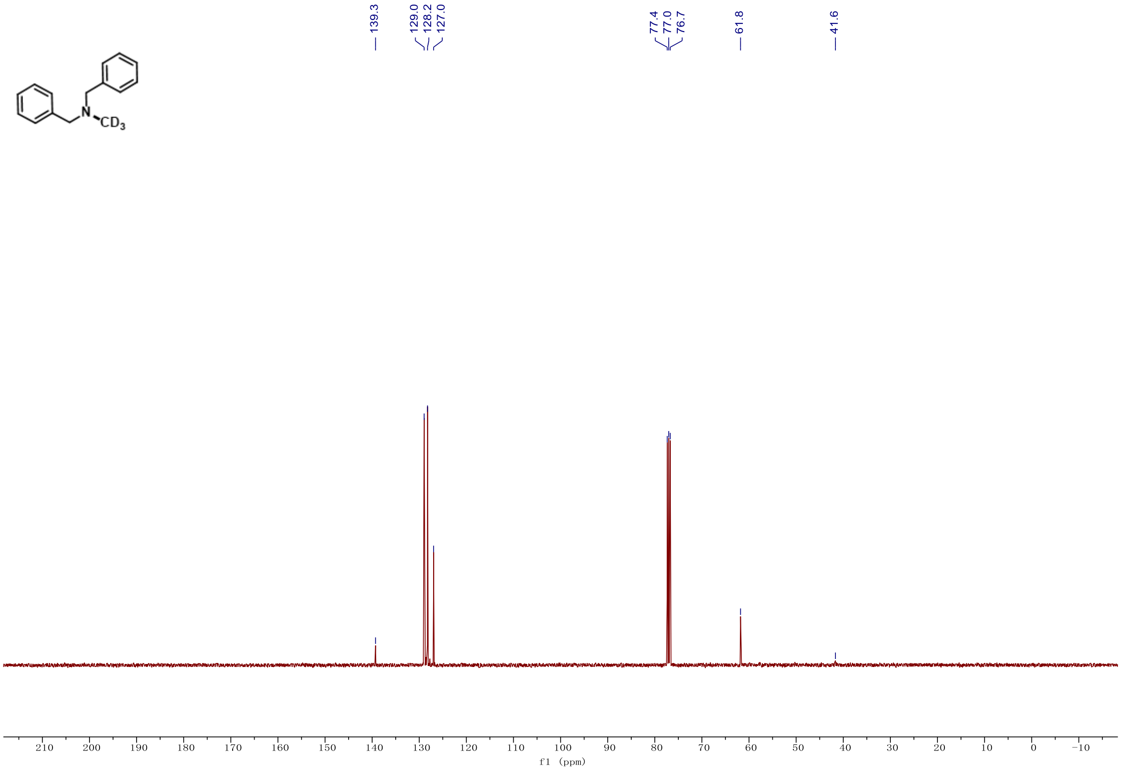

^{1}H NMR of Compound A1 (400 MHz, $CDCl_3$)

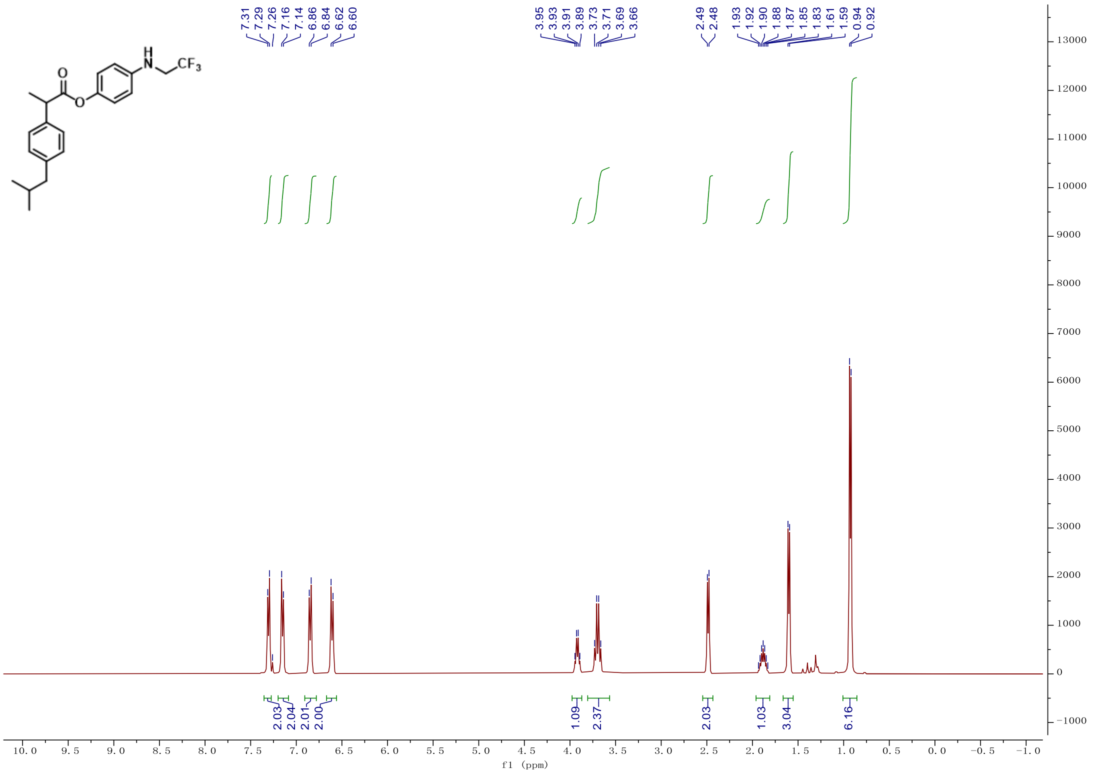

^{13}C NMR of Compound A1 (101 MHz, $CDCl_3$)

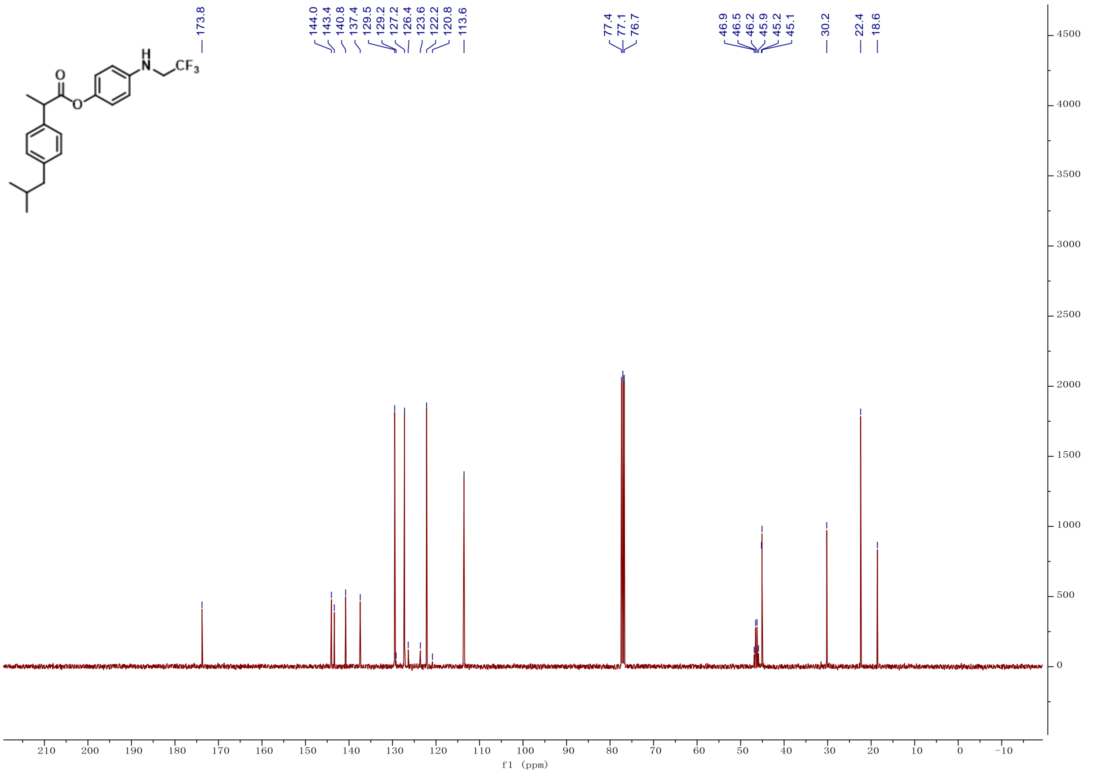

^{19}F NMR of Compound A1 (376 MHz, $CDCl_3$)

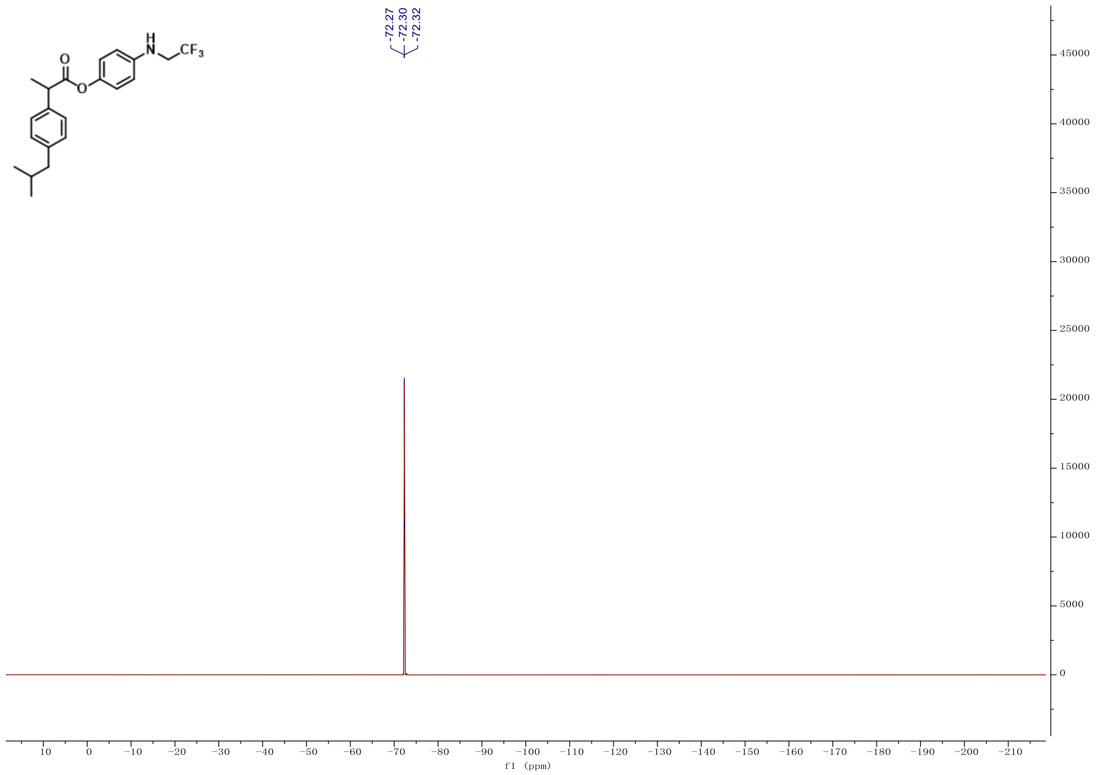

^1H NMR of Compound A2 (400 MHz, $CDCl_3$)

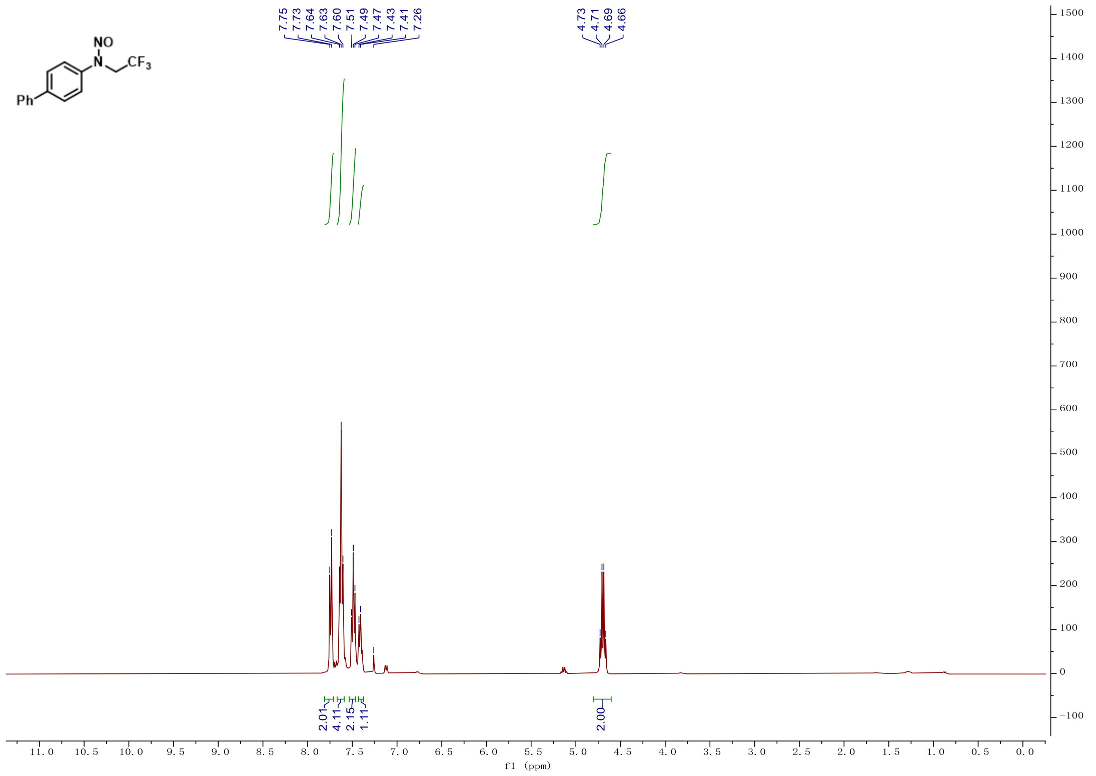

^{13}C NMR of Compound A2 (101 MHz, $CDCl_3$)

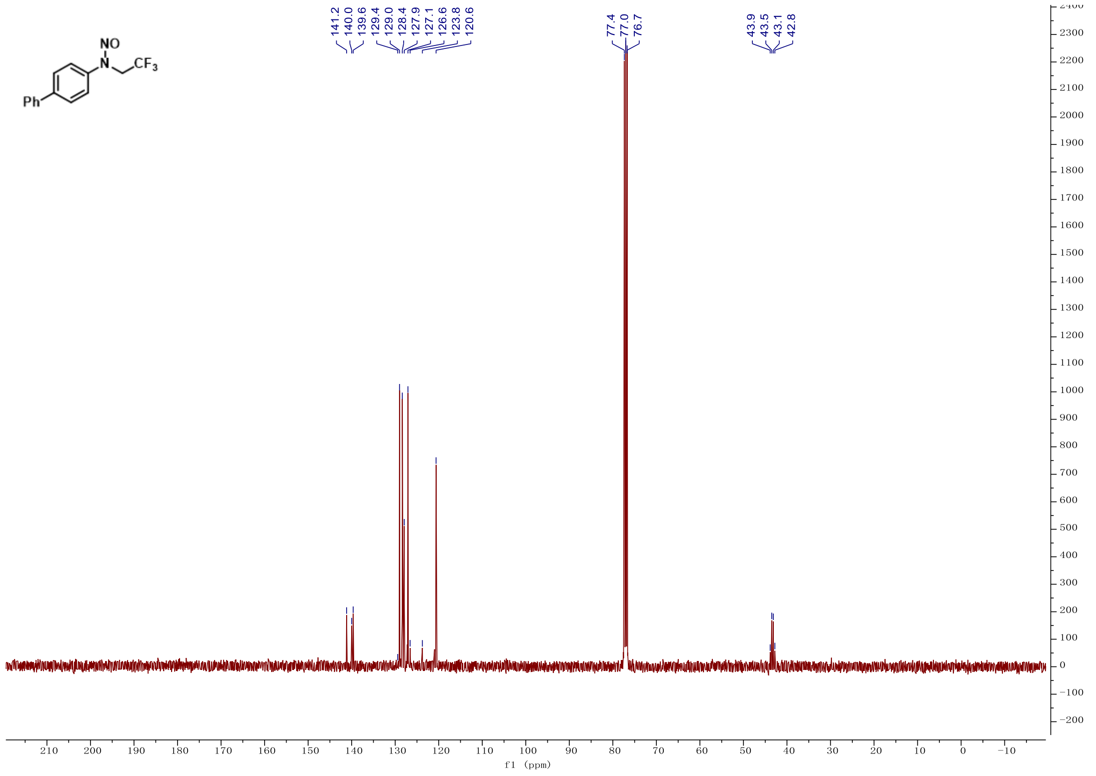

^{19}F NMR of Compound A2 (376 MHz, $CDCl_3$)

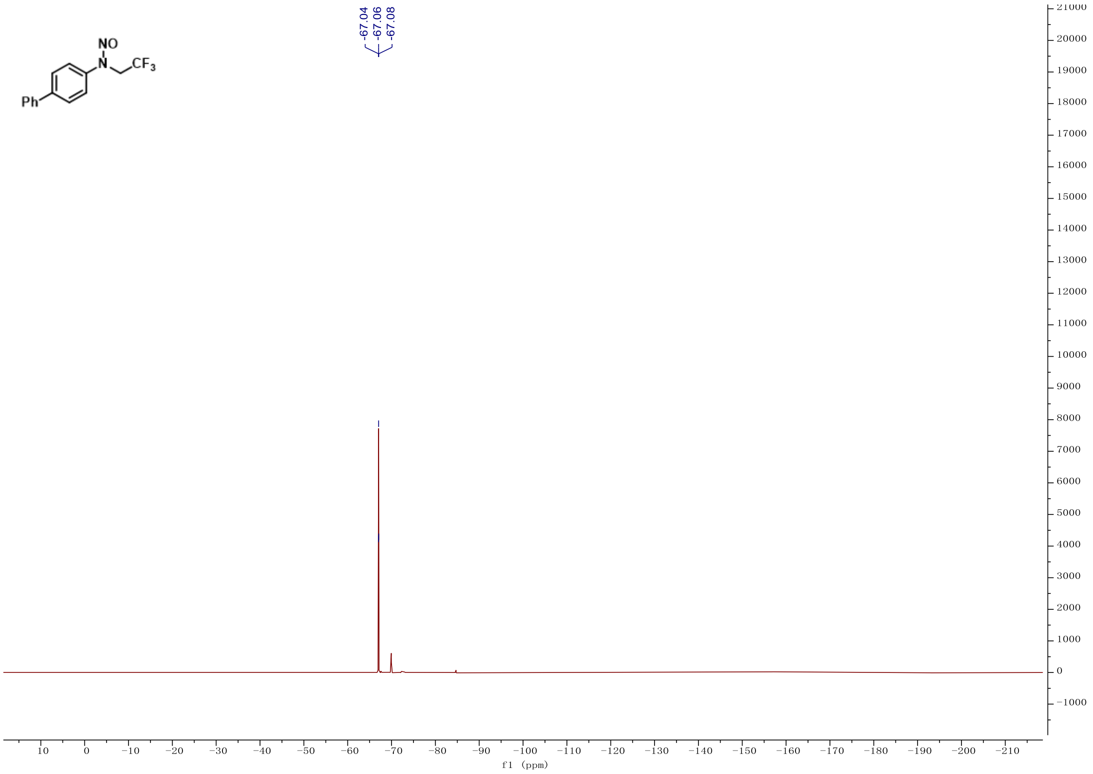

^{1}H NMR of Compound A3 (400 MHz, $CDCl_3$)

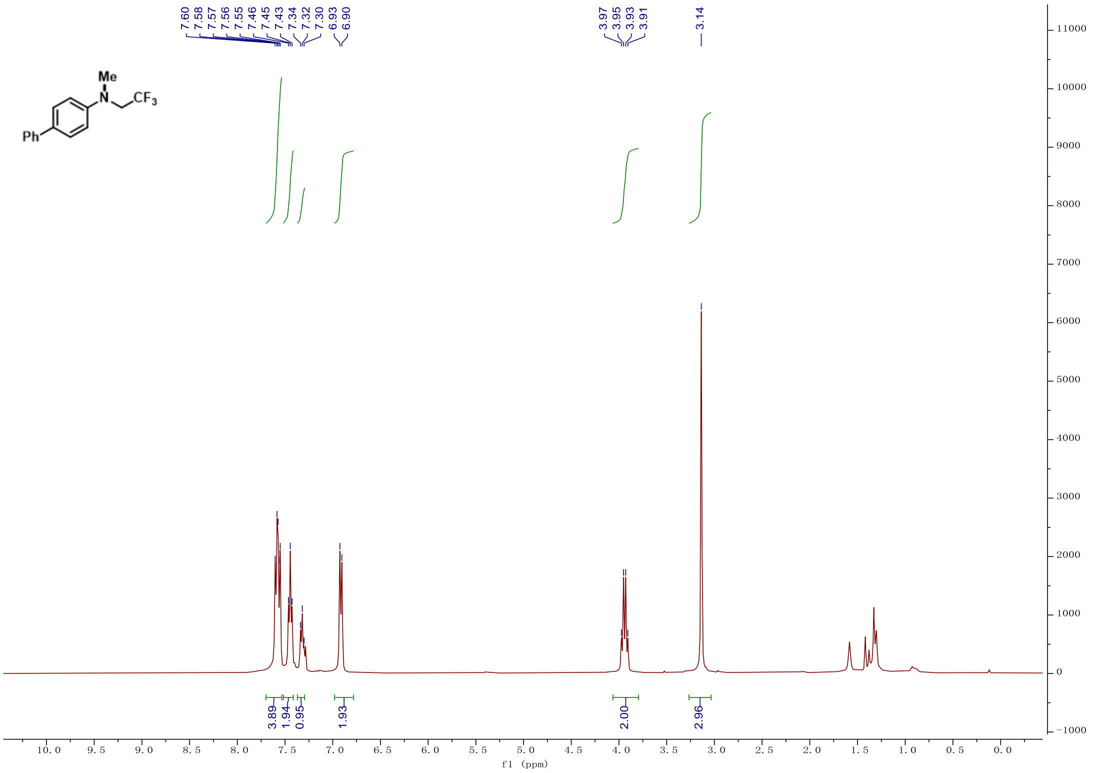

^{13}C NMR of Compound A3 (101 MHz, $CDCl_3$)

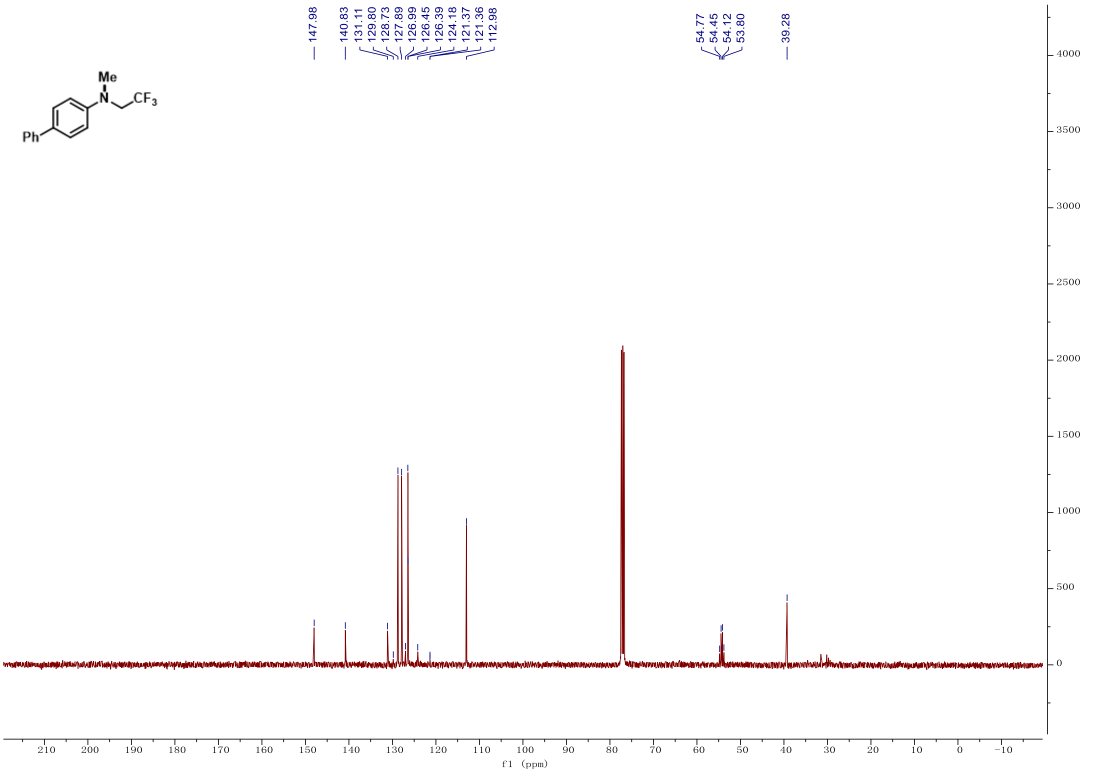

^{19}F NMR of Compound A3 (376 MHz, $CDCl_3$)

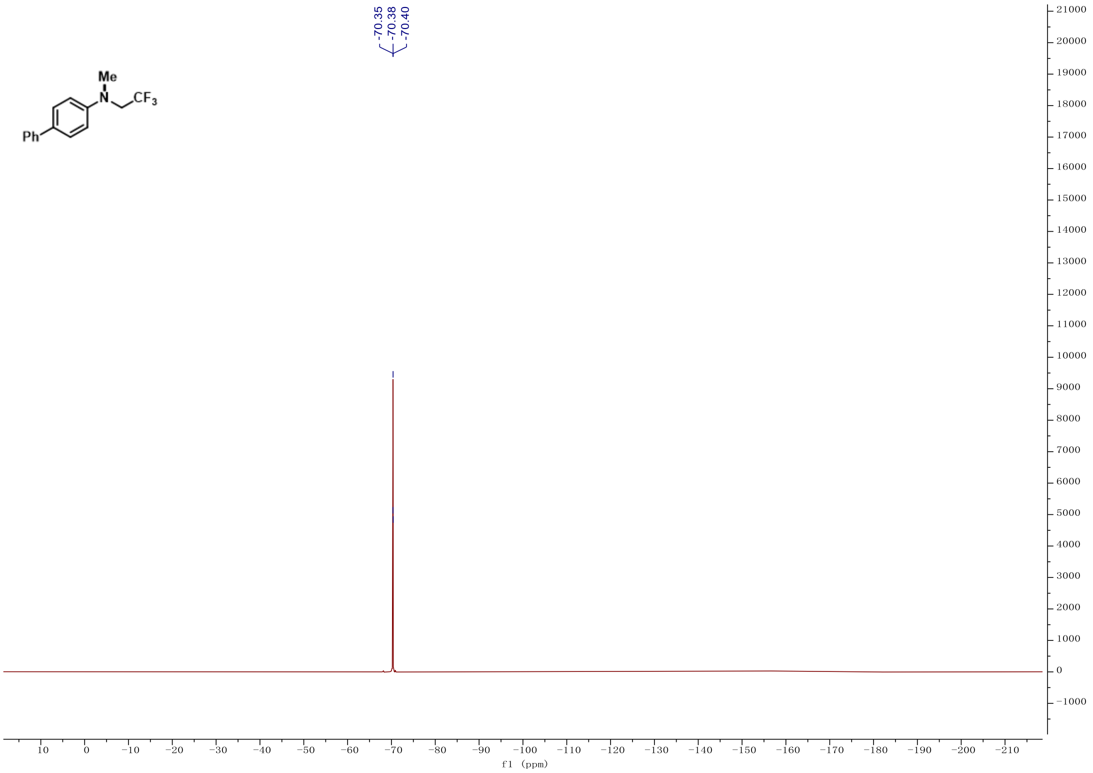

^{1}H NMR of Compound A4 (400 MHz, $CDCl_3$)

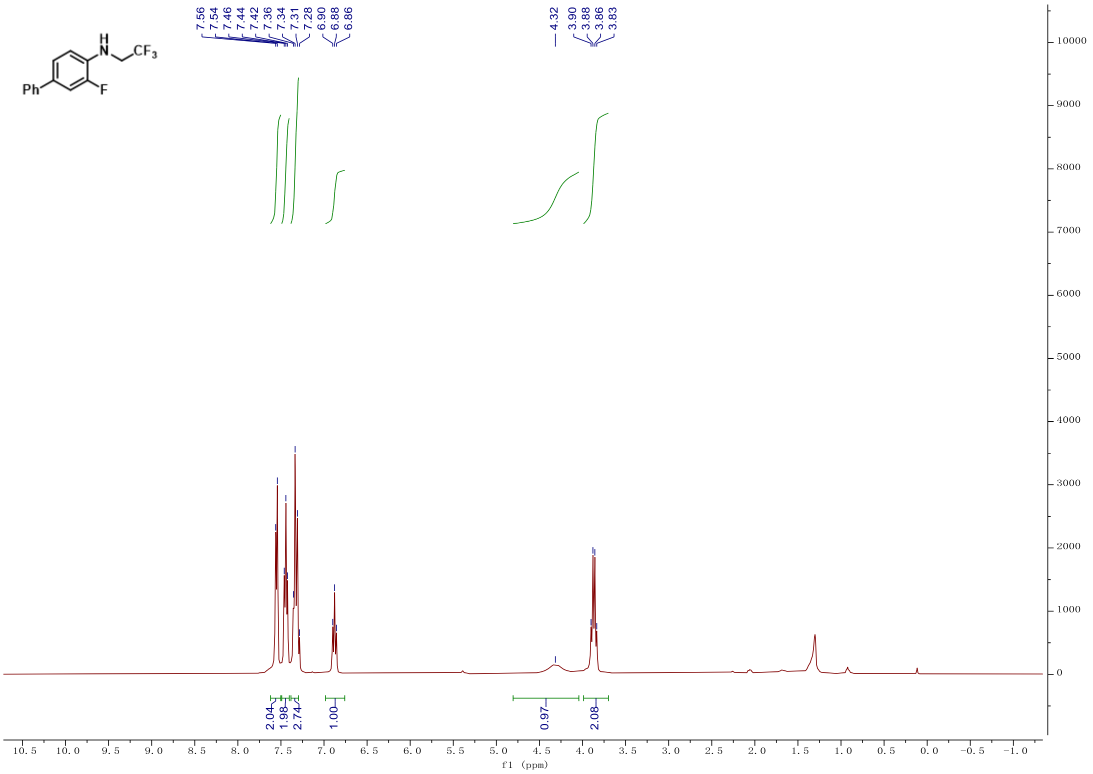

^{13}C NMR of Compound A4 (101 MHz, $CDCl_3$)

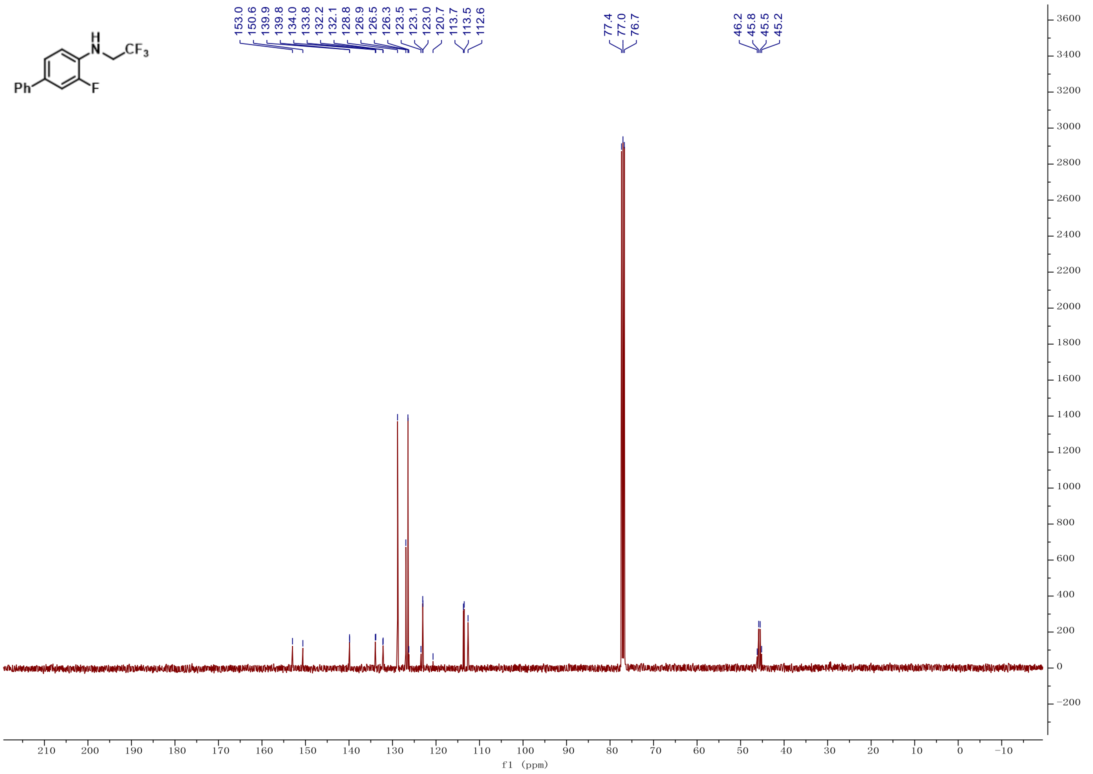

^{19}F NMR of Compound A4 (376 MHz, $CDCl_3$)

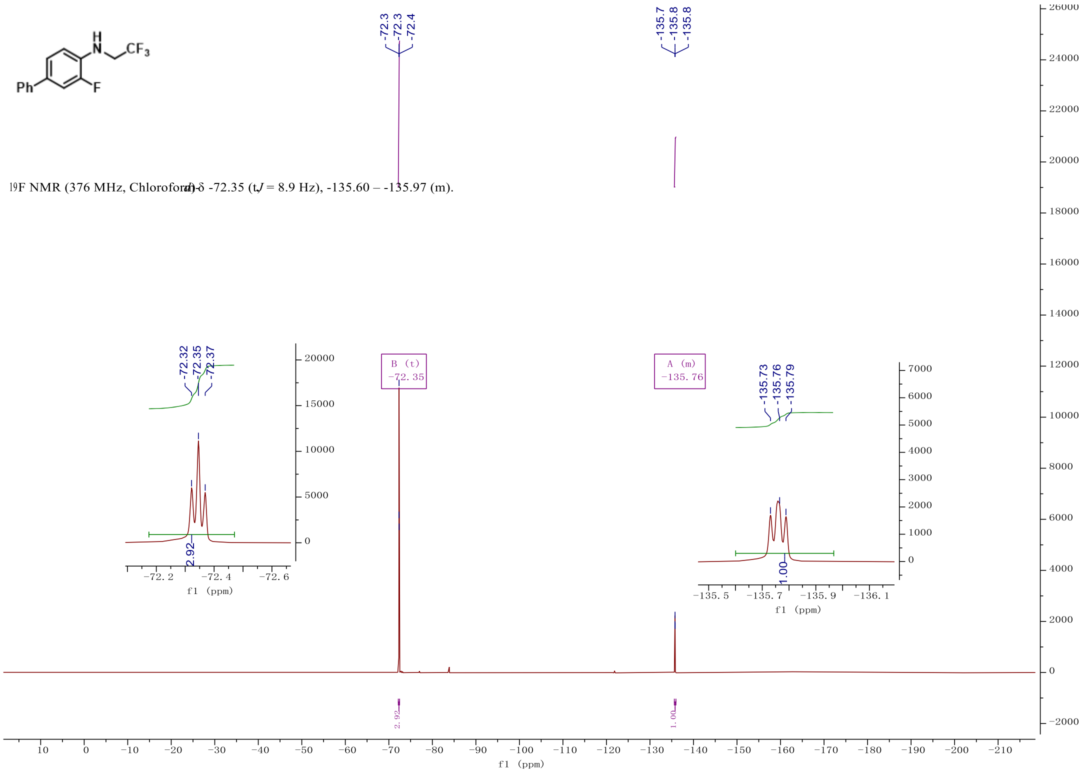

4. Supplementary References

1. Chaudhary, P., Gupta, S., Muniyappan, N., Sabiah, S. & Kandasamy, J. An efficient synthesis of *N*-nitrosamines under solvent, metal and acid free conditions using *tert*-butyl nitrite. *Green Chem.* **18**, 2323–2330 (2016).
2. Szpera, R., Moseley, D. F. J., Smith, L. B., Sterling, A. J. & Gouverneur, V. The fluorination of C–H bonds: developments and perspectives. *Angew. Chem. Int. Ed.* **58**, 14824–14848 (2019).
3. Pagar, V. V. & RajanBabu, T. V. Tandem catalysis for asymmetric coupling of ethylene and enynes to functionalized cyclobutanes. *Science* **361**, 68–72 (2018).
4. Frisch, M. J. et al. *Gaussian 16*, *Revision C.01* (Gaussian, Inc., Wallingford, CT, 2019).
5. Becke, A. D. Density-functional thermochemistry. III. The role of exact exchange. *J. Chem. Phys.* **98**, 5648–5652 (1993).
6. Grimme, S. et al. A consistent and accurate *ab initio* parametrization of density functional dispersion correction (DFT-D) for the 94 elements H-Pu. *J. Chem. Phys.* **132**, 154104 (2010).
7. Lee, C., Yang, W. & Parr, R. G. Development of the Colle-Salvetti correlation-energy formula into a functional of the electron density. *Phys. Rev. B* **37**, 785–789 (1988).
8. Grimme, S., Ehrlich, S. & Goerigk, L. Effect of the damping function in dispersion corrected density functional theory. *J. Comput. Chem.* **32**, 1456–1465 (2011).
9. Zhao, Y. & Truhlar, D. G. The M06 suite of density functionals for main group thermochemistry, thermochemical kinetics, noncovalent interactions, excited states, and transition elements. *Theor. Chem. Acc.* **120**, 215–241 (2008).
10. Marenich, A. V., Cramer, C. J. & Truhlar, D. G. Universal solvation model based on solute electron density and on a continuum model of the solvent defined by the bulk dielectric constant and atomic surface tensions. *J. Phys. Chem. B* **113**, 6378–6396 (2009).
11. Winget, P., Dolney, D. M., Giesen, D. J., Cramer, C. J. & Truhlar, D. G. *Minnesota Solvent Descriptor Database* (Department of Chemistry and Supercomputer Institute, University of Minnesota, Minneapolis, 1999).
12. Wang, H., Tu, Y. H., Liu, D. Y. & Hu, X.-G. Cu-Catalyzed/mediated synthesis of *N*-fluoroalkylanilines from arylboronic acids: fluorine effect on the reactivity of fluoroalkylamines. *Org. Biomol. Chem.* **16**, 6634–6637 (2018).
13. Chen, S., Wang, H., Jiang, W., Rui, P.-X. & Hu, X.-G. Synthesis of tri(di)fluoroethylanilines via copper-catalyzed coupling reaction of tri(di)fluoroethylamine with (hetero)aromatic bromides. *Org. Biomol. Chem.* **17**, 9799–9807 (2019).
14. Xin, J. & Leng, F. *N*-Arylation of fluoroalkylamine and trifluoroacetamide through Cu-catalysis. *Chem. Sel.* **4**, 12124–12127 (2019).
15. Brusoe, A. T. & Hartwig, J. F. Palladium-catalyzed arylation of fluoroalkylamines. *J. Am. Chem. Soc.* **137**, 8460–8468 (2015).
16. Pan, Y. et al. B(C6F5)3-catalyzed deoxygenative reduction of amides to amines with ammonia borane. *Adv. Synth. Catal.* **361**, 2301–2308 (2019).
17. Ma, S. S. et al. Co(dppbsa)-catalyzed reductive *N*,*N*-dimethylation of nitroaromatics with CO2 and hydrosilane. *Green Chem.* **25**, 8625–8632 (2023).
18. Ma, Z. et al. Development of iron-based single atom materials for general and efficient synthesis of amines. *Angew. Chem. Int. Ed.* **63**, e202407859 (2024).
19. Borthakur, I., Nandi, S., Bilora, Y., Sadhu, B. & Kundu, S. Reductive aminomethylation using

ammonium formate and methanol as N1 and C1 source: direct synthesis of mono- and di-methylated amines. *ACS Catal.* **14**, 5847–5857 (2024).
20. Lu, C., Qiu, Z., Zhu, Y. & Lin, B.-L. Scalable direct *N*-methylation of drug-like amines using 12CO2/13CO2 by simple inorganic base catalysis. *Sci. Bull.* **64**, 723–729 (2019).
21. Sarita, S. P., Akhilesh, R. & Sanjay, B. W. Synthesis and evaluation of some new 11-[(N4-substituted)-1'-piperazinyl] dibenz [b,f][1,4]-thiazepines for antiparkinson activity. *J. Pharm. Res.* **5**, 3726–3730 (2012).
22. Khan, J., Taneja, N., Yadav, N. & Hazra, C. K. Silane-mediated, facile C–H and N–H methylation using formaldehyde. *Chem. Commun.* **60**, 11367–11370 (2024).
23. Concha-Puelles, M. A., Torres-González, S., Robles-Henríquez, R. & Luehr, S. Ruthenium-catalyzed selective mono *N*-ethylation of arylamines and tandem reduction/*N*-ethylation of nitroarenes using triethylamine and formic acid. *J. Org. Chem.* **89**, 8773–8781 (2024).
24. Du, Y. et al. Strongly reducing, visible-light organic photoredox catalysts as sustainable alternatives to precious metals. *Chem. Eur. J.* **23**, 10962–10968 (2017).
25. Hermant, F. et al. Reductive alkylation of thioamides with Grignard reagents in the presence of Ti(OiPr)4: insight and extension. *Organometallics* **33**, 5643–5653 (2014).
26. He, S. et al. Discovery, optimization, and characterization of novel chlorcyclizine derivatives for the treatment of hepatitis C virus infection. *J. Med. Chem.* **59**, 841–853 (2016).
27. Zhang, Z. et al. Semiconductor photocatalysis to engineering deuterated *N*-alkyl pharmaceuticals enabled by synergistic activation of water and alkanols. *Nat. Commun.* **11**, 4722 (2020).
