# Supplementary material for: Electroreductive amination of carboxylic acids by cobalt catalysis
Source: Nat Commun. 2025 Aug 4;16:7167. doi: 10.1038/s41467-025-62396-4 (PMC12322302; doi:10.1038/s41467-025-62396-4)
Supplement: Supplementary file 2 — Description of Additional Supplementary Files [file 41467_2025_62396_MOESM2_ESM.pdf]

Description of Additional Supplementary Files

File Name: Supplementary Data 1

Description: The Cartesian coordinates of optimized geometries including starting materials, transition states, intermediates, and products associated with the DFT calculations are included in this file.
